# A Mortality Analysis of Letermovir Prophylaxis for Cytomegalovirus (CMV) in CMV-seropositive Recipients of Allogeneic Hematopoietic Cell Transplantation

**DOI:** 10.1093/cid/ciz490

**Published:** 2019-06-08

**Authors:** Per Ljungman, Michael Schmitt, Francisco M Marty, Johan Maertens, Roy F Chemaly, Nicholas A Kartsonis, Joan R Butterton, Hong Wan, Valerie L Teal, Kendra Sarratt, Yoshihiko Murata, Randi Y Leavitt, Cyrus Badshah

**Affiliations:** 1 Department of Cellular Therapy and Allogeneic Stem Cell Transplantation, Karolinska University Hospital and Karolinska Institutet, Stockholm, Sweden; 2 University of Heidelberg, Germany; 3 Dana-Farber Cancer Institute and Brigham and Women’s Hospital, Boston, Massachusetts; 4 Universitaire Ziekenhuizen Leuven, Belgium; 5 University of Texas, MD Anderson Cancer Center, Houston; 6 Merck & Co., Inc., Kenilworth, New Jersey

**Keywords:** letermovir, cytomegalovirus, mortality, hematopoietic cell transplantation

## Abstract

**Background:**

In a phase 3 trial, letermovir reduced clinically significant cytomegalovirus infections (CS-CMVi) and all-cause mortality at week 24 versus placebo in CMV-seropositive allogeneic hematopoietic cell transplantation (HCT) recipients. This post hoc analysis of phase 3 data further investigated the effects of letermovir on all-cause mortality.

**Methods:**

Kaplan-Meier survival curves were generated by treatment group for all-cause mortality. Observations were censored at trial discontinuation for reasons other than death or at trial completion. Hazard ratios (HRs) and 95% confidence intervals (CIs) were calculated using Cox modeling, adjusting for risk factors associated with mortality.

**Results:**

Of 495 patients with no detectable CMV DNA at randomization, 437 had vital-status data available through week 48 post-HCT at trial completion (101 deaths, 20.4%). Following letermovir prophylaxis, the HR for all-cause mortality was 0.58 (95% CI, 0.35–0.98; *P* = .04) at week 24 and 0.74 (95% CI, 0.49–1.11; *P* = .14) at week 48 post-HCT versus placebo. Incidence of all-cause mortality through week 48 post-HCT in the letermovir group was similar in patients with or without CS-CMVi (15.8 vs 19.4%; *P = *.71). However, in the placebo group, all-cause mortality at week 48 post-HCT was higher in patients with versus those without CS-CMVi (31.0% vs 18.2%; *P* = .02). The HR for all-cause mortality in patients with CS-CMVi was 0.45 (95% CI, 0.21–1.00; *P* = .05) at week 48 for letermovir versus placebo.

**Conclusions:**

Letermovir may reduce mortality by preventing or delaying CS-CMVi in HCT recipients.

**Clinical Trials Registration:**

clinicaltrials.gov, NCT02137772.

## Teaser

This post hoc mortality analysis of phase 3 data suggests that letermovir prophylaxis reduced all-cause mortality versus placebo in cytomegalovirus (CMV)-seropositive allogeneic hematopoietic-cell transplantation recipients by preventing or delaying clinically significant CMV infections.

**(See the Editorial Commentary by Perales and Papanicolaou on pages 1534–5.)**

Cytomegalovirus (CMV) infection and disease remain clinically important complications after allogeneic hematopoietic cell transplantation (HCT) [1]. Advances in CMV diagnosis and management, such as the introduction of sensitive polymerase chain reaction–based CMV viral load assays and the commonly used strategy of preemptive antiviral therapy, have reduced the risk of development of CMV infection and disease, particularly in the first months after HCT [2–5]. Recent studies have also shown that, in HCT recipients, CMV seropositivity remains associated with decreased overall survival, which is most likely mediated through both direct and indirect effects of the virus [4, 6]. CMV-seronegative HCT recipients from CMV-seropositive donors also have poorer clinical outcomes [6, 7].

In controlled trials, CMV prophylaxis has been demonstrated to reduce the incidence of CMV infection [8, 9], but effects on measurable clinical outcomes (eg, overall survival) have been limited [8, 10, 11]. Additionally, anti-CMV agents, such as ganciclovir, foscarnet, and cidofovir are also associated with significant side effects [2, 8, 12].

Letermovir is an antiviral agent with a unique mechanism of action through inhibition of the CMV terminase complex [13, 14]. Marty et al [15] recently showed in a randomized, double-blind, placebo-controlled, prospective clinical trial that letermovir reduced the risk of clinically significant CMV infection (CS-CMVi), defined as the occurrence of CMV disease or initiation of anti-CMV preemptive therapy (PET) based on detection of CMV DNA in plasma. Furthermore, patients receiving letermovir had a lower risk for all-cause mortality at 24 weeks after HCT compared with placebo. Herein, we further analyze the effects of letermovir prophylaxis on all-cause mortality in HCT recipients.

## METHODS

### Trial Population and Design

From June 2014 to March 2016, 570 CMV-seropositive allogeneic HCT recipients were randomized in a multicenter, double-blind, placebo-controlled phase 3 trial to evaluate the safety and efficacy of letermovir for CMV prophylaxis (further details on trial population and design have been previously described [15]). Briefly, patients in this trial were randomized in a 2:1 ratio to receive either letermovir 480 mg/day (or 240 mg/day for patients receiving cyclosporine) or placebo through week 14 (~100 days) post-HCT. Randomization was stratified according to trial site and risk of CMV reactivation at baseline. As described by Marty et al, high risk of CMV reactivation was defined as meeting 1 or more of the following criteria at the time of randomization: having a related donor with at least 1 mismatch at 1 of the specified 3 human leukocyte antigen (HLA) gene loci (HLA-A, B, or DR), having an unrelated donor with at least 1 mismatch at 1 of the specified 4 HLA gene loci (HLA-A, B, C, and DRB1), having a haploidentical donor, the use of umbilical-cord blood as the stem-cell source, the use of ex vivo T-cell–depleted grafts, and having graft-versus-host disease (GVHD) of grade 2 or greater that led to the use of 1 mg/kg per day or more of prednisone (or its equivalent). All patients who did not meet the definition of being at high risk were categorized low risk of CMV reactivation (Supplementary material).

A total of 565 randomized patients received at least 1 dose of study drug. Of these, 495 did not have detectable CMV DNA at randomization and constituted the primary efficacy population (full analysis set [FAS]). Patient characteristics were generally well balanced across the treatment groups and have been described elsewhere [15]. The primary efficacy endpoint of the trial was the proportion of patients with CS-CMVi through week 24 post-HCT. Therefore, data on CS-CMVi are available only through week 24 post-HCT and were not collected subsequently in the study. Trial patients were subsequently followed for an additional 24 weeks until trial completion at week 48 post-HCT in order to ascertain CMV disease, mortality, and health outcomes data. All investigator-reported cases of suspected CMV disease were confirmed by a Clinical Adjudication Committee.

### Mortality

All-cause mortality (defined as death due to any reason) through week 48 post-HCT was a prespecified exploratory endpoint. The date of death and the reason for death were collected for all patients until they discontinued from the trial either prior to or at week 48 post-HCT in the clinical trial database. Additional information on patients’ vital status was subsequently collected after trial completion for those patients who had discontinued from the trial prior to week 48 post-HCT and whose vital status was unknown after discontinuation (“poststudy information”). At the time of trial completion, vital-status information was unavailable for 58 patients but was eventually obtained for 44 of those 58 patients. Information on vital status through week 48 post-HCT was ultimately available for 97.2% (481 of 495) of the patients in the FAS.

### Statistical Methods

Kaplan-Meier survival curves are presented for the time to all-cause mortality by treatment group. Observations were censored at trial discontinuation for reasons other than death or at trial completion. A nominal, 2-sided *P* value for the between-group differences in time to all-cause mortality is provided using the log-rank test stratified by risk of CMV reactivation at baseline. Kaplan-Meier event rates with 95% confidence intervals (CIs) were calculated for each treatment group.

Possible risk factors associated with all-cause mortality were evaluated in univariate Cox proportional hazard models. The hazard ratio (HR) and 95% CIs were calculated for all candidate risk factors. Acute GVHD grades II–IV was modeled as a time-dependent covariate. Candidate covariates were included in a multivariable Cox regression model: a factor with *P* < .25 was entered into a multivariable regression model with stepwise selection and the factor had to be significant at the 0.15 level for it to remain in the model. Using the multivariable Cox regression model, adjusted HRs for all-cause mortality were calculated. Since CS-CMVi through week 24 post-HCT was highly correlated with treatment, baseline risk of CMV reactivation, and acute GVHD through week 24 post-HCT, it was not included in the initial multivariable model selection. The selected model was chosen by using data through week 24 post-HCT. This same model was applied to the data through week 48 post-HCT. All the analyses were performed with the use of SAS software, version 9.4 (SAS Institute).

The FAS population with vital-status information available at the completion of the trial was used for most analyses presented in this publication. Additionally, a post hoc sensitivity analysis of the time to all-cause mortality, which included all poststudy vital-status information obtained from patients who discontinued the study prior to week 48 post-HCT, was performed.

## RESULTS

### All-cause Mortality

Among 495 patients with no detectable CMV DNA at randomization, there was a significant difference in all-cause mortality through week 24 post-HCT between the 2 treatment groups [15]. Sensitivity analysis using all available vital status data through week 24 post-HCT showed similar results for incidence of all-cause mortality: 40 of 325 (12.3%; 95% CI, 8.9–16.4) in the letermovir group and 32 of 170 (18.8%; 95% CI, 13.2–25.5) in the placebo group. The Kaplan-Meier event rate for all-cause mortality at week 24 post-HCT was lower in the letermovir group (12.1%; 95% CI, 8.6–15.7%) compared with the placebo group (17.2%; 95% CI, 11.5–22.9%; log-rank test, 2-sided *P = *.04) as shown in Supplementary Figure 1.

Furthermore, univariate and multivariable Cox models for time to all-cause mortality through week 24 were computed for possible factors associated with mortality (Table 1). In addition to treatment group and baseline risk of CMV reactivation, 2 factors were selected through a Cox proportional hazards model with stepwise selection: age and acute GVHD grades II–IV as a time-dependent variable. After adjusting for age, baseline risk of CMV reactivation, and acute GVHD grades II–IV, the HR for all-cause mortality was 0.58 (95% CI, 0.35–0.98; *P* = .04) in the selected model.

**Table 1.** Proportional Hazard Models of Possible Risk Factors for All-cause Mortality Through Week 24 Post–hematopoietic Cell Transplantation (Full Analysis Set)

		Deaths by Week 24	Univariate Model		Selected Model	
Covariate	N	n (%)	HR (95% CI)	*P* Value	HR (95% CI)	*P* Value
Total	495	59 (11.9)	…		…	
Letermovir	325	32 (9.8)	0.60 (0.36–1.00)	.05	0.58 (0.35–0.98)	.04
Placebo (reference)	170	27 (15.9)	…		…	
Baseline risk of CMV reactivation (randomization strata)						
High risk	147	24 (16.3)	1.67 (1.00–2.81)	.05	1.83 (1.08–3.12)	.026
Low risk (reference)	348	35 (10.1)	…		…	
Gender						
Male	280	37 (13.2)	1.31 (0.77–2.22)	.32		
Female (reference)	215	22 (10.3)	…		…	
Age						
Per 10-year increase			1.14 (0.93–1.39)	.21	1.22 (0.99–1.50)	.06
Race						
Nonwhite	79	8 (10.1)	0.85 (0.40–1.79)	.67	…	
White (reference)	416	51 (12.3)	…		…	
Ethnicity						
Hispanic or Latino	34	3 (8.8)	0.71 (0.22–2.25)	.55	…	
Not Hispanic or unknown (reference)	461	56 (12.1)	…		…	
Weight						
Per 10-kg increase			0.99 (0.86–1.15)	.93	…	
Donor CMV serostatus						
Negative or unknown	197	29 (14.7)	1.52 (0.92–2.54)	.11	…	
Positive (reference)	298	30 (10.1)	…		…	
Patients engrafted at baseline						
No or NA	329	42 (12.8)	1.29 (0.74–2.27)	.37	…	
Yes (reference)	166	17 (10.2)	…		…	
Primary reason for HCT						
Myelodysplastic syndrome	79	8 (10.1)	0.72 (0.33–1.60)	.43	…	
Lymphoma	61	8 (13.1)	0.96 (0.43–2.12)	.92	…	
Acute lymphocytic leukemia	40	5 (12.5)	0.86 (0.33–2.25)	.76	…	
Other diseases	128	12 (9.4)	0.66 (0.33–1.30)	.23	…	
Acute myeloid leukemia (reference)	187	26 (13.9)	…			
HLA matching and donor type						
Matched unrelated	192	21 (10.9)	1.03 (0.54–1.95)	.93	…	
Mismatched related	70	8 (11.4)	1.12 (0.48–2.60)	.79	…	
Mismatched unrelated	67	13 (19.4)	1.96 (0.95–4.04)	.07	…	
Matched related (reference)	166	17 (10.2)	…		…	
Haploidentical related donor						
Yes	66	8 (12.1)	1.03 (0.49–2.17)	.94	…	
No (reference)	429	51 (11.9)	…		…	
Stem-cell source						
Bone marrow	115	11 (9.6)	0.81 (0.42–1.56)	.52	…	
Cord blood	22	5 (22.7)	2.02 (0.80–5.11)	.14	…	
Peripheral blood (reference)	358	43 (12)	…		…	
Conditioning regimen						
Myeloablative	239	25 (10.5)	0.79 (0.47–1.32)	.36	…	
Reduced intensity or nonmyeloablative (reference)	256	34 (13.3)	…		…	
Antithymocyte globulin use						
Yes	167	15 (9.0)	0.62 (0.35–1.12)	.11	…	
No (reference)	328	44 (13.4)	…		…	
Immunosuppressant use						
Tacrolimus	214	17 (12.6)	1.16 (0.68–1.98)	.58	…	
Other	29	5 (17.2)	1.59 (0.61–4.14)	.34	…	
Cyclosporine (reference)	252	27 (10.7)	…		…	
CS-CMVi through week 24 (time dependent)^a^						
Yes	128	17 (13.3)	2.07 (1.14–3.73)	.02	…	
No (reference)	367	42 (11.4)	…		…	
Acute GVHD grades II–IV (time dependent)						
Yes	133	24 (18.6)	3.00 (1.75–5.15)	<.001	3.10 (1.79–5.35)	<.001
No (reference)	362	35 (9.6)	…		…	

Abbreviations: CI, confidence interval; CMV, cytomegalovirus; CS-CMVi, clinically significant cytomegalovirus infection; GVHD, graft-versus-host disease; HCT, hematopoietic cell transplantation; HLA, human leukocyte antigen; HR, hazard ratio; NA, not applicable.

^a^CS-CMVi through week 24 post-HCT was highly correlated with treatment, baseline risk of CMV, and acute GVHD through week 24 post-HCT, which is why it was not included in the stepwise model selection.

The tendency for lower all-cause mortality in the letermovir group continued through week 48 post-HCT [15]. Sensitivity analysis using all available vital-status data showed similar results: among 481 participants with vital-status data available through week 48 post-HCT, incidence of all-cause mortality was 76 of 325 (23.4%) in the letermovir group and 46 of 170 (27.1%) in the placebo group. The Kaplan-Meier event rate for all-cause mortality at week 48 post-HCT was numerically lower in the letermovir group (23.8%; 95% CI, 19.1–28.5%) compared with the placebo group (27.6%; 95% CI, 20.8–34.4%) (log-rank test, 2-sided *P* = .21), as shown in Supplementary Figure 2.

Additional univariate and multivariable Cox models for time to all-cause mortality through week 48 post-HCT were computed for possible factors associated with mortality (Table 2). After adjusting for age, baseline risk of CMV reactivation, and acute GVHD grades II–IV, the HR for all-cause mortality was 0.74 (95% CI, 0.49–1.11; *P* = .14) in the letermovir group versus the placebo group.

**Table 2.** Proportional Hazard Models of Possible Risk Factors for All-cause Mortality Through Week 48 Post–hematopoietic Cell Transplantation (Full Analysis Set)

		Deaths by Week 48	Univariate Model		Selected Model	
Covariate	N	n (%)	HR (95% CI)	*P* Value	HR (95% CI)	*P* Value
Total	495	101 (20.4)	…		…	
Letermovir	325	61 (18.8)	0.76 (0.51–1.13)	.17	0.74 (0.49–1.11)	.14
Placebo (reference)	170	40 (23.5)	…		…	
Baseline risk for CMV reactivation (randomization strata)						
High risk	147	39 (26.5)	1.57 (1.05–2.35)	.03	1.74 (1.16–2.62)	.01
Low risk (reference)	348	62 (17.8)	…		…	
Gender						
Male	280	63 (22.5)	1.33 (0.89–1.98)	.17	…	
Female (reference)	215	38 (17.7)	…		…	
Age						
Per 10-year increase			1.21 (1.03–1.42)	.02	1.29 (1.09–1.51)	.002
Race						
Nonwhite	79	17 (21.5)	1.09 (0.64–1.83)	.76	…	
White (reference)	416	84 (20.2)	…		…	
Ethnicity						
Hispanic or Latino	34	5 (14.7)	0.63 (0.26–1.56)	.32	…	
Not Hispanic or unknown (reference)	461	96 (20.8)	…		…	
Weight						
Per 10-kg increase			1.00 (0.90–1.12)	.95	…	
Donor CMV serostatus						
Negative or unknown	197	45 (22.8)	1.32 (0.89–1.96)	.16	…	
Positive (reference)	298	56 (18.8)	…		…	
Patients engrafted at baseline						
No or NA	329	73 (22.2)	1.38 (0.89–2.14)	.14	…	
Yes (reference)	166	28 (16.9)	…		…	
Primary reason for HCT						
Myelodysplastic syndrome	79	11 (13.9)	0.62 (0.32–1.21)	.16	…	
Lymphoma	61	14 (23.0)	1.07 (0.59–1.97)	.82	…	
Acute lymphocytic leukemia	40	13 (32.5)	1.43 (0.77–2.67)	.26	…	
Other diseases	128	22 (17.2)	0.73 (0.44–1.23)	.24	…	
Acute myeloid leukemia (reference)	187	41 (21.9)	…		…	
HLA matching and donor type						
Matched unrelated	192	33 (17.2)	0.86 (0.53–1.39)	.53	…	
Mismatched related	70	19 (27.1)	1.43 (0.81–2.53)	.22	…	
Mismatched unrelated	67	17 (25.4)	1.42 (0.79–2.55)	.25	…	
Matched related (reference)	166	32 (19.3)	…		…	
Haploidentical related donor						
Yes	66	19 (28.8)	1.53 (0.93–2.51)	.10	…	
No (reference)	429	82 (19.1)	…		…	
Stem-cell source						
Bone marrow	115	25 (21.7)	1.11 (0.70–1.76)	.65	…	
Cord blood	22	6 (27.3)	1.54 (0.67–3.55)	.31	…	
Peripheral blood (reference)	358	70 (19.6)	…		…	
Conditioning regimen						
Myeloablative	239	43 (18)	0.79 (0.53–1.17)	.24	…	
Reduced intensity or nonmyeloablative (reference)	256	58 (22.7)	…		…	
Antithymocyte globulin use						
Yes	167	29 (17.4)	0.71 (0.46–1.09)	.12	…	
No (reference)	328	72 (22.0)	…		…	
Immunosuppressant use						
Tacrolimus	214	52 (24.3)	1.52 (1.01–2.30)	.05	…	
Other	29	9 (31.0)	1.18 (0.92–3.90)	.08	…	
Cyclosporine (reference)	252	40 (15.9)	…		…	
CS-CMVi through week 24 (time dependent)^a^						
Yes	128	31 (24.2)	1.67 (1.08–2.59)	.02	…	
No (reference)	367	70 (19.1)	…		…	
Acute GVHD grades II–IV (time dependent)						
Yes	133	39 (29.3)	2.34 (1.55–3.52)	<.001	2.52 (1.67–3.82)	<.001
No (reference)	362	62 (17.1)	…		…	

Abbreviations: CI, confidence interval; CMV, cytomegalovirus; CS-CMVi, clinically significant cytomegalovirus infection; GVHD, graft-versus-host disease; HCT, hematopoietic cell transplantation; HLA, human leukocyte antigen; HR, hazard ratio; NA, not applicable.

^a^CS-CMVi through week 24 post-HCT was highly correlated with treatment, baseline risk for CMV, and acute GVHD through week 24 post-HCT, which is why it was not included in the multivariate model selection.

### All-cause Mortality Through Week 48 Post-HCT in Patients With and Without Clinically Significant CMV Infection

To further evaluate the reduction in all-cause mortality in the letermovir group compared with the placebo group, the incidence of all-cause mortality through week 48 post-HCT was also examined among the subsets of patients with or without CS-CMVi through week 24 post-HCT, which was the primary endpoint in the trial (Table 3). The incidence of all-cause mortality in the placebo group was substantially higher in patients with CS-CMVi, despite the use of PET, than in patients without CS-CMVi (31.0% vs 18.2%, respectively). The HR of mortality for CS-CMVi compared with no CS-CMVi (CS-CMVi was treated as a time-dependent variable) was 2.34 (95% CI, 1.17–4.67) in the placebo group after adjusting for baseline age, with a nominal *P* = .02 (GVHD and baseline risk of CMV reactivation were not adjusted for in the model due to multicollinearity). This indicates that mortality is associated with CMV reactivation even in subjects who received the current standard of care (active viral load monitoring with initiation of PET in subjects with documented viremia).

**Table 3.** All-cause Mortality Through Week 48 Post–hematopoietic Cell Transplant Among Patients Who Developed Clinically Significant Cytomegalovirus Infection (Full Analysis Set)

	Letermovir (n = 325)			Placebo (n = 170)		
	n/N (%)	HR^a^ (95% CI)	*P* Value	n/N (%)	HR^a^ (95% CI)	*P* Value
CS-CMVi (time dependent)	9/57 (15.8)	1.15 (0.56–2.37)	.71	22/71 (31.0)	2.34 (1.17–4.67)	.02
No CS-CMVi (time dependent)	52/268 (19.4)	…		18/99 (18.2)	…	

Graft-versus-host disease and baseline risk of CMV reactivation were not adjusted for in the model due to multicollinearity (both variables were highly correlated with CS-CMVi). CS-CMVi is treated as a time-dependent variable in the model because the time of onset of CS-CMVi varies for each subject. Death includes all-cause mortality through week 48 post-HCT. Clinically significant CMV infection is defined through week 24 post-HCT. Denominator in the first row only includes subjects with clinically significant CMV infection and does not include subjects who discontinued early and had missing data. Every subject is counted a single time for each applicable row and column.

Abbreviations: CI, confidence interval; CMV, cytomegalovirus; CS-CMVi, clinically significant cytomegalovirus infection; HCT, hematopoietic cell transplantation; HR, hazard ratio.

^a^HR is adjusted for baseline age.

In contrast, in the letermovir group, the incidence of all-cause mortality was similar in patients with or without CS-CMVi through week 24 post-HCT (15.8% vs 19.4%, respectively) (Table 3). The HR (95% CI) of mortality for CS-CMVi versus no CS-CMVi in the letermovir group (CS-CMVi was treated as a time-dependent variable) was 1.15 (95% CI, 0.56–2.37), with a nominal *P* = .71 after adjusting for baseline age. The HR was reduced from 2.34 without letermovir intervention (placebo) to 1.15 with letermovir intervention, indicating that letermovir is an effect modifier for CS-CMVi effect on mortality.

**Figure 1.**
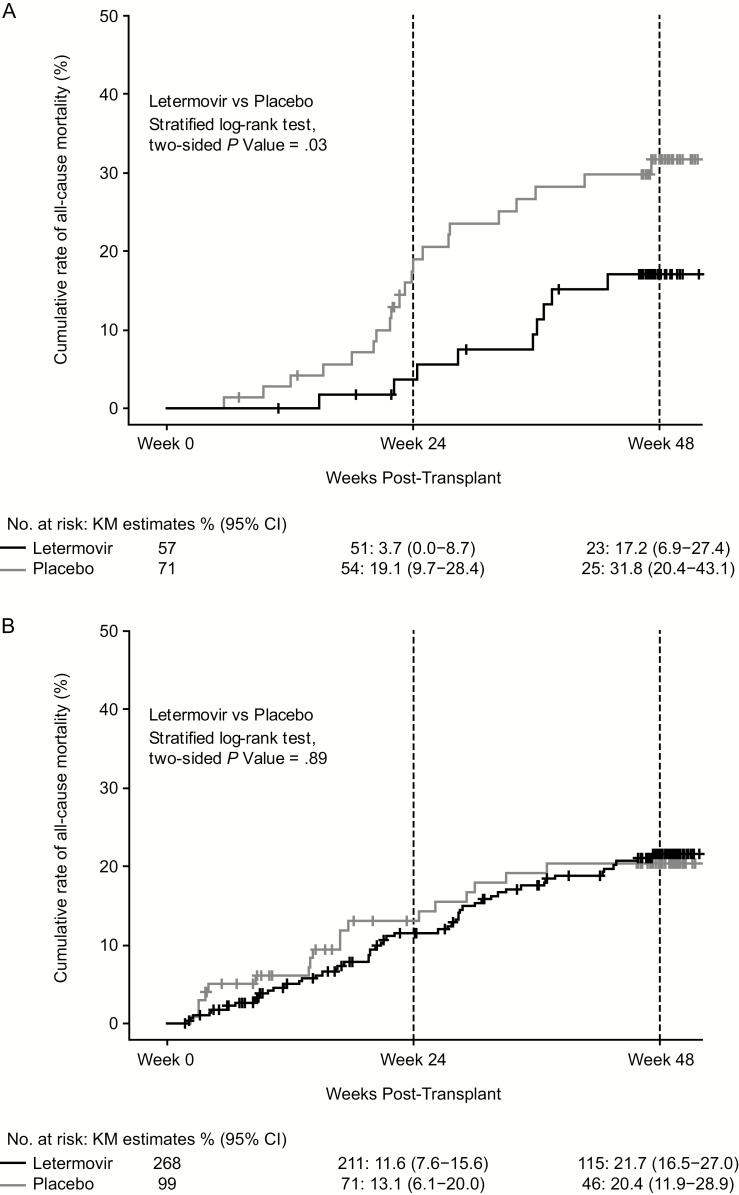
All-cause mortality through week 48 post-HCT in participants with (*A*) and without (*B*) CS-CMVi through week 24 post-HCT. Abbreviations: CI, confidence interval; CS-CMVi, clinically significant cytomegalovirus infection; HCT, hematopoietic stem-cell transplantation; KM, Kaplan-Meier.

Given the differential effect of CS-CMVi on mortality by treatment group, a Cox regression model was fitted to estimate the treatment effect in patients with and without CS-CMVi by adding CS-CMVi (as a time-dependent variable) and interaction between CS-CMVi and treatment into the selected model in Table 1. After adjusting for other risk factors, the HR for all-cause mortality was 0.45 (95% CI, 0.21–1.00; *P* = .05) for letermovir versus placebo among patients who developed CS-CMVi through week 24 and the HR was 1.05 (95% CI, 0.61–1.81; *P* = .85) for letermovir versus placebo among patients who did not develop CS-CMVi (Table 4). These results are consistent with those seen in Kaplan-Meier plots of all-cause mortality through week 48 post-HCT in subjects with and without CS-CMVi through week 24 post-HCT, as shown in Figure 1.

**Table 4.** Proportional Hazard Model for All-cause Mortality Through Week 48 Post–hematopoietic Cell Transplant

Factors	Multivariable HR (95% CI)	*P* Value
Letermovir vs placebo with CS-CMVi	0.45 (0.21–1.00)	.05
Letermovir vs placebo without CS-CMVi	1.05 (0.61–1.81)	.85
CS-CMVi through week 24 (time dependent)	2.05 (1.09–3.88)	.03
Acute GVHD grades II–IV (time dependent)	2.58 (1.69–3.92)	<.001
Age (by 10-year increase)	1.29 (1.10–1.52)	.002
Baseline CMV risk of CMV reactivation (high vs low)	1.70 (1.13–2.57)	.01

Abbreviations: CI, confidence interval; CMV, cytomegalovirus; CS-CMVi, clinically significant cytomegalovirus infection; GVHD, graft-versus-host disease; HCT, hematopoietic stem-cell transplantation; HR, hazard ratio.

### Causes of Death

The reported primary causes of death at weeks 24 and 48 post-HCT are shown in Table 5. The most frequently reported causes of death at both time points included acute myeloid leukemia, GVHD, sepsis, pneumonia, and respiratory failure. None of these deaths were considered to be related to the study medication by investigators.

**Table 5.** Most Common Causes of All-cause Mortality by Preferred Terms (Full Analysis Set)

	Through Week 24 Post-HCT, n (%)		Through Week 48 Post-HCT, n (%)	
	Letermovir	Placebo	Letermovir	Placebo
Total patients in population	325	170	325	170
Patients who died	32 (9.8)	27 (15.9)	61 (18.8)	40 (23.5)
AML^a^	7 (2.2)	7 (4.1)	12 (3.7)	10 (5.9)
GVHD^b^	2 (0.6)	3 (1.8)	7 (2.2)	7 (4.1)
Sepsis^c^	4 (1.2)	3 (1.8)	6 (1.8)	4 (2.4)
Pneumonia	1 (0.3)	1 (0.6)	2 (0.6)	3 (1.8)
Respiratory failure^d^	2 (0.6)	0 (0.0)	7 (2.2)	1 (0.6)
ALL^e^	2 (0.6)	1 (0.6)	3 (0.9)	1 (0.6)
Multiple organ dysfunction syndrome	2 (0.6)	2 (1.2)	2 (0.6)	2 (1.2)
Septic shock	1 (0.3)	2 (1.2)	2 (0.6)	2 (1.2)

Note that this table only lists the most common causes of all-cause mortality. Every patient is counted a single time for each applicable row and column.

Abbreviations: ALL, acute lymphatic leukemia; AML, acute myeloid leukemia; GVHD, graft-versus-host disease; HCT, hematopoietic cell transplantation.

^a^Includes AML and recurrent AML.

^b^Includes GVHD, acute GVHD, and gastrointestinal GVHD.

^c^Includes Enterococcal sepsis, Klebsiella sepsis, and Neutropenic sepsis.

^d^Includes acute respiratory failure.

^e^Includes ALL and recurrent ALL.

Of the 14 (2.8%) subjects (8 [2.5%] in the letermovir group and 6 [3.5%] in the placebo group) who developed CMV disease through week 48 post-HCT, a fatal outcome was reported for 5 (1.0%) subjects (2 [0.6%] in the letermovir group; 3 [1.8%] in the placebo group). The primary reported causes of death in the 2 subjects in the letermovir group were bacterial pneumonia and cerebrovascular accident. In the placebo group, the 3 subjects died of pneumonia (due to CMV, aspergillosis, and *Escherichia coli* infection), septic shock, and intracranial hemorrhage, respectively. None of these deaths was reported as being related to the study medication.

## DISCUSSION

Cytomegalovirus seropositivity and reactivation have been shown to be associated with reduced survival after allogeneic HCT [4, 16]. Prophylaxis of CMV-seropositive HCT recipients with letermovir modified this risk by preventing CS-CMVi, which was also associated with reduced all-cause mortality through week 24 post-HCT [15].

A mortality benefit associated with letermovir that is mediated directly through the inhibition of CMV replication is consistent with recently reported findings from retrospective cohort studies in HCT recipients, which suggested that any level of CMV viremia is associated with an increased risk of overall mortality after HCT [16], independently of the use of PET [17]. Since significantly fewer patients in the letermovir group developed CS-CMVi compared with the placebo group and the incidence of death was lower in the letermovir group compared with placebo [15], these results suggest that the reduction in all-cause mortality observed with letermovir correlates with the prevention of CMV viremia.

In patients with CS-CMVi, the mortality rate in the letermovir group was lower than in the placebo group (15.8% vs 31%). Of those in the letermovir treatment group, 43.9% developed CS-CMVi through week 14 post-HCT (“early CMV reactivation”) compared with 94% in the placebo group; the remaining patients in the letermovir group developed CS-CMVi between weeks 14 and 24 post-HCT (“late CMV reactivation”) [15]. Since immune reconstitution following allogeneic HCT improves over time, delaying CMV reactivation is clinically important, as this delay ensures that the reconstituted immune system can modulate the clinical consequences associated with CMV reactivation [17, 18]. This would also be applicable to patients previously treated with letermovir prophylaxis. Thus, in the letermovir group, we speculate that the observed delay in time to viremia likely contributed to the observed mortality benefit.

Another possible contributing factor to the decrease in all-cause mortality is that the decreased and delayed need for PET (usually with ganciclovir/valganciclovir), with its associated toxicities, in the letermovir group as compared with placebo reduced the negative effects of these effective but bone-marrow–toxic antiviral drugs after HCT [19].

The period of highest risk of CMV reactivation and/or disease is during the first 3 months post-HCT. Letermovir was administered to study participants during this 3-month period and likely decreased mortality by reducing CS-CMVi during this time, as well as by delaying CS-CMVi to a later stage of lower risk during the next 3 months as mentioned above. The separation of the 2 Kaplan-Meier curves (letermovir vs placebo) for all-cause mortality is consistent with this assertion and resulted in a statistically significant difference between the treatment arms through week 24 post-HCT. As the study was not powered to detect a difference between treatment groups in all-cause mortality at week 48, a larger separation of the curves would have been necessary to demonstrate a statistically significant difference at this time point.

As observed in Table 5, the most common causes of mortality were similar at weeks 24 and 48 post-HCT. The difference in all-cause mortality (an absolute difference of ~4–5%) between the 2 treatment arms persisted through week 48 post-HCT; however, the results were not statistically significant at this time point. Due to the lower number of patients who were at risk of CS-CMVi at week 48 post-HCT, a further separation of the curves would have been required to retain a significant effect on all-cause mortality; however, such separation did not occur after approximately week 28 post-HCT. This is likely due to the risk of developing CS-CMVi decreasing over time as patients became more immune competent and were able to control CMV reactivation. Alternatively, CS-CMVi occurring after discontinuing prophylaxis could have had a negative impact on overall mortality, reducing the separation between the curves, but this possibility is not supported by the data. Thus, while the trend in lower all-cause mortality in the letermovir arm continued through week 48 post-HCT, there was no further separation of the curves, which accounts for the results not being statistically different after week 24 post-HCT.

The effectiveness of letermovir provides an opportunity to prospectively study the impact of CMV replication on post-HCT outcomes in a systematic manner in different categories of HCT recipients, rather than relying on retrospective registry and noninterventional data. As previously reported, the impact of a prophylactic intervention will be higher in patients at high risk of CMV reactivation and/or disease [15]. Thus, it is also possible that longer letermovir prophylaxis is needed in some patient populations to allow better immune reconstitution. This is supported by the trend to lower all-cause mortality at week 48. However, this needs to be explored further in future trials.

## Supplementary Data

Supplementary materials are available at *Clinical Infectious Diseases* online. Consisting of data provided by the authors to benefit the reader, the posted materials are not copyedited and are the sole responsibility of the authors, so questions or comments should be addressed to the corresponding author.

ciz490_suppl_Supplementary_Figure_1

ciz490_suppl_Supplementary_Figure_2

ciz490_suppl_Supplementary_Data

ciz490_suppl_Supplementary_Materials

## Notes

***Acknowledgments.*** Editorial support, under the direction of the authors, was provided by Paul O’Neill of CMC AFFINITY, a division of McCann Health Medical Communications Ltd, Glasgow, United Kingdom, in accordance with Good Publication Practice (GPP3) guidelines. This assistance was funded by Merck Sharp & Dohme Corporation, a subsidiary of Merck & Co., Inc., Kenilworth, New Jersey.

***Financial support.*** This work was supported by Merck Sharp & Dohme Corporation, a subsidiary of Merck & Co., Inc., Kenilworth, New Jersey.

***Potential conflicts of interest.*** P. L. has received grants from Astellas, Inc, Merck & Co., Inc., Oxford Immunotech, and Shire and other support from AiCuris and Shire Pharmaceuticals. M. S. declares grants from Novartis, personal fees from Shire and Sandoz, and other fees from Merck Sharp & Dohme and Kite/Gilead. F. M. M. has received grants and personal fees from Astellas Pharma, Inc, Chimerix, Inc., Merck & Co., Inc., and Shire Pharmaceuticals and personal fees from Fate Therapeutics, LFB Group, S.A. Pharmachem, Roche Molecular Diagnostics, and REgeneron. J. M. has received grants from Immuno-Myologics Inc. and OLM Diagnostics; grants, personal fees, and nonfinancial support from Gilead Sciences, Merck & Co., Inc., and Pfizer, Inc.; and personal fees and nonfinancial support from F2G, Cidara, and Basilea Pharmaceuticals. R. F. C. has received grants and personal fees from Chimerix, Inc., Shire, and Merck & Co., Inc.; grants from Novartis; and personal fees from Astellas, Inc., and Oxford Immunotec. N. A. K., J. R. B., H. W., V. L. T., K. S., Y. M., R. Y. L., and C. B. are employees of Merck Sharp & Dohme Corporation, a subsidiary of Merck & Co., Inc., Kenilworth, New Jersey, and may own stock and/or stock options in the company. All authors have submitted the ICMJE Form for Disclosure of Potential Conflicts of Interest. Conflicts that the editors consider relevant to the content of the manuscript have been disclosed.
