# Supplementary figures and images for: A Mortality Analysis of Letermovir Prophylaxis for Cytomegalovirus (CMV) in CMV-seropositive Recipients of Allogeneic Hematopoietic Cell Transplantation
Source: Clin Infect Dis. 2019 Jun 8;70(8):1525–33. doi: 10.1093/cid/ciz490 (PMC7146004; doi:10.1093/cid/ciz490)

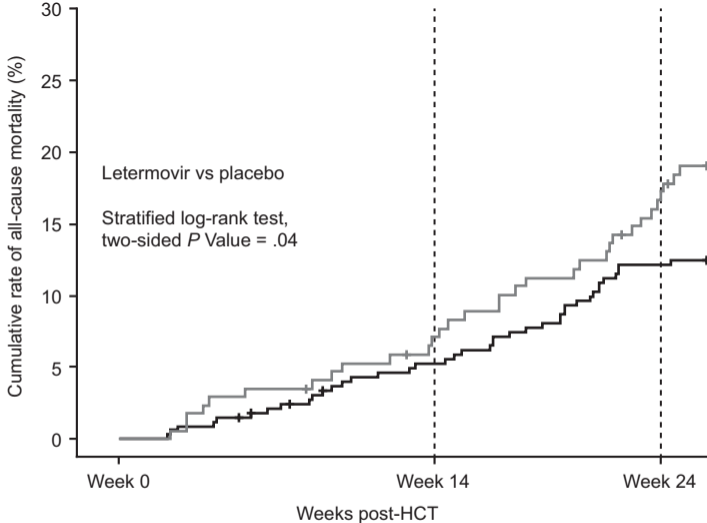

No. at risk: KM estimates % (95% CI)

|              |     |                     |                       |
|--------------|-----|---------------------|-----------------------|
| — Letermovir | 325 | 304: 5.3 (2.8–7.7)  | 282: 12.1 (8.6–15.7)  |
| — Placebo    | 170 | 156: 7.1 (3.2–11.0) | 139: 17.2 (11.5–22.9) |

Supplement: ciz490_suppl_Supplementary_Figure_1 [file ciz490_suppl_supplementary_figure_1.pdf]

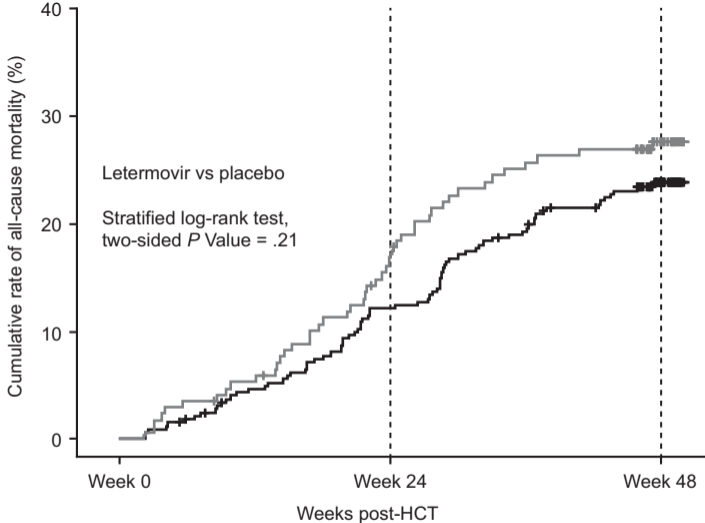

No. at risk: KM estimates % (95% CI)

|           |     |                       |                       |
|-----------|-----|-----------------------|-----------------------|
| Letemovir | 325 | 282: 12.1 (8.6–15.7)  | 165: 23.8 (19.1–28.5) |
| Placebo   | 170 | 139: 17.2 (11.5–22.9) | 81: 27.6 (20.8–34.4)  |

Supplement: ciz490_suppl_Supplementary_Figure_2 [file ciz490_suppl_supplementary_figure_2.pdf]
