# Supplementary material for: A Mortality Analysis of Letermovir Prophylaxis for Cytomegalovirus (CMV) in CMV-seropositive Recipients of Allogeneic Hematopoietic Cell Transplantation
Source: Clin Infect Dis. 2019 Jun 8;70(8):1525–33. doi: 10.1093/cid/ciz490 (PMC7146004; doi:10.1093/cid/ciz490)
Supplement: ciz490_suppl_Supplementary_Data [file ciz490_suppl_supplementary_data.pdf]

THIS PROTOCOL AND ALL OF THE INFORMATION RELATING TO IT ARE CONFIDENTIAL AND PROPRIETARY PROPERTY OF MERCK SHARP & DOHME CORP., A SUBSIDIARY OF MERCK & CO., INC., WHITEHOUSE STATION, NJ, U.S.A.

SPONSOR:

Merck Sharp & Dohme Corp., a subsidiary of Merck & Co., Inc.
(hereafter referred to as the Sponsor or Merck)
One Merck Drive
P.O. Box 100
Whitehouse Station, NJ 08889-0100, U.S.A.

Protocol-specific Sponsor Contact information can be found in the Investigator Trial File Binder.

TITLE:

A Phase III Randomized, Placebo-controlled Clinical Trial to Evaluate the Safety and Efficacy of MK-8228 (Letermovir) for the Prevention of Clinically Significant Human Cytomegalovirus (CMV) Infection in Adult, CMV-Seropositive Allogeneic Hematopoietic Stem Cell Transplant Recipients

IND NUMBER: 104,706
EudraCT NUMBER: 2013-003831-31

TABLE OF CONTENTS

1.0 TRIAL SUMMARY ... **10**

2.0 TRIAL DESIGN ... **11**

2.1 Trial Design ... **11**

2.2 Trial Diagram ... **15**

3.0 OBJECTIVE(S) & HYPOTHESIS(ES) ... **15**

3.1 Primary Objective(s) & Hypothesis(es) ... **15**

3.2 Secondary Objective(s) & Hypothesis(es) ... **15**

3.3 Exploratory Objectives ... **16**

4.0 BACKGROUND & RATIONALE ... **17**

4.1 Background ... **17**

4.1.1 Pharmaceutical and Therapeutic Background ... 17

4.2 Rationale ... **18**

4.2.1 Rationale for the Trial and Selected Subject Population ... 18

4.2.2 Rationale for Dose Selection/Regimen ... 19

4.2.3 Rationale for Endpoints ... 21

4.2.3.1 Efficacy Endpoints ... 21

4.2.3.2 Safety Endpoints ... 23

4.2.3.3 Pharmacokinetic Endpoints ... 23

4.2.3.4 Future Biomedical Research ... 23

5.0 METHODOLOGY ... **24**

5.1 Entry Criteria ... **24**

5.1.1 Diagnosis/Condition for Entry into the Trial ... 24

5.1.2 Subject Inclusion Criteria ... 24

5.1.3 Subject Exclusion Criteria ... 25

5.2 Trial Treatments ... **27**

5.2.1 Dose Selection ... 29

5.2.1.1 Dose Selection ... 29

5.2.2 Dose Modification ... 29

5.2.3 Timing of Dose Administration ... 30

5.2.4 Trial Blinding/Masking 30
5.3 Randomization or Treatment Allocation 31
5.4 Stratification 31
5.5 Concomitant Medications/Vaccinations (allowed & prohibited) 31
5.6 Rescue Medications & Supportive Care 33
5.7 Diet/Activity/Other Considerations 33
5.8 Subject Withdrawal/Discontinuation Criteria 34
5.9 Subject Replacement Strategy 35
5.10 Beginning and End of the Trial 35
5.11 Clinical Criteria for Early Trial Termination 36
6.0 TRIAL FLOW CHART 37
7.0 TRIAL PROCEDURES 43
7.1 Trial Procedures 43
7.1.1 Administrative Procedures 43
7.1.1.1 Informed Consent 43
7.1.1.1.1 General Informed Consent 43
7.1.1.1.2 Consent and Collection of Specimens for Future Biomedical Research 43
7.1.1.2 Inclusion/Exclusion Criteria 44
7.1.1.3 Subject Identification Card 44
7.1.1.4 Medical History 44
7.1.1.5 Prior and Concomitant Medications Review 44
7.1.1.5.1 Prior Medications 44
7.1.1.5.2 Concomitant Medications 44
7.1.1.6 HSCT Details Review 44
7.1.1.7 Assignment of Screening Number 44
7.1.1.8 Assignment of Randomization Number 45
7.1.1.9 Trial Compliance (Study Therapy) 45
7.1.2 Clinical Procedures/Assessments 45
7.1.2.1 Physical Examination 45
7.1.2.2 Weight and Height Assessment 45
7.1.2.3 Vital Signs 46

7.1.2.4 12-Lead Electrocardiogram 46
7.1.2.5 Child Pugh Score 46
7.1.2.6 Birth Control Confirmation........ 46
7.1.2.7 Adverse Events Monitoring 46
7.1.2.8 CMV Disease Assessment 47
7.1.2.9 Health Outcomes Assessment........ 47
7.1.2.10 Quality of Life Assessment........ 47
7.1.3 Laboratory Procedures/Assessments 47
7.1.3.1 Laboratory Safety Evaluations (Hematology, Chemistry and Urinalysis) .. 47
7.1.3.2 Pharmacokinetic/Pharmacodynamic Evaluations 49
7.1.3.2.1 Blood Collection for Pharmacokinetic Sampling 49
7.1.3.3 CMV DNA PCR Testing 49
7.1.3.4 CMV DNA Sequence Analysis 50
7.1.3.5 Future Biomedical Research 50
7.1.4 Other Procedures........ 50
7.1.4.1 Withdrawal/Discontinuation 50
7.1.4.1.1 Withdrawal From Future Biomedical Research 51
7.1.4.2 Blinding/Unblinding 51
7.1.5 Visit Requirements........ 51
7.1.5.1 Screening........ 51
7.1.5.2 Study Therapy Period 52
7.1.5.2.1 Day 1 Visit 53
7.1.5.2.2 Study Therapy Administration........ 53
7.1.5.3 Follow-up Period 54
7.1.5.4 CMV Infection or Early Discontinuation Visit........ 54
7.2 Assessing and Recording Adverse Events 55
7.2.1 Definition of an Overdose for This Protocol and Reporting of Overdose to the Sponsor........ 56
7.2.2 Reporting of Pregnancy and Lactation to the Sponsor 56
7.2.3 Immediate Reporting of Adverse Events to the Sponsor........ 57
7.2.3.1 Serious Adverse Events 57
7.2.3.2 Events of Clinical Interest........ 57
7.2.3.3 Protocol-Specific Exceptions to Serious Adverse Event Reporting........ 58

7.2.4 Evaluating Adverse Events 58
7.2.5 Sponsor Responsibility for Reporting Adverse Events 61
7.3 TRIAL GOVERNANCE AND OVERSIGHT 61
7.3.1 Scientific Advisory Committee 61
7.3.2 Executive Oversight Committee 61
7.3.3 Data Monitoring Committee 61
7.3.4 Clinical Adjudication Committee 62
8.0 STATISTICAL ANALYSIS PLAN 62
8.1 Statistical Analysis Plan Summary 62
8.1.1 Efficacy Analyses 62
8.1.2 Safety Analyses 63
8.1.3 Power and Sample Size 63
8.1.4 Interim Analysis 64
8.2 Statistical Analysis Plan 64
8.2.1 Responsibility for Analyses/ In-House Blinding 64
8.2.2 Hypotheses/Estimation 65
8.2.3 Analysis Endpoints 65
8.2.3.1 Efficacy/Pharmacokinetic Endpoints 66
8.2.3.1.1 Efficacy Endpoints 66
8.2.3.1.2 Exploratory Endpoints 67
8.2.3.2 Safety Endpoints 68
8.2.4 Analysis Populations 68
8.2.4.1 Efficacy Analysis Populations 68
8.2.4.2 Safety Analysis Populations 69
8.2.5 Statistical Methods 69
8.2.5.1 Statistical Methods for Efficacy Analyses 69
8.2.5.1.1 Primary Efficacy Analysis 69
8.2.5.1.2 Secondary Efficacy Analysis 69
8.2.5.1.3 Exploratory Analysis 72
8.2.5.1.4 Missing Data Handling 72
8.2.5.2 Statistical Methods for Safety Analyses 73

8.2.5.3 Summaries of Baseline Characteristics, Demographics, and Other Analyses 74
8.2.6 Multiplicity 75
8.2.7 Sample Size and Power Calculations 75
8.2.7.1 Sample Size and Power for Efficacy Analysis 75
8.2.7.2 Sample Size and Power for Safety Analysis 76
8.2.8 Subgroup Analyses and Effects of Baseline Factors 76
8.2.9 Interim Analyses 77
8.2.10 Compliance/Medication Adherence 77
8.2.11 Extent of Exposure 78
9.0 LABELING, PACKAGING, STORAGE AND RETURN OF CLINICAL SUPPLIES 78
9.1 Investigational Product 78
9.2 Packaging and Labeling Information 78
9.3 Clinical Supplies Disclosure 79
9.4 Storage and Handling Requirements 79
9.5 Returns and Reconciliation 79
9.6 Standard Policies 79
10.0 ADMINISTRATIVE AND REGULATORY DETAILS 79
10.1 Confidentiality 79
10.1.1 Confidentiality of Data 79
10.1.2 Confidentiality of Subject Records 80
10.1.3 Confidentiality of Investigator Information 80
10.1.4 Confidentiality of IRB/IEC Information 80
10.2 Compliance with Financial Disclosure Requirements 81
10.3 Compliance with Law, Audit and Debarment 81
10.4 Compliance with Trial Registration and Results Posting Requirements 83
10.5 Quality Management System 83
10.6 Data Management 83
10.7 Publications 83
11.0 LIST OF REFERENCES 85
12.0 APPENDICES 88

12.1 Merck Code of Conduct for Clinical Trials 88
12.2 Collection and Management of Specimens for Future Biomedical Research 90
12.3 Understanding the Intent, Scope and Public Health Benefits of Exploratory Biomarker Research: A Guide for IRBs/IECs and Investigational Site Staff 96
12.4 Definition of CMV Disease in Hematopoietic Stem Cell Transplant (HSCT) Recipients 107
12.5 Child-Pugh Classification for Severity of Liver Disease 110
12.6 Clinical Experience with IV Formulation 111
12.7 Approximate Blood/Tissue Volumes Drawn/Collected by Trial Visit and by Sample Types 120

13.0 SIGNATURES 121
13.1 Sponsor's Representative 121
13.2 Investigator 121

LIST OF TABLES

Table 1 Study Therapy – Oral (Tablet) Formulation 28
Table 2 Study Therapy – IV Formulation 28
Table 3 Blinding of Tablets Related to CsA Use 30
Table 4 Laboratory Tests 48
Table 5 Evaluating Adverse Events 59
Table 6 Summary of Analysis Strategy for Key Efficacy Endpoints 63
Table 7 Analysis Strategy for Efficacy Variables 71
Table 8 Analysis Strategy for Safety Parameters 74
Table 9 Upper Bound of the Two-Sided 95% Confidence Interval for the True Proportion of Subjects with an AE 76
Table 10 Product Descriptions 78

LIST OF FIGURES

Figure 1 Trial Diagram 15

1.0 TRIAL SUMMARY

Abbreviated Title	MK-8228 vs. Placebo in Prevention of CMV infection in HSCT Recipients
Trial Phase	Phase III
Clinical Indication	Prevention of clinically significant CMV infection in allogeneic HSCT recipients
Trial Type	Interventional
Type of control	Placebo
Route of administration	Oral, Intravenous
Trial Blinding	Double-blind
Treatment Groups	Arm 1: MK-8228 240 mg once daily (qd), if receiving concomitant cyclosporin A (CsA), or MK-8228 480 mg qd, if not on CsA, through Week 14 (~100 days) post-transplant<br><br>Arm 2: Placebo qd, through Week 14 (~100 days) post-transplant
Number of trial subjects	Approximately 540 subjects will be enrolled.
Estimated duration of trial	The sponsor estimates that the trial will require approximately 40 months from the time the first subject signs the informed consent until the last subject's last visit.
Duration of Participation	Each subject will participate in the trial for approximately 49 weeks from the time the subject signs the Informed Consent Form (ICF) through the final contact. After a screening phase which may vary from 7 days pre-transplant to 28 days post-transplant, each subject will receive assigned study therapy (MK-8228 or placebo) through Week 14 (~100 days) post-transplant. After the end of study therapy, each subject will continue to be followed through Week 24 post-transplant. Additionally, they will have follow-up visits at Weeks 32, 40, and 48 post-transplant.
Randomization Ratio	2:1 ratio of MK-8228: placebo (~360 subjects to receive MK-8228 and ~180 subjects to receive placebo)

2.0 TRIAL DESIGN

2.1 Trial Design

This is a randomized, placebo-controlled, multi-site, double-blind trial of MK-8228 (also known as letermovir, AIC246, AIC001; hereafter referred to as MK-8228) in the prevention of clinically significant human cytomegalovirus (CMV) infection in adult, CMV-seropositive allogeneic hematopoietic stem cell transplant (HSCT) recipients. Clinically significant CMV infection is defined as the occurrence of either one of the following outcomes:

- onset of CMV end-organ disease, or
- initiation of anti-CMV pre-emptive therapy (PET) based on documented CMV viremia (as measured by the central laboratory) and the clinical condition of the subject.

Approximately 540 eligible HSCT recipients will be randomized in a 2:1 ratio to receive MK-8228 or placebo (i.e., ~360 and ~180 on MK-8228 and placebo, respectively) at any time from the day of transplant until 28 days post-transplant. Both oral (tablet) and intravenous (IV) formulations of MK-8228 (and placebo) will be available for study therapy. Subjects will be initiated with the oral (tablet) formulation of study therapy provided they are able to swallow and do not have a condition (e.g., vomiting, diarrhea, or a malabsorptive condition) that may interfere with the absorption of the tablets. For subjects who cannot swallow and/or have a condition that may interfere with the absorption of the oral formulation (e.g., vomiting, diarrhea, or a malabsorptive condition), study therapy can be initiated with or switched to the IV formulation. The IV formulation should be switched to oral study therapy (i.e., at the next planned dose) as soon as such subjects are able to swallow and/or the condition necessitating the use of the IV formulation resolves. Use of the IV formulation should generally be limited to 4 weeks or less in duration. However, it will be left to the investigator's discretion to continue IV administration beyond 4 weeks, if the benefit/risk ratio supports continued administration.

The dose of MK-8228 will either be 240 mg once daily (qd), for subjects receiving concomitant cyclosporin A (CsA), or 480 mg qd, if the subject is not on CsA. As CsA has been shown to increase MK-8228 levels, the dose of MK-8228 must be adjusted for subjects taking CsA (concomitant use of other immunosuppressive agents like tacrolimus does not require this adjustment). Placebo for MK-8228 will be administered to maintain study blinding.

Subjects will be stratified by 1) study center and 2) risk (for reactivation of CMV disease) factor group. Risk factor groups include 2 categories as defined below:

1. High risk: Subjects meeting one or more of the following criteria at the time of randomization:
    - Human leukocyte antigen (HLA)-related (sibling) donor with at least one mismatch at one of the following three HLA-gene loci: HLA-A, -B or -DR,
    - Haploidentical donor,

- Unrelated donor with at least one mismatch at one of the following four HLA-gene loci: HLA-A, -B, -C and -DRB1,
- Use of umbilical cord blood as stem cell source,
- Use of *ex vivo* T-cell-depleted grafts,
- Grade 2 or greater graft-versus-host disease (GVHD), requiring the use of systemic corticosteroids (defined as the use of ≥ 1 mg/kg/day of prednisone or equivalent dose of another corticosteroid).

2. Low risk: All subjects not meeting definition of high risk.

Subjects must have documented seropositivity for CMV (recipient CMV IgG seropositivity [R+]) within one year prior to transplantation to be eligible for the study. Donor CMV serostatus may either be positive (D+) or negative (D-). Screening of potential eligible subjects may begin 7 days prior to transplantation. Subjects will have plasma samples tested for CMV viremia using the CMV DNA PCR assay (for initial screening purposes, results of the assay done at a local laboratory will be acceptable).

After establishing absence of CMV viremia, subjects will be tested once a week by the central laboratory using the CMV DNA PCR assay from the time of transplantation until randomization in order to minimize enrollment of those with active CMV replication in the study. Any subject who tests positive for CMV viremia (as documented by central or local laboratory test results) prior to randomization at any time point will be excluded from the study, even if subsequent tests are negative for CMV viremia. Once randomized, CMV viremia will be monitored at the time intervals detailed in the Trial Flow Chart (Section 6.0).

Study therapy (with MK-8228 or placebo) may begin as early as the day of transplant and no later than 28 days post-transplant. Study therapy will continue through Week 14 (~100 days) post-transplant with the primary intent of preventing clinically significant CMV infection. On the day of randomization, eligibility for enrollment into the study should be confirmed (including confirmation that HSCT has taken place). At that time, subjects should have no documented CMV viremia (as confirmed by the central laboratory) from a plasma sample collected within 7 days prior to randomization. In addition, creatinine clearance and liver function test results within 7 days prior to randomization should also be available and within the range allowable in this study as outlined in Section 5.1.3 (Subject Exclusion Criteria).

Once enrolled in the study, subjects will have study visits scheduled at weekly intervals during the treatment period which will be through Week 14 (~100 days) post-transplant. Thereafter, subjects will be followed through Week 24 (~6 months) post-transplant. At all study visits through Week 24 post-transplant, plasma samples will be collected for CMV DNA PCR testing (for testing by central laboratory) and the investigator must assess the subject to determine if the subject meets one of the criteria for clinically significant CMV infection (as defined above).

Following completion of the primary study period at Week 24 (~6 months) post-transplant, all subjects will remain in the study through Week 48 post-transplant in order to continue collecting information on (1) CMV disease; (2) health outcomes data such as incidence of all-cause mortality, re-hospitalizations (including those for CMV-related causes), GVHD, and opportunistic infections; and (3) quality of life (QoL) measures using validated patient

reported outcome tools. Study visits will occur at Weeks 32, 40, and 48 post-transplant to collect this information and to collect plasma samples for CMV DNA PCR testing (by the central laboratory) at these time points.

For subjects who develop clinically significant CMV infection during the study treatment period (up to Week 14 post-transplant): When the investigator intends to initiate either treatment for CMV disease or PET, the subject should have a CMV Infection Visit. It is very important to ensure that all procedures, as outlined in the Trial Flow Chart (Section 6.0), are performed at the CMV Infection Visit **immediately prior to** the initiation of treatment of CMV diseases or initiation of PET (i.e., on the day anti-CMV therapy is initiated). Most importantly, a plasma sample for CMV PCR testing at the central laboratory should be collected at this visit. Thereafter, the subject should be discontinued from study therapy and treated according to the local standard of care (outside the context of the study). Such subjects will however, continue to be followed in the study (despite discontinuing study therapy and initiating anti-CMV therapy) and complete all remaining study visits. At such subsequent visits, all procedures specified in the Trial Flow Chart should be completed with the exception of study therapy administration, pharmacokinetic (PK) assessments, and study medication diary review.

For subjects with clinically significant CMV infection during the post-treatment [follow-up] period (after Week 14 and through Week 24 post-transplant): When an investigator intends to initiate either treatment for CMV disease or PET, the subject should have a CMV Infection Visit. It is very important to ensure that all procedures, as outlined in the Trial Flow Chart (Section 6.0), are performed at the CMV Infection Visit **immediately prior to** the initiation of treatment of CMV diseases or initiation of PET (i.e., on the day anti-CMV therapy is initiated). Most importantly, a plasma sample for CMV PCR testing at the central laboratory should be collected at this visit. Thereafter, the subject can be treated according to the local standard of care (outside the context of the study). Such subjects will however, continue to be followed in the study (despite initiation of anti-CMV therapy) and complete all remaining study visits). At such subsequent visits, all procedures specified in the Trial Flow Chart should be completed.

Note: It is mandatory to send a plasma sample for CMV DNA PCR testing to the central laboratory **immediately prior to** (i.e., on the day of) initiating treatment for CMV disease or PET in **ALL** instances.

In the event confirmatory test results from the central laboratory are not available within the timeframe the investigator wishes to initiate anti-CMV therapy, the investigator may use a positive local laboratory test result (CMV DNA PCR or pp65 antigen only) in order to make the decision. When local laboratory test results are used for initiating anti-CMV therapy, two plasma samples for CMV DNA PCR testing must be sent to the central laboratory. The first sample must be collected immediately prior to (i.e., on the day of) initiating anti-CMV therapy. The second sample must be collected 48-72 hours after initiating anti-CMV therapy. The local laboratory result must also be reported in such instances.

Subjects who are discontinued from the study for any reason will not be replaced. Subjects who discontinue the study early up to Week 24 post-transplant should complete an Early Discontinuation Visit. Subjects who discontinue the study early after Week 24 post-

transplant should have the procedures scheduled for the Week 48 post-transplant visit completed at the time of discontinuation from the study.

In order to ensure safe trial conduct, an independent, unblinded, external Data Monitoring Committee (DMC) will be established for ongoing safety evaluation. The first safety assessment will be done when approximately 10% of the randomized subjects (~54 subjects, 36 on MK-8228 and 18 on placebo) either complete study therapy through Week 14 (~100 days) post-transplant or discontinue therapy prior to that time point. Thereafter, the DMC will review all safety data approximately every 6 months during the trial (or at longer time intervals in case of slow enrollment). In addition, the DMC will also assess futility (i.e., lack of efficacy) when approximately 40% of randomized subjects either complete study therapy through Week 14 (~100 days) post-transplant or discontinue prior to that time point. The DMC will review the safety and interim futility data, consider the overall risk and benefit of continuing the trial to study participants, and make a recommendation to the Executive Oversight Committee (EOC) whether the trial should continue in accordance with the protocol.

Additionally, an independent, blinded Clinical Adjudication Committee (CAC) will be established. This CAC will review clinical, virological, and histopathological data as well as the investigator's assessments for adjudicating all potential cases of CMV end-organ disease, as defined in Appendix 12.4, throughout the trial. The adjudication of cases by the CAC will take precedence over the investigator's assessment.

Population (sparse) pharmacokinetics will be performed on all subjects in this trial. Intensive pharmacokinetic testing will be performed on a subset of subjects (~100 subjects including ~67 on MK-8228 and ~ 33 on placebo). Viral resistance testing will be performed in subjects with clinically significant CMV infection.

Specific procedures to be performed during the trial, as well as their prescribed times and associated visit windows, are outlined in the Trial Flow Chart - Section 6.0. Details of each procedure are provided in Section 7.0 – Trial Procedures.

This study will be conducted in conformance with Good Clinical Practices.

2.2 Trial Diagram

The trial design is depicted in **Figure 1**.

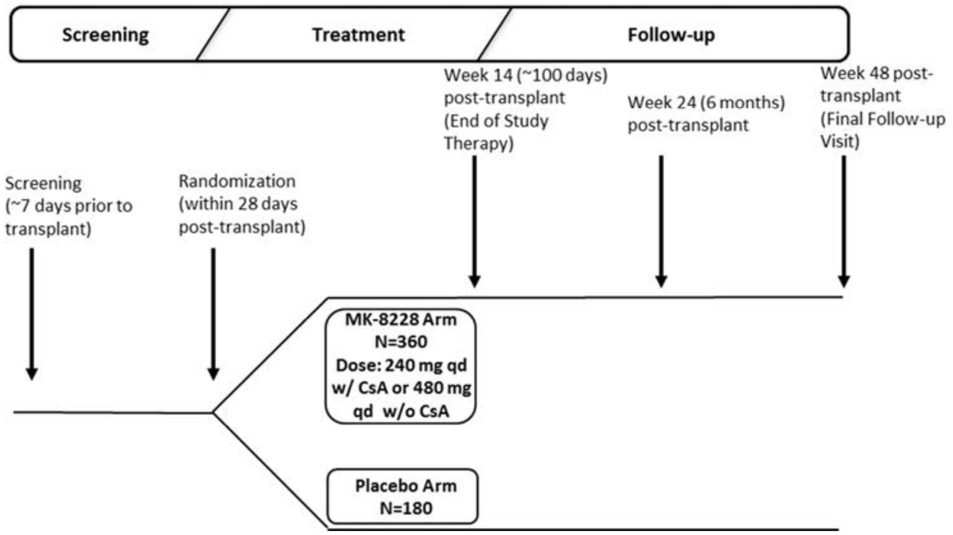

Figure 1 Trial Diagram

3.0 OBJECTIVE(S) & HYPOTHESIS(ES)

3.1 Primary Objective(s) & Hypothesis(es)

1) **Objective:** To evaluate the efficacy of MK-8228 in the prevention of clinically significant CMV infection through Week 24 (~6 months) post-transplant following administration of MK-8228 or placebo.

   Hypothesis: MK-8228 is superior to placebo in the prevention of clinically significant CMV infection, as assessed by the proportion of subjects with CMV end-organ disease or initiation of anti-CMV pre-emptive therapy (PET) based on documented CMV viremia and the subject's clinical condition through Week 24 (~6 months) post-transplant.

3.2 Secondary Objective(s) & Hypothesis(es)

1) **Objective**: To evaluate the safety and tolerability of MK-8228.
2) **Objective**: To evaluate the efficacy of MK-8228 in the prevention of clinically significant CMV infection through Week 14 (~100 days) post-transplant.
3) **Objective**: To evaluate the efficacy of MK-8228 as assessed by time to onset of clinically significant CMV infection through Week 24 (~6 months) post-transplant.
4) **Objective:** To determine the incidence of CMV disease through Week 14 post-transplant and Week 24 post-transplant.
5) **Objective:** To assess the incidence of PET for CMV viremia through Week 14 post-transplant and Week 24 post-transplant.

6) **Objective:** To assess the time to initiation of PET for CMV viremia through Week 14 post-transplant and Week 24 post-transplant.

3.3 Exploratory Objectives

1) **Objective:** To determine the incidence of CMV disease through Week 48 post-transplant.
2) **Objective:** To determine the incidence of all-cause mortality through Week 14 post-transplant, Week 24 post-transplant, and Week 48 post-transplant.
3) **Objective:** To determine the incidence of opportunistic infection other than CMV infection (i.e., systemic bacterial and invasive fungal infection) through Week 14 post-transplant, Week 24 post-transplant, and Week 48 post-transplant.
4) **Objective:** To determine the incidence of acute and/or chronic graft-versus-host disease (GVHD) after randomization through Week 14 post-transplant, Week 24 post-transplant, and Week 48 post-transplant.
5) **Objective:** To determine the incidence of all re-hospitalizations (following initial hospital discharge) and re-hospitalizations for CMV infection/disease through Week 14 post-transplant, Week 24 post-transplant, and Week 48 post-transplant.
6) **Objective:** To assess the incidence of CMV viremia through Week 14 post-transplant and Week 24 post-transplant.
7) **Objective:** To assess the time to CMV viremia through Week 14 post-transplant and Week 24 post-transplant.
8) **Objective:** To determine the incidence of engraftment through Week 14 post-transplant and Week 24 post-transplant. (Engraftment is defined as documented absolute neutrophil counts ≥ 500/mm^3 on 3 consecutive days.)
9) **Objective:** To determine the time to engraftment through Week 14 post-transplant and Week 24 post-transplant.
10) **Objective:** To evaluate antiviral resistance to MK-8228 in prophylaxis failures.
11) **Objective:** To assess quality of life using the EuroQol (EQ)-5D and Functional Assessment of Cancer Therapy (FACT-BMT) questionnaires through Week 48 post-transplant.
12) **Objective:** To evaluate the pharmacokinetics (PK) of MK-8228.

4.0 BACKGROUND & RATIONALE

4.1 Background

CMV continues to be an important complication after allogeneic HSCT [1,2]. The clinical effects of CMV can be divided into direct and indirect effects [1,2]. The direct effects which have been extensively described include the spectrum of CMV disease manifestations. CMV gastroenteritis is the most common clinical presentation in this population. While pneumonia is the most serious manifestation, it has become relatively infrequent with current preventative strategies for CMV disease in HSCT recipients [2,3]. Other rare manifestations of CMV disease include hepatitis, retinitis and encephalitis [1,2]. The indirect effects of CMV include its immunosuppressive effects, which can lead to an increased incidence of systemic bacterial and invasive fungal disease as well as acute and chronic GVHD [2,4].

Recipient CMV seropositivity remains associated with poor outcomes especially in high risk patients such as unrelated donors or cord blood recipients [2,5]. The source of stem cells and the conditioning regimens may also influence both the time to reactivation as well as the severity of disease [5-7]. For preventive purposes, both antiviral prophylaxis and PET (the practice of active surveillance for viral replication and initiating treatment with the detection of viremia) with antivirals are used to manage CMV reactivation in HSCT patients [1]. A recent international survey documented that PET with ganciclovir (GCV) is more commonly used for the purpose, though GCV toxicity is associated with significant myelosuppression and prolonged neutropenia [1,2,7,8]. All currently available anti-CMV agents are associated with significant toxicity and resistance to and cross-resistance across these antiviral agents is increasingly being reported. Thus, antivirals are not routinely used for the prophylaxis of CMV infection in HSCT patients, and there is a clear need for safe and well-tolerated drugs with novel mechanisms of action against CMV that can be used for prophylaxis in HSCT patients.

MK-8228 (letermovir) is an antiviral agent with potent reversible activity against CMV with a novel mechanism of action. It has generally been safe and well tolerated in Phase I and II trials. This study will evaluate the safety and efficacy of MK-8228 in preventing clinically significant CMV infection in adult, CMV IgG-seropositive, allogeneic HSCT patients.

4.1.1 Pharmaceutical and Therapeutic Background

Refer to the Investigator's Brochure (IB) and Appendix 12.6 for detailed background information on MK-8228.

There is no oral anti-CMV drug approved for prophylaxis of CMV disease in HSCT patients. Use of GCV, valganciclovir (VGCV), and foscarnet are limited by their toxicity profiles (prolonged myelosuppression, and renal toxicity and electrolyte abnormalities, respectively) [9]. Acyclovir and valacyclovir have limited efficacy against the virus [2,8,10]. Additionally, there is increasing emergence of resistance and cross-resistance to currently available antiviral agents [9,11]. Thus, there is an urgent need for newer efficacious agents with better tolerability and novel mechanisms of action for CMV prophylaxis in HSCT patients.

MK-8228, which belongs to a new class of anti-CMV agents, has a novel mechanism of action. It has demonstrated potent, selective, and reversible inhibition of CMV activity in

preclinical studies *in vitro* and efficacy against the virus *in vivo* [11,12]. It inhibits the viral terminase complex (UL56/UL89), an enzyme that plays an important role in cleavage of viral deoxyribonucleic acid (DNA) into unit-length genome and packaging it into procapsids [12,13].

While drug resistance remains rare, most resistance arises during treatment with GCV (or VGCV) as they are used in ~ 90% of patients as first-line agents. Drug resistance is usually seen after treatment with these antiviral agents for duration of weeks to months [9]. GCV and VGCV undergo intracellular phosphorylation by a viral kinase, which is encoded by the CMV gene UL97 during infection and the majority of resistance mutations with use of these therapies map to this gene [11]. It has been postulated that UL97 mutations arise first and confer moderate resistance to GCV (or VGCV) but not to other CMV antivirals, such as cidofovir or foscarnet.

However, all current anti-CMV agents act through the viral polymerase (UL54) and resistance mapping to this gene product leads to cross-resistance among all available agents [11]. MK-8228, through its novel mechanism of action may offer a viable alternative against virus resistant to current anti-CMV drugs. To date, no cross-resistance has been demonstrated between this agent and GCV, foscarnet, cidofovir and acyclovir [14].

4.2 Rationale

4.2.1 Rationale for the Trial and Selected Subject Population

Details regarding specific benefits and risks for subjects participating in this clinical trial may be found in the accompanying IB and Informed Consent documents.

CMV is one of the most important complications after allogeneic HSCT. It can cause multi-organ disease in HSCT recipients, including pneumonia, hepatitis, gastroenteritis, retinitis, and encephalitis [1,2]. Disease can develop both early (within 3 months) and late (> 3 months) after transplantation [15-18]. Recipient CMV seropositivity (IgG) remains a risk factor for mortality associated with transplantation despite major advances in early diagnosis and management of CMV replication and disease [17-21].

CMV infection and disease are associated with “direct effects” - the clinical manifestations of the disease - as well as “indirect effects” [1,2]. Indirect effects include increased risk of opportunistic bacterial and invasive fungal infections in patients with CMV infection [2,4, 22]. Another indirect effect of CMV infection includes the association between CMV and either acute and/or chronic GVHD. Seropositive patients with acute GVHD are at an increased risk of CMV disease [23-25]. Conversely, CMV infection can be a risk factor for acute GVHD in patients receiving T-cell-depleted grafts, as well as for chronic GVHD [19,26,27].

Existing treatment options for CMV are associated with significant toxicity, and there is an unmet medical need for an efficacious anti-CMV drug without dose-limiting toxicities such as bone marrow suppression or renal toxicity in this patient population. Additionally, there is increasing emergence of cross-resistance to existing anti-CMV drugs, albeit at a low rate. MK-8228 (letermovir) belongs to a new class of anti-CMV agents with a novel mechanism of action with:

a) significant anti-CMV activity both *in vitro* and *in vivo* studies,
b) a favorable safety profile demonstrated in several Phase I and Phase II trials, and
c) activity against viral isolates resistant to marketed anti-CMV agents which map to the UL-54 or UL-97 genes as MK-8228 activity maps to the UL56 (terminase) gene [14].

Accordingly, this trial will investigate the safety and efficacy of MK-8228 administered through Week 14 (~100 days) post-transplant for the prevention of clinically significant CMV infection in adult, CMV IgG seropositive recipients of allogeneic HSCT. The trial will include subjects particularly at high risk for CMV disease as defined in Section 2.1 (Trial Design).

4.2.2 Rationale for Dose Selection/Regimen

Please refer to the MK-8228 IB for further details of preclinical data and study results in humans.

Rationale for Dose Selection

MK-8228 belongs to a new class of anti-CMV agents which have a different mechanism of action compared to currently available drugs for the treatment of CMV infection. By inhibiting the viral terminase complex, the drug plays a key role in cleavage and packaging of genomic virus DNA into provirions.

MK-8228 is anticipated to be efficacious based on both the *in vitro* potency of letermovir as well as its *in vivo* efficacy in a Phase IIB dose-ranging trial (AIC246-01-II-02) in HSCT recipients. *In vitro*, the drug exhibits potent activity against CMV in cell cultures with EC_{90} values in the nanomolar range.

The drug has also been shown to be efficacious in the prophylaxis of CMV disease in a Phase IIB study of HSCT recipients (AIC246-01-II-02). In this trial, three doses of MK-8228, 60 mg qd (n=33), 120 mg qd (n=31) and 240 mg qd (n=34), were compared to placebo (n=31) when given over 84 days for CMV prophylaxis.

One of the primary efficacy endpoints, the incidence of overall failure of CMV prophylaxis over the 84 day treatment period, was significantly reduced in the primary population, the Full Analysis Set (FAS), with the 120 mg and 240 mg doses of MK-8228 (32%, p=0.014 and 29%, p=0.007, respectively) when compared to placebo. However, the second primary efficacy endpoint, the time to onset of overall failure was significantly reduced in the 240-mg arm alone (p=0.002), but not the 120-mg arm (p=0.126), compared to placebo in the FAS. Furthermore, all sensitivity analyses confirmed the statistical significance of both primary endpoints in the FAS for the 240-mg once-daily dose of MK-8228 versus placebo.

Of note, the only 2 subjects in the 240-mg arm who failed CMV prophylaxis in the study had CMV viremia on the first day of treatment, indicating pre-treatment CMV replication. Therefore, in effect, there was no CMV prophylaxis failure with the 240-mg once-daily dose of MK-8228 when excluding subjects with active CMV replication on Day 1 of treatment.

In a Phase IIA proof-of-concept trial (AIC001-2-001), 18 subjects were treated with 40 mg bid or 80 mg qd for 14 days. MK-8228 was generally safe and well tolerated in this study. Similarly, in the Phase IIB dose ranging study (AIC246-01-II-02, described above), 98 subjects were treated with 60 mg qd, 120 mg qd, or 240 mg qd of MK-8228 for 84 days. All doses were well tolerated with a safety profile similar to placebo.

MK-8228 has been safe and generally well tolerated in 14 Phase I and the Phase II clinical trials. In the Phase I trials MK-8228 was administered in single oral or IV doses ranging from 5 mg to 960 mg (n=235) in healthy male and female subjects, and multiple oral or IV doses ranging from 30 mg qd to 320 mg bid (n=230) in healthy subjects as well as subjects with moderate to severe hepatic impairment for up to 14.5 days. In these trials, there were no deaths and there was one serious adverse event (SAE) which was not considered to be drug-related. Similarly, MK-8228 was safe and well-tolerated in 116 transplant patients in the Phase II studies who were exposed to doses ranging from 80 mg qd/40 mg bid to 240 mg qd for up to 84 days.

In all the above trials, MK-8228 had no significant effects on vital signs, clinical or laboratory parameters, or electrocardiogram (ECG).

Phase I studies have demonstrated that co-administrations with cyclosporin A (CsA) increases MK-8228 exposure ~3 fold. Further analyses using the Phase IIB study data indicate that exposure with the 240 mg dose of MK-8228 administered alone overlaps exposure levels of the 60 and 120 mg once-daily doses which are associated with virologic failures. Most such failures occurred at MK-8228 levels with AUC_{tau} values < 45,000 ng*h/mL. Consequently, an efficacy target for success in > 90% of the subjects, was set at AUC_{tau} levels ≥ 45,000 ng*h/mL. It is predicted that this target level will be achieved with a dose of 240 mg of MK-8228 once daily in subjects receiving CsA, and with 480 mg of MK-8228 once daily in the absence of CsA. Modeling and simulation data indicate that exposure levels of MK-8228 with the 480 mg dose in the absence of CsA will not exceed exposure levels seen with the 240 mg dose of MK-8228 when administered with CsA in the Phase IIB data (n=18). These exposure levels were not also associated with any significantly increased adverse events when compared to placebo used in the study. Additional preliminary population PK analyses indicate that other covariates including gender and weight do not have a meaningful effect on MK-8228 exposure.

Based on all available safety data, MK-8228 efficacy in the Phase II studies, and the exposure-response data, this study will use a dose of 240 mg qd for subjects receiving CsA and 480 mg qd in subjects who are not receiving CsA concomitantly.

Rationale for Study Duration

Most HSCT patients are at highest risk for CMV disease within the first 3 months (~100 days) after transplantation. Antiviral prophylaxis with MK-8228 was efficacious when used for 84 days in the Phase IIB (AIC246-01-II-02) study outlined above. Therefore, study therapy with MK-8228 will be used for antiviral prophylaxis through Week 14 (~100 days)

post-transplant in this study. Subjects will then be followed through Week 24 post-transplant (an additional 10 weeks following completion of study therapy) in order to evaluate the incidence of late-onset CMV infection/disease. Finally, further information regarding CMV disease, health-outcomes measures (such as incidence of all-cause mortality, re-hospitalizations, GVHD, and opportunistic infections), as well as quality of life (QoL) measures, will be collected up to Week 48 post-transplant.

4.2.3 Rationale for Endpoints

4.2.3.1 Efficacy Endpoints

The primary efficacy endpoint of the study will be the proportion of subjects with clinically significant CMV infection through Week 24 (~6 months) post-transplant, defined as the occurrence of either one of the following outcomes:

- onset of CMV end-organ disease

  OR

- initiation of anti-CMV PET based on documented CMV viremia (as measured by the central laboratory) and the clinical condition of the subject. Initiation of PET in this study refers to the practice of initiating therapy with the following approved anti-CMV agents when active CMV viral replication is documented: ganciclovir, valganciclovir, foscarnet, and/or cidofovir.

CMV disease will be determined using the definitions in Appendix 12.4 and confirmed by an independent, blinded Clinical Adjudication Committee (CAC). The CAC will review clinical, virological, and histopathological data as well as the investigator's assessments throughout the trial for adjudicating all potential cases of CMV disease. The adjudication of cases by the CAC will take precedence over the investigator's assessment.

Currently, with most centers using CMV preventive strategies, including PET, the overall incidence of CMV disease in HSCT patients has declined to around 5% in the first 3 months post-transplant, from 20-30% prior to the routine use of preventive measures [2,4,6,19,28-31]. Accordingly, sample sizes required to show efficacy of novel anti-CMV drugs for antiviral prophylaxis using the incidence of CMV disease alone would be high [27,31]. Thus, the primary endpoint of this study will also include the incidence of anti-CMV PET initiation based on detection of CMV viremia and the clinical condition of the subject.

As detection of CMV in plasma or blood is associated with an increased risk of CMV disease [32-35], CMV viral DNA as a measure of CMV infection is already used routinely in clinical practice to initiate and monitor PET [2,9,29,36,37]. Patients with high viral loads or with cumulative high viral loads are at an increased risk of developing disease than those with lower viral loads [36,37]. However, there is no clinically validated viral load threshold for initiating pre-emptive therapy at this point in time. Some centers initiate pre-emptive therapy using a single cut-off value for viremia, while others use a risk-based approach for the purpose [2,9].

In this study, CMV viremia (viral load) will be measured on plasma samples using the Roche COBAS® AmpliPrep/COBAS TaqMan® (CAP/CTM) assay, which will be performed by the central laboratory. The lower limit of quantification (LLoQ) for this assay is 137 IU/ml which is ~150 copies/mL (using a conversion factor of 1.1 copies/IU as per the assay package insert). Results will be reported as:

- < LLoQ, not detected
- < LLoQ, detected
- CMV titer results within the linear range of the test
- > ULoQ (upper limit of quantification): CMV titer results above the linear range of the test

While any detectable CMV viral DNA results in the Roche CAP/CTM assay from the central laboratory is acceptable for the purpose of documenting viremia as a component of the primary endpoint, it is strongly recommended that investigators should not initiate PET when CMV viral load is below the LLoQ, but detectable (<LLoQ, detected). The guidance regarding viral load thresholds for initiation of PET in this trial are based on risk as defined in the study stratification (see Section 2.1, Trial Design) as well as consideration of standard practice described in ref. 9, and are as follows:

- High risk: single viral DNA ≥150 copies/mL
- Low risk: single viral DNA >300 copies/mL or confirmed viral DNA between 150 - 300 copies/mL (with the second value higher than the first)

While the viral threshold suggested for initiating PET in low-risk patients in the reference cited above [9] may be as high as 1,000 copies/ml by the assay used at the Fred-Hutchinson Cancer Research Center (FHCRC), this viral DNA level corresponds to a level of ~ 300 copies/ml using the Roche CAP/CTM assay, which will be used in this study (1,000 copies/ml in the FHCRC assay ~ 250 IU/ml in the Roche CAP/CTM assay ~ 275 copies/ml in the Roche CAP/CTM assay, PPD [redacted] personal communication]). Importantly, these thresholds for initiation of PET are provided as guidance based on the subject's risk for CMV disease at baseline. However, specific thresholds for initiating PET are not mandated per protocol as a subject's risk status and clinical condition may change during the course of the trial and is best assessed by the investigator taking care of the subject.

Note: **All** study-related CMV DNA PCR samples must be sent to the central laboratory at the designated time points in the Trial Flow Chart (Section 6.0). It is strongly recommended that investigators use viremia (at levels > LLoQ) detected by the central laboratory to drive decisions for initiating PET. It is mandatory to send a plasma sample for CMV DNA PCR testing to the central laboratory **immediately prior to** (i.e., on the day of) initiating treatment for CMV disease or PET.

In the event test results from the central laboratory are not available within the timeframe the investigator wishes to initiate anti-CMV therapy, the investigator may use a positive local laboratory test (CMV DNA PCR or pp65 antigen only) result in order to make the decision. When local laboratory test results are used for initiating anti-CMV therapy, two plasma samples for CMV DNA PCR testing must be sent to the central laboratory. The first sample must be collected immediately prior to (i.e., on the day of) initiating anti-CMV therapy. The second sample must be collected 48-72 hours after initiating anti-CMV therapy. The local laboratory result must also be reported in such instances.

4.2.3.2 Safety Endpoints

The safety and tolerability of MK-8228 will be assessed by a clinical evaluation of adverse experiences and evaluation of other study parameters including vital signs, physical examination, 12-lead ECGs, and standard laboratory safety tests at appropriate time points as specified in the Trial Flow Chart (Section 6.0). Serum inhibin B, luteinizing hormone (LH), follicle-stimulating hormone (FSH), and testosterone levels in men will be collected to monitor testicular function. Adverse experiences should be graded and recorded as outlined in Section 7.2. Subjects may be asked to return for unscheduled visits for additional safety monitoring.

4.2.3.3 Pharmacokinetic Endpoints

Population PK will be performed on all subjects. Intensive PK will be performed on a subset of subjects (~100 subjects, including ~67 on MK-8228 and ~33 on placebo). The PK endpoints for MK-8228 are: area under the concentration-time curve to the end of the dosing period (AUC_{0-24}), area under the concentration-time curve up to the last measurable concentration (AUC_{0-last}), maximum concentration observed (C_{max}, for subjects receiving tablet formulation), time to maximum observed plasma drug concentration (t_{max}, for subjects receiving tablet formulation), concentration at the end of infusion (C_{eoi}, for subjects receiving IV formulation), and minimum concentration observed (C_{trough}). Plasma samples for pharmacokinetic (PK) evaluations will be collected at the time points described in the Trial Flow Chart (Section 6.0).

4.2.3.4 Future Biomedical Research

The Sponsor will conduct Future Biomedical Research on specimens routinely and specifically collected during this clinical trial. This research may include genetic analyses (DNA), and/or the measurement of other analytes.

Such research is for biomarker testing to address emergent questions not described elsewhere in the protocol (as part of the main trial) and will only be conducted on specimens from appropriately consented subjects. The objective of collecting specimens for Future Biomedical Research is to explore and identify biomarkers that inform the scientific understanding of diseases and/or their therapeutic treatments. For instance, exploratory pharmacogenetic (PGt) studies may be performed if significant Pharmacokinetic/Pharmacodynamic (PK/PD) relationships are observed or adverse events are identified. Genomic markers of disease may also be investigated. Such retrospective pharmacogenetic studies will be conducted with appropriate biostatistical design and analysis

and compared to PK/PD results or clinical outcomes. Any significant PGt relationships to outcome would require validation in future clinical trials. The overarching goal is to use such information to develop safer, more effective drugs, and/or to ensure that subjects receive the correct dose of the correct drug at the correct time. The details of this Future Biomedical Research sub-trial are presented in Section 12.2 - Collection and Management of Specimens for Future Biomedical Research. Additional informational material for institutional review boards/ethics committees (IRBs/ERCs) and investigational site staff is provided in Section 12.3.

5.0 METHODOLOGY

5.1 Entry Criteria

5.1.1 Diagnosis/Condition for Entry into the Trial

Male/Female subjects with receipt of an allogeneic HSCT of at least 18 years of age will be enrolled in this trial.

5.1.2 Subject Inclusion Criteria

In order to be eligible for participation in this trial, the subject must:

1. be ≥ 18 years of age on the day of signing informed consent.
2. have documented seropositivity for CMV (recipient CMV IgG seropositivity [R+]) within 1 year before HSCT.
3. be receiving a first allogeneic HSCT (bone marrow, peripheral blood stem cell, or cord blood transplant).
4. be within 28 days post-HSCT at the time of randomization.
5. be highly unlikely to become pregnant or to impregnate a partner since they meet at least one of the following criteria:
   a) A female subject who is not of reproductive potential is eligible without requiring the use of contraception. A female subject who is not of reproductive potential is defined as one who: (1) has reached natural menopause (defined as 6 months of spontaneous amenorrhea with serum follicle-stimulating hormone [FSH] levels in the postmenopausal range as determined by the local laboratory, or 12 months of spontaneous amenorrhea); (2) is 6 weeks post-surgical bilateral oophorectomy with or without hysterectomy; or (3) has undergone bilateral tubal ligation. Spontaneous amenorrhea does not include cases for which there is an underlying disease that causes amenorrhea (e.g., anorexia nervosa).
   b) A male subject who is not of reproductive potential is eligible without requiring the use of contraception. A male subject who is not of reproductive potential is defined as one whom has undergone a successful vasectomy. A successful vasectomy is defined as: (1) microscopic documentation of azoospermia, or (2) a vasectomy more than 2 years ago with no resultant pregnancy despite sexual activity postvasectomy.

c) A male or female subject who is of reproductive potential agrees to true abstinence or to use (or have their partner use) 2 acceptable methods of birth control starting from the time of consent through 90 days after the last dose of study therapy. Longer periods of birth control may be required per local requirements. True abstinence is defined as abstinence in line with the preferred and usual lifestyle of the subject. Periodic abstinence (e.g., abstinence only on certain calendar days, abstinence only during ovulation period, use of symptothermal method, use of post-ovulation methods) and withdrawal are not acceptable methods of contraception. Acceptable methods of birth control are: intrauterine device (IUD), diaphragm with spermicide, contraceptive sponge, condom, and vasectomy OR use of appropriate double barrier contraception as per local regulations or guidelines. Hormonal contraceptives (e.g., birth control pills, transdermal patch, or injectables) are unacceptable methods of birth control for use in this study because it is not known whether these methods are affected by co-administration of MK-8228.

6. be able to read, understand, and complete questionnaires and diaries.
7. understand the study procedures, alternative treatment available, and risks involved with the study, and voluntarily agree to participate by giving written informed consent. The subject may also provide consent for Future Biomedical Research. However, the subject may participate in the main trial without participating in Future Biomedical Research.

5.1.3 Subject Exclusion Criteria

The subject must be excluded from participating in the trial if the subject:

1. received a previous allogeneic HSCT (**Note**: Receipt of a previous autologous HSCT is acceptable).
2. has a history of CMV end-organ disease within 6 months prior to randomization.
3. has evidence of CMV viremia from a central or local laboratory at any time prior to randomization.
4. received within 7 days prior to screening or plans to receive during the study any of the following:
   - ganciclovir
   - valganciclovir
   - foscarnet
   - acyclovir (at doses > 3200 mg PO per day or > 25 mg/kg IV per day)
   - valacyclovir (at doses > 3000 mg PO per day)
   - famciclovir (at doses > 1500 mg PO per day)
5. received within 30 days prior to screening or plans to receive during the study any of the following:
   - cidofovir
   - CMV hyper-immune globulin
   - Any investigational CMV antiviral agent/biologic therapy

6. has suspected or known hypersensitivity to active or inactive ingredients of letermovir formulations.
7. has severe hepatic insufficiency (defined as Child-Pugh Class C; see Appendix 12.5).
8. has serum aspartate aminotransferase (AST) or alanine aminotransferase (ALT) > 5 x the upper limit of normal (ULN) or serum total bilirubin > 2.5 x ULN.

   Note: Subjects who meet this exclusion criterion may, at the discretion of the investigator, have one repeat testing done. If the repeat value does not meet this criterion, they may continue in the screening process. Only the specific out of range value should be repeated (not the entire panel).
9. has end-stage renal impairment with a creatinine clearance less than 10 mL/min, as calculated by the Cockcroft-Gault equation using serum creatinine.

   $$\text{Creatinine Clearance (Males)} = \frac{\text{(weight in kg) (140 – age)}}{\text{(72) (creatinine in mg/dL)}}$$

   Creatinine Clearance (Females) = 0.85 x the value obtained with formula above

   Note: Subjects who meet this exclusion criterion may, at the discretion of the investigator, have one repeat testing done. If the repeat value does not meet this criterion, they may continue in the screening process. Only the specific out of range value should be repeated (not the entire panel).
10. has both moderate hepatic insufficiency AND moderate renal insufficiency.

    Note: Moderate hepatic insufficiency is defined as Child Pugh Class B (see Appendix 12.5); moderate renal insufficiency is defined as a creatinine clearance less than 50 mL/min, as calculated by the Cockcroft-Gault equation (as above), respectively.
11. has an uncontrolled infection on the day of randomization.
12. requires mechanical ventilation or is hemodynamically unstable at the time of randomization.
13. has documented positive results for human immunodeficiency virus antibody (HIV-Ab), hepatitis C virus antibody (HCV-Ab) with detectable HCV RNA, or hepatitis B surface antigen (HBsAg) within 90 days prior to randomization.
14. has active solid tumor malignancies with the exception of localized basal cell or squamous cell skin cancer or the condition under treatment (e.g., lymphomas).
15. is pregnant or expecting to conceive, is breastfeeding, or plans to breastfeed from the time of consent through 90 days after the last dose of study therapy.
16. is expecting to donate eggs or sperm starting from the time of consent through 90 days after the last dose of study therapy.

17. is currently participating or has participated in a study with an *unapproved* investigational compound or device within 28 days, or 5X half-life of the investigational compound (excluding monoclonal antibodies), whichever is longer, of initial dosing on this study. Subjects previously treated with a monoclonal antibody will be eligible to participate after a 28-day washout period.

    Note: Investigational chemotherapy regimens involving *approved* agents and investigational antimicrobial regimens involving *approved* antibacterial/antifungal/antiviral agents, investigational radiotherapy studies, or other observational studies are allowed.

18. has previously participated in this study or any other study involving MK-8228 (letermovir).
19. has previously participated or is currently participating in any study involving administration of a CMV vaccine or another CMV investigational agent, or is planning to participate in a study of a CMV vaccine or another CMV investigational agent during the course of this study.
20. is or has an immediate family member (spouse or children) who is investigational site or Sponsor staff directly involved with this trial.
21. is, at the time of signing informed consent, a user of recreational or illicit drugs or has had a recent history (within the last year) of drug or alcohol abuse or dependence.

    Note: Subject who has a history of recreational marijuana use which is not deemed excessive by the subject's investigator or does not interfere with the subject's daily function may participate in the study but must be instructed to discontinue any further use of recreational marijuana prior to entry into trial and throughout the trial period.

22. has a history or current evidence of any condition, therapy, lab abnormality, or other circumstance that might confound the results of the study, interfere with the subject's participation for the full duration of the study, or would be put at undue risk as judged by the investigator, such that it is not in the best interest of the subject to participate in this study.

5.2 Trial Treatments

In this study, subjects will be randomized in a 2:1 ratio with ~360 subjects on MK-8228 and ~180 subjects on placebo. Both oral (tablet) and IV formulations of MK-8228 (and placebo) to be used in this trial are outlined below in Table 1 and Table 2, respectively. Subjects will be initiated with the oral (tablet) formulation of study therapy provided they are able to swallow and do not have a condition (e.g., vomiting, diarrhea, or a malabsorptive condition) that may interfere with the absorption of the tablets. For subjects who cannot swallow and/or have a condition that may interfere with the absorption of the oral formulation (e.g., vomiting, diarrhea, or a malabsorptive condition), study therapy can be initiated with or switched to the IV formulation. Use of the IV formulation should generally be limited to 4 weeks or less in duration. However, it will be left to the investigator's discretion to continue IV administration beyond 4 weeks, if the benefit/risk ratio supports continued administration. Simultaneous use of IV and oral study therapy is not allowed.

Table 1 Study Therapy – Oral (Tablet) Formulation

Drug	Dose/Potency	Dose Frequency	Route of Administration	Regimen/Treatment Period	Use
MK-8228 (for subjects on CsA)	240 mg	Once daily (qd)	Oral	Through Week 14 (~100 days) post-transplant	Experimental
MK-8228 (for subjects not on CsA)	480 mg (two 240-mg tablets)	Once daily (qd)	Oral	Through Week 14 (~100 days) post-transplant	Experimental
Placebo to MK-8228*	NA	Once daily (qd)	Oral	Through Week 14 (~100 days) post-transplant	Placebo-comparator

CsA = Cyclosporin A

*The number of placebo tablets will mimic the MK-8228 dosing scheme in an effort to maintain the blind (i.e., 1 placebo tablet if on CsA; 2 placebo tablets if not on CsA).

Table 2 Study Therapy – IV Formulation

Drug	Dose/Potency	Dose Frequency	Route of Administration	Regimen/Treatment Period	Use
MK-8228 (for subjects on CsA)	240 mg	Once daily (qd)	Intravenous*	Through Week 14 (~100 days) post-transplant**	Experimental
MK-8228 (for subjects not on CsA)	480 mg	Once daily (qd)	Intravenous*	Through Week 14 (~100 days) post-transplant**	Experimental
Placebo to MK-8228	NA	Once daily (qd)	Intravenous*	Through Week 14 (~100 days) post-transplant**	Placebo-comparator

CsA = Cyclosporin A.

*The method of administration including the concentration, volume, rate, and duration of infusion is provided in the Investigator Trial File Binder.

**The IV formulation should be switched to oral study therapy (i.e., at the next planned dose) as soon as such subjects are able to swallow and/or the condition necessitating the use of the IV formulation resolves.

Study therapy may begin as early as the day of transplant to no later than 28 days post-transplant, once the subject is determined to be negative for CMV viremia (no evidence of CMV viremia from a central or local laboratory at any time point **and** confirmed by the central laboratory on a sample collected from the subject within 7 days prior to randomization).

5.2.1 Dose Selection

5.2.1.1 Dose Selection

The rationale for selection of doses to be used in this trial is provided in Section 4.0 – Background & Rationale. There are no specific calculations or evaluations required to be performed in order to administer the proper dose to each subject.

Both IV and oral (tablet) formulations of MK-8228 (and matching placebo) will be available. Study therapy should be initiated with the oral formulation in all subjects who are able to swallow and do not have any condition that may interfere with the absorption of oral medication (e.g., vomiting, diarrhea, or a malabsorptive condition).

The IV formulation of MK-8228 contains the excipient hydroxylpropyl-β-cyclodextrin, which can accumulate in patients with renal insufficiency. In this regard, cyclodextrin is also an excipient in other approved IV agents, including IV voriconazole (given with the excipient, sulfolbutylether-β-cyclodextrin). Recent data suggest that, despite accumulation of cyclodextrin in patients with reduced renal function, baseline renal function is not a predictor of worsening renal function in patients receiving IV voriconazole [38]. Additionally, the quantity of cyclodextrin in the MK-8228 IV formulation is less than that in the IV voriconazole formulation (1800 mg of cyclodextrin/vial for a 240-mg dose of MK-8228 as compared to 3200 mg of cyclodextrin/vial for a 200-mg dose of voriconazole, which is administered twice a day).

Based on the above, the use of IV MK-8228 is permitted in subjects with renal insufficiency, provided creatinine clearance is >10 mL/min. However, the IV formulation should only be used when subjects are either unable to swallow or have a condition (e.g., vomiting, diarrhea, or malabsorptive condition) that may interfere with the absorption of the oral formulation. Subjects on IV MK-8228 should be switched to the oral formulation as soon as they are able to swallow and/or the condition that warranted the use of the IV formulation has resolved.

5.2.2 Dose Modification

The same dose of MK-8228 will be administered for both formulations. Subjects in the MK-8228 treatment group will receive either 240 mg MK-8228 qd, if receiving concomitant CsA, or 480 mg MK-8228 qd, if not on CsA. If CsA is initiated after starting study therapy, the next dose of MK-8228 (administered up to 24 hours later) should be decreased from 480 mg qd to 240 mg qd. If CsA is discontinued in a subject already receiving study therapy, the next dose of MK-8228 (administered up to 24 hours later) should be increased from 240 mg qd to 480 mg qd. Depending on CsA co-administration, the number of placebo tablets administered will be the same as MK-8228 tablets, in order to maintain study blind.

5.2.3 Timing of Dose Administration

Study therapy should be administered/taken at the same time each day. Tablets are to be swallowed whole (i.e., no crushing or chewing the tablet is allowed). Study therapy may be administered with or without food.

If a subject misses a dose, the missed dose should be given as soon as possible during the same day. If more than 18 hours have gone by after the regular dosing time, then the missed dose should be skipped and the normal dosing schedule should be resumed. The next dose should not be doubled in order to "make up" what has been missed.

5.2.4 Trial Blinding/Masking

A double-blind/masking technique will be used. Oral MK-8228 and matching placebo (given as oral tablet) will be packaged identically so that treatment blind/masking is maintained. The subject, the investigator and Sponsor personnel or delegate(s) who are involved in the treatment or clinical evaluation of the subjects are unaware of the treatment group assignments.

The oral (tablet) formulation will be packaged identically so that treatment blind/masking is maintained. A placebo image to MK-8228 will be implemented to maintain study blinding and placebo tablets will be indistinguishable from MK-8228. Subjects on CsA will receive either one tablet of 240 mg MK-8228 or one tablet of placebo. Subjects not on CsA will receive either two tablets of 240 mg MK-8228 or two tablets of placebo.

Table 3 Blinding of Tablets Related to CsA Use

	MK-8228 Group	Placebo Group
On CsA	One 240-mg MK-8228 tablet	One placebo tablet
Off CsA	Two 240-mg MK-8228 tablets	Two placebo tablets

IV MK-8228 and matching placebo (given as an IV infusion) will be prepared in a blinded fashion by an unblinded pharmacist (or qualified trial site personnel). Normal saline will be used as the placebo to IV MK-8228. The Sponsor will provide opaque covers for the IV bags and tubing in order to assist with blinding study therapy.

The IV formulation will be dispensed in a blinded fashion by an unblinded pharmacist (or qualified trial site personnel). Because this is a double-blind study, the investigator, study personnel, and subject must remain blinded to the IV study therapy. In order to maintain the blinding, the unblinded pharmacist (or qualified trial site personnel designated to prepare IV study therapy) will be responsible solely for the preparation and administration of the IV study therapy. He/she will not be involved in evaluating subjects for efficacy or safety.

See Section 7.1.4.2, Blinding/Unblinding, for a description of the method of unblinding a subject during the trial, should such action be warranted.

5.3 Randomization or Treatment Allocation

Randomization will occur centrally using an interactive voice response system/integrated web response system (IVRS/IWRS). There are 2 treatment arms. Subjects will be assigned randomized treatment in an 2:1 ratio to MK-8228 and matching placebo, respectively. With approximately 540 subjects enrolled in this study, ~360 will receive MK-8228 and ~180 will receive placebo.

5.4 Stratification

Randomization will be stratified according to the following factors:

Subjects will be stratified by:

1. Study center, and
2. Risk factor group. Risk factor groups include high or low risk, as defined below:

(i) <u>High risk</u>: Subjects meeting <u>one or more of the following criteria</u> at the time of randomization:

- Human leukocyte antigen (HLA)-related (sibling) donor with at least one mismatch at one of the following three HLA-gene loci: HLA-A, -B or -DR,
- Haploidentical donor,
- Unrelated donor with at least one mismatch at one of the following four HLA-gene loci: HLA-A, -B, -C and -DRB1,
- Use of umbilical cord blood as stem cell source,
- Use of *ex vivo* T-cell-depleted grafts,
- Grade 2 or greater graft-versus-host disease (GVHD), requiring the use of systemic corticosteroids (defined as the use of ≥ 1 mg/kg/day of prednisone or equivalent dose of another corticosteroid).

(ii) <u>Low risk</u>: All subjects <u>not</u> meeting definition of high risk.

5.5 Concomitant Medications/Vaccinations (allowed & prohibited)

Medications or vaccinations specifically prohibited in the exclusion criteria are not allowed during the ongoing trial. Listed below are some specific restrictions for concomitant therapy or vaccination during the course of the trial. If there is a clinical indication for one of these or other medications or vaccinations specifically prohibited during the trial, discontinuation from trial therapy or vaccination may be required. The investigator should discuss any questions regarding this with the local Clinical Monitor. The final decision on any supportive therapy or vaccination rests with the investigator and/or the subject's primary physician. However, the decision to continue the subject on trial therapy or vaccination schedule requires the mutual agreement of the investigator, the Sponsor and the subject.

Allowed Medications/Therapies

The following medications/therapies are **allowed** in this study.

- Standard antimicrobial prophylaxis (e.g., levofloxacin for bacteria, fluconazole/ voriconazole/posaconazole for fungi)
- Acyclovir, valacyclovir, or famciclovir for prophylaxis of herpes simplex virus (HSV) or varicella zoster virus (VZV) infections at doses no greater than prohibited doses of these medications (see below)
- All types of prior conditioning regimens (including myeloablative, reduced-intensity, or non-myeloablative regimens)
- Prior/Ongoing graft manipulation regimens (including various *ex-vivo* or *in-vivo* T-cell depletion or selection regimens)
- GVHD prophylaxis regimens

Prohibited Medications/Therapies

The following medications/therapies are **prohibited** in this study.

Antiviral drugs or therapies for prevention/treatment of CMV, including but not limited to:

- ganciclovir
- valganciclovir
- foscarnet
- cidofovir
- acyclovir (at doses > 3200 mg PO per day or > 25 mg/kg IV per day)
- valacyclovir (at doses > 3000 mg PO per day)
- famciclovir (at doses > 1500 mg PO per day)
- CMV hyper-immune globulin
- any investigational CMV antiviral agent/biologic therapy
- CMV vaccine

Investigational Agents

Investigational agents are not permitted with the following exceptions: (1) Investigational chemotherapy regimens involving *approved* agents and (2) investigational antimicrobial regimens involving *approved* antibacterial/antifungal/antiviral agents.

Medications/Therapies to be Administered with Caution

The following medications/therapies are not prohibited, but should be **used with caution**.

Preclinical studies suggest MK-8228 acts as a weak to moderate inhibitor of cytochrome (CYP)3A4, CYP2C8, CYP2B6 and the transporters OATP1B1 and OATP1B3. It is therefore possible that MK-8228 may increase the exposure of co-administered drugs whose primary route of clearance involves these enzymes or transporters.

CYP3A substrates with narrow therapeutic range, including but not limited to:

- fentanyl
- amiodarone, flecainide, propafenone, quinidine
- pimozide
- ergot derivatives
- midazolam and triazolam
- sildenafil or tadalafil when used for the treatment of pulmonary arterial hypertension
- drosperinone
- lovastatin and simvastatin
- cisapride
- alfuzosin
- warfarin
- astemizole
- itraconazole

CYP2C8 substrates with narrow therapeutic range, including but not limited to:

- mycophenolate mofetil

OATP1B1 substrates, including but not limited to:

- statins
- bosentan
- glyburide

Concomitant Immunosuppressant Use

Levels of cyclosporine and tacrolimus have been shown to increase ~ 2 fold with MK-8228 co-administration. Therefore, levels of cyclosporine, tacrolimus, sirolimus and everolimus should be closely monitored by the investigator, especially when initiating study therapy or changing the dosage of study therapy, as dose adjustments of these immunosuppressant agents may be needed when co-administered with MK-8228.

5.6 Rescue Medications & Supportive Care

In the event of clinically significant CMV infection (CMV disease or initiation of PET based on CMV viremia and the clinical condition of the subject [see Section 4.2.3.1]) at any time during the 48 week post-transplant period, study therapy will be discontinued (if the subject is on study therapy) and the subject may be treated according to the local standard of care (outside the context of the study). In this setting, any of the prohibited anti-CMV medications (outlined in Section 5.5) may be used.

5.7 Diet/Activity/Other Considerations

Study therapy can be administered with or without food.

Subjects must avoid consumption of grapefruit, Seville oranges or their respective juices, and other quinine-containing drinks or food during the trial from 2 weeks prior to study drug administration until 72 hours after the final administration of study drug.

5.8 Subject Withdrawal/Discontinuation Criteria

Subjects may withdraw consent at any time for any reason or be dropped from the trial at the discretion of the investigator should any untoward effect occur. In addition, a subject may be withdrawn by the investigator or the Sponsor if enrollment into the trial is inappropriate, the trial plan is violated, or for administrative and/or other safety reasons. Specific details regarding discontinuation or withdrawal procedures; including specific details regarding withdrawal from Future Biomedical Research, are provided in Section 7.1.4 – Other Procedures.

In this trial, a subject may discontinue from treatment but continue to participate in the regularly scheduled activities, as long as the subject does not withdraw consent. Discontinuation from treatment is permanent. Once a subject has discontinued treatment, even though he/she continues to be monitored in the trial, he/she shall not be allowed to begin treatment again.

A subject must be discontinued from the trial for any of the following reasons:

- The subject or legal representative (such as a parent or legal guardian) withdraws consent.

A subject **must** be discontinued from study therapy (<u>but should continue to be monitored in the study</u>) for any of the following reasons:

- The subject meets the criteria for clinically significant CMV infection (see Section 4.2.3.1).
- The subject becomes pregnant during the study.
- The subject's investigator feels it is in the best interest of the subject to discontinue.
- The subject **may** be discontinued from study therapy for any of the following reasons:
  - Any AE/SAE assessed by the physician investigator as possibly or probably related to study therapy. Investigator may continue the subject in the trial if it is deemed to be in the best interest of the subject to stay on study therapy.
  - Failure to comply with the dosing, evaluations, or other requirements of the trial.
- The subject has a medical condition or personal circumstance which, in the opinion of the investigator and/or Sponsor, places the subject at unnecessary risk through continued participation in the trial or does not allow the subject to adhere to the requirements of the protocol.

<u>**For subjects who develop clinically significant CMV infection during the study treatment period (up to Week 14 post-transplant):**</u> When the investigator intends to initiate either treatment for CMV disease or PET, the subject should have a CMV Infection Visit. It is very important to ensure that all procedures, as outlined in the Trial Flow Chart (Section 6.0), are performed at the CMV Infection Visit <u>**immediately prior to**</u> the initiation of treatment of CMV diseases or initiation of PET (i.e., on the day anti-CMV therapy is initiated). Most importantly, a plasma sample for CMV PCR testing at the central laboratory should be collected at this visit. Thereafter, the subject should be discontinued from study therapy and treated according to the local standard of care (outside the context of the study).

Such subjects will however, continue to be followed in the study (despite discontinuing study therapy and initiating anti-CMV therapy) and complete all remaining study visits. At such subsequent visits, all procedures specified in the Trial Flow Chart should be completed with the exception of study therapy administration, pharmacokinetic (PK) assessments, and study medication diary review.

For subjects with clinically significant CMV infection during the post-treatment [follow-up] period (after Week 14 and through Week 24 post-transplant): When an investigator intends to initiate either treatment for CMV disease or PET, the subject should have a CMV Infection Visit. It is very important to ensure that all procedures, as outlined in the Trial Flow Chart (Section 6.0), are performed at the CMV Infection Visit **immediately prior to** the initiation of treatment of CMV diseases or initiation of PET (i.e., on the day anti-CMV therapy is initiated). Most importantly, a plasma sample for CMV PCR testing at the central laboratory should be collected at this visit. Thereafter, the subject can be treated according to the local standard of care (outside the context of the study). Such subjects will however, continue to be followed in the study (despite initiation of anti-CMV therapy) and complete all remaining study visits. At such subsequent visits, all procedures specified in the Trial Flow Chart should be completed.

Note: It is mandatory to send a plasma sample for CMV DNA PCR testing to the central laboratory **immediately prior to** (i.e., on the day of) initiating treatment for CMV disease or PET in **ALL** instances.

In the event confirmatory test results from the central laboratory are not available within the timeframe the investigator wishes to initiate anti-CMV therapy, the investigator may use a positive local laboratory test result (CMV DNA PCR or pp65 antigen only) in order to make the decision. When local laboratory test results are used for initiating anti-CMV therapy, two plasma samples for CMV DNA PCR testing must be sent to the central laboratory. The first sample must be collected immediately prior to (i.e., on the day of) initiating anti-CMV therapy. The second sample must be collected 48-72 hours after initiating anti-CMV therapy. The local laboratory result must also be reported in such instances.

Subjects who are discontinued from the study for any reason will not be replaced. Subjects who discontinue the study early up to Week 24 post-transplant should complete an Early Discontinuation Visit. Subjects who discontinue the study early after Week 24 post-transplant should have the procedures scheduled for the Week 48 post-transplant visit completed at the time of discontinuation from the study.

5.9 Subject Replacement Strategy

A subject that discontinues from the trial will not be replaced.

5.10 Beginning and End of the Trial

The overall trial begins when the first subject signs the informed consent form. The overall trial ends when the last subject completes the last trial visit, discontinues from the trial or is lost to follow-up (i.e. the subject is unable to be contacted by the investigator).

5.11 Clinical Criteria for Early Trial Termination

Early trial termination will be the result of the criteria specified below:

1. The trial is deemed to be futile based on the results of the interim analysis for futility.
2. The EOC determines that the extent (incidence and/or severity) of emerging adverse effects/clinical endpoints is such that the risk/benefit ratio to the trial population as a whole is unacceptable.

6.0 TRIAL FLOW CHART

Visit No.	1	2	3	4	5	6	7	8	9	10	11	12	13	14	15	16	17	18	19	20	21	22	23	24	25
	Screen	Treatment (Week 1)		Treatment (Weeks 2 to 14[c])													Post-treatment Follow-up (Weeks 15 to 24[c])					Post-treatment Follow-up (Weeks 25 to 48[c])			
Week No. (unless otherwise indicated)	SCR[a]	Day1[b]	Day 7	2	3	4	5	6	7	8	9	10[d]	11[d]	12[d]	13[d]	End of Study Therapy Visit 14[d]	16	18	20	22	24	32	40	48	CMV Infection or Early Discon Visit[e]
Follow-up (FU) Week No.																	FU2	FU4	FU6	FU8	FU10	FU18	FU26	FU34	
Visit Window			±2 days	±3 days													±4 days					±2 weeks			
**Administrative Procedures**																									
Informed Consent	X																								
Informed Consent for Future Biomedical Research (optional)	X																								
Inclusion/Exclusion Criteria	X	X																							
Subject Identification Card	X																								
Medical History	X																								
Prior/Concomitant Medication Review[f]	X	X	X	X	X	X	X	X	X	X	X	X	X	X	X	X	X	X	X	X	X	X	X	X	X
Study Therapy Allocation/ Randomization		X																							
HSCT Details Review[g]		X																							
Study Medication Diary Review[h]		X	X	X	X	X	X	X	X	X	X	X	X	X	X	X									X

Visit No.	1	2	3	4	5	6	7	8	9	10	11	12	13	14	15	16	17	18	19	20	21	22	23	24	25
	Screen	Treatment (Week 1)		Treatment (Weeks 2 to 14[c])													Post-treatment Follow-up (Weeks 15 to 24[c])					Post-treatment Follow-up (Weeks 25 to 48[c])			
Week No. (unless otherwise indicated)	SCR[a]	Day1[b]	Day 7	2	3	4	5	6	7	8	9	10[d]	11[d]	12[d]	13[d]	End of Study Therapy Visit 14[d]	16	18	20	22	24	32	40	48	CMV Infection or Early Discon Visit[e]
Follow-up (FU) Week No.																	FU2	FU4	FU6	FU8	FU10	FU18	FU26	FU34	
Visit Window			±2 days	±3 days													±4 days					±2 weeks			
**Clinical Procedures/ Assessments**																									
Physical Examination[i]	X	X	X	X	X	X	X	X	X	X	X	X	X	X	X	X	X								X
Weight	X															X									X
Height	X																								
Vital Signs (heart rate, blood pressure, respiratory rate, body temperature)[j]	X	X	X	X	X	X	X	X	X	X	X	X	X	X	X	X	X								X
12-Lead Electrocardiogram[k]	X			X												X									
Child-Pugh Score (see Appendix 12 5)	x																								
Subject Confirmation of Birth Control[l]	X	X	X	X	X	X	X	X	X	X	X	X	X	X	X	X	X	X	X	X	X				X
Adverse Events Monitoring[m]	X	X	X	X	X	X	X	X	X	X	X	X	X	X	X	X	X	X	X	X	X	X	X	X	X

Visit No.	1	2	3	4	5	6	7	8	9	10	11	12	13	14	15	16	17	18	19	20	21	22	23	24	25
	Screen	Treatment (Week 1)		Treatment (Weeks 2 to 14[c])													Post-treatment Follow-up (Weeks 15 to 24[c])					Post-treatment Follow-up (Weeks 25 to 48[c])			
Week No. (unless otherwise indicated)	SCR[a]	Day1[b]	Day 7	2	3	4	5	6	7	8	9	10[d]	11[d]	12[d]	13[d]	End of Study Therapy Visit 14[d]	16	18	20	22	24	32	40	48	CMV Infection or Early Discon Visit[e]
Follow-up (FU) Week No.																	FU2	FU4	FU6	FU8	FU10	FU18	FU26	FU34	
Visit Window			±2 days	±3 days													±4 days					±2 weeks			
**Laboratory Procedures/ Assessments[n]**																									
Chemistry/ Hematology	X[o]	X		X		X				X				X		X	X								X
Urinalysis	X[o]	X														X									X
Serum β-Human Chorionic Gonadotropin (β-hCG) in women of childbearing potential	X																								
Urine Pregnancy Test in women of childbearing potential		X				X				X						X									X
Serum inhibin B, LH, FSH, testosterone levels in men		X														X					X				X
HIV/Hepatitis B, C Screen[p]	X																								
Blood for Future Biomedical Research (optional)[q]		X																							

Visit No.	1	2	3	4	5	6	7	8	9	10	11	12	13	14	15	16	17	18	19	20	21	22	23	24	25
	Screen	Treatment (Week 1)		Treatment (Weeks 2 to 14[c])													Post-treatment Follow-up (Weeks 15 to 24[c])					Post-treatment Follow-up (Weeks 25 to 48[c])			
Week No. (unless otherwise indicated)	SCR[a]	Day1[b]	Day 7	2	3	4	5	6	7	8	9	10[d]	11[d]	12[d]	13[d]	End of Study Therapy Visit 14[d]	16	18	20	22	24	32	40	48	CMV Infection or Early Discon Visit[e]
Follow-up (FU) Week No.																	FU2	FU4	FU6	FU8	FU10	FU18	FU26	FU34	
Visit Window			±2 days	±3 days													±4 days					±2 weeks			
**CMV Procedures/ Assessments**																									
CMV DNA PCR[r]	X	X	X	X	X	X	X	X	X	X	X	X	X	X	X	X	X	X	X	X	X	X	X	X	X
CMV Disease Assessment (see Appendix 12.4)	X	X	X	X	X	X	X	X	X	X	X	X	X	X	X	X	X	X	X	X	X	X	X	X	X
Health Outcomes Assessment[s]		X				X				X						X	X		X		X	X	X	X	X
Quality of Life Assessment[t]		X														X					X			X	X
CMV DNA Sequence Analysis[u]																									X
**Pharmacokinetics**																									
Population PK[v]			X	X		X		X		X		X		X		X									
Intensive PK[w]			X																						
**Study Therapy Administration**																									
MK-8228/Placebo[x,y]		X	X	X	X	X	X	X	X	X	X	X	X	X	X	X									

a. Screening may begin 7 days prior to transplantation. Subjects will have plasma samples tested for CMV viremia using the CMV DNA PCR assay (for initial screening purposes, results of the assay done at a local laboratory will be acceptable). After establishing absence of CMV viremia, subjects will be tested once a week by the central laboratory using the CMV DNA PCR assay from the time of transplantation until randomization (Day 1).

b. Start of study therapy is Day 1. On the day of randomization, eligibility for enrollment into the study should be confirmed (including confirmation that HSCT has taken place). At that time, subjects should have no documented CMV viremia, as confirmed by CMV DNA PCR assay at the central laboratory (the Roche COBAS® AmpliPrep/COBAS TaqMan® [CAP/CTM] assay) in a plasma sample collected within 7 days prior to randomization. Creatinine clearance and liver function test results within 7 days prior to randomization should also be available and be within the range allowable in this study, as outlined in Section 5.1.3 (Subject Exclusion Criteria). Study therapy may begin as early as the day of transplant and no later than 28 days post-transplant. Study therapy will continue through Week 14 (~100 days) post-transplant. Day 1 procedures/assessments must be performed prior to first dose of study therapy.

c. Weeks correspond to the end of the numbered weeks after randomization, i.e., Week 2, 3, 4, etc. corresponds to Days 14, 21, 28 after randomization, respectively.

d. End of Study Therapy Visit may occur on Week 10, 11, 12, 13, or 14 depending on when study therapy was started during the 28-day post-transplant window. For example, if study therapy was started on the day of transplant, the End of Study Therapy Visit would be the Week 14 Visit (which corresponds to Week 14 post-transplant). If study therapy was started 28 days post-transplant, the End of Study Therapy Visit would be the Week 10 Visit (which corresponds to Week 14 post-transplant).

e. The visit will be a CMV Infection Visit for all subjects who discontinue study therapy due to clinically significant CMV infection defined as the occurrence of CMV disease or the initiation of PET, or for subjects who are either diagnosed with CMV disease or require initiation of PET after study therapy completion during the follow-up period (through Week 24 post-transplant). The visit will be an Early Discontinuation Visit for those subjects who are prematurely discontinued from the trial up to Week 24 post-transplant. All procedures should be performed at this visit **immediately prior to** the initiation of treatment of CMV diseases or initiation of PET (i.e., on the day anti-CMV therapy is initiated). Most importantly, a plasma sample for CMV PCR testing at the central laboratory should be collected at this visit.

f. Includes review of consumption of grapefruit, Seville oranges or their respective juices, and other quinine-containing drinks or food. **Anti-CMV medications administered for treatment of CMV disease or for initiation of PET and all drug/biologic therapies used to prevent/treat GVHD should be recorded at every visit through Week 48 post-transplant.** During the follow-up period (after Week 14 through Week 48 post-transplant), concomitant medication review is limited to the above and all antimicrobials (antibacterials, antifungals, antivirals), chemotherapy agents, and immunosuppressant agents.

g. To collect all relevant data at randomization (Day 1) related to the recent HSCT including details regarding the conditioning regimen used, the date and type of transplant, the source of stem cells, type of graft manipulation, presence of GVHD, and GVHD prophylaxis regimen (if any) used.

h. Study Medication Diary completion and review will begin once subject is discharged from the hospital.

i. After randomization (Day 1), the physical examination does not need to be performed at every visit; a targeted physical exam should be performed only if a subject has any complaints.

j. Vital signs include heart rate (sitting), blood pressure (sitting), respiratory rate, and body temperature (oral). Subjects should be resting in a semi-recumbent position for at least 10 minutes prior to measurement of vital signs. After randomization (Day 1), vital signs should only be performed if targeted physical examination is performed.

k. All 12-Lead ECGs will be obtained after the subject has remained in a semi-recumbent position for 10 minutes.

l. Subject must use acceptable methods of contraception from the time of consent through 90 days after the last dose of study therapy.

m. Adverse event monitoring should include the collection of all adverse events while on study therapy and for 2 weeks following completion of study therapy (i.e., through Follow-up Week 2 Visit) for all subjects including those who have discontinued study therapy but are continuing in the study. Thereafter, only drug related serious adverse events (SAEs) and SAEs leading to death will be collected through Week 48 post-transplant. AEs occurring prior to study therapy administration, as a result of protocol-specified procedure or intervention, should also be reported. Refer to Section 7.2 (Assessing and Recording Adverse Events) for further details.

n. Refer to Section 7.1.3 (Laboratory Procedures/Assessments) for further details regarding the laboratory safety tests.

o. For screening purposes, values from the subject's chart for required chemistry, hematology, and urinalysis tests are acceptable. If not available, this testing should be performed by the central laboratory.

p. Perform HIV/Hepatitis B, C Screen only if not previously documented within the last 90 days. If hepatitis C virus antibody is positive, RNA PCR results should be provided (or, if not available, RNA PCR testing will be performed by the central laboratory).

q. Participation in the Future Biomedical Research component of this study is optional. Informed consent for future biomedical research samples must be obtained before the DNA sample is collected. DNA for analysis should be obtained predose, on Day 1 (or with the next scheduled blood draw), as the last sample drawn, on randomized subjects only, or at a later date as soon as the informed consent is obtained.

r. For initial screening, results of CMV DNA PCR assay performed at a local laboratory will be acceptable to establish absence of CMV viremia. From the time of transplantation until randomization, CMV DNA PCR testing will be performed once a week by the central laboratory using the Roche CAP/CTM Assay. Thereafter, CMV DNA PCR testing will be performed by the central laboratory at every visit as specified. A plasma sample for CMV PCR testing at the central laboratory will also be collected at the CMV Infection or Early Discontinuation Visit. When local laboratory test results are used for initiating anti-CMV therapy, two samples for CMV DNA PCR testing must be sent to the central laboratory. The first sample must be collected immediately prior to (i.e., on the day of) initiating anti-CMV therapy. The second sample must be collected 48-72 hours after initiating anti-CMV therapy. In such instances the results of the local laboratory CMV PCR must also be reported. If the subject consents to the Future Biomedical Research sub-study, any leftover samples from CMV DNA PCR will be stored for future research.

s. To collect information such as all-cause mortality, re-hospitalizations, GVHD, opportunistic infections (i.e., systemic bacterial and invasive fungal infections), and engraftment.

t. The EuroQol (EQ)-5D and Functional Assessment of Cancer Therapy – Bone Marrow Transplant (FACT-BMT) questionnaires which are validated patient reported outcome tools will be used to measure quality of life.

u. CMV DNA Sequence Analysis to be performed only in subjects with clinically significant CMV infection.

v. Population (sparse) PK samples will be collected in all subjects. The 8 samples at the indicated visits will be collected 0 – 2 hours pre-dose. As treatment may range 10 – 14 weeks, the Week 12 and Week 14 visit samples may not be collected in all subjects.

w. Intensive PK sampling will be performed in a subset of ~100 subjects. The 5 samples will be collected at the Day 7 visit at the following time points: pre-dose, 1 hour (± 10 min) following oral administration (or within less than 10 min **before or after** infusion completion, **but not during** infusion completion, when given IV), 2.5 hours following oral/IV administration (± 30 min), 8 hours following oral/IV administration (range of 6-10 hours), and 24 hours following oral/IV administration (range of 22-24 hours; 0-2 hours prior to next day's dose).

x. Both oral (tablet) and intravenous (IV) formulation of MK-8228 (and placebo) will be available for study therapy. Subjects will be initiated with the oral (tablet) formulation of study therapy provided they are able to swallow and do not have a condition (e.g., vomiting, diarrhea, or malabsorptive condition) that may interfere with the absorption of the tablets. For subjects who cannot swallow and/or have a condition that may interfere with the absorption of the oral formulation, study therapy can be initiated with or switched to the IV formulation. The IV formulation should be switched to oral study therapy (i.e., at the next planned dose) as soon as such subjects are able to swallow and/or the condition necessitating the use of the IV formulation resolves. Use of the IV formulation should generally be limited to 4 weeks or less in duration. However, it will be left to the investigator's discretion to continue IV administration beyond 4 weeks, if the benefit/risk ratio supports continued administration.

y. In this study, the dose of MK-8228 will change based on whether concomitant CsA is received. The same dose of MK-8228 will be administered for both formulations. Subjects in the MK-8228 treatment group will receive either 240 mg MK-8228 qd, if receiving concomitant CsA, or 480 mg MK-8228 qd, if not on CsA. If CsA is initiated after starting study therapy, the next dose of MK-8228 (administered up to 24 hours later) should be decreased to 240 mg qd. If CsA is discontinued in a subject, the next dose of MK-8228 (administered up to 24 hours later) should be increased from 240 mg to 480 mg qd. Corresponding changes in tablets for oral formulation with changes in CsA dosing will also occur in the placebo group in an effort to maintain the study blind (see Section 5.2.3).

7.0 TRIAL PROCEDURES

7.1 Trial Procedures

The Trial Flow Chart - Section 6.0 summarizes the trial procedures to be performed at each visit. Individual trial procedures are described in detail below. It may be necessary to perform these procedures at unscheduled time points if deemed clinically necessary by the investigator.

Furthermore, additional evaluations/testing may be deemed necessary by the investigator and or the Sponsor for reasons related to subject safety. In some cases, such evaluation/testing may be potentially sensitive in nature (e.g., HIV, Hepatitis C, etc.), and thus local regulations may require that additional informed consent be obtained from the subject. In these cases, such evaluations/testing will be performed in accordance with those regulations.

7.1.1 Administrative Procedures

7.1.1.1 Informed Consent

The investigator must obtain documented consent from each potential subject prior to participating in a clinical trial or Future Biomedical Research.

7.1.1.1.1 General Informed Consent

Consent must be documented by the subject's dated signature or by the subject's legally acceptable representative's dated signature on a consent form along with the dated signature of the person conducting the consent discussion.

A copy of the signed and dated consent form should be given to the subject before participation in the trial.

The initial informed consent form, any subsequent revised written informed consent form and any written information provided to the subject must receive the IRB/ERC's approval/favorable opinion in advance of use. The subject or his/her legally acceptable representative should be informed in a timely manner if new information becomes available that may be relevant to the subject's willingness to continue participation in the trial. The communication of this information will be provided and documented via a revised consent form or addendum to the original consent form that captures the subject's dated signature or by the subject's legally acceptable representative's dated signature.

Specifics about a trial and the trial population will be added to the consent form template at the protocol level.

The informed consent will adhere to IRB/ERC requirements, applicable laws and regulations and Sponsor requirements.

7.1.1.1.2 Consent and Collection of Specimens for Future Biomedical Research

The investigator or qualified designee will explain the Future Biomedical Research consent to the subject, answer all of his/her questions, and obtain written informed consent before performing any procedure related to the Future Biomedical Research sub-trial. A copy of the informed consent will be given to the subject.

7.1.1.2 Inclusion/Exclusion Criteria

All inclusion and exclusion criteria will be reviewed by the investigator or qualified designee to ensure that the subject qualifies for the trial at screening and on Day 1 (randomization).

7.1.1.3 Subject Identification Card

All subjects will be given a Subject Identification Card identifying them as participants in a research trial. The card will contain trial site contact information (including direct telephone numbers) to be utilized in the event of an emergency. The investigator or qualified designee will provide the subject with a Subject Identification Card after the subject provides written informed consent.

7.1.1.4 Medical History

A medical history will be obtained by the investigator or qualified designee at screening.

7.1.1.5 Prior and Concomitant Medications Review

7.1.1.5.1 Prior Medications

The investigator or qualified designee will review prior medication use, including any protocol-specified washout requirement, and record prior medication taken by the subject within 30 days prior to screening. This includes review of consumption of grapefruit, Seville oranges or their respective juices, and other quinine-containing drinks or food.

7.1.1.5.2 Concomitant Medications

The investigator or qualified designee will record medication, if any, taken by the subject during the trial. Anti-CMV medications administered for treatment of CMV disease or for initiation of PET and all drug/biologic therapies used to prevent/treat GVHD should be recorded at every visit through Week 48 post-transplant. During the follow-up period (after Week 14 through Week 48 post-transplant), concomitant medication review is limited to the above and all antimicrobials (antibacterials, antifungals, antivirals), chemotherapy agents, and immunosuppressant agents.

7.1.1.6 HSCT Details Review

All relevant data about the HSCT will be collected on Day 1 (at randomization). This includes details regarding the conditioning regimen used, the date and type of transplant, the source of stem cells, type of graft manipulation, presence of GVHD, and GVHD prophylaxis regimen (if any) used.

7.1.1.7 Assignment of Screening Number

All consented subjects will be given a unique screening number that will be used to identify the subject for all procedures that occur prior to randomization or treatment allocation. Each subject will be assigned only one screening number. Screening numbers must not be re-used for different subjects. Any subject who is screened multiple times will retain the original screening number assigned at the initial screening visit.

7.1.1.8 Assignment of Randomization Number

All eligible subjects will be randomly allocated to trial treatment and will receive a randomization number. The randomization number identifies the subject for all procedures occurring after randomization. Once a randomization number is assigned to a subject, it can never be re-assigned to another subject.

A single subject cannot be assigned more than 1 randomization number.

7.1.1.9 Trial Compliance (Study Therapy)

The Study Medication Diary will be completed electronically via a hand-held device once the subject is discharged from the hospital. The investigator/study coordinator will train the subject in the use of the electronic Study Medication Diary. The subject will be instructed to enter the number of tablets of study therapy taken during the study therapy period. At visits when used/unused study therapy are returned, site personnel must verify the accuracy of the dosing diary by comparing entries with amounts of returned study therapy. If a discrepancy is noted, the investigator/study coordinator must discuss the discrepancy with the subject, and the explanation must be documented. The investigator/study coordinator will be responsible for transferring the appropriate information from the Study Medication Diary to the appropriate case report form.

Interruptions from the protocol specified treatment plan for ≥7 days require consultation between the investigator and the Sponsor and written documentation of the collaborative decision on subject management.

7.1.2 Clinical Procedures/Assessments

7.1.2.1 Physical Examination

All physical examinations must be performed by the principal investigator or subinvestigator (physician, physician assistant or nurse practitioner).

A complete physical examination, performed at the screening visit and Day 1 (randomization), includes the following assessments: general appearance, head, eyes, ears/nose/throat, neck, lymph nodes, skin, lungs, heart, abdomen, musculoskeletal, and neurologic evaluations. Breast, rectal, and genitourinary/pelvic exams should be performed when clinically indicated.

After randomization (Day 1), the physical examination does not need to be performed at every visit; a <u>targeted</u> physical exam should be performed only if a subject has any complaints. The timing of physical examinations is indicated in the Trial Flow Chart (Section 6.0).

7.1.2.2 Weight and Height Assessment

The subject's weight and height will be assessed as indicated in the Trial Flow Chart (Section 6.0).

7.1.2.3 Vital Signs

Vital signs will be assessed as indicated in the Trial Flow Chart (Section 6.0).

Vital signs will include heart rate (sitting), blood pressure (sitting), respiratory rate, and body temperature (oral). Subjects should be resting in a semi-recumbent position for at least 10 minutes prior to measurement of vital signs.

Note: Oral temperatures should be taken orally, but if oral is not possible, tympanic, rectal, and axillary temperatures are acceptable.

After Day 1 (randomization), vital signs should only be performed if targeted physical examination is performed.

7.1.2.4 12-Lead Electrocardiogram

12-Lead ECG measurements will be performed using a central vendor as indicated in the Trial Flow Chart (Section 6.0).

Special care must be taken for proper lead placement. Subjects should be shaved as necessary for proper lead placement. Subjects should be resting in a semi-recumbent position for at least 10 minutes prior to having ECG readings obtained.

7.1.2.5 Child Pugh Score

The Child Pugh Score will be assessed at screening according to Appendix 12.5.

7.1.2.6 Birth Control Confirmation

Subjects must use acceptable methods of contraception from the time of consent through 90 days after the last dose of study therapy. Confirmation must be obtained by site personnel that subjects and their partner(s) are using acceptable methods of contraception. This assessment must be documented in the subject's study chart at each specified visit.

7.1.2.7 Adverse Events Monitoring

Adverse event monitoring will include the collection of all adverse events while on study therapy and for 2 weeks following completion of study therapy (i.e., through Follow-up Week 2 Visit) in all subjects, including those who have discontinued study therapy but continue to be followed-up in the study. Thereafter, only drug related serious adverse events (SAEs) and SAEs leading to death will be collected through Week 48 post-transplant. AEs occurring prior to study therapy administration, as a result of protocol-specified procedure or intervention, should also be reported. Refer to Section 7.2 (Assessing and Recording Adverse Events) for further details.

7.1.2.8 CMV Disease Assessment

CMV disease will be assessed from screening through Week 48 post-transplant. Diagnostic criteria for the evaluation of CMV disease are outlined in Appendix 12.4. The investigator will ensure that clinical information, radiology results, and specimens for the appropriate diagnostic tests (including, but not limited to, viral culture, histopathology, immunohistochemical analysis, *in situ* hybridization, CMV PCR) as outlined in Appendix 12.4 will be collected.

7.1.2.9 Health Outcomes Assessment

Information such as all-cause mortality, re-hospitalizations, GVHD, opportunistic infections (i.e., systemic bacterial and invasive fungal infections), and engraftment will be collected as part of the Health Outcomes Assessment for this study as indicated in the Trial Flow Chart (Section 6.0).

7.1.2.10 Quality of Life Assessment

Two questionnaires, the EuroQol (EQ)-5D version 3L and the Functional Assessment of Cancer Therapy – Bone Marrow Transplant (FACT-BMT) version 4, are validated tools of patient reported outcomes. These 2 questionnaires will be used to assess quality of life (QoL). These QoL assessments will be collected at the time points indicated in the Trial Flow Chart (Section 6.0).

The EQ-5D consists of five general health questions and a visual analog scale.

The FACT-BMT comprises 47 questions measuring the following domains: physical well-being, social/family well-being, emotional well-being, functional well-being and additional concerns related to the subject's clinical condition.

These measures will be completed electronically via a hand-held device. The investigator/study coordinator will train the subject in the use of the device. The subject will be instructed to complete these measures at the designated visits prior to any study procedures. The investigator/study coordinator will also confirm that the measures have been completed prior to performing any study procedures.

7.1.3 Laboratory Procedures/Assessments

Details regarding specific laboratory procedures/assessments to be performed in this trial are provided below. The total amount of blood/tissue to be drawn/collected over the course of the trial (from pre-trial to post-trial visits), including approximate blood/tissue volumes drawn/collected by visit and by sample type per subject can be found in Section 12.7.

7.1.3.1 Laboratory Safety Evaluations (Hematology, Chemistry and Urinalysis)

The specific laboratory tests for hematology, chemistry and urinalysis to be performed within this study are specified in Table 4. The time points for these assessments are indicated in the Trial Flow Chart (Section 6.0).

Table 4 Laboratory Tests

**Hematology**[a]	**Chemistry**[a]	**Urinalysis**[a]	**Other**
Hematocrit	Albumin	Blood	Follicle Stimulating Hormone (FSH), luteinizing hormone (LH), testosterone and inhibin B levels in males
Hemoglobin	Alkaline phosphatase	Glucose	Serum β-human chorionic gonadotropin (β-hCG)
Platelet count	Alanine aminotransferase (ALT)	Protein	Urine β-human chorionic gonadotropin (β-hCG)
WBC (total and differential)	Aspartate aminotransferase (AST)	Specific gravity	Hepatitis B surface antigen (HBsAg)[b]
	Bicarbonate	Microscopic exam, if abnormal results are noted	Hepatitis C virus antibody (HCV-Ab)[b]
	Calcium		Hepatitis C RNA PCR[b]
	Chloride		HIV antibody (HIV-Ab)[b]
	Creatinine		CMV DNA PCR[c]
	Creatinine Clearance (screening only)		CMV DNA Sequence Analysis[d]
	Glucose		
	Phosphorus		
	Potassium		
	Sodium		
	Total Bilirubin		
	Direct Bilirubin		
	Indirect Bilirubin		
	Total protein		
	Blood Urea Nitrogen		
	Prothrombin time (PT)<br>International normalized ratio (INR)		

[a] For screening, values from the subject's chart for required chemistry, hematology, and urinalysis tests are acceptable. If not available, this testing should be performed by the central laboratory.

[b] HIV/Hepatitis B, C testing only performed if results not previously documented within 90 days of screening. If hepatitis C virus antibody is positive, RNA PCR results should be provided (or, if not available, RNA PCR testing will be performed by the central laboratory).

[c] For initial screening, results of CMV DNA PCR assay performed at a local laboratory will be acceptable to establish absence of CMV viremia. Thereafter, CMV DNA PCR testing will be performed by the central laboratory using the Roche CAP/CTM Assay.

[d] CMV DNA Sequence Analysis to be performed only in subjects with clinically significant CMV infection.

7.1.3.2 Pharmacokinetic/Pharmacodynamic Evaluations

7.1.3.2.1 Blood Collection for Pharmacokinetic Sampling

Sample collection, storage, and shipment instructions for plasma samples will be provided in the operations/laboratory manual.

Population (sparse) PK samples will be collected in all subjects. The 8 samples will be collected 0-2 hours pre-dose at the Day 7, Weeks 2, 4, 6, 8, 10, 12 and 14 visits. As treatment may range 10 – 14 weeks, the Week 12 and 14 visit samples may not be collected in all subjects.

Intensive PK sampling will be performed in a subset of subjects (~100 subjects, including ~67 subjects on MK-8228 and ~33 subjects on placebo). The 5 samples will be collected at the Day 7 visit (i.e., on Days 5-9 after starting study therapy) at the following time points: pre-dose, 1 hour (± 10 min) following oral administration (or within less than 10 min **before or after** infusion completion, **but not during** infusion completion, when given IV), 2.5 hours following oral/IV administration (± 30 min), 8 hours following oral/IV administration (range of 6-10 hours), and 24 hours following oral/IV administration (range of 22-24 hours; 0-2 hours prior to next day's dose).

7.1.3.3 CMV DNA PCR Testing

Sample collection, storage, and shipment instructions for plasma samples will be provided in the operations/laboratory manual.

CMV DNA PCR testing will be performed using the Roche COBAS® AmpliPrep/COBAS TaqMan® (CAP/CTM) assay at the central laboratory. From the time of transplantation until randomization, samples must be collected **once a week** and sent to the **central laboratory**. Thereafter, samples must be collected **at every visit** and sent to the **central laboratory**, as indicated in the Trial Flow Chart (Section 6.0).

Note: It is mandatory to send a plasma sample for CMV DNA PCR testing to the central laboratory **immediately prior to** (i.e., on the day of) initiating treatment for CMV disease or PET.

In the event test results from the central laboratory are not available within the timeframe the investigator wishes to initiate anti-CMV therapy, the investigator may use a positive local laboratory test result in order to make the decision. When local laboratory test results are used for initiating anti-CMV therapy, two plasma samples for CMV DNA PCR testing must be sent to the central laboratory. The first sample must be collected immediately prior to (i.e., on the day of) initiating anti-CMV therapy. The second sample must be collected 48-72 hours after initiating anti-CMV therapy. The local laboratory result must also be reported in such instances.

7.1.3.4 CMV DNA Sequence Analysis

Sample collection, storage, and shipment instructions for plasma samples will be provided in the operations/laboratory manual.

CMV DNA Sequence Analysis to be performed only in subjects with clinically significant CMV infection. Resistance to MK-8228 will be monitored by retrospective genotypic analysis of the CMV terminase gene UL56 in CMV DNA extracts from selected plasma samples collected as indicated in the Trial Flow Chart (Section 6.0). Samples will be analyzed after unblinding by standard population sequencing technology through an established contract laboratory with validated protocols in place. In subjects with multiple CMV-positive samples, the last on-therapy and follow-up samples will be used for analysis.

CMV DNA sequencing will focus on the UL56 terminase gene. Based on in vitro selection and characterization of mutant viruses that escape inhibition of MK-8228, several mutations clustered between amino acids 230 and 370 have been identified in a distinct mutation "hot-spot" region of UL56, whereas no resistance-associated mutations have been identified thus far in other terminase subunits or putative associated viral proteins. Natural sequence polymorphisms within UL56 will be identified by comparison with a standard reference strain and with samples obtained from control arm of the trials. Variants potentially associated with viral resistance may be further characterized by recombinant phenotyping analysis, where specific variants will be engineered into bacterial artificial chromosome (BAC)-based recombinant viruses and evaluated for drug susceptibility and replication fitness.

7.1.3.5 Future Biomedical Research

The following specimens are to be obtained as part of Future Biomedical Research:

- Blood for genomics use
- Leftover main study plasma collected for CMV DNA PCR for future research

7.1.4 Other Procedures

7.1.4.1 Withdrawal/Discontinuation

When a subject discontinues/withdraws prior to trial completion, all applicable activities scheduled for the Early Discontinuation Visit (if subject discontinues/withdraws from the trial up to Week 24 post-transplant) or Week 48 post-transplant (if subject discontinues/withdraws from the trial after Week 24 post-transplant) should be performed at the time of discontinuation. Any adverse events which are present at the time of discontinuation/withdrawal should be followed in accordance with the safety requirements outlined in Section 7.2 - Assessing and Recording Adverse Events. **Note:** When a subject is discontinued from *study therapy* due to clinically significant CMV infection, a CMV Infection Visit must be performed prior to initiation of treatment of CMV diseases or initiation of PET. These subjects will continue to be followed in the study and complete all remaining study visits through Week 48 post-transplant.

7.1.4.1.1 Withdrawal From Future Biomedical Research

Subjects may withdraw their consent for Future Biomedical Research and have their specimens and all derivatives destroyed. Subjects may withdraw consent at any time by writing to the principal investigator for the main trial. If medical records for the main trial are still available, the investigator will contact the Sponsor using the designated mailbox [redacted] and a form will be provided by the Sponsor to obtain appropriate information to complete specimen withdrawal. Subsequently, the subject's specimens will be removed from the biorepository and be destroyed. A letter will be sent from the Sponsor to the investigator confirming the destruction. It is the responsibility of the investigator to inform the subject of completion of destruction. Any analyses in progress at the time of request for destruction or already performed prior to the request being received by the Sponsor will continue to be used as part of the overall research trial data and results. No new analyses would be generated after the request is received.

In the event that the medical records for the main trial are no longer available (e.g., if the investigator is no longer required by regulatory authorities to retain the main trial records) or the specimens have been completely anonymized, there will no longer be a link between the subject’s personal information and their specimens. In this situation, the request for specimen destruction cannot be processed.

7.1.4.2 Blinding/Unblinding

IVRS/IWRS should be used for emergency unblinding treatment assignment in the event that this is required for subject safety.

In the event that unblinding has occurred, the circumstances around the unblinding (e.g., date and reason) must be documented promptly, and the Sponsor Clinical Monitor notified as soon as possible. Only the principal investigator or delegate and the respective subject’s code should be unblinded. Trial site personnel and Sponsor personnel directly associated with the conduct of the trial should not be unblinded.

7.1.5 Visit Requirements

Visit requirements are outlined in Section 6.0 - Trial Flow Chart. Specific procedure-related details are provided above in Section 7.1 - Trial Procedures.

7.1.5.1 Screening

Screening of potential eligible subjects may begin 7 days prior to transplantation through randomization. Potential subjects will be evaluated to determine if they fulfill the Inclusion/Exclusion entry requirements as described in Section 5.1 (Entry Criteria). The investigator will discuss with each potential subject the nature of the study and its requirements/restrictions. All screening procedures listed under Visit 1 of the Trial Flow Chart (Section 6.0) will be performed. Subjects will be instructed that they are required to use two acceptable methods of birth control starting from the time of consent through 90 days after the last dose of study therapy (or longer if dictated by local regulations). Subjects will also be instructed about the restrictions for concomitant medications, as noted in Section 5.5.

For screening purposes, values from the subject's chart for required chemistry, hematology, and urinalysis tests are acceptable. If not available, this testing should be performed by the central laboratory. HIV, hepatitis B, and hepatitis C screening should only be performed if not previously documented within the last 90 days. If hepatitis C virus antibody is positive, RNA PCR results should be provided (or, if not available, RNA PCR testing will be performed by the central laboratory).

CMV procedures/assessments will also be performed at screening. For initial screening, results of CMV DNA PCR assay performed at a local laboratory will be acceptable to establish absence of CMV viremia. Thereafter, CMV DNA PCR testing will be performed once a week by the central laboratory, using the Roche COBAS® AmpliPrep/COBAS TaqMan® (CAP/CTM) assay, in order to exclude those with active CMV replication prior to study therapy initiation.

On the day of randomization, eligibility for enrollment into the study should be confirmed. At that time, subjects have already received their HSCT and will be considered eligible for randomization once (a) they are determined to be negative for CMV viremia (no evidence of CMV viremia from a central or local laboratory at any time point **<u>and</u>** confirmed by the central laboratory on a sample collected from the subject within 7 days prior to randomization), and (b) have acceptable creatinine clearance and liver function test values (i.e., within the range allowable in this study, as outlined in Section 5.1.3 [Subject Exclusion Criteria]) from testing performed within 7 days prior to randomization.

Presence of CMV disease in the screening period will be assessed according to Appendix 12.4.

7.1.5.2 Study Therapy Period

Study therapy (with MK-8228 or placebo) may begin as early as the day of transplant and no later than 28 days post-transplant. Study therapy will continue through Week 14 (~100 days) post-transplant. Study therapy visits will occur weekly through Week 14 (~100 days) post-transplant.

The Day 1 Visit (as shown in the Trial Flow Chart, Section 6.0) will be day the subject is randomized and study therapy is initiated. Study therapy will continue through the End of Study Therapy Visit. The End of Study Therapy Visit may occur at the Week 10, 11, 12, 13, or 14 Visit depending on when study therapy is started during the 28-day post-transplant window. For example, if study therapy is started on the day of transplant, the End of Study Therapy Visit will be the Week 14 Visit (which corresponds to Week 14 post-transplant). If study therapy is started 28 days post-transplant, the End of Study Therapy Visit will be the Week 10 Visit (which corresponds to Week 14 post-transplant).

All procedures listed under the weekly study therapy visits in the Trial Flow Chart (Section 6.0) will be performed at the corresponding visit. After randomization, the physical examination does not need to be performed at every visit; a <u>targeted</u> physical exam should be performed only if a subject has any complaints. After randomization, vital signs should only be performed if targeted physical examination is performed.

7.1.5.2.1 Day 1 Visit

Day 1 procedures/assessments listed on the Trial Flow Chart must be performed prior to initiation of study therapy. Assessment of quality of life (using FACT-BMT and EQ-5D questionnaires) should be completed prior to any study procedures at this visit.

Laboratory safety evaluations (hematology, chemistry, and urinalysis) specified in Section 7.1.3.1 will be performed prior to study therapy initiation. These samples will be sent to the appropriate central laboratory(ies) following the procedure(s) described in the manual(s).

For female subjects, a urine pregnancy test will be performed at the site prior to study therapy initiation. If the urine pregnancy test result is negative, the subject will be eligible for randomization and the remainder of the Day 1 testing/procedures will be performed. If the urine pregnancy result is positive, the subject must not be randomized.

For male subjects, serum inhibin B, luteinizing hormone (LH), follicle stimulating hormone (FSH), and testosterone testing will be performed.

7.1.5.2.2 Study Therapy Administration

Following completion of the Day 1 procedures/assessments and confirmation of eligibility (including availability of results from samples for CMV PCR, creatinine clearance, and liver function tests), the subject will be randomized. The site pharmacist or study coordinator will contact the IVRS for assignment of the study therapy to be administered. Sites should not call the IVRS for study therapy administration until the subject has met all criteria for the study and is ready to receive the first dose of study therapy on Day 1.

The first dose of study therapy will be administered at the Day 1 Visit. The oral or IV formulation of MK-8228 or placebo will be dispensed via the IVRS. Subjects will be initiated with the oral (tablet) formulation of study therapy provided they are able to swallow and do not have a condition (e.g., vomiting, diarrhea, or a malabsorptive condition) that may interfere with the absorption of the tablets. For subjects who cannot swallow and/or have a condition that may interfere with the absorption of the oral formulation, study therapy can be initiated with or switched to the IV formulation. The IV formulation should be switched to oral study therapy (i.e., at the next planned dose) as soon as such subjects are able to swallow and/or the condition necessitating the use of the IV formulation resolves. Use of the IV formulation should generally be limited to 4 weeks or less in duration. However, it will be left to the investigator’s discretion to continue IV administration beyond 4 weeks, if the benefit/risk ratio supports continued administration.

The same dose of MK-8228 will be administered for both formulations. Subjects in the MK-8228 treatment group will receive either 240 mg MK-8228 qd, if receiving concomitant CsA, or 480 mg MK-8228 qd, if not on CsA. If CsA is initiated after starting study therapy, the next dose of MK-8228 (administered up to 24 hours later) should be decreased to 240 mg qd. If CsA is discontinued in a subject, the next dose of MK-8228 (administered up to 24 hours later) should be increased from 240 mg to 480 mg qd. Corresponding changes in tablets for oral formulation with changes in CsA dosing will also occur in the placebo group in an effort to maintain the study blind (see Section 5.2.3).

After Day 1, study therapy will continue through Week 14 (~100 days) post-transplant. During this period, samples for CMV DNA PCR should be sent **at every visit** to the **central laboratory** as per the Trial Flow Chart (Section 6.0).

The subject will be trained in the use of the electronic Study Medication Diary. Once the subject is discharged from the hospital, he/she will be instructed to enter the number of tablets of study therapy taken during the study therapy period.

7.1.5.3 Follow-up Period

After the last day of study therapy, subjects will continue to be followed though Week 24 (~6 months) post-transplant. Visits will occur every 2 weeks between Week 14 post-transplant and Week 24 post-transplant, and all procedures listed in the Trial Flow Chart (Section 6.0) corresponding to the visits will be performed.

Following the primary study period through Week 24 (~6 months) post-transplant, subjects will remain in the study through Week 48 post-transplant in order to continue collecting information on CMV disease, health outcomes, and quality of life. Visits will occur at Weeks 32, 40, and 48 post-transplant and all procedures listed in the Trial Flow Chart (Section 6.0) corresponding to the visits will be performed.

During the follow-up period, samples for CMV DNA PCR should be sent **at every visit** to the **central laboratory** as per the Trial Flow Chart (Section 6.0).

Adverse event monitoring should include the collection of all adverse events while on study therapy and for 2 weeks following completion of study therapy (i.e., through Follow-up Week 2 Visit) in all subjects, including those who have discontinued study therapy but are continuing in the study. Thereafter, only drug related SAEs and SAEs leading to death will be collected through Week 48 post-transplant (i.e., through Follow-up Week 34 Visit).

7.1.5.4 CMV Infection or Early Discontinuation Visit

The CMV Infection Visit will be performed for all subjects who will be discontinued from study therapy due to clinically significant CMV infection requiring either treatment of disease or initiation of PET. It is very important to ensure that all procedures, as outlined in the Trial Flow Chart (Section 6.0), are performed at the CMV Infection Visit **immediately prior to** the initiation of treatment of CMV diseases or initiation of PET (i.e., on the day anti-CMV therapy is initiated). Most importantly, a plasma sample for CMV PCR testing at the central laboratory should be collected at this visit.

After this visit, such subjects will continue to be followed in the study and complete all remaining visits through Week 48 post-transplant as outlined in the Trial Flow Chart (Section 6.0). All specified procedures during the study therapy period will be completed for these subjects with the exception of study therapy administration, PK assessments, and study medication diary review.

The CMV Infection Visit will also be performed for all subjects who require either treatment for disease or initiation of PET after study therapy completion, during the follow-up period (after Week 14 through Week 24 post-transplant). It is very important to ensure that all procedures, as outlined in the Trial Flow Chart (Section 6.0), are performed at the CMV

Infection Visit **<u>immediately prior to</u>** the initiation of treatment of CMV diseases or initiation of PET (i.e., on the day anti-CMV therapy is initiated). Most importantly, a plasma sample for CMV PCR testing at the central laboratory should be collected at this visit.

After this visit, such subjects will continue to be followed in the study and complete all remaining visits through Week 48 post-transplant as outlined in the Trial Flow Chart (Section 6.0).

<u>Note</u>: It is mandatory to send a plasma sample for CMV DNA PCR testing to the central laboratory **<u>immediately prior to</u>** (i.e., on the day of) initiating treatment for CMV disease or PET in **<u>ALL</u>** instances.

In the event confirmatory test results from the central laboratory are not available within the timeframe the investigator wishes to initiate anti-CMV therapy, the investigator may use a positive local laboratory test result (from CMV DNA PCR or pp65 antigen only) to make the decision. When local laboratory test results are used for initiating anti-CMV therapy, two plasma samples for CMV DNA PCR testing must be sent to the <u>central laboratory</u>. The first sample must be collected immediately prior to (i.e., on the day of) initiating anti-CMV therapy. The second sample must be collected 48-72 hours after initiating anti-CMV therapy. The local laboratory result must also be reported in such instances.

The Early Discontinuation Visit will be performed for all subjects who are prematurely discontinued up to Week 24 post-transplant from the *<u>study, not study therapy</u>*. It is very important to ensure that all procedures, as outlined in the Trial Flow Chart (Section 6.0), are performed in such subjects at this visit prior to discontinuing the subject from the trial. Most importantly, a plasma sample for CMV PCR testing at the central laboratory should be collected at this visit.

7.2 Assessing and Recording Adverse Events

An adverse event is defined as any untoward medical occurrence in a patient or clinical investigation subject administered a pharmaceutical product and which does not necessarily have to have a causal relationship with this treatment. An adverse event can therefore be any unfavourable and unintended sign (including an abnormal laboratory finding, for example), symptom, or disease temporally associated with the use of a medicinal product or protocol-specified procedure, whether or not considered related to the medicinal product or protocol-specified procedure. Any worsening (i.e., any clinically significant adverse change in frequency and/or intensity) of a preexisting condition that is temporally associated with the use of the Sponsor’s product, is also an adverse event.

Changes resulting from normal growth and development that do not vary significantly in frequency or severity from expected levels are not to be considered adverse events. Examples of this may include, but are not limited to, teething, typical crying in infants and children and onset of menses or menopause occurring at a physiologically appropriate time.

Sponsor's product includes any pharmaceutical product, biological product, device, diagnostic agent or protocol-specified procedure, whether investigational (including placebo or active comparator medication) or marketed, manufactured by, licensed by, provided by or distributed by the Sponsor for human use.

Adverse events may occur during the course of the use of the Sponsor's product in clinical trials or within the follow-up period specified by the protocol, or prescribed in clinical practice, from overdose (whether accidental or intentional), from abuse and from withdrawal.

Adverse events may also occur in screened subjects during any pre-allocation baseline period as a result of a protocol-specified intervention, including washout or discontinuation of usual therapy, diet, placebo treatment or a procedure.

All adverse events will be recorded from the time the consent form is signed through 2 weeks following the end of the treatment period (Week 16, Follow-up Week 2) for all subjects including those who have discontinued study therapy and at each examination on the Adverse Event case report forms/worksheets.

7.2.1 Definition of an Overdose for This Protocol and Reporting of Overdose to the Sponsor

In this trial, an overdose is any dose higher than two times the dose specified in Section 5.2 (Trial Treatments).

If an adverse event(s) is associated with ("results from") the overdose of Sponsor's product or vaccine, the adverse event(s) is reported as a serious adverse event, even if no other seriousness criteria are met.

If a dose of Sponsor's product or vaccine meeting the protocol definition of overdose is taken without any associated clinical symptoms or abnormal laboratory results, the overdose is reported as a non-serious Event of Clinical Interest (ECI), using the terminology "accidental or intentional overdose without adverse effect."

All reports of overdose with and without an adverse event must be reported within 24 hours to the Sponsor either by electronic media or paper. Sponsor Contact information can be found in the Investigator Trial File Binder.

7.2.2 Reporting of Pregnancy and Lactation to the Sponsor

Although pregnancy and lactation are not considered adverse events, it is the responsibility of investigators or their designees to report any pregnancy or lactation in a subject (spontaneously reported to them) that occurs during the trial or within 14 days of completing the trial. All subjects who become pregnant must be followed to the completion/termination of the pregnancy. Pregnancy outcomes of spontaneous abortion, missed abortion, benign hydatidiform mole, blighted ovum, fetal death, intrauterine death, miscarriage and stillbirth must be reported as serious events (Important Medical Events). If the pregnancy continues to term, the outcome (health of infant) must also be reported.

Such events must be reported within 24 hours to the Sponsor either by electronic media or paper. Sponsor Contact information can be found in the Investigator Trial File Binder.

7.2.3 Immediate Reporting of Adverse Events to the Sponsor

7.2.3.1 Serious Adverse Events

A serious adverse event is any adverse event occurring at any dose or during any use of Sponsor's product that:

- Results in death;
- Is life threatening;
- Results in persistent or significant disability/incapacity;
- Results in or prolongs an existing inpatient hospitalization;
- Is a congenital anomaly/birth defect;
- Is a cancer;
- Is associated with an overdose;
- Is an other important medical event

Refer to Table 5 for additional details regarding each of the above criteria.

Any serious adverse event, or follow up to a serious adverse event, including death due to any cause that occurs to any subject from the time the consent is signed through 2 weeks following the end of the treatment period (Week 16, Follow-up Week 2) for all subjects including those who have discontinued study therapy, whether or not related to the Sponsor's product, must be reported within 24 hours to the Sponsor either by electronic media or paper. Sponsor Contact information can be found in the Investigator Trial File Binder.

Additionally, any serious adverse event, considered by an investigator who is a qualified physician to be related to the Sponsor's product or death due to any cause that is brought to the attention of the investigator at any time outside of the time period specified in the previous paragraph also must be reported immediately to the Sponsor.

All subjects with serious adverse events must be followed up for outcome.

7.2.3.2 Events of Clinical Interest

Selected non-serious and serious adverse events are also known as Events of Clinical Interest (ECI) and must be recorded as such on the Adverse Event case report forms/worksheets and reported within 24 hours to the Sponsor either by electronic media or paper. Sponsor Contact information can be found in the Investigator Trial File Binder.

Events of clinical interest for this trial include:

- an overdose of Sponsor's product, as defined in Section 7.2.1 - Definition of an Overdose for This Protocol and Reporting of Overdose to the Sponsor, that is not associated with clinical symptoms or abnormal laboratory results.

- an elevated AST or ALT lab value that is greater than or equal to 3X the upper limit of normal and an elevated total bilirubin lab value that is greater than or equal to 2X the upper limit of normal and, at the same time, an alkaline phosphatase lab value that is less than 2X the upper limit of normal, as determined by way of protocol-specified laboratory testing or unscheduled laboratory testing.*

*Note: These criteria are based upon available regulatory guidance documents. The purpose of the criteria is to specify a threshold of abnormal hepatic tests that may require an additional evaluation for an underlying etiology. The trial site guidance for assessment and follow up of these criteria can be found in the Investigator Trial File Binder.

7.2.3.3 Protocol-Specific Exceptions to Serious Adverse Event Reporting

7.2.4 Evaluating Adverse Events

An investigator who is a qualified physician will evaluate all adverse events with respect to the elements outlined in Table 5 The investigator's assessment of causality is required for each adverse event. Refer to Table 5 for instructions in evaluating adverse events.

Table 5 Evaluating Adverse Events

<table>
<tr><td rowspan="3">Maximum Intensity</td><td>Mild</td><td>awareness of sign or symptom, but easily tolerated (for pediatric trials, awareness of symptom, but easily tolerated)</td></tr>
<tr><td>Moderate</td><td>discomfort enough to cause interference with usual activity (for pediatric trials, definitely acting like something is wrong)</td></tr>
<tr><td>Severe</td><td>incapacitating with inability to work or do usual activity (for pediatric trials, extremely distressed or unable to do usual activities)</td></tr>
<tr><td rowspan="10">Seriousness</td><td colspan="2">A serious adverse event (AE) is any adverse event occurring at any dose or during any use of Sponsor's product that:</td></tr>
<tr><td colspan="2">†Results in death; or</td></tr>
<tr><td colspan="2">†Is life threatening; or places the subject, in the view of the investigator, at immediate risk of death from the event as it occurred [Note: This does not include an adverse event that, had it occurred in a more severe form, might have caused death.]; or</td></tr>
<tr><td colspan="2">†Results in a persistent or significant disability/incapacity (substantial disruption of one's ability to conduct normal life functions); or</td></tr>
<tr><td colspan="2">†Results in or prolongs an existing inpatient hospitalization (hospitalization is defined as an inpatient admission, regardless of length of stay, even if the hospitalization is a precautionary measure for continued observation. (Note: Hospitalization [including hospitalization for an elective procedure] for a preexisting condition which has not worsened does not constitute a serious adverse event.); or</td></tr>
<tr><td colspan="2">†Is a congenital anomaly/birth defect (in offspring of subject taking the product regardless of time to diagnosis); or</td></tr>
<tr><td colspan="2">Is a cancer; or</td></tr>
<tr><td colspan="2">Is associated with an overdose (whether accidental or intentional). Any adverse event associated with an overdose is considered a serious adverse event. An overdose that is not associated with an adverse event is considered a non-serious event of clinical interest and must be reported within 24 hours.</td></tr>
<tr><td colspan="2">Other important medical events that may not result in death, not be life threatening, or not require hospitalization may be considered a serious adverse event when, based upon appropriate medical judgment, the event may jeopardize the subject and may require medical or surgical intervention to prevent one of the outcomes listed previously (designated above by a †).</td></tr>
<tr style="display:none"><td colspan="2"></td></tr>
<tr><td>Duration</td><td colspan="2">Record the start and stop dates of the adverse event. If less than 1 day, indicate the appropriate length of time and units</td></tr>
<tr><td>Action taken</td><td colspan="2">Did the adverse event cause the Sponsor's product to be discontinued?</td></tr>
<tr><td rowspan="4">Relationship to Sponsor's Product</td><td colspan="2">Did the Sponsor's product cause the adverse event? The determination of the likelihood that the Sponsor's product caused the adverse event will be provided by an investigator who is a qualified physician. The investigator's signed/dated initials on the source document or worksheet that supports the causality noted on the AE form, ensures that a medically qualified assessment of causality was done. This initialed document must be retained for the required regulatory time frame. The criteria below are intended as reference guidelines to assist the investigator in assessing the likelihood of a relationship between the test drug and the adverse event based upon the available information.
The following components are to be used to assess the relationship between the Sponsor's product and the AE; the greater the correlation with the components and their respective elements (in number and/or intensity), the more likely the Sponsor's product caused the adverse event:</td></tr>
<tr><td>Exposure</td><td>Is there evidence that the subject was actually exposed to the Sponsor's product such as: reliable history, acceptable compliance assessment (pill count, diary, etc.), expected pharmacologic effect, or measurement of drug/metabolite in bodily specimen?</td></tr>
<tr><td>Time Course</td><td>Did the AE follow in a reasonable temporal sequence from administration of the Sponsor's product?
Is the time of onset of the AE compatible with a drug-induced effect (applies to trials with investigational medicinal product)?</td></tr>
<tr><td>Likely Cause</td><td>Is the AE not reasonably explained by another etiology such as underlying disease, other drug(s)/vaccine(s), or other host or environmental factors</td></tr>
</table>

<table>
<tr><td rowspan="3">Relationship to Sponsor's Product (continued)</td><td colspan="2">The following components are to be used to assess the relationship between the Sponsor's product and the AE: (continued)</td></tr>
<tr><td>Dechallenge</td><td>Was the Sponsor's product discontinued or dose/exposure/frequency reduced?
If yes, did the AE resolve or improve?
If yes, this is a positive dechallenge. If no, this is a negative dechallenge.
(Note: This criterion is not applicable if: (1) the AE resulted in death or permanent disability; (2) the AE resolved/improved despite continuation of the Sponsor's product; (3) the trial is a single-dose drug trial); or (4) Sponsor's product(s) is/are only used one time.)</td></tr>
<tr><td>Rechallenge</td><td>Was the subject re-exposed to the Sponsor's product in this trial?
If yes, did the AE recur or worsen?
If yes, this is a positive rechallenge. If no, this is a negative rechallenge.
(Note: This criterion is not applicable if: (1) the initial AE resulted in death or permanent disability, or (2) the trial is a single-dose drug trial); or (3) Sponsor's product(s) is/are used only one time.)
NOTE: IF A RECHALLENGE IS PLANNED FOR AN ADVERSE EVENT WHICH WAS SERIOUS AND WHICH MAY HAVE BEEN CAUSED BY THE SPONSOR'S PRODUCT, OR IF RE-EXPOSURE TO THE SPONSOR'S PRODUCT POSES ADDITIONAL POTENTIAL SIGNIFICANT RISK TO THE SUBJECT THEN THE RECHALLENGE MUST BE APPROVED IN ADVANCE BY THE U.S. CLINICAL MONITOR AND THE INSTITUTIONAL REVIEW BOARD/INDEPENDENT ETHICS COMMITTEE.</td></tr>
<tr><td></td><td>Consistency with Trial Treatment Profile</td><td>Is the clinical/pathological presentation of the AE consistent with previous knowledge regarding the Sponsor's product or drug class pharmacology or toxicology?</td></tr>
<tr><td colspan="3">The assessment of relationship will be reported on the case report forms /worksheets by an investigator who is a qualified physician according to his/her best clinical judgment, including consideration of the above elements.</td></tr>
<tr><td colspan="2">Record one of the following:</td><td>Use the following scale of criteria as guidance (not all criteria must be present to be indicative of a Sponsor's product relationship).</td></tr>
<tr><td colspan="2">Yes, there is a reasonable possibility of Sponsor's product relationship.</td><td>There is evidence of exposure to the Sponsor's product. The temporal sequence of the AE onset relative to the administration of the Sponsor's product is reasonable. The AE is more likely explained by the Sponsor's product than by another cause.</td></tr>
<tr><td colspan="2">No, there is not a reasonable possibility of Sponsor's product relationship</td><td>Subject did not receive the Sponsor's product OR temporal sequence of the AE onset relative to administration of the Sponsor's product is not reasonable OR there is another obvious cause of the AE. (Also entered for a subject with overdose without an associated AE.)</td></tr>
</table>

7.2.5 Sponsor Responsibility for Reporting Adverse Events

All Adverse Events will be reported to regulatory authorities, IRB/IECs and investigators in accordance with all applicable global laws and regulations.

7.3 TRIAL GOVERNANCE AND OVERSIGHT

7.3.1 Scientific Advisory Committee

This trial was developed in collaboration with a Scientific Advisory Committee (SAC). The SAC comprises both Sponsor and non-Sponsor scientific experts who provide input with respect to trial design, interpretation of trial results and subsequent peer-reviewed scientific publications.

7.3.2 Executive Oversight Committee

The Executive Oversight Committee (EOC) comprises members of Sponsor Senior Management. The EOC will receive and decide upon any recommendations made by the Data Monitoring Committee (DMC) regarding the trial.

7.3.3 Data Monitoring Committee

To supplement the routine trial monitoring outlined in this protocol, an external Data Monitoring Committee (DMC) will monitor the interim data from this trial. The voting members of the committee are external to the Sponsor. The members of the DMC must not be involved with the trial in any other way (e.g., they cannot be trial investigators) and must have no competing interests that could affect their roles with respect to the trial. The DMC will include 4 clinicians experienced in Infectious Diseases and 1 external statistician; this is in addition to the unblinded trial statistician who will be a non-voting member of the committee.

The DMC will make recommendations to the EOC regarding steps to ensure both subject safety and the continued ethical integrity of the trial. Also, the DMC will review interim trial results, consider the overall risk and benefit to trial participants (see Section 8.1.4 - Interim Analyses) and recommend to the EOC if the trial should continue in accordance with the protocol.

Specific details regarding responsibilities and governance, including the roles and responsibilities of the various members and the Sponsor protocol team; meeting facilitation; the trial governance structure; and requirements for and proper documentation of DMC reports, minutes, and recommendations will be described in a separate charter that is reviewed and approved by the DMC. The DMC will monitor the trial at an appropriate frequency, as described in the detailed DMC charter. The DMC will also make recommendations to the Sponsor protocol team regarding steps to ensure both subject safety and the continued ethical integrity of the trial.

7.3.4 Clinical Adjudication Committee

A Clinical Adjudication Committee (CAC) will evaluate the following events for the purposes of confirming them according to the criteria in Section 8.0 – Statistical Analysis Plan, as well as evaluating the presence of confounding factors.

1) CMV disease, as defined in Appendix 12.4.: This role is important to standardize the evaluation of all suspected cases of CMV disease occurring during the trial.

All personnel involved in the adjudication process will remain blinded to treatment allocation throughout the trial. Specific details regarding endpoint definitions can be found in the Adjudication Charter.

8.0 STATISTICAL ANALYSIS PLAN

8.1 Statistical Analysis Plan Summary

This section contains a brief summary of the statistical analyses for this trial. Full detail is in the Statistical Analysis Plan (SAP) (Section 8.2).

8.1.1 Efficacy Analyses

The primary and key secondary endpoints, primary analysis population, and statistical methods that will be employed for the efficacy analyses are presented in Table 6 below.

The FAS population consists of all randomized subjects who have received at least one dose of study medication and had no detectable CMV viral DNA (measured by central laboratory) on Day 1 (when study therapy is initiated).

The primary hypothesis will be evaluated by comparing MK-8228 to placebo in the proportion of subjects with clinically significant CMV infection (as defined in Sections 2.1 and 4.2.3.1) through Week 24 post-transplant in the FAS population. Other efficacy analyses will be considered supportive and/or explanatory.

Table 6 Summary of Analysis Strategy for Key Efficacy Endpoints

Endpoint/Variable (Description, Timepoint)	Statistical Method	Analysis Population	Missing Data Approach
Primary:			
Proportion of subjects with clinically significant CMV infection through Week 24 (~6 months) post-transplant	Stratified Mantel-Haenszel	Full Analysis set	Non-Completer = Failure*
Key Secondary:			
Proportion of subjects with clinically significant CMV infection through Week 14 (~100 days) post-transplant	Stratified Mantel-Haenszel	Full Analysis set	Non-Completer = Failure*
Time to onset of clinically significant CMV infection through Week 24 (~6 months) post-transplant	Kaplan-Meier plot	Full Analysis set	N/A
* Non-completers refer to subjects who prematurely discontinued from the study (See Section 8.2.5.1.4 for details). N/A = not applicable.			

8.1.2 Safety Analyses

All AEs will be collected through 14 days after completion of treatment period (through Follow-up Week 2 Visit). Thereafter, any SAEs related to study medication or SAEs leading to death will be collected through Week 48 post-transplant. Safety and tolerability will be assessed by statistical and clinical review of all safety data collected throughout the study. The All-Subjects-as-Treated population will be employed for safety analyses.

Statistical analyses of adverse events will follow the 3-tiered analysis approach. For this study, there is no pre-specified Tier 1 event that will be formally compared using inferential statistics. Tier 2 events include AEs that occur in ≥ 4 subjects in any one treatment group and also include (1) at least one adverse event; (2) a drug-related adverse event; (3) a serious adverse event; (4) a serious and drug-related adverse event and (5) an adverse event leading to discontinuation. Tier 2 events will be assessed via point estimates with 95% confidence intervals provided for between-group comparisons. Descriptive safety endpoints (Tier 3) include all other safety parameters not analyzed as a Tier 2 safety endpoint. These Tier 3 safety endpoints include AEs, laboratory assessments, vital signs, and ECGs. Only point estimates by treatment group are provided for Tier 3 safety parameters.

8.1.3 Power and Sample Size

Based on literature data for placebo and on the results from the Phase II study AIC246-01-II-02, the incidence rate of clinically significant CMV infection for subjects receiving placebo is expected to be approximately 35%. It is expected that the MK-8228 arm will reduce this incidence by half to an incidence of approximately 17%. It is further anticipated that about 20% of subjects will be discontinued from the trial for both treatment arms for reasons other than virologic failure. Since the primary missing data approach will be Non-Completer = Failure approach, 20% was added to the expected incidence of clinically significant CMV infection for the placebo arm (55%) and the MK-8228 arm (37%) for sample size and power calculations.

A sample size of approximately 540 subjects is planned using a 2:1 randomization ratio (~360 subjects in the MK-8228 arm and ~180 subjects in the placebo arm). Excluding 15% subjects with detectable CMV DNA on Day 1, the evaluable number of subjects in the FAS population is 459 in total (306 in the MK-8228 arm and 153 in the placebo arm). A futility interim analysis (IA) will be conducted when approximately 40% of the subjects have completed treatment or discontinued prior to completing treatment. With the current sample size, this study will have 90.5% overall power. If the study were designed without the futility analysis, the power would be 95%.

Although there is no intention for stopping for overwhelming efficacy at the interim time, a small amount of alpha ($\alpha = 0.0001$) will be allocated for statistical rigor. At the end of the trial, a 1-sided p-value that is less than or equal to 0.0249 will be used for declaring efficacy success.

8.1.4 Interim Analysis

A futility analysis will be conducted when 40% randomized subjects either complete study therapy through Week 14 (~100 days) post-transplant or discontinue prior to that time point. Results will be reviewed by the DMC. The endpoint, timing, and purpose of the interim analyses are summarized in the table below. The decision rule and other statistical details are further described in Section 8.2.

In addition, periodic safety reviews will be conducted. The first safety review will occur when 10% of randomized subjects either complete study therapy through Week 14 (~100 days) post-transplant or discontinue prior to that time point. Thereafter, the DMC will review all safety data approximately every 6 months during the trial (or at longer time intervals in case of slow enrollment).

8.2 Statistical Analysis Plan

This section outlines the statistical analysis strategy and procedures for the study. If, after the study has begun, changes are made to primary and/or key secondary objectives, or the statistical methods related to those objectives, then the protocol will be amended (consistent with ICH Guideline E-9). Changes to exploratory or other non-confirmatory analyses made after the protocol has been finalized, along with an explanation as to when and why they occurred, will be listed in the Clinical Study Report (CSR) for the study. Post hoc exploratory analyses will be clearly identified in the CSR. No separate Statistical Analysis Plan (SAP) will be issued for this study.

8.2.1 Responsibility for Analyses/ In-House Blinding

The Clinical Biostatistics department or designee will generate the randomized allocation schedule(s) for study treatment assignment. Randomization will be implemented in an interactive voice/web response system (IVRS/IWRS). The statistical analysis of the data obtained from this study will be the responsibility of the Clinical Biostatistics department of the SPONSOR. Certain specific analyses such as those for pharmacokinetic (PK), pharmacogenetics, and quality of life measures will be the responsibility of the appropriate departments of the SPONSOR.

This study has a primary study period (from Day 1 through Week 24 post-transplant), followed by an extension period to Week 48 post-transplant in order to collect CMV disease, health outcomes and quality of life data. The primary study period will be conducted as a double-blind study under in-house blinding procedures. The official, final database for the primary study period will not be unblinded until medical/scientific review has been performed, protocol violators have been identified, and data have been declared final and complete. The CSR will be finalized after results from the primary study period (through Week 24 post-transplant) are complete; all available post Week 24 data pertaining to mortality and CMV disease will be provided. Results of the extension period (through Week 48 post-transplant) will be presented in a separate report.

The Clinical Biostatistics department or designee will generate the randomized allocation schedule(s) for study treatment assignment. Randomization will be implemented in an interactive voice/web response system (IVRS/IWRS).

Planned interim analyses are described in Section 8.1.4. Study enrollment is likely to be ongoing at the time of any interim analyses. Blinding to treatment assignment will be maintained at all investigational sites. The results of interim analyses will not be shared with the investigators prior to the completion of the study. Subject-level unblinding will be restricted to an external unblinded group of statisticians. Treatment-level results of the interim analysis will be provided by the external statisticians to the DMC. Limited additional SPONSOR personnel may be unblinded to the treatment level results of the interim analysis (analyses), if required, in order to act on the recommendations of the DMC. The extent to which individuals are unblinded with respect to results of interim analyses will be documented by the external statisticians.

Prior to final study unblinding, the external statisticians will not be involved in any discussions regarding modifications to the protocol, statistical methods, identification of protocol violators, or data validation efforts.

Pharmacokinetic measurements will be conducted in support of exposure-response evaluations. Additionally, a small team as specified in a separate Modeling and Simulation (M&S) Modeling Analysis Plan, and who are separate from the study team, will be unblinded for the purpose of preparing the pharmacokinetic analyses. No interim data or results will be shared with the study team, and the unblinded group will not be members of the study team. No alpha adjustment will be made for this administrative look.

8.2.2 Hypotheses/Estimation

Objectives and hypotheses of the study are stated in Section 3.0.

8.2.3 Analysis Endpoints

Efficacy and safety endpoints that will be evaluated are listed in the following sections.

8.2.3.1 Efficacy/Pharmacokinetic Endpoints

8.2.3.1.1 Efficacy Endpoints

An initial description of efficacy measures is provided in Section 4.2.3.1.

The primary efficacy endpoint will be the proportion of subjects with clinically significant CMV infection through Week 24 (~6 months) post-transplant, defined as the occurrence of either one of the following outcomes:

- onset of CMV end-organ disease

  OR

- initiation of anti-CMV PET based on documented CMV viremia (as measured by the central laboratory) and the clinical condition of the subject.

CMV end-organ disease will be determined using the definitions in Appendix 12.4 and confirmed by an independent, blinded Clinical Adjudication Committee (CAC). The adjudication of cases by the CAC (i.e., the final CAC assessment) will take precedence over the investigator's assessment. Only the CAC-confirmed cases of CMV end-organ disease will be included in the CMV end-organ disease category. However, investigator-assessed CMV end-organ disease cases which were not confirmed by the CAC but in whom anti-CMV therapy was initiated (in the setting of documented CMV viremia at a central laboratory) will be included in the initiation of PET category and, therefore, qualify as having clinically significant CMV infection. Concordance/discordance between CAC and investigator assessment will be summarized.

Documented viremia is defined as any detectable CMV viral DNA measured by the Roche COBAS® AmpliPrep/COBAS TaqMan® (CAP/CTM) System in the central laboratory at the time of initiation of anti-CMV therapy plus or minus a 7-day window period. Initiation of anti-CMV therapy without documented CMV viremia (using the central laboratory) will not be considered as a case for clinically significant CMV infection. Similarly, detectable CMV viral DNA alone without initiation of anti-CMV therapy will not be considered as a case for clinically significant CMV infection.

The secondary efficacy endpoints are:

1) Proportion of subjects with clinically significant CMV infection through Week 14 (~100 days) post-transplant.

For this endpoint, case counting will use the same definition as in the primary efficacy endpoint.

2) Time to onset of clinically significant CMV infection through Week 24 (~6 months) post-transplant.

The time to onset of clinically significant CMV infection will be calculated in days, from the day of randomization to the day of onset of CMV end-organ disease or to the day of initiation of anti-CMV PET. For cases where CMV end-organ disease is confirmed by the CAC, the earliest clinical manifestation (sign/symptom) related to the CMV organ involvement will be identified by the CAC as part of their medical review and used as the time of onset of CMV

end organ disease. For cases where anti-CMV PET is initiated in the setting of documented viremia (including those applicable cases where CMV end-organ disease was not confirmed by the CAC), the start date of anti-CMV therapy will be used. If both criteria for clinically significant CMV infection are met, the time to onset will be calculated from the day of randomization to the earlier day on which one of the criteria is met.

3) Proportion of subjects with CMV disease through Week 14 post-transplant and Week 24 post-transplant.

For this endpoint, case counting will use the same definition for CMV end-organ disease as in the primary efficacy endpoint.

4) Proportion of subjects with initiation of PET for documented CMV viremia through Week 14 post-transplant and Week 24 post-transplant.

For this endpoint, case counting will use the same definition for initiation of PET for documented CMV viremia as in the primary efficacy endpoint.

5) The time to initiation of PET for documented CMV viremia through Week 24 post-transplant.

The time to initiation of PET for documented CMV viremia will be calculated in days, from the day of randomization to the start date of anti-CMV therapy including those applicable cases where CMV end-organ disease was not confirmed by the CAC.

8.2.3.1.2 Exploratory Endpoints

1 Proportion of subjects with CMV disease through Week 48 post-transplant.

2 Proportion of subjects with all-causes mortality through Week 14 post-transplant, Week 24 post-transplant, and Week 48 post-transplant.

3 Proportion of subjects with opportunistic infection other than CMV infection (i.e., systemic bacterial and invasive fungal infection) through Week 14 post-transplant, Week 24 post-transplant, and Week 48 post-transplant.

4 Proportion of subjects with acute and/or chronic graft-versus-host disease (GVHD) after randomization through Week 14 post-transplant, Week 24 post-transplant, and Week 48 post-transplant.

5 Proportion of subjects with all re-hospitalizations (following initial hospital discharge) and re-hospitalizations for CMV infection/disease through Week 14 post-transplant, Week 24 post-transplant, and Week 48 post-transplant.

6 Proportion of subjects with documented CMV viremia through Week 14 post-transplant, and Week 24 post-transplant.

7 The time to documented CMV viremia through Week 24 post-transplant.

8 Proportion of subjects with engraftment through Week 14 post-transplant and Week 24 post-transplant.

9 The time to onset of engraftment through Week 24 post-transplant.

10 Antiviral resistance to MK-8228 in prophylaxis failures.

11 Quality of life assessment (see Section 7.1.2.10 for details).

12 Pharmacokinetic Endpoints (see Section 4.2.3.3 for details).

8.2.3.2 Safety Endpoints

An initial description of safety measures is provided in Section 4.2.3.2.

All AEs will be collected through 14 days after completion of treatment period (through Follow-up Week 2 Visit). Thereafter, any SAEs related to study medication or SAEs leading to death will be collected through Week 48 post-transplant.

Safety endpoints will be analyzed using a 3-tiered approach (see Section 8.2.5.2).

8.2.4 Analysis Populations

8.2.4.1 Efficacy Analysis Populations

The Full Analysis Set (FAS) population will serve as the primary population for the analysis of efficacy data in this study. The FAS consists of all randomized subjects who receive at least one dose of study medication and had no detectable CMV viral DNA (measured by the central laboratory) on Day 1 (when study therapy is initiated).

A supportive analysis using the Per Protocol (PP) Set will be performed for the primary and key secondary efficacy endpoints. The PP population is a subset of the FAS population and it excludes subjects due to important deviations from the protocol that may substantially affect the results of the primary and key secondary efficacy endpoints. Potential violations that may result in the exclusion of a subject from the PP population include:

- Failure to reasonably adhere to the dosing schedule for the study medication
- Failure to comply with specific inclusion/exclusion criteria
- Use of a prohibited concomitant medication during the treatment period that may impact on the efficacy assessment

The final determination on protocol violations will be made prior to the final unblinding of the database and will be documented in a protocol violator memo.

Subjects will be included in the treatment group to which they are randomized for the analysis of efficacy data using both the FAS and PP populations.

8.2.4.2 Safety Analysis Populations

The All Subjects as Treated (ASaT) population will be used for the analysis of safety data in this study. The ASaT population consists of all randomized subjects who received at least one dose of study treatment. Subjects will be included in the treatment group corresponding to the study treatment they actually received for the analysis of safety data using the ASaT population. For most subjects this will be the treatment group to which they are randomized. Subjects who take incorrect study treatment for the entire treatment period will be included in the treatment group corresponding to the study treatment they actually received.

At least one laboratory or vital sign measurement obtained subsequent to at least one dose of study treatment is required for inclusion in the analysis of each specific parameter. To assess change from baseline, a baseline measurement is also required.

8.2.5 Statistical Methods

8.2.5.1 Statistical Methods for Efficacy Analyses

8.2.5.1.1 Primary Efficacy Analysis

To test the primary hypothesis that MK-8228 is superior to placebo in the prevention of clinically significant CMV infection, the stratum-adjusted Mantel-Haenszel method (with continuity correction) will be used to compare the proportion of subjects with clinically significant CMV infection through Week 24 (~6 months) post-transplant between the two treatment groups. Stratification factors such as high and low risk groups will be included in the primary efficacy analysis. Cochran Mantel-Haenszel weights will be used to calculate the overall between group differences across strata. MK-8228 is concluded superior to placebo if 1-sided p-value is less than or equal to 0.0249 (see Section 8.2.6 for alpha adjustment). Due to the expected large number of study centers, center will not be included as stratification factors in the primary efficacy analysis, but may be explored as a sensitivity analysis.

The primary efficacy analysis will be performed on the FAS population. The primary missing data approach will be the Non-Completer= Failure approach (See Section 8.2.5.1.4 for details). Supportive analyses using PP population and different missing data approaches will also be conducted (see Table 7).

8.2.5.1.2 Secondary Efficacy Analysis

To assess the first secondary endpoint, the proportion of subjects with clinically significant CMV infection through Week 14 (~100 days) post-transplant, the same stratum-adjusted Mantel-Haenszel method as described in the primary efficacy analysis will be performed. No formal hypothesis testing will be done on this endpoint.

Analysis on the second secondary endpoint- time to onset of clinically significant CMV infection through Week 24 (~6 months) post-transplant will be conducted using the non-parametric Kaplan-Meier method. The Kaplan-Meier curve will be plotted by treatment group and p-value for the between group difference in time to onset of clinically significant CMV infection will be provided using the log-rank test.

To assess the third secondary endpoint- proportion of subjects with CMV disease through Week 14 post-transplant and Week 24 post-transplant, stratum-adjusted Mantel-Haenszel method as described in the primary efficacy analysis will be performed. Only CAC confirmed CMV end-organ disease cases will be included in this analysis. Concordance/discordance between CAC and investigator assessment in CMV end-organ disease will be summarized.

To assess the fourth secondary endpoint- proportion of subjects with initiation of PET for documented viremia through Week 14 post-transplant and Week 24 post-transplant, stratum-adjusted Mantel-Haenszel method as described in the primary efficacy analysis will be performed. If there are cases where anti-CMV therapy is initiated with no detectable CMV DNA using the central laboratory data, a sensitivity analysis will be provided using the local laboratory results. In addition, another sensitivity analysis will be performed for initiation of PET based on viremia using the protocol recommended viral load threshold (see Section 4.2.3.1) instead of any detectable CMV DNA for documented viremia.

Analysis on the fifth secondary endpoint- time to initiation of PET for documented CMV viremia through Week 24 post-transplant will be conducted using the non-parametric Kaplan-Meier method. The Kaplan-Meier curve will be plotted by treatment group and p-value for the between group difference in time to onset of clinically significant CMV infection will be provided using the log-rank test.

Table 7 Analysis Strategy for Efficacy Variables

Endpoint/Variable (Description, Time Point)	Primary vs. Supportive Approach†	Statistical Method	Analysis Population	Missing Data Approach*
**Primary Endpoint**				
Proportion of subjects with clinically significant CMV infection through Week 24 (~6 months) post-transplant	P	Stratified M & H‡	FAS	NC=F
	S	Stratified M & H‡	PP	NC=F
	S	Stratified M & H‡	FAS	DAO
**Secondary Endpoints**				
Proportion of subjects with clinically significant CMV infection through Week 14 (~100 days) post-transplant	P	Stratified M & H‡	FAS	NC=F
	S	Stratified M & H‡	PP	NC=F
	S	Stratified M & H‡	FAS	DAO
Time to onset of clinically significant CMV infection through Week 24 (~6 months) post- transplant	P	Kaplan-Meier plot	FAS	N/A
Proportion of subjects with CMV disease through Week 14 post-transplant and Week 24 post-transplant	P	Stratified M & H‡	FAS	NC=F
	S	Stratified M & H‡	PP	NC=F
	S	Stratified M & H‡	FAS	DAO
Proportion of subjects with initiation of PET for documented CMV viremia through Week 14 post-transplant and Week 24 post-transplant.	P	Stratified M & H‡	FAS	NC=F
	S	Stratified M & H‡	PP	NC=F
	S	Stratified M & H‡	FAS	DAO
The time to initiation of PET for documented CMV viremia through Week 24 post-transplant.	P	Kaplan-Meier plot	FAS	N/A

* NC=F: Non-Completers equal failure. Non-completers refer to subjects who prematurely discontinued from the study. DAO = Data as observed, N/A = not applicable
† P=Primary approach; S=Secondary/supportive approach.
‡ Stratified Mantel-Haenszel method by high and low risk factors.

8.2.5.1.3 Exploratory Analysis

Summary statistics and 95% confidence intervals will be provided by treatment group for the following exploratory endpoints:

- proportion of subjects with CMV disease through Week 48 post-transplant
- proportion of subjects with all-cause mortality through Week 14 post-transplant, Week 24 post-transplant, and Week 48 post-transplant
- proportion of subjects with opportunistic infection (i.e., systemic bacterial infection or invasive fungal infection) through Week 14 post-transplant, Week 24 post-transplant, and Week 48 post-transplant
- proportion of subjects with GVHD through Week 14 post-transplant, Week 24 post-transplant, and Week 48 post-transplant
- proportion of subjects with all re-hospitalizations (following initial hospital discharge) and re-hospitalizations for CMV infection/disease through Week 14 post-transplant, Week 24 post-transplant, and Week 48 post-transplant.
- proportion of subjects with documented viremia (as measured by the central laboratory) through Week 14 post-transplant and Week 24 post-transplant.
- proportion of subjects with engraftment through Week 14 post-transplant and Week 24 post-transplant.

Kaplan-Meier plots will be provided for the time to documented viremia and the time to onset of engraftment through Week 24 post-transplant.

8.2.5.1.4 Missing Data Handling

The primary missing data approach will be the Non-Completer= Failure approach. Non-completers refer to subjects who prematurely discontinued from the study. A subject who discontinued study medication but remained in the study follow-up will not be considered as a non-completer.

A secondary missing data approach is the Data-As-Observed (DAO). With this approach, any subject with missing value for a particular endpoint will be excluded from the analysis.

8.2.5.2 Statistical Methods for Safety Analyses

Safety and tolerability will be assessed by clinical review of all relevant parameters including adverse experiences, laboratory tests, vital signs, and ECG measurements

The analysis of safety results will follow a 3-tiered approach. The tiers differ with respect to the analyses that will be performed. Safety parameters or adverse events that are identified a priori constitute “Tier 1” safety endpoints that will be subject to inferential testing for statistical significance with p-values and 95% confidence intervals provided for between-group comparisons. Other safety parameters will be considered "Tier 2" or "Tier 3". Tier 2 parameters (requires that at least 4 subjects in each treatment group exhibit the event) will be assessed via point estimates with 95% confidence intervals provided for between-group comparisons; only point estimates by treatment group are provided for Tier 3 safety parameters.

The threshold of at least 4 events was chosen because the 95% confidence interval for the between-group difference in percent incidence will always include zero when treatment groups of equal size each have less than 4 events and thus would add little to the interpretation of potentially meaningful differences. Because many 95% confidence intervals may be provided without adjustment for multiplicity, the confidence intervals should be regarded as a helpful descriptive measure to be used in review, not a formal method for assessing the statistical significance of the between-group differences in adverse experiences and predefined limits of change.

For this protocol, there are no Tier 1 events (see Table 8 below). In addition to the criteria described above, the following will also be analyzed as Tier 2 events: Proportion of subjects with: (1) at least one adverse event; (2) a drug-related adverse event; (3) a serious adverse event; (4) a serious and drug-related adverse event and (5) an adverse event leading to discontinuation.

Table 8 Analysis Strategy for Safety Parameters

Safety Tier	Safety Endpoint	p-Value	95% CI for Treatment Comparison	Descriptive Statistics
Tier 1	No tier 1 event defined in the current protocol	X	X	X
Tier 2	Any AE		X	X
	Any Drug-Related AE		X	X
	Any Serious AE		X	X
	Any Drug-Related Serious AE		X	X
	Discontinuation due to AE		X	X
	Any AE with incidence ≥ 4 subjects in one of the treatment group		X	X
Tier 3	Any AE with incidence < 4 subjects in both treatment groups			X
	Change from Baseline Results (laboratory, ECG, vital signs)			X

AE = adverse event; CI = confidence interval

95% confidence intervals will be based on the method of Miettenen and Nurminen [39]

Missing values will be handled using the Data-As-Observed (DAO) approach.

8.2.5.3 Summaries of Baseline Characteristics, Demographics, and Other Analyses

Demographic and Baseline Characteristics

The comparability of the treatment groups for each relevant characteristic will be assessed by the use of tables. No statistical hypothesis tests will be performed on these characteristics. The number and percentage of subjects screened, randomized, the primary reasons for screen failure, and the primary reason for discontinuation will be displayed. Demographic variables (e.g., age, gender, and high and low risk), indication for HSCT, prior and concomitant therapies will be summarized by treatment either by descriptive statistics or categorical tables.

PK/PD

Based on pharmacokinetic data obtained within this study, population PK and PK/PD analyses will be performed. The prospective details of this analysis will be specified in a separate modeling analysis plan.

Quality of Life Analysis

Standard algorithms will be used to compute total and subscale scores for the FACT-BMT and EQ-5D questionnaires as specified by the instrument developers. This trial is not powered to detect statistically significant differences in QOL scores between the treatment arms. Therefore, the analysis plan for the QOL instruments will be primarily descriptive in nature.

Questionnaire data will be analyzed using summary statistics at each administration time point by treatment arm. Actual subject information collected will be used with no imputations for missing data. Simple t-tests may be conducted post-hoc to calculate if there is a statistically significant difference in Quality of life scores at each time point of measurement between the treatment arms. Multivariate models may also be developed to understand subject risk factors that are significantly associated with patient quality of life scores.

8.2.6 Multiplicity

This study has one interim futility analysis, periodic interim safety reviews and one primary hypothesis testing at the end. Since stopping for futility and periodic safety checks do not inflate type I error rate, no alpha spending is planned for these analyses.

However, although there is no intention for stopping for overwhelming efficacy at the interim time, a small amount of alpha ($\alpha = 0.0001$) will be allocated for statistical rigor. At the end of the trial, a 1-sided p-value that is less than or equal to 0.0249 will be used for declaring efficacy success.

8.2.7 Sample Size and Power Calculations

8.2.7.1 Sample Size and Power for Efficacy Analysis

Based on literature data for placebo and on the results from the Phase II study AIC246-01-II-02, the incidence rate of clinically significant CMV infection for subjects receiving placebo is expected to be approximately 35%. It is expected that the MK-8228 arm will reduce this incidence by half to an incidence of approximately 17%. It is further anticipated that about 20% of subjects will be discontinued from trial for both treatment arms for reasons other than virologic failure. Since the primary missing data approach will be noncompleter = failure approach, 20% was added to the expected incidence of clinically significant CMV infection for the placebo arm (55%) and the MK-8228 arm (37%) for sample size and power calculations.

A sample size of approximately 540 subjects is planned using a 2:1 randomization ratio (~360 subjects in the MK-8228 arm and ~180 subjects in the placebo arm). Excluding 15% subjects with detectable CMV DNA on Day 1, the evaluable number of subjects in the FAS population is 459 in total (306 in the MK-8228 arm and 153 in the placebo arm). An Interim futility analysis will be conducted when approximately 40% of the subjects have completed treatment or discontinued prior to completing treatment. With the current sample size, this study will have 90.5% overall power. If the study were designed without the futility analysis, the power would be 95%.

8.2.7.2 Sample Size and Power for Safety Analysis

Table 9 gives the upper bound of the two-sided 95% Clopper-Pearson exact confidence interval for the true proportion of subjects with a particular adverse experience corresponding to various observed numbers of subjects with such adverse experience in a sample of 360 subjects and 180 subjects. For example, if a particular adverse experience is not observed in any of the 360 subjects in the MK-8228 arm, then we can conclude with 95% confidence that the true proportion is no more than 1.0%.

Table 9 Upper Bound of the Two-Sided 95% Confidence Interval for the True Proportion of Subjects with an AE

n	Observed Number of Subjects With AE (%)	95% Upper Bound for the True Proportion (%)
360	0 (0.0)	1.0
	12 (3.3)	5.7
	24 (6.7)	9.8
	36 (10.0)	13.6
180	0 (0.0)	2.0
	6 (3.3)	7.1
	12 (6.7))	11.4
	18 (10.0)	15.3

8.2.8 Subgroup Analyses and Effects of Baseline Factors

To determine whether the treatment effect is consistent across various subgroups, the estimate of the treatment effect (with a nominal 95% CI) for the primary efficacy endpoint will be estimated within each category of the following classification variables:

- Gender (Male, Female)
- Age (use median age cutoff)
- Race (white, black, Asian, other)
- Ethnicity (Hispanic, Not Hispanic)
- High and low risk

The consistency of the treatment effect will be assessed descriptively via summary statistics by category for the classification variables listed above. Other clinically relevant variables may be identified for which additional subgroup analyses may be performed.

8.2.9 Interim Analyses

An interim futility analysis will be conducted when 40% of randomized subjects either complete study therapy through Week 14 (~100 days) post-transplant or discontinue prior to that time point. The non-binding stopping boundary for the futility analysis will be based on the Hwang-Shih-DeCani spending function with gamma = 3.1. This trial may be stopped for futility at the IA if the 1-sided p-value for comparing MK-8228 to placebo is greater than 0.196. This p-value boundary corresponds to an approximate difference of 6.6 percentage points in the observed incidence of clinically significant CMV infection between the two groups. Under the null hypothesis of no treatment difference, the probability of correctly stopping the trial for futility is approximately 80%. Under the alternative hypothesis, the probability of incorrectly stopping the trial is approximately 7%.

In addition, periodic safety reviews will be conducted. The first safety review will occur when 10% of randomized subjects either complete study therapy through Week 14 (~100 days) post-transplant or discontinue prior to that time point. Thereafter, the DMC will review all safety data approximately every 6 months during the trial (or at longer time intervals in case of slow enrollment).

The interim analyses will be performed by an external independent statistician and the results will be shared with the DMC members. The DMC recommendations will be communicated to the Sponsor as specified in the DMC Charter.

Blinding to treatment assignment will be maintained at all investigational sites. The results of the interim analyses will not be shared with the investigators prior to the completion of the trial. The Executive Oversight Committee (EOC) of the sponsor will receive recommendations throughout the trial from the DMC and is responsible for acting upon the recommendations of the DMC. The EOC will not have access to unblinded data or reports unless it is deemed necessary by the DMC to have this information in order to be able to act upon a DMC recommendation. The EOC will be completely independent of, and separate from, the trial team performing the medical monitoring and supervising the operational aspects of the protocol.

The external unblinded statistician who will prepare the analyses will serve as a non-voting member of the DMC. This individual will not be involved in any discussions regarding modifications to the protocol, statistical methods, identification of protocol violations, or data validation efforts after the interim analyses.

8.2.10 Compliance/Medication Adherence

Drug accountability data for MK-8228 and the corresponding placebo will be collected during the study. A day within the study will be considered an “On-Therapy” day if the subject takes at least one dose. For a subject who is followed for the entire study period, the “Number of Days Should be on Therapy” is the total number of days from randomization to the last scheduled day for treatment administration for that subject. For a subject who discontinued from the study medication, the “Number of Days Should be on Therapy” is the total number of days from randomization to the date of the last dose of study medication.

For each subject, percent compliance will then be calculated using the following formula:

$$\text{Percent Compliance} = \frac{\text{Number of Days on Therapy}}{\text{Number of Days Should be on Therapy}} \text{ x } 100.$$

Compliance rates will be summarized for each treatment group and individual compliance rates will factor into the identification of protocol violators as discussed in Section 8.2.4.

In addition, percent of subjects on CsA and duration of CsA use will be reported.

8.2.11 Extent of Exposure

The extent of exposure to study treatment will be evaluated by summary statistics for the "Number of Days on Therapy" by treatment group.

9.0 LABELING, PACKAGING, STORAGE AND RETURN OF CLINICAL SUPPLIES

9.1 Investigational Product

The investigator shall take responsibility for and shall take all steps to maintain appropriate records and ensure appropriate supply, storage, handling, distribution and usage of investigational product in accordance with the protocol and any applicable laws and regulations.

Clinical Supplies will be provided by the Sponsor as summarized in Table 10.

Table 10 Product Descriptions

Product Name & Potency	Dosage Form
MK-8228 240 mg or Placebo	Tablet
MK-8228 240 mg	IV*
*Normal saline will be used as the placebo to IV MK-8228.	

9.2 Packaging and Labeling Information

Clinical supplies will be affixed with a clinical label in accordance with regulatory requirements.

For the tablet formulation, subjects will receive blinded bottles (each containing 1 week supply of study therapy). No kitting is required.

For the IV formulation, open-label vials of MK-8228 will be supplied. The IV formulation will be dispensed in a blinded fashion by an unblinded pharmacist (or qualified trial site personnel). Normal saline will be used as the placebo to IV MK-8228. The Sponsor will provide opaque covers for the IV bags and tubing to assist with blinding the study therapy. Because this is a double-blind study, the investigator, study personnel, and subject must remain blinded to the IV study therapy. In order to maintain the blinding, the unblinded pharmacist (or qualified study site personnel designated to prepare the IV supplies) will be

responsible solely for the preparation and administration of the IV study therapy. He/she will not be involved in evaluating subjects for efficacy or safety.

9.3 Clinical Supplies Disclosure

The central electronic randomization system (IVRS/IWRS) should be used in order to unblind subjects and to unmask drug identity. The Sponsor will not provide random code/disclosure envelopes or lists with the clinical supplies.

9.4 Storage and Handling Requirements

Clinical supplies must be stored in a secure, limited-access location under the storage conditions specified on the label.

Receipt and dispensing of trial medication must be recorded by an authorized person at the trial site.

Clinical supplies may not be used for any purpose other than that stated in the protocol.

9.5 Returns and Reconciliation

The investigator is responsible for keeping accurate records of the clinical supplies received from the Sponsor or designee, the amount dispensed to and returned by the subjects and the amount remaining at the conclusion of the trial.

For all trial sites, the local country Sponsor personnel or designee will provide appropriate documentation that must be completed for drug accountability and return.

9.6 Standard Policies

Trial site personnel will have access to a central electronic randomization system (IVRS/IWRS system) to allocate subjects, to assign drug to subjects and to manage the distribution of clinical supplies. Each person accessing the IVRS system must be assigned an individual unique PIN. They must use only their assigned PIN to access the system, and they must not share their assigned PIN with anyone.

10.0 ADMINISTRATIVE AND REGULATORY DETAILS

10.1 Confidentiality

10.1.1 Confidentiality of Data

By signing this protocol, the investigator affirms to the Sponsor that information furnished to the investigator by the Sponsor will be maintained in confidence, and such information will be divulged to the institutional review board, ethics review committee (IRB/ERC) or similar or expert committee; affiliated institution and employees, only under an appropriate understanding of confidentiality with such board or committee, affiliated institution and employees. Data generated by this trial will be considered confidential by the investigator, except to the extent that it is included in a publication as provided in the Publications section of this protocol.

10.1.2 Confidentiality of Subject Records

By signing this protocol, the investigator agrees that the Sponsor (or Sponsor representative), IRB/ERC, or regulatory authority representatives may consult and/or copy trial documents in order to verify worksheet/case report form data. By signing the consent form, the subject agrees to this process. If trial documents will be photocopied during the process of verifying worksheet/case report form information, the subject will be identified by unique code only; full names/initials will be masked prior to transmission to the Sponsor.

By signing this protocol, the investigator agrees to treat all subject data used and disclosed in connection with this trial in accordance with all applicable privacy laws, rules and regulations.

10.1.3 Confidentiality of Investigator Information

By signing this protocol, the investigator recognizes that certain personal identifying information with respect to the investigator, and all subinvestigators and trial site personnel, may be used and disclosed for trial management purposes, as part of a regulatory submissions, and as required by law. This information may include:

- name, address, telephone number and e-mail address;
- hospital or clinic address and telephone number;
- curriculum vitae or other summary of qualifications and credentials; and
- other professional documentation.

Consistent with the purposes described above, this information may be transmitted to the Sponsor, and subsidiaries, affiliates and agents of the Sponsor, in your country and other countries, including countries that do not have laws protecting such information. Additionally, the investigator's name and business contact information may be included when reporting certain serious adverse events to regulatory authorities or to other investigators. By signing this protocol, the investigator expressly consents to these uses and disclosures.

If this is a multicenter trial, in order to facilitate contact between investigators, the Sponsor may share an investigator's name and contact information with other participating investigators upon request.

10.1.4 Confidentiality of IRB/IEC Information

The Sponsor is required to record the name and address of each IRB/IEC member that reviews and approves this trial. The Sponsor is also required to document that each IRB/IEC meets regulatory and ICH GCP requirements by requesting and maintaining records of the names and qualifications of the IRB/IEC members and to make these records available for regulatory agency review upon request by those agencies.

10.2 Compliance with Financial Disclosure Requirements

Financial Disclosure requirements are outlined in the US Food and Drug Administration Regulations, Financial Disclosure by Clinical Investigators (21 CFR Part 54). It is the Sponsor's responsibility to determine, based on these regulations, whether a request for Financial Disclosure information is required. It is the investigator's/subinvestigator's responsibility to comply with any such request.

The investigator/subinvestigator(s) agree, if requested by the Sponsor in accordance with 21 CFR Part 54, to provide his/her financial interests in and/or arrangements with the Sponsor to allow for the submission of complete and accurate certification and disclosure statements. The investigator/subinvestigator(s) further agree to provide this information on a Certification/Disclosure Form, commonly known as a financial disclosure form, provided by the Sponsor or through a secure password-protected electronic portal provided by the Sponsor. The investigator/subinvestigator(s) also consent to the transmission of this information to the Sponsor in the United States for these purposes. This may involve the transmission of information to countries that do not have laws protecting personal data.

10.3 Compliance with Law, Audit and Debarment

By signing this protocol, the investigator agrees to conduct the trial in an efficient and diligent manner and in conformance with this protocol; generally accepted standards of Good Clinical Practice (e.g., International Conference on Harmonization of Technical Requirements for Registration of Pharmaceuticals for Human Use Good Clinical Practice: Consolidated Guideline and other generally accepted standards of good clinical practice); and all applicable federal, state and local laws, rules and regulations relating to the conduct of the clinical trial.

The Code of Conduct, a collection of goals and considerations that govern the ethical and scientific conduct of clinical investigations sponsored by Merck, is provided in Section 12.1 - Merck Code of Conduct for Clinical Trials.

The investigator also agrees to allow monitoring, audits, IRB/ERC review and regulatory authority inspection of trial-related documents and procedures and provide for direct access to all trial-related source data and documents.

The investigator agrees not to seek reimbursement from subjects, their insurance providers or from government programs for procedures included as part of the trial reimbursed to the investigator by the Sponsor.

The investigator shall prepare and maintain complete and accurate trial documentation in compliance with Good Clinical Practice standards and applicable federal, state and local laws, rules and regulations; and, for each subject participating in the trial, provide all data, and, upon completion or termination of the clinical trial, submit any other reports to the Sponsor as required by this protocol or as otherwise required pursuant to any agreement with the Sponsor.

Trial documentation will be promptly and fully disclosed to the Sponsor by the investigator upon request and also shall be made available at the trial site upon request for inspection, copying, review and audit at reasonable times by representatives of the Sponsor or any regulatory authorities. The investigator agrees to promptly take any reasonable steps that are

requested by the Sponsor as a result of an audit to cure deficiencies in the trial documentation and worksheets/case report forms.

The investigator must maintain copies of all documentation and records relating to the conduct of the trial in compliance with all applicable legal and regulatory requirements. This documentation includes, but is not limited to, the protocol, worksheets/case report forms, advertising for subject participation, adverse event reports, subject source data, correspondence with regulatory authorities and IRBs/ERCs, consent forms, investigator's curricula vitae, monitor visit logs, laboratory reference ranges, laboratory certification or quality control procedures and laboratory director curriculum vitae. By signing this protocol, the investigator agrees that documentation shall be retained until at least 2 years after the last approval of a marketing application in an ICH region or until there are no pending or contemplated marketing applications in an ICH region or until at least 2 years have elapsed since the formal discontinuation of clinical development of the investigational product. Because the clinical development and marketing application process is variable, it is anticipated that the retention period can be up to 15 years or longer after protocol database lock. The Sponsor will determine the minimum retention period and notify the investigator when documents may be destroyed. The sponsor also recognizes that documents may need to be retained for a longer period if required by local regulatory requirements. All trial documents shall be made available if required by relevant regulatory authorities. The investigator must consult with and obtain written approval by the Sponsor prior to discarding trial and/or subject files.

ICH Good Clinical Practice guidelines recommend that the investigator inform the subject's primary physician about the subject's participation in the trial if the subject has a primary physician and if the subject agrees to the primary physician being informed.

The investigator will promptly inform the Sponsor of any regulatory authority inspection conducted for this trial.

Persons debarred from conducting or working on clinical trials by any court or regulatory authority will not be allowed to conduct or work on this Sponsor's trials. The investigator will immediately disclose in writing to the Sponsor if any person who is involved in conducting the trial is debarred or if any proceeding for debarment is pending or, to the best of the investigator's knowledge, threatened.

In the event the Sponsor prematurely terminates a particular trial site, the Sponsor will promptly notify that trial site's IRB/IEC.

According to European legislation, a Sponsor must designate an overall coordinating investigator for a multi-center trial (including multinational). When more than one trial site is open in an EU country, Merck, as the Sponsor, will designate, per country, a national principal coordinator (Protocol CI), responsible for coordinating the work of the principal investigators at the different trial sites in that Member State, according to national regulations. For a single-center trial, the Protocol CI is the principal investigator. In addition, the Sponsor must designate a principal or coordinating investigator to review the trial report that summarizes the trial results and confirm that, to the best of his/her knowledge, the report accurately describes the conduct and results of the trial [Clinical Study Report (CSR) CI]. The Sponsor may consider one or more factors in the selection of the individual to serve as the Protocol CI and or CSR CI (e.g., availability of the CI during the

anticipated review process, thorough understanding of clinical trial methods, appropriate enrollment of subject cohort, timely achievement of trial milestones). The Protocol CI must be a participating trial investigator.

10.4 Compliance with Trial Registration and Results Posting Requirements

Under the terms of the Food and Drug Administration Modernization Act (FDAMA) and the Food and Drug Administration Amendments Act (FDAAA), the Sponsor of the trial is solely responsible for determining whether the trial and its results are subject to the requirements for submission to the Clinical Trials Data Bank, http://www.clinicaltrials.gov. Merck, as Sponsor of this trial, will review this protocol and submit the information necessary to fulfill these requirements. Merck entries are not limited to FDAMA/FDAAA mandated trials. Information posted will allow subjects to identify potentially appropriate trials for their disease conditions and pursue participation by calling a central contact number for further information on appropriate trial locations and trial site contact information.

By signing this protocol, the investigator acknowledges that the statutory obligations under FDAMA/FDAAA are that of the Sponsor and agrees not to submit any information about this trial or its results to the Clinical Trials Data Bank.

10.5 Quality Management System

By signing this protocol, the Sponsor agrees to be responsible for implementing and maintaining a quality management system with written development procedures and functional area standard operating procedures (SOPs) to ensure that trials are conducted and data are generated, documented, and reported in compliance with the protocol, accepted standards of Good Clinical Practice, and all applicable federal, state, and local laws, rules and regulations relating to the conduct of the clinical trial.

10.6 Data Management

The investigator or qualified designee is responsible for recording and verifying the accuracy of subject data. By signing this protocol, the investigator acknowledges that his/her electronic signature is the legally binding equivalent of a written signature. By entering his/her electronic signature, the investigator confirms that all recorded data have been verified as accurate.

Detailed information regarding Data Management procedures for this protocol will be provided by the Sponsor.

10.7 Publications

This trial is intended for publication, even if terminated prematurely. Publication may include any or all of the following: posting of a synopsis online, abstract and/or presentation at a scientific conference, or publication of a full manuscript. The Sponsor will work with the authors to submit a manuscript describing trial results within 12 months after the last data become available, which may take up to several months after the last subject visit in some cases such as vaccine trials. However, manuscript submission timelines may be extended on OTC trials. For trials intended for pediatric-related regulatory filings, the investigator agrees to delay publication of the trial results until the Sponsor notifies the investigator that all

relevant regulatory authority decisions on the trial drug have been made with regard to pediatric-related regulatory filings. Merck will post a synopsis of trial results for approved products on www.clinicaltrials.gov by 12 months after the last subject's last visit for the primary outcome, 12 months after the decision to discontinue development, or product marketing (dispensed, administered, delivered or promoted), whichever is later.

These timelines may be extended for products that are not yet marketed, if additional time is needed for analysis, to protect intellectual property, or to comply with confidentiality agreements with other parties. Authors of the primary results manuscript will be provided the complete results from the Clinical Study Report, subject to the confidentiality agreement. When a manuscript is submitted to a biomedical journal, the Sponsor's policy is to also include the protocol and statistical analysis plan to facilitate the peer and editorial review of the manuscript. If the manuscript is subsequently accepted for publication, the Sponsor will allow the journal, if it so desires, to post on its website the key sections of the protocol that are relevant to evaluating the trial, specifically those sections describing the trial objectives and hypotheses, the subject inclusion and exclusion criteria, the trial design and procedures, the efficacy and safety measures, the statistical analysis plan, and any amendments relating to those sections. The Sponsor reserves the right to redact proprietary information.

For multicenter trials, subsequent to the multicenter publication (or after public disclosure of the results online at www.clinicaltrials.gov if a multicenter manuscript is not planned), an investigator and his/her colleagues may publish their data independently. In most cases, publication of individual trial site data does not add value to complete multicenter results, due to statistical concerns. In rare cases, publication of single trial site data prior to the main paper may be of value. Limitations of single trial site observations in a multicenter trial should always be described in such a manuscript.

Authorship credit should be based on 1) substantial contributions to conception and design, or acquisition of data, or analysis and interpretation of data; 2) drafting the article or revising it critically for important intellectual content; and 3) final approval of the version to be published. Authors must meet conditions 1, 2 and 3. Significant contributions to trial execution may also be taken into account to determine authorship, provided that contributions have also been made to all three of the preceding authorship criteria. Although publication planning may begin before conducting the trial, final decisions on authorship and the order of authors' names will be made based on participation and actual contributions to the trial and writing, as discussed above. The first author is responsible for defending the integrity of the data, method(s) of data analysis and the scientific content of the manuscript.

The Sponsor must have the opportunity to review all proposed abstracts, manuscripts or presentations regarding this trial 45 days prior to submission for publication/presentation. Any information identified by the Sponsor as confidential must be deleted prior to submission; this confidentiality does not include efficacy and safety results. Sponsor review can be expedited to meet publication timelines.

11.0 LIST OF REFERENCES

1. Ljungman P, Griffiths P, Paya C. Definitions of cytomegalovirus infection and disease in transplant recipients. Clin Infect Dis 2002;34:1094-7.

2. Boeckh M. Complications, diagnosis, management, and prevention of CMV infections: Current and future. Am Soc Hematol 2011:305-9.

3. Boeckh M, Geballe AP. Cytomegalovirus: Pathogen, paradigm, and puzzle. J Clin Invest 2011;121(5):1673-80.

4. Boeckh M, Nichols WG. The impact of cytomegalovirus serostatus of donor and recipient before hematopoietic stem cell transplantation in the era of antiviral prophylaxis and preemptive therapy. Blood 2004;103:2003-8.

5. Nakamae H, Kirby KA, Sandmaier BM, Norasetthada L, Maloney DG, Maris MB. Effect of conditioning regimen intensity on cmv infection in allogeneic hematopoietic cell transplantation. Biol Blood Marrow Transplant 2009;15:694-703.

6. Marty FM, Ljungman P, Papanicolaou GA, Winston DJ, Chemaly RF, Strasfeld L, et al. Maribavir prophylaxis for prevention of cytomegalovirus disease in recipients of allogeneic stem-cell transplants: A phase 3, double-blind, placebo-controlled, randomised trial. Lancet Infect Dis 2011;11:284-92.

7. Sauter C, Abbound M, Jia X, Heller G, Gonzales A, Lubin M, et al. Serious infection risk and immune recovery after double-unit cord blood transplantation without antithymocyte globulin. Biol Blood Marrow Transplant 2011;17:1460-71.

8. Pollack M, Heugel J, Xie H, Leisenring W, Storek J, Young J, et al. An international comparison of current strategies to prevent herpesvirus and fungal infections in hematopoietic cell transplant recipients. Biol Blood Marrow Transplant 2011;17:664-73.

9. Boeckh M, Ljungman P. How we treat cytomegalovirus in hemtopoietic cell transplant recipients. Blood 2009;113:5711-9.

10. Biron KK. Antiviral drugs for cytomegalovirus diseases. Antiviral Res 2006;71:154-63.

11. Lurain NS, Chou S. Antiviral drug resistance of human cytomegalovirus. Clin Microbiol Rev 2010;23(4):689-712.

12. Lischka P, Hewlett G, Wunberg T, Baumeister J, Paulsen D, Goldner T, et al. In vitro and in vivo activities of the novel anticytomegalovirus compound AIC246. Antimicrob Agents Ch 2010;54(3):1290-7.

13. Goldner T, Hewlett G, Ettischer N, Ruebsamen-Schaeff H, Zimmerman H, Lischka P. The novel anticytomegalovirus compound AIC246 (letermovir) inhibits human cytomegalovirus replication through a specific antiviral mechanism that involves the viral terminase. J Virol 2011;85(20):10884-93.

14. Kaul DR, Stoelben S, Cober E, Ojo T, Sandusky E, Lischka P, et al. First report of successful treatment of multidrug-resistant cytomegalovirus disease with the novel anti-CMV compound AIC246. Am J Transplant 2011;11:1079-84.

15. Boeckh M, Leisenring W, Riddell SR, Bowden RA, Huang M-L, Myerson D, et al. Late cytomegalovirus disease and mortality in recipients of allogeneic hematopoietic stem cell transplants: Importance of viral load and T-cell immunity. Blood 2003;101(2):407-14.

16. Krause H, Hebart H, Jahn G, Müller CA, Einsele H. Screening for CMV-specific T cell proliferation to identify patients at risk of developing late onset CMV disease. Bone Marrow Transpl 1997;19:1111-6.

17. Zaia JA, Gallez-Hawkins M, Tegtmeier BR, ter Veer A, Li X, Niland JC, et al. Late cytomegalovirus disease in marrow transplantation is predicted by virus load in plasma. J Infect Dis 1997;176:782-5.

18. Broers AEC, van der Holt R, van Esser JWJ, Gratama J-W, Henzen-Logmans S, Kuenen-Boumeester V, et al. Increased transplant-related morbidity and mortality in CMV-seropositive patients despite highly effective prevention of CMVdisease after allogeneic T-cell-depleted stem cell transplantation. Blood 2000;95(7):2240-5.

19. Craddock C, Szydlo RM, Dazzi F, Olavarria E, Cwynarski K, Yong A, et al. Cytomegalovirus seropositivity adversely influences outcome after T-depleted unrelated donor transplant in patients with chronic myeloid leukaemia: The case for tailored graft-versus-host disease prophylaxis. Br J Haematol 2001;112:228-36.

20. Meijer E, Dekker AW, Rozenberg-Arska M, Weersink AJL, Verdonck LF. Influence of cytomegalovirus seropositivity on outcome after T cell-depleted bone marrow transplantation: Contrasting results between recipients of grafts from related and unrelated donors. Clin Infect Dis 2002;35:703-12.

21. Ljungman P, Perez-Bercoff L, Jonsson J, Avetisyan G, Sparrelid E, Aschan J, et al. Risk factors for the development of cytomegalovirus disease after allogeneic stem cell transplantation. Haematologica 2006;91(1):78-83.

22. Miller W, Flynn P, McCullough J, Balfour Jr. HH, Goldman A, Haake R, et al. Cytomegalovirus infection after bone marrow transplantation: An association with acute graft-v-host disease. Blood 1986;67(4):1162-7.

23. Özdemir E, Saliba RM, Champlin RE, Couriel DR, Giralt SA, de Lima M. Risk factors associated with late cytomegalovirus reactivation after allogeneic stem cell transplantation for hematological malignancies. Bone Marrow Transpl 2007;40:125-36.

24. Martino R, Rovira M, Carreras E, Solano C, Sierra J, De La Rubia J, et al. Severe infections after allogeneic peripheral blood stem cell transplantation: a matched-pair comparison of unmanipulated and CD34+ cell-selected transplantation. Haematologica 2001;86:1075-86.

25. Söderberg C, Larsson S, Bergstedt-Lindqvist S, Möller E. Definition of a subset of human peripheral blood mononuclear cells that are permissive to human cytomegalovirus infection. J Virol 1993;67(6):3166-75.

26. Larsson K, Aschan J, Remberger M, Ringdén O, Winiarski J, Ljungman P. Reduced risk for extensive chronic graft-versus-host disease in patients receiving transplants with human leukocyte antigen-identical sibling donors given polymerase chain

reaction-based preemptive therapy against cytomegalovirus. Transplantation 2004;77(4):526-31.

27. Marty FM, Boeckh M. Maribavir and human cytomegalovirus-what happened in the clinical trials and why might the drug have failed? Curr Opin Vir 2011;1:555-62.

28. Boeckh M, Bowden RA, Goodrich JM, Pettinger M, Meyers JD. Cytomegalovirus antigen detection in peripheral blood leukocytes after allogeneic marrow transplantation. Blood 1992;80(5):1358-64.

29. Winston DJ, Young JH, Pullarkat V, Papanicolaou GA, Vij R, Vance E, et al. Maribavir prophylaxis for prevention of cytomegalovirus infection in allogeneic stem cell transplant recipients: a multicenter, randomized, double-blind, placebo-controlled, dose-ranging study. Blood 2008;111(11):5403-10.

30. Ljungman P, Aschan J, Lewensohn-Fuchs I, Carlens S, Larsson K, Lönnqvist B, et al. Results of different strategies for reducing cytomegalovirus-associated mortality in allogeneic stem cell transplant recipients. Transplantation 1998;66(10):1330-4.

31. Snydman DR. Why did maribavir fail in stem-cell transplants? Lancet Infect Dis 2011;11:255-7.

32. Gerna G, Lilleri D, Furione M, Baldanti F. Management of human cytomegalovirus infection in transplantation: Validation of virologic cut-offs for preemptive therapy and immunological cut-offs for protection. New Microbiol 2011;34:229-54.

33. Lowance D, Neumayer H-H, Legendre CM, Squifflet J-P, Kovarik J, Brennan PJ, et al. Valacyclovir for the prevention of cytomegalovirus disease after renal transplantation. N Engl J Med 1999;340:1462-70.

34. Gor D, Sabin C, Prentice HG, Vyas N, Man S, Griffiths PD, et al. Longitudinal fluctuations in cytomegalovirus load in bone marrow transplant patients: Relationship between peak virus load, donor/recipient serostatus, acute gvhd and cmv disease. Bone Marrow Transpl 1998;21:597-605.

35. Atkinson C, Emery VC. Cytomegalovirus quantification: Where to next in optimising patient management? J Clin Virol 2011;51:219-24.

36. Härter G, Michel D. Antiviral treatment of cytomegalovirus infection: An update. Expert Opin Pharmacother 2012;13(5):623-7.

37. Emery VC, Sabin CA, Coppe AV, Gor D, Hassan-Walker AF, Griffiths PD. Application of viral-load kinetics to identify patients who develop cytomegalovirus disease after transplantation. Lancet 2000;355:2032-6.

38. Neofytos D, Lombardi LR, Shields RK, Ostrander D, Warren L, Nguyen MH, et al. Administration of Voriconazole in Patients With Renal Dysfunction. Clin Infect Dis 2012;54(7):913-21.

39. Miettinen O, Nurminen M. Comparative analysis of two rates. Stat Med 1985;4:213-26.

12.0 APPENDICES

12.1 Merck Code of Conduct for Clinical Trials

Merck*
Code of Conduct for Clinical Trials

I. Introduction

A. Purpose

Merck, through its subsidiaries, conducts clinical trials worldwide to evaluate the safety and effectiveness of our products. As such, we are committed to designing, implementing, conducting, analyzing and reporting these trials in compliance with the highest ethical and scientific standards. Protection of subject safety is the overriding concern in the design of clinical trials. In all cases, Merck clinical trials will be conducted in compliance with local and/or national regulations and in accordance with the ethical principles that have their origin in the Declaration of Helsinki.

B. Scope

Such standards shall be endorsed for all clinical interventional investigations sponsored by Merck irrespective of the party (parties) employed for their execution (e.g., contract research organizations, collaborative research efforts). This Code is not intended to apply to trials which are observational in nature, or which are retrospective. Further, this Code does not apply to investigator-initiated trials which are not under the control of Merck.

II. Scientific Issues

A. Trial Conduct

1. Trial Design

Except for pilot or estimation trials, clinical trial protocols will be hypothesis-driven to assess safety, efficacy and/or pharmacokinetic or pharmacodynamic indices of Merck or comparator products. Alternatively, Merck may conduct outcomes research trials, trials to assess or validate various endpoint measures, or trials to determine subject preferences, etc.

The design (i.e., subject population, duration, statistical power) must be adequate to address the specific purpose of the trial. Research subjects must meet protocol entry criteria to be enrolled in the trial.

2. Site Selection

Merck selects investigative sites based on medical expertise, access to appropriate subjects, adequacy of facilities and staff, previous performance in Merck trials, as well as budgetary considerations. Prior to trial initiation, sites are evaluated by Merck personnel to assess the ability to successfully conduct the trial.

3. Site Monitoring/Scientific Integrity

Trial sites are monitored to assess compliance with the trial protocol and general principles of Good Clinical Practice. Merck reviews clinical data for accuracy, completeness and consistency. Data are verified versus source documentation according to standard operating procedures. Per Merck policies and procedures, if fraud, misconduct or serious GCP-non-Compliance are suspected, the issues are promptly investigated. When necessary, the clinical site will be closed, the responsible regulatory authorities and ethics review committees notified and data disclosed accordingly.

B. Publication and Authorship

To the extent scientifically appropriate, Merck seeks to publish the results of trials it conducts. Some early phase or pilot trials are intended to be hypothesis-generating rather than hypothesis testing. In such cases, publication of results may not be appropriate since the trial may be underpowered and the analyses complicated by statistical issues of multiplicity.

Merck's policy on authorship is consistent with the requirements outlined in the ICH-Good Clinical Practice guidelines. In summary, authorship should reflect significant contribution to the design and conduct of the trial, performance or interpretation of the analysis, and/or writing of the manuscript. All named authors must be able to defend the trial results and conclusions. Merck funding of a trial will be acknowledged in publications.

III. Subject Protection

A. IRB/ERC review

All clinical trials will be reviewed and approved by an independent IRB/ERC before being initiated at each site. Significant changes or revisions to the protocol will be approved by the IRB/ERC prior to implementation, except that changes required urgently to protect subject safety and well-being may be enacted in anticipation of IRB/ERC approval. For each site, the IRB/ERC and Merck will approve the subject informed consent form.

B. Safety

The guiding principle in decision-making in clinical trials is that subject welfare is of primary importance. Potential subjects will be informed of the risks and benefits of, as well as alternatives to, trial participation. At a minimum, trial designs will take into account the local standard of care. Subjects are never denied access to appropriate medical care based on participation in a Merck clinical trial.

All participation in Merck clinical trials is voluntary. Subjects are enrolled only after providing informed consent for participation. Subjects may withdraw from a Merck trial at any time, without any influence on their access to, or receipt of, medical care that may otherwise be available to them.

C. Confidentiality

Merck is committed to safeguarding subject confidentiality, to the greatest extent possible. Unless required by law, only the investigator, sponsor (or representative) and/or regulatory authorities will have access to confidential medical records that might identify the research subject by name.

D. Genomic Research

Genomic Research will only be conducted in accordance with informed consent and/or as specifically authorized by an Ethics Committee.

IV. Financial Considerations

A. Payments to Investigators

Clinical trials are time- and labor-intensive. It is Merck's policy to compensate investigators (or the sponsoring institution) in a fair manner for the work performed in support of Merck trials. Merck does not pay incentives to enroll subjects in its trials. However, when enrollment is particularly challenging, additional payments may be made to compensate for the time spent in extra recruiting efforts.

Merck does not pay for subject referrals. However, Merck may compensate referring physicians for time spent on chart review to identify potentially eligible subjects.

B. Clinical Research Funding

Informed consent forms will disclose that the trial is sponsored by Merck, and that the investigator or sponsoring institution is being paid or provided a grant for performing the trial. However, the local IRB/ERC may wish to alter the wording of the disclosure statement to be consistent with financial practices at that institution. As noted above, publications resulting from Merck trials will indicate Merck as a source of funding.

C. Funding for Travel and Other Requests

Funding of travel by investigators and support staff (e.g., to scientific meetings, investigator meetings, etc.) will be consistent with local guidelines and practices including, in the U.S., those established by the American Medical Association (AMA).

V. Investigator Commitment

Investigators will be expected to review Merck's Code of Conduct as an appendix to the trial protocol, and in signing the protocol, agree to support these ethical and scientific standards.

* In this document, "Merck" refers to Merck Sharp & Dohme Corp. and Schering Corporation, each of which is a subsidiary of Merck & Co., Inc. Merck is known as MSD outside of the United States and Canada. As warranted by context, Merck also includes affiliates and subsidiaries of Merck & Co., Inc."

12.2 Collection and Management of Specimens for Future Biomedical Research

1. Definitions

a. Biomarker: A biological molecule found in blood, other body fluids, or tissues that is a sign of a normal or abnormal process or of a condition or disease. A biomarker may be used to see how well the body responds to a treatment for a disease or condition.[1]

b. Pharmacogenomics: The investigation of variations of DNA and RNA characteristics as related to drug/vaccine response.2

c. Pharmacogenetics: A subset of pharmacogenomics, pharmacogenetics is the influence of variations in DNA sequence on drug/vaccine response.2

d. DNA: Deoxyribonucleic acid.

e. RNA: Ribonucleic acid.

2. Scope of Future Biomedical Research

The DNA and left over plasma specimen(s) collected in the current trial will be used to study various causes for how subjects may respond to a drug/vaccine. The DNA and left over plasma specimen(s) will be stored to provide a resource for future trials conducted by Merck focused on the study of biomarkers responsible for how a drug/vaccine enters and is removed by the body, how a drug/vaccine works, other pathways a drug/vaccine may interact with, or other aspects of disease. The specimen(s) may be used for future assay development and/or drug/vaccine development.

It is now well recognized that information obtained from studying and testing clinical specimens offers unique opportunities to enhance our understanding of how individuals respond to drugs/vaccines, enhance our understanding of human disease and ultimately improve public health through development of novel treatments targeted to populations with the greatest need. All specimens will be used by Merck or designees and research will be monitored and reviewed by a committee of our scientists and clinicians.

3. Summary of Procedures for Future Biomedical Research

a. Subjects for Enrollment

All subjects enrolled in the clinical trial will be considered for enrollment in the Future Biomedical Research sub-trial.

b. Informed Consent

Informed consent for specimens (i.e., DNA, RNA, protein, etc.) will be obtained during screening for protocol enrollment from all subjects or legal guardians, at a trial visit by the investigator or his or her designate. Informed consent for Future Biomedical Research should be presented to the subjects on Visit 1. If delayed, present consent at next possible Subject Visit. Informed consent must be obtained prior to collection of all Future Biomedical Research specimens. Consent forms signed by the subject will be kept at the clinical trial site under secure storage for regulatory reasons. Information contained on the consent form alone cannot be traced to any specimens, test results, or medical information once the specimens have been rendered de-identified.

Subjects are not required to participate in the Future Biomedical Research sub-trial in order to participate in the main trial. Subjects who decline to sign the Future Biomedical Research informed consent will not have the specimen collected nor will they be discontinued from the main trial.

A template of each trial site's approved informed consent will be stored in the Sponsor's clinical document repository. Each consent will be assessed for appropriate specimen permissions.

Each informed consent approved by an ethics committee is assigned a unique tracking number. The tracking number on this document will be used to assign specimen permissions for each specimen into the Entrusted Keyholder's Specimen Database.

c. eCRF Documentation for Future Biomedical Research Specimens

Documentation of both consent and acquisition of Future Biomedical Research specimens will be captured in the electronic Case Report Forms (eCRFs). Reconciliation of both forms will be performed to assure that only appropriately-consented specimens are used for this sub-trial's research purposes. Any specimens for which such an informed consent cannot be verified will be destroyed.

d. Future Biomedical Research Specimen Collections

Blood specimens for DNA or RNA isolation will usually be obtained at a time when the subject is having blood drawn for other trial purposes. Specimens like tissue and bone marrow will usually be obtained at a time when the subject is having such a procedure for clinical purposes.

Specimens will be collected and sent to the laboratory designated for the trial where they will be processed (e.g., DNA or RNA extraction, etc) following the Merck approved policies and procedures for specimen handling and preparation.

If specimens are collected for a specific genotype or expression analysis as an objective to the main trial, this analysis is detailed in the main body of this protocol (**Section 8.0 – Statistical Analysis Plan**). These specimens will be processed, analyzed, and the remainder of the specimen will be destroyed. The results of these analyses will be reported along with the other trial results. A separate specimen will be obtained from properly-consented subjects in this protocol for storage in the biorepository for Future Biomedical Research.

4. Confidential Subject Information for Future Biomedical Research

In order to optimize the research that can be conducted with Future Biomedical Research specimens, it is critical to link subject' clinical information with future test results. In fact little or no research can be conducted without connecting the clinical trial data to the specimen. The clinical data allow specific analyses to be conducted. Knowing subject characteristics like gender, age, medical history and treatment outcomes are critical to understanding clinical context of analytical results.

To maintain privacy of information collected from specimens obtained for Future Biomedical Research, Merck has developed secure policies and procedures. All specimens will be de-identified as described below.

At the clinical trial site, unique codes will be placed on the Future Biomedical Research specimens for transfer to the storage facility. This first code is a random number which does not contain any personally identifying information embedded within it. The link (or key) between subject identifiers and this first unique code will be held at the trial site. No personal identifiers will appear on the specimen tube.

This first code will be replaced with a second code at a Merck designated storage/lab facility. The second code is linked to the first code via a second key. The specimen is now double coded. Specimens with the second code are sometimes referred to as de-identified specimens. The use of the second code provides additional confidentiality and privacy protection for subjects over the use of a single code. Access to both keys would be needed to link any data or specimens back to the subject's identification.

The second code is stored separately from the first code and all associated personal specimen identifiers. A secure link, the second key, will be utilized to match the second code to the first code to allow clinical information collected during the course of the trial to be associated with the specimen. This second key will be transferred under secure procedures by the Merck designated facility to an Entrusted Keyholder at Merck. The second code will be logged into the primary biorepository database at Merck and, in this database, this identifier will not have identifying demographic data or identifying clinical information (i.e., race, sex, age, diagnosis, lab values) associated with it. The specimen will be stored in a designated biorepository site with secure policies and procedures for specimen storage and usage.

The second key can be utilized to reconstruct the link between the results of future biomedical research and the clinical information, at the time of analysis. This linkage would not be possible for the scientist conducting the analysis, but can only be done by the Merck Entrusted Keyholder under strict security policies and procedures. The Merck Entrusted Keyholder will link the information and then issue a de-identified data set for analysis. The only other circumstance by which future biomedical research data would be directly linked to the full clinical data set would be those situations mandated by regulatory authorities (e.g., EMEA, FDA), whereby this information would be directly transferred to the regulatory authority.

5. Biorepository Specimen Usage

Specimens obtained for the Merck Biorepository will be used for analyses using good scientific practices. However, exploratory analyses will not be conducted under the highly validated conditions usually associated with regulatory approval of diagnostics. The scope of research performed on these specimens is limited to the investigation of the variability in biomarkers that may correlate with a clinical phenotype in subjects.

Analyses utilizing the Future Biomedical Research specimens may be performed by Merck, or an additional third party (e.g., a university investigator) designated by Merck. The investigator conducting the analysis will be provided with double coded specimens. Re-association of analysis results with corresponding clinical data will only be conducted by the Merck Entrusted Keyholder. Any contracted third party analyses will conform to the specific scope of analysis outlined in this sub-trial. Future Biomedical Research specimens remaining with the third party after the specific analysis is performed will be

returned to the sponsor or destroyed and documentation of destruction will be reported to Merck.

6. Withdrawal From Future Biomedical Research

Subjects may withdraw their consent for Future Biomedical Research and have their specimens and all derivatives destroyed. Subjects may withdraw consent at any time by contacting the principal investigator for the main trial. If medical records for the main trial are still available, the investigator will contact Merck using the designated mailbox PPD [redacted] and a form will be provided by Merck to obtain appropriate information to complete specimen withdrawal. Subsequently, the subject's specimens will be removed from the biorepository and be destroyed. A letter will be sent from Merck to the investigator confirming the destruction. It is the responsibility of the investigator to inform the subject of completion of destruction. Any analyses in progress at the time of request for destruction or already performed prior to the request being received by the Sponsor will continue to be used as part of the overall research trial data and results. No new analyses would be generated after the request is received.

In the event that the medical records for the main trial are no longer available (e.g., if the investigator is no longer required by regulatory authorities to retain the main trial records) or the specimens have been completely anonymized, there will no longer be a link between the subject's personal information and their specimens. In this situation, the request for specimen destruction can not be processed.

7. Retention of Specimens

Future Biomedical Research specimens will be stored in the biorepository for potential analysis for up to 20 years from acquisition. Specimens may be stored for longer if a regulatory or governmental authority has active questions that are being answered. In this special circumstance, specimens will be stored until these questions have been adequately addressed.

Specimens from the trial site will be shipped to a central laboratory and then shipped to the Merck designated biorepository. The specimens will be stored under strict supervision in a limited access facility which operates to assure the integrity of the specimens. Specimens will be destroyed according to Merck policies and procedures and this destruction will be documented in the biorepository database.

8. Data Security

Separate databases for specimen information and for results from the Future Biomedical Research sub-trial will be maintained by Merck. This is done to separate the future exploratory test results (which include genetic data) from the clinical trial database thereby maintaining a separation of subject number and these results. The separate databases are accessible only to the authorized Sponsor and the designated trial administrator research personnel and/or collaborators. Database user authentication is highly secure, and is accomplished using network security policies and practices based in international standards (e.g., ISO17799) to protect against unauthorized access. The Merck Entrusted Keyholder maintains control over access to all specimen data. These

data are collected for future biomedical research purposes only as specified in this sub-trial will not be used for any other purpose.

9. Reporting of Future Biomedical Research Data to Subjects

There is no definitive requirement in either authoritative ethical guidelines or in relevant laws/regulations globally that research results have to be, in all circumstances, returned to the trial participant. Some guidelines advocate a proactive return of data in certain instances. No information obtained from exploratory laboratory studies will be reported to the subject or family, and this information will not be entered into the clinical database maintained by Merck on subjects. Principle reasons not to inform or return results to the subject include: lack of relevance to subject health, limitations of predictive capability, concerns of misinterpretation and absence of good clinical practice standards in exploratory research typically used for diagnostic testing.

If any exploratory results are definitively associated with clinical significance for subjects while the clinical trial is still ongoing, investigators will be contacted with information as to how to offer clinical diagnostic testing (paid for by Merck) to subjects enrolled and will be advised that counseling should be made available for all who choose to participate in this diagnostic testing.

If any exploratory results are definitively associated with clinical significance after completion of a clinical trial, Merck will publish the results without revealing specific subject information, inform all trial sites who participated in the Merck clinical trial and post anonymized results on our website or other accredited website(s) that allow for public access (e.g., disease societies who have primary interest in the results) in order that physicians and patients may pursue clinical diagnostic testing if they wish to do so.

10. Gender, Ethnicity and Minorities

Although many diagnoses differ in terms of frequency by ethnic population and gender, every effort will be made to recruit all subjects diagnosed and treated on Merck clinical trials for future biomedical research. When trials with specimens are conducted and subjects identified to serve as controls, every effort will be made to group specimens from subjects and controls to represent the ethnic and gender population representative of the disease under current investigation.

11. Risks Versus Benefits of Future Biomedical Research

For future biomedical research, risks to the subject have been minimized. Risks include those associated with venipuncture to obtain the whole blood specimen. This specimen will be obtained at the time of routine blood specimens drawn in the main trial.

Merck has developed strict security, policies and procedures to address subject data privacy concerns. Data privacy risks are largely limited to rare situations involving possible breach of confidentiality. In this highly unlikely situation there is risk that the information, like all medical information, may be misused.

It is necessary for subject-related data (i.e., ethnicity, diagnosis, drug therapy and dosage, age, toxicities, etc.) to be re-associated to double coded specimens at the time of data analysis. These subject data will be kept in a separate, secure Merck database, and all specimens will be stripped of subject identifiers. No information concerning results

obtained from future biomedical research will be entered into clinical records, nor will it be released to outside persons or agencies, in any way that could be tied to an individual subject.

12. Self-Reported Ethnicity

Subjects who participate in future biomedical research will be asked to provide self-reported ethnicity. Subjects who do not wish to provide this data may still participate in future biomedical research.

13. Questions

Any questions related to the future biomedical research should be e-mailed directly to PPD

14. References

1. National Cancer Institute: http://www.cancer.gov/dictionary/?searchTxt=biomarker
2. International Conference on Harmonization: DEFINITIONS FOR GENOMIC BIOMARKERS, PHARMACOGENOMICS, PHARMACOGENETICS, GENOMIC DATA AND SAMPLE CODING CATEGORIES - E15; http://www.ich.org/LOB/media/MEDIA3383.pdf

12.3 Understanding the Intent, Scope and Public Health Benefits of Exploratory Biomarker Research: A Guide for IRBs/IECs and Investigational Site Staff

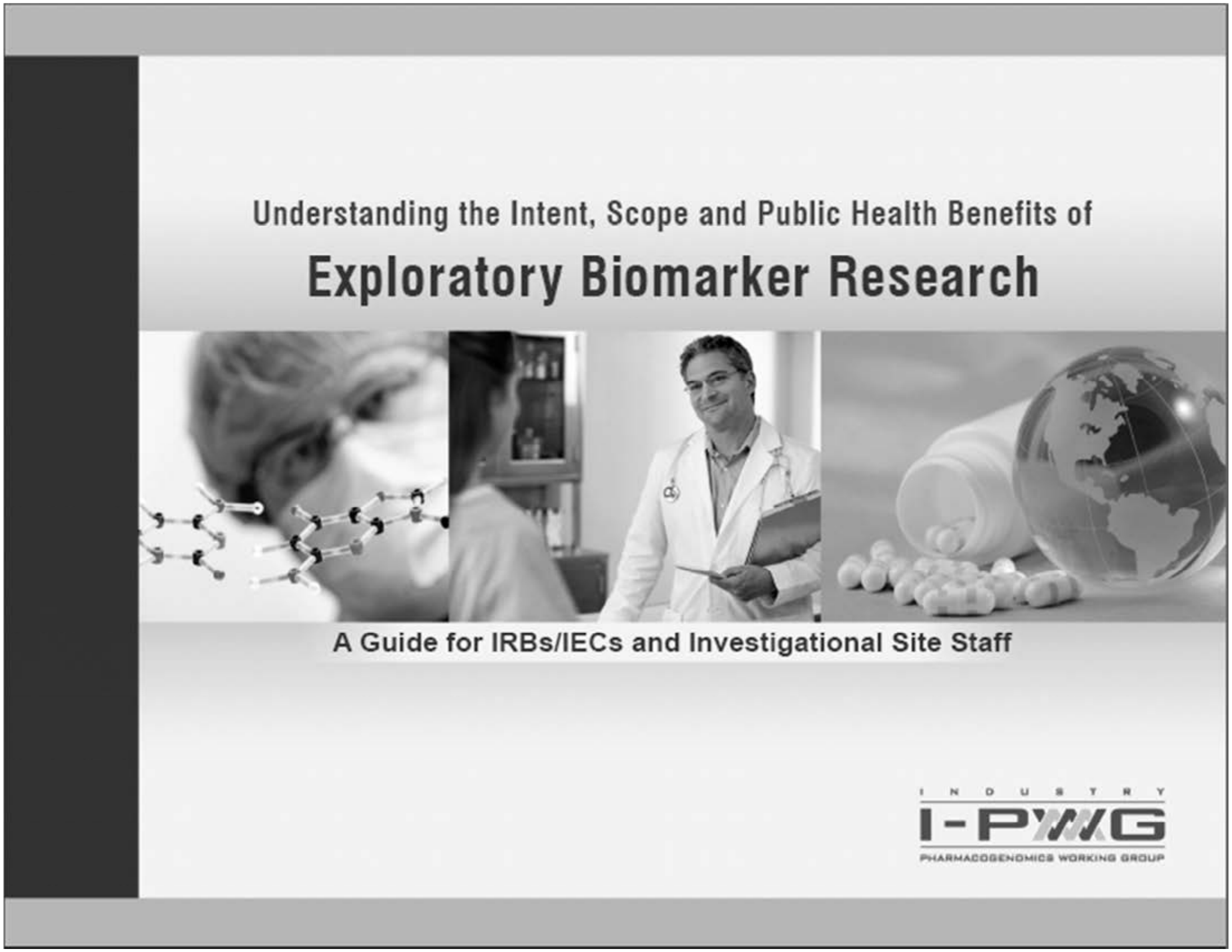

This informational brochure is intended for IRBs/IECs and Investigational Site Staff. The brochure addresses issues relevant to specimen collection for biomarker research in the context of pharmaceutical drug and vaccine development.

Developed by
The Industry Pharmacogenomics Working Group (I-PWG)
www.i-pwg.org

1. What is a Biomarker and What is Biomarker Research?

A biomarker is a *"characteristic that is objectively measured and evaluated as an indicator of normal biological processes, pathogenic processes, or pharmacologic responses to a therapeutic intervention".* [1]

Biomarker research, including research on pharmacogenomic biomarkers, is a tool used to improve the development of pharmaceuticals and understanding of disease. It involves the analysis of biomolecules (such as DNA, RNA, proteins, and lipids), or other measurements (such as blood pressure or brain images) in relation to clinical endpoints of interest. Biomarker research can be influential across all phases of drug development, from drug discovery and preclinical evaluations to clinical development and post-marketing studies. This brochure focuses on biomarker research involving analysis of biomolecules from biological samples collected in clinical trials. Please refer to I-PWG Pharmacogenomic Informational Brochure[2] and ICH Guidance E15[3] for additional information specific to pharmacogenomic biomarkers.

2. Why is Biomarker Research Important?

Importance to Patients and Public Health

Biomarker research is helping to improve our ability to predict, detect, and monitor diseases and improve our understanding of how individuals respond to drugs. This research underlies personalized medicine: a tailored approach to patient treatment based on the molecular analysis of genes, proteins, and metabolites.[4] The goal of biomarker research is to aid clinical decision-making toward safer and more efficacious courses of treatment, improved patient outcomes, and overall cost-savings. It also allows for the continued development and availability of drugs that are effective in certain sub-populations when they otherwise might not have been developed due to insufficient efficacy in the broader population.

Recent advances in biomedical technology, including genetic and molecular medicine, have greatly increased the power and precision of analytical tools used in health research and have accelerated the drive toward personalized medicine. In some countries, highly focused initiatives have been created to promote biomarker research (e.g., in the US: www.fda.gov/oc/initiatives/criticalpath/ ; in the EU: www.imi.europa.eu/index_en.html).

Importance to Drug Development

Biomarker research is being used by the pharmaceutical industry to streamline the drug development process. Some biomarkers are used as substitutes or "surrogates" for safety or efficacy endpoints in clinical trials particularly where clinical outcomes or events cannot practically or ethically be measured (e.g., cholesterol as a surrogate for cardiovascular disease).[5] By using biomarkers to assess patient response, ineffective drug candidates may be terminated earlier in the development process in favor of more promising drug candidates. Biomarkers are being used to optimize clinical trial designs and outcomes by identifying patient populations that are more likely to respond to a drug therapy or to avoid specific adverse events.

Biomarker research is also being used to enhance scientific understanding of the mechanisms of both treatment response and disease processes, which can help to identify future targets for drug development. Depending on the clinical endpoints in a clinical trial, biomarker sample collection may either be a required or optional component of the trial. However, both mandatory and optional sample collections are important for drug development.

3. Importance of Biomarkers to Regulatory Authorities

Regulatory health authorities are increasingly aware of the benefits of biomarkers and how they may be used for drug approval, clinical trial design, and clinical care. Biomarkers have been used to establish risk:benefit profiles. For example, the FDA has modified the US warfarin (Coumadin®) label to include the analysis of *CYP2C9* and *VKORC1* genes to guide dosing regimens. Health authorities such as the FDA (USA), EMEA (European Union), MHLW (Japan), and ICH (International) are playing a key role in advancing this scientific field as it applies to pharmaceutical development by creating the regulatory infrastructure to facilitate this research. Numerous regulatory guidances and concept papers have already been issued, many of which are available through www.i-pwg.org. Global regulatory authorities have highlighted the importance of biomarker research and the need for the pharmaceutical industry to take the lead in this arena.[3, 6-24]

4. How are Biomarkers Being Used in Drug/Vaccine Development?

Biomarker research is currently being used in drug/vaccine development to:

- Explain variability in response among participants in clinical trials
- Better understand the mechanism of action or metabolism of investigational drugs
- Obtain evidence of pharmacodynamic activity (i.e., how the drug affects the body) at the molecular level
- Address emerging clinical issues such as unexpected adverse events
- Determine eligibility for clinical trials to optimize trial design
- Optimize dosing regimens to minimize adverse reactions and maximize efficacy
- Develop drug-linked diagnostic tests to identify patients who are more likely or less likely to benefit from treatment or who may be at risk of experiencing adverse events
- Provide better understanding of mechanisms of disease
- Monitor clinical trial participant response to medical interventions

> Biomarker research, including research on banked samples, should be recognized as an important public health endeavor for the overall benefit of society, whether by means of advancement of medical science or by development of safer and more effective therapies.[7] Since the value of collected samples may increase over time as scientific discoveries are made, investment in long-term sample repositories is a key component of biomarker research.

I-PWG

5. Biomarkers are Already a Reality in Health Care

A number of drugs now have biomarker information included in their labels.[25] Biomarker tests are already being used in clinical practice to serve various purposes:

Predictive biomarkers (efficacy) – In clinical practice, predictive efficacy biomarkers are used to predict which patients are most likely to respond, or not respond, to a particular drug. Examples include: i) *Her2/neu* overexpression analysis required for prescribing trastuzumab (Herceptin®) to breast cancer patients, ii) *c-kit* expression analysis prior to prescribing imatinib mesylate (Gleevec®) to gastrointestinal stromal tumor patients, and iii) *KRAS* mutational status testing prior to prescribing panitumumab (Vectibix®) or cetuximab (Erbitux®) to metastatic colorectal cancer patients.

Predictive biomarkers (safety) – In clinical practice, predictive safety biomarkers are used to select the proper drug dose or to evaluate the appropriateness of continued therapy in the event of a safety concern. Examples include: i) monitoring of blood potassium levels in patients receiving drospirenone and ethinyl estradiol (Yasmin®) together with daily long-term drug regimens that may increase serum potassium, and ii) prospective *HLA-B*5701* screening to identify those at increased risk for hypersensitivity to abacavir (Ziagen®).

Surrogate biomarkers – In clinical practice, surrogate biomarkers may be used as alternatives to measures such as survival or irreversible morbidity. Surrogate biomarkers are measures that are reasonably likely, based on epidemiologic, therapeutic, pathophysiologic, or other evidence, to predict clinical benefit. Examples include: i) LDL level as a surrogate for risk of cardiovascular diseases in patients taking lipid-lowering agents such as atorvastatin calcium (Lipitor®), ii) blood glucose as a surrogate for clinical outcomes in patients taking anti-diabetic agents, and iii) HIV plasma viral load and CD4 cell counts as surrogates for time-to-clinical-events and overall survival in patients receiving antiretroviral therapy for HIV disease.

Prognostic biomarkers – Biomarkers can also help predict clinical outcomes independent of any treatment modality. Examples of prognostic biomarkers used in clinical practice include: i) CellSearch™ to predict progression-free survival in breast cancer, ii) anti-CCP (cyclic citrullinated protein) for the severity of rheumatoid arthritis, iii) estrogen receptor status for breast cancer, and iv) anti-dsDNA for the severity of systemic lupus erythematosus.

6. Biomarker Samples from Clinical Trials: An Invaluable Resource

Adequate sample sizes and high-quality data from controlled clinical trials are key to advancements in biomarker research. Samples collected in clinical trials create the opportunity for investigation of biomarkers related to specific drugs, drug classes, and disease areas. Clinical drug development programs are therefore an invaluable resource and a unique opportunity for highly productive biomarker research. In addition to conducting independent research, pharmaceutical companies are increasingly contributing to consortia efforts by pooling samples, data, and expertise in an effort to conduct rigorous and efficient biomarker research and to maximize the probability of success.[26-27]

7. Informed Consent for Collection & Banking of Biomarker Samples

Collection of biological samples in clinical trials must be undertaken with voluntary informed consent of the participant (or legally-acceptable representative). Policies

and regulations for legally-appropriate informed consent vary on national, state, and local levels, but are generally based on internationally recognized pillars of ethical conduct for research on human subjects.[28-31]

Optional vs. Required Subject Participation

Depending on the relevance of biomarker research to a clinical development program at the time of protocol development, the biomarker research may be a core required component of a trial (e.g., key to elucidating the drug mechanism of action or confirming that the drug is interacting with the target) or may be optional (e.g., to gain valuable knowledge that enhances the understanding of diseases and drugs). Informed consent for the collection of biomarker samples may be presented either in the main clinical informed consent form or as a separate informed consent form, with approaches varying somewhat across pharmaceutical companies. The relevance of biomarker research to a clinical development program may change over time as the science evolves. The samples may therefore increase in value after a protocol is developed.

Consent for Future Research Use

While it can be a challenge to specify the details of the research that will be conducted in the future, the I-PWG holds the view that future use of samples collected for exploratory biomarker research in clinical trials should be permissible when i) the research is scientifically sound, ii) participants are informed of the scope of the intended future research, even if this is broadly defined (see potential uses in Section 4 above), iii) autonomy is respected by providing the option to consent separately to future use of samples or by providing the option to terminate further use of samples upon request (consent withdrawal / sample destruction), and iv) industry standards for confidentiality protection per Good Clinical Practice guidelines are met.[3, 31] Importantly, any research using banked samples should be consistent with the original informed consent, except where otherwise permitted by local law or regulation.

Important elements of informed consent for **future use** of samples include, but are not limited to:[39]

The scope of research – Where the scope of the potential future research is broad, participants should be informed of the boundaries of the research. While it may not be possible to describe the exact analytical techniques that will be used, or specific molecules that will be analyzed, it is possible to clearly articulate in reasonable detail the type of research to be conducted and its purpose. Information regarding whether stored samples may be shared with other parties or utilized for commercialization purposes should also be addressed.

Withdrawal of consent / sample destruction – The informed consent form should inform participants of their right to withdraw their consent / request destruction of their samples. This should include the mechanisms for exercising that right and any limitations to exercising that right. For example, participants should be informed that it is not possible to destroy samples that have been anonymized.[3] In addition, according to industry standards and regulatory guidance, participants should be informed that data already generated prior to a consent withdrawal request are to be maintained as part of the study data.[38]

The duration of storage – The permissible duration of storage may vary according to the nature and uses of the samples and may also vary on national, state, and local levels. The intended duration of storage, including indefinite storage, should be specified.

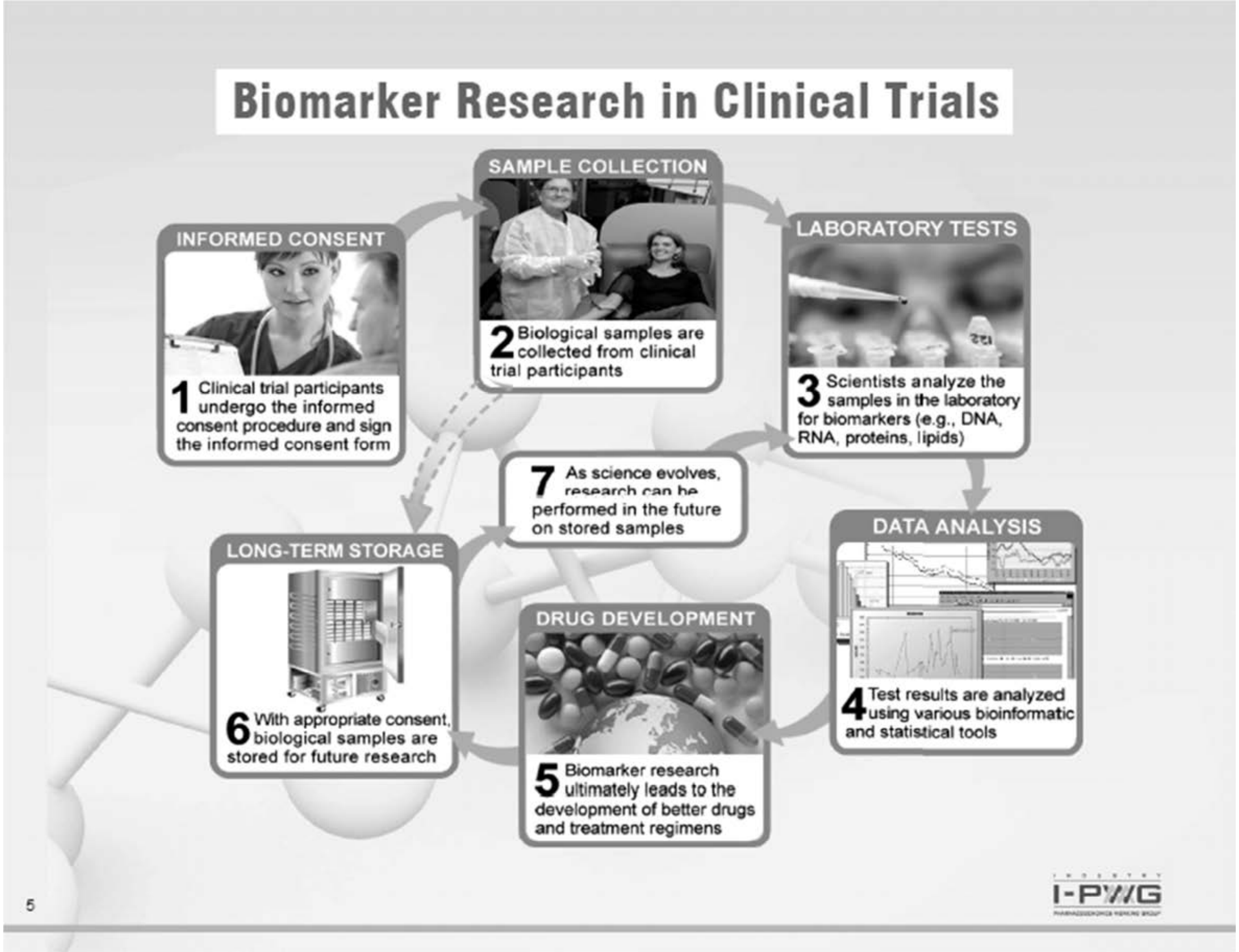
Biomarker Research in Clinical Trials
INFORMED CONSENT
1 Clinical trial participants undergo the informed consent procedure and sign the informed consent form
SAMPLE COLLECTION
2 Biological samples are collected from clinical trial participants
LABORATORY TESTS
3 Scientists analyze the samples in the laboratory for biomarkers (e.g., DNA, RNA, proteins, lipids)
DATA ANALYSIS
4 Test results are analyzed using various bioinformatic and statistical tools
DRUG DEVELOPMENT
5 Biomarker research ultimately leads to the development of better drugs and treatment regimens
LONG-TERM STORAGE
6 With appropriate consent, biological samples are stored for future research
7 As science evolves, research can be performed in the future on stored samples
5
I-PWG

8. Biomarker Sample Collection in Different Countries

Collection of biological samples for biomarker research is straightforward in most jurisdictions. Some countries have specific laws and regulations regarding collection, labeling, storage, export, and/or use of exploratory samples. In addition, some regulations distinguish between DNA and non-DNA samples or between samples used for diagnostic purposes and samples collected for scientific research. Processes for the collection, labeling, storage, export, and/or use of biomarker samples should always adhere to the laws and regulations of the country/region in which those samples are collected.

9. Return of Research Results to Study Participants

Policies for the return of biomarker research results to study participants who request them vary among pharmaceutical companies. There are many considerations that pharmaceutical companies weigh when determining their policy regarding the return of biomarker research results to study participants. These include:

i) the conditions under which biomarker research results were generated (i.e., exploratory research laboratory versus accredited diagnostic laboratory)

ii) whether the results will have an impact on the medical care of the participant or on a related person, if applicable

iii) whether genetic counseling is recommended (for genetic results)

iv) the ability to accurately link the result to the individual from whom the sample was collected

v) international, national, and local guidelines, policies, legislation, and regulations regarding participants' rights to access data generated on them

Renegar *et al*. 2006 and Article 29 Data Protection Working Party (an advisory committee to the European Commission on the European Data Protection Directive) have addressed these considerations in detail in relation to pharmacogenomic research data and provided a list of documents addressing the general issue of return of research results.[34-35]

10. Benefits and Risks Associated with Biomarker Research

Benefits
While it may not always directly benefit the study participant who is providing the samples, biomarker research can improve overall understanding of disease and treatment of future patients receiving therapies developed from such research. Patients are now benefiting from retrospective biomarker research conducted on samples collected from clinical trials and stored for exploratory research. One example is the recent label update to the EGFR antibody drugs cetuximab (Erbitux®) and panitumumab (Vectibix®) which highlights the value of *KRAS* status as a predictive biomarker for treatment of metastatic colorectal cancer with this class of drug.

The humanitarian benefit of human research is recognized by the Nuremberg Code.[28,33] Provided that the degree of risk does not exceed that determined by the humanitarian importance of the problem to be solved, research participants should not be denied the right to contribute to the greater common good.[28,32]

Risks
Risks associated with biomarker research are primarily related to the physical aspects of obtaining the sample and to patient privacy concerns.

Physical risks associated with biomarker sample collection in clinical trials can be characterized in two ways: i) negligible additional risk when the biomarker sample is collected as part of a procedure conducted to support

other core trial objectives, and ii) some added risk where the sampling procedure would otherwise have not been performed as a core component of a trial. Risks are also determined by the invasiveness of the sample collection procedure.

Privacy risks are generally those associated with the inappropriate disclosure and misuse of data. Pharmaceutical companies have policies and procedures for confidentiality protection to minimize this risk for all data collected and generated in clinical trials. These may vary across companies, but are based on industry standards of confidentiality and privacy protection highlighted in the following section. Importantly, privacy risks inherent to biomarker data are no greater than other data collected in a clinical trial.

11. Privacy, Confidentiality, and Patient Rights

Maintaining the privacy of study participants and the confidentiality of information relating to them is of paramount concern to industry researchers, regulators, and patients. Good Clinical Practice (GCP), the standard adhered to in pharmaceutical clinical research, is a standard that

"...provides assurance that the data and reported results are credible and accurate, and that the rights, integrity, and confidentiality of trial subjects are protected",

where confidentiality is defined as, *"The prevention of disclosure, to other than authorized individuals, of a sponsor's proprietary information or of a subject's identity."*

This standard dictates that *"the confidentiality of records that could identify subjects should be protected, respecting the privacy and confidentiality rules in accordance with applicable regulatory requirements."* [31]

Exploratory biomarker research in pharmaceutical development is commonly conducted in research laboratories that are not accredited to perform diagnostic tests used for healthcare decision-making. Therefore, results from exploratory biomarker research usually are not appropriate for use in making decisions about a trial participant's health. In addition, exploratory research data should not be included as part of a participant's medical record accessible for use by insurance companies. Legislation and policies to protect individuals against discrimination based on genetic information continually evolve based on social, ethical, and legal considerations. Examples of such legislation include the Human Tissue Act 2004 (UK) and the Genetic Information Nondiscrimination Act (GINA) 2008 (USA).[36-37]

12.Where to Get More Information?

Educational resources related to biomarker and pharmacogenomic research that caters to health care professionals, IRBs/IECs, scientists, and patients are continually being created and are publicly available. Links to many of these resources are available through the I-PWG website: www.i-pwg.org.

13. What is I-PWG?

The Industry Pharmacogenomics Working Group (I-PWG) (formerly the Pharmacogenetics Working Group) is a voluntary association of pharmaceutical companies engaged in pharmacogenomic research. The Group's activities focus on non-competitive educational, informational, ethical, legal, and regulatory topics. The Group provides information and expert opinions on these topics and sponsors educational/ informational programs to promote better understanding of pharmacogenomic and other biomarker research for key stakeholders. The I-PWG interacts with regulatory author-

ities and policy groups to ensure alignment. More information about the I-PWG is available at: www.i-pwg.org.

14. Contributing authors

Monique A. Franc, Teresa Hesley, Feng Hong, Ronenn Roubenoff, Jasjit Sarang, Andrea Tyukody Renninger, Amelia Warner

15. References

1. Atkinson AJ, Colburn WA, DeGruttola VG, et al. Biomarkers and surrogate endpoints: Preferred definitions and conceptual framework. Clinical Pharmacology & Therapeutics 2001; 69(3): 89-95. (Accessed at: www.ncbi.nlm.nih.gov/pubmed/11240971)
2. I - PWG Pharmacogenomics Informational Brochure, 2008. (Accessed at: http//:www.i-pwg.org/cms/index.php?option=com_docman&task=doc_download&gid=77&Itemid=118)
3. ICH E15 – Definitions for Genomic Biomarkers, Pharmacogenomics, Pharmacogenetics, Genomic Data and Sample Coding Categories. April 2008. (Accessed at: www.fda.gov/OHRMS/DOCKETS/98fr/FDA-2008-D-0199-gdl.pdf and at: http://www.ich.org/LOB/media/MEDIA3383.pdf)
4. Davis JC, Furstenthal L, Desai AA, et al. The microeconomics of personalized medicine: today's challenge and tomorrow's promise. Nature Reviews Drug Discovery, 2009; 8: 279. (Accessed at:http: www.nature.com/nrd/journal/v8/n4/abs/nrd2825.html)
5. Berns B, Démolis P, Scheulen ME. How can biomarkers become surrogate endpoints? European Journal of Cancer Supplements 2007; 5: 37-40. (Accessed at: www.journals.elsevierhealth.com/periodicals/ejcsup/issues/contents?issue_key=S1359-6349%2807%29X0031-4)
6. Lesko LJ, Woodcock J. Translation of pharmacogenomics and pharmacogenetics: a regulatory perspective. Nature Reviews Drug Discovery, 2004; 3: 763-769. (Accessed at: www.nature.com/nrd/journal/v3/n9/abs/nrd1499.html)
7. Lesko LJ, Woodcock J. Pharmacogenomic-guided drug development: regulatory perspective. The Pharmacogenomics Journal, 2002; 2: 20-24. (Accessed at www.ncbi.nlm.nih.gov/pubmed/11990376)
8. Petricoin EF, Hackett JL, Lesko LJ, et al. Medical applications of microarray technologies: a regulatory science perspective. Nat Genet., 2002; 32: 474-479. (Accessed at: www.nature.com/ng/journal/v32/n4s/abs/ng1029.html)
9. Lesko LJ, Salerno RA, Spear BB, et al. Pharmacogenetics and pharmacogenomics in drug development and regulatory decision making: report of the first FDA-PWG-PhRMA-DruSafe Workshop. J Clin Pharmacol., 2003; 43: 342-358. (Accessed at: http://jcp.sagepub.com/cgi/content/abstract/43/4/342)
10. Salerno RA, Lesko LJ. Pharmacogenomics in Drug Development and Regulatory Decision-making: the Genomic Data Submission (GDS) Proposal. Pharmacogenomics, 2004; 5: 25-30. (Accessed at: www.futuremedicine.com/doi/pdf/10.2217/14622416.5.1.25)
11. Frueh FW, Goodsaid F, Rudman A, et al. The need for education in pharmacogenomics: a regulatory perspective. The Pharmacogenomics Journal, 2005; 5: 218-220. (Accessed at: www.nature.com/tpj/journal/v5/n4/abs/6500316a.html)
12. Genomic Biomarkers Related to Drug Response: Context, Structure and Format of Qualification Submissions. ICH E16 Step 3 draft. (Accessed at: www.emea.europa.eu/pdfs/human/ich/38063609endraft.pdf)
13. Guiding principles Processing Joint FDA EMEA Voluntary Genomic Data Submissions (VGDSs) within the framework of the Confidentiality Arrangement. May 19, 2006. (Accessed at: www.fda.gov/downloads/Drugs/ScienceResearch/ResearchAreas/Pharmacogenetics/ucm085378.pdf)
14. Guidance for Industry Pharmacogenomic Data Submissions. FDA. March 2005. (Accessed at: www.fda.gov/downloads/Drugs/GuidanceComplianceRegulatoryInformation/Guidances/ucm079849.pdf)
15. Pharmacogenomic Data Submissions - Companion Guidance. FDA Draft Guidance. August 2007. (Accessed at: www.fda.gov/downloads/Drugs/GuidanceComplianceRegulatoryInformation/Guidances/ucm079855.pdf)
16. Reflection Paper on Pharmacogenomics in Oncology. EMEA. 2008. (Accessed at: www.emea.europa.eu/pdfs/human/pharmacogenetics/12843506endraft.pdf)
17. Position paper on Terminology in Pharmacogenetics. EMEA. 2002. (Accessed at: www.emea.europa.eu/pdfs/human/press/pp/307001en.pdf)
18. Concept paper on the development of a Guideline on the use of pharmacogenomic methodologies in the pharmacokinetic evaluation of medicinal products. EMEA. 2009. (Accessed at: www.emea.europa.eu/pdfs/human/pharmacogenetics/6327009en.pdf)
19. Reflection paper on Pharmacogenomic samples, testing and data handling. EMEA. 2007. (Accessed at: www.emea.europa.eu/pdfs/human/pharmacogenetics/20191406en.pdf)
20. Ishiguro A, Toyoshima S, Uyama Y. Current Japanese regulatory situations of pharmacogenomics in drug administration. Expert Review of Clinical Pharmacology, 2008;1: 505-514. (Accessed at: www.ingentaconnect.com/content/ftd/ecp/2008/00000001/00000004/art00007)
21. Amur S, Frueh FW, Lesko LJ, et al. Integration and use of

I-PWG

biomarkers in drug development, regulation and clinical practice: A US regulatory practice. Biomarkers Med. 2008; 2, 305-311. (Accessed at: www.ingentaconnect.com/content/fm/bmm/2008/00000002/00000003/art00010?crawler=true)

22. Mendrick DL, Brazell C, Mansfield EA, et al. Pharmacogenomics and regulatory decision making: an international perspective. The Pharmacogenomics Journal. 2006; 6(3), 154-157. (Accessed at: www.nature.com/tpj/journal/v6/n3/abs/6500364a.html)

23. Pendergast MK. Regulatory agency consideration of pharmacogenomics. Exp Biol Med (Maywood). 2008; 233:1498-503. (Accessed at: www.ebmonline.org/cgi/content/abstract/233/12/1498)

24. Goodsaid F, Frueh F. Process map proposal for the validation of genomic biomarkers. Pharmacogenomics., 2006; 7(5):773-82 (Accessed at: www.futuremedicine.com/doi/abs/10.2217/14622416.7.5.773)

25. FDA Table of Valid Genomic Biomarkers in the Context of Approved Drug Labels. (Accessed at: www.fda.gov/Drugs/ScienceResearch/ResearchAreas/Pharmacogenetics/ucm083378.htm)

26. International Serious Adverse Event Consortium. (Accessed at: www.saeconsortium.org)

27. Predictive Safety Testing Consortium. (Accessed at: www.c-path.org/pstc.cfm)

28. Nuremberg code. (Accessed at: http://ohsr.od.nih.gov/guidelines/nuremberg.html)

29. Declaration of Helsinki. (Accessed at: http://ohsr.od.nih.gov/guidelines/helsinki.html)

30. Belmont report. (Accessed at: http://ohsr.od.nih.gov/guidelines/belmont.html)

31. ICH E6(R1) – Guideline for Good Clinical Practice. June 1996. (Accessed at: www.ich.org/LOB/media/MEDIA482.pdf)

32. Barnes M, Heffernan K. The "Future Uses" Dilemma: Secondary Uses of Data and Materials by Researchers for Commercial Research Sponsors. Medical Research Law & Policy, 2004; 3: 440-450.

33. Eriksson S, Helgesson G. Potential harms, anonymization, and the right to withdraw consent to biobank research. Eur J Hum Genet., 2005; 13:1071-1076. (Accessed at: www.nature.com/ejhg/journal/v13/n9/pdf/5201458a.pdf)

34. Renegar G, Webster CJ, Stuerzebecher S, et al. Returning genetic research results to individuals: points-to-consider. Bioethics 2006; 20: 24-36. (Accessed at: http://www3.interscience.wiley.com/cgi-bin/fulltext/118562753/PDFSTART)

35. Article 29 Data Protection Working Party. (Accessed at: www.ec.europa.eu/justice_home/fsj/privacy/workinggroup/index_en.htm)

36. Human Tissue Act 2004 (UK). (Accessed at: www.opsi.gov.uk/acts/acts2004/en/ukpgaen_20040030_en_1)

37. Genetic Information Nondiscrimination Act. (Accessed at: http://frwebgate.access.gpo.gov/cgi-bin/getdoc.cgi?dbname=110_cong_public_laws&docid=f:publ233.110.pdf)

38. Guidance for Sponsors, Clinical Investigators, and IRBs Data Retention When Subjects Withdraw from FDA-Regulated Clinical Trials. FDA October 2008 www.fda.gov/OHRMS/DOCKETS/98fr/FDA-2008-D-0576-gdl.pdf

39. Anderson C, Gomez-Mancilla B, Spear BB, Barnes DM, Cheeseman K, Shaw P, Friedman J, McCarthy A, Brazell C, Ray SC, McHale D, Hashimoto L, Sandbrink R, Watson ML, Salerno RA, on behalf of The Pharmacogenetics Working Group. Elements of Informed Consent for Pharmacogenetic Research; Perspective of the Pharmacogenetics Working Group. Pharmacogenomics Journal 2002;2:284-92. (Accessed at: www.nature.com/tpj/journal/v2/n5/abs/6500131a.html)

I-PWG

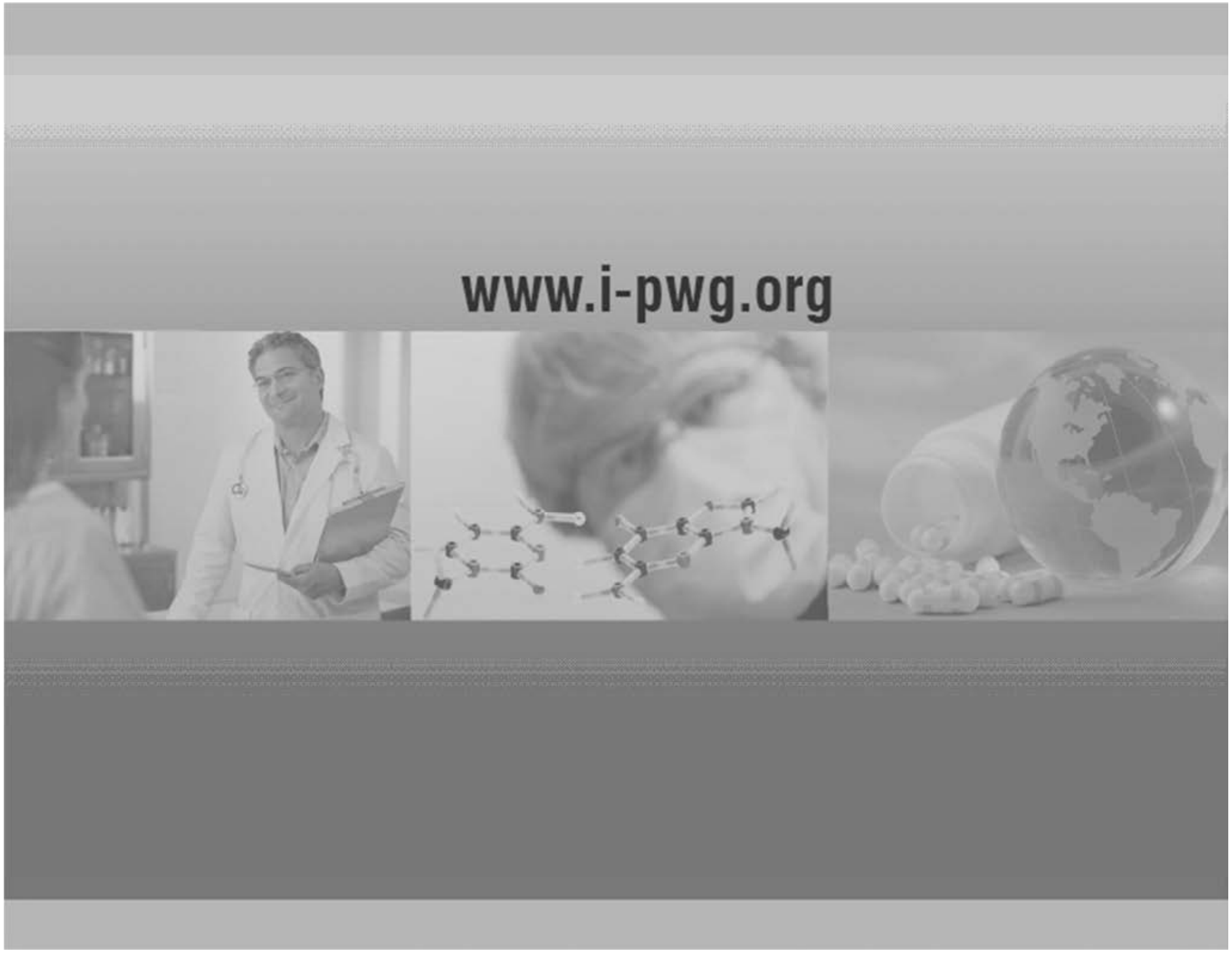
www.i-pwg.org

12.4 Definition of CMV Disease in Hematopoietic Stem Cell Transplant (HSCT) Recipients

CMV Disease	Diagnostic Criteria	Notes
Pneumonia	Signs and/or symptoms of pulmonary disease<br>**AND**<br>detection of CMV in BAL or tissue samples (virus isolation, histopathology, immunohistochemical analysis or *in situ* hybridization).	• PCR may be too sensitive, so detection of CMV by PCR alone is insufficient for the diagnosis of CMV pneumonia.<br>• Detection of fungal copathogens like *Aspergillus spp*. + "halo" sign (radiology) indicates fungal, rather than CMV pneumonia.<br>• Superinfection or coinfection with other pathogens may occur and should be noted when present.
Gastrointestinal (GI) Disease	Symptoms from upper or lower GI tract<br>**AND**<br>macroscopic endoscopic findings on endoscopy<br>**AND**<br>detection of CMV virus (isolation, histopathology, immunohistochemical analysis or *in situ* hybridization) in a GI biopsy specimen.	• Detection of CMV by PCR alone is insufficient for the diagnosis of CMV GI disease.
Hepatitis	Increased bilirubin and/or increased enzymes<br>**AND**<br>no other documented cause of hepatitis<br>**AND**<br>detection of CMV infection (culture, histopathology, immunohistochemical analysis or *in situ* hybridization) in a liver biopsy specimen.	• Detection of CMV by PCR alone is insufficient as it may represent transient viremia. Hence, PCR is insufficient to diagnose CMV hepatitis.<br>• Documentation of CMV in liver biopsy specimen (i.e., by culture, histopathology, immunohistochemical analysis or *in situ* hybridization) is needed.<br>• Coinfection with other pathogens like HCV may be present without excluding the diagnosis of CMV hepatitis.

CMV Disease	Diagnostic Criteria	Notes
Central Nervous System (CNS) disease	CNS symptoms<br>**AND**<br>detection of CMV in CSF samples (culture or PCR) OR in a brain biopsy specimen (culture, histopathology, immunohistochemical analysis or *in situ* hybridization).	N/A
Retinitis	Lesions typical of CMV retinitis must be confirmed by an ophthalmologist.	N/A
Nephritis	CMV infection (culture, immunohistochemical analysis or *in situ* hybridization)<br>**AND**<br>histologic features of CMV infection in a renal biopsy specimen in a patient with renal dysfunction.	• Detection of CMV by PCR alone is insufficient for the diagnosis of CMV nephritis.<br>• Furthermore, detection of CMV in urine of patient with renal dysfunction does not fulfill the definition of CMV nephritis.
Cystitis	CMV infection (culture, immunohistochemical analysis or *in situ* hybridization)<br>**AND**<br>histologic features of CMV infection in bladder biopsy specimen in a patient with cystitis.	• Detection of CMV by PCR alone is insufficient for the diagnosis of CMV cystitis.<br>• Furthermore, detection of CMV in urine of patient with symptoms does not fulfill the definition of CMV cystitis.
Myocarditis	CMV infection (culture, immunohistochemical analysis or *in situ* hybridization)<br>**AND**<br>histologic features of CMV infection in heart biopsy specimen in a patient with myocarditis.	• Detection of CMV by PCR alone is insufficient for diagnosis of CMV myocarditis.
Pancreatitis	CMV infection (culture, immunohistochemical analysis or *in situ* hybridization)<br>**AND**<br>histologic features of CMV infection in pancreatitis biopsy	• Detection of CMV by PCR alone is insufficient for the diagnosis of CMV pancreatitis.

CMV Disease	Diagnostic Criteria	Notes
	specimen in a patient with pancreatitis.	
Other disease categories	Compatible signs and symptoms **AND** documentation of CMV by biopsy.	• Detection of CMV by PCR alone is insufficient.
Reference [1]<br>BAL = bronchoalveolar lavage; CMV = cytomegalovirus; CNS = central nervous system; CSF = cerebrospinal fluid; GI = gastrointestinal; HCV = hepatitis C virus; PCR = polymerase chain reaction.		

12.5 Child-Pugh Classification for Severity of Liver Disease

	**Scoring by Anomaly**		
**Signs or symptom**	**1 point**	**2 points**	**3 points**
Hepatic encephalopathy[1]	absent	Grade 1 or Grade 2	Grade 3 or Grade 4
Ascites	absent	mild	moderate
Bilirubin (μmol/L)	< 2 mg/dL	2 – 3 mg/dL	> 3 mg/dL
Albumin (g/dL)	> 3.5 g/dL	2.8 – 3.5 g/dL	< 2.8 g/dL
Prothrombin time (INR)	< 1.7	1.7 – 2.3	> 2.3

[1]Hepatic encephalopathy grading:
Grade 1: Altered mood/confusion
Grade 2: Inappropriate behavior, impending stupor, somnolence
Grade 3: Markedly confused, stuporous but arousable
Grade 4: Comatose/unresponsive

**Child Pugh Score Interpretation**	
5 – 6 points	Child-Pugh stage A (mild hepatic insufficiency)
7 – 9 points	Child-Pugh stage B (moderate hepatic insufficiency*)
>10 points	Child-Pugh stage C (severe hepatic insufficiency)

*If hypoalbuminemia is the only abnormality noted, the subject will need to have a score of ≥7 to qualify for moderate hepatic insufficiency for this study.

12.6 Clinical Experience with IV Formulation

AIC246-01-I-13 'A single centre, 3-part trial to investigate (A) the safety, tolerability and pharmacokinetics of multiple ascending oral doses and (B) multiple intravenous doses of letermovir, and (C) the effect of letermovir under steady state conditions on the pharmacokinetics of a single oral dose of digoxin, a sensitive P-glycoprotein substrate'

This clinical trial was conducted in 3 parts.

Part A - the safety, tolerability and pharmacokinetics of single and multiple ascending oral doses of 240 mg bid (twice daily), 360 mg bid and 360 mg qd (once daily) of letermovir

Study design

Twelve healthy female subjects received twice daily (bid) oral dosing of 240 mg letermovir from Day 1 to Day 6 in an open-label design. PK samples were taken over 1 dosing interval to 12 hours post-dose on Day 6. Data of this dosing group are also part of Part C of trial AIC246-01-I-13.

Single and multiple ascending doses of 360 mg bid and 360 mg qd were administered to healthy female subjects using a randomized, double blind, placebo-controlled design. In both groups, 10 subjects (8 on letermovir; 2 on placebo) received a single 360 mg or placebo dose on Day 1, with PK profiling to Day 4 (72 hours) followed by either qd or bid dosing of 360 mg or placebo from Day 4 to Day 9. Placebo subjects were pooled to one group consisting of 4 subjects.

Safety

Overall, oral 240 mg bid, 360 mg bid and 360 mg qd administration of letermovir was safe and well tolerated. There were no serious adverse events (SAEs) and no withdrawals due to AEs or any other reasons. No dose limiting or severe AEs were reported. AEs were reported at each dose level and no trend in incidence or nature of AEs was seen when comparing placebo to 240 mg bid, 360 mg bid and 360 mg qd letermovir dosing. There were no clinically significant changes in clinical laboratory tests, physical examinations, vital signs, Holter ECGs, telemetry and 12-lead ECG parameters. There were no effects on QTcF up to the highest letermovir dose of 360 mg bid.

In the 360 mg bid group 23 treatment emergent adverse events (TEAEs) in 6 of 8 (75%) subjects were reported, 14 were of mild and 9 of moderate intensity (headache, migraine, abdominal pain and nausea). In the 360 mg qd group 28 TEAEs were reported in 7 of 8 (87.5%) subjects, 26 were of mild intensity and 2 of moderate intensity (catheter site pain and dizziness) and in the 240 mg bid (daily dose 480 mg) group 6 of 12 [50%] subjects reported 9 TEAEs which were all of mild intensity. In the placebo group, 14 TEAEs in 3 of

4 (75%) subjects were reported of which 7 were of mild and 7 of moderate intensity (dyspepsia and catheter site pain). Overall, the most frequently occurring TEAE was headache, reported in 8 of 28 (28.6%) letermovir treated subjects.

The most common TEAEs judged by the investigator to be related to letermovir were headache (4 (50%) subjects on 360 mg qd; 3 (25%) subjects on 240 mg bid [daily dose 480 mg] , 1 (25%) on placebo), nausea (3 (37.5%) subjects on 360 mg qd; 2 (25%) on 360 mg bid [daily dose 720 mg]; 1 (25%) on placebo), and dizziness (3 (37.5%) subjects on 360 mg qd; 1 (12.5%) subjects on 360 mg bid [daily dose 720 mg]).

Pharmacokinetic Results

After oral dosing of letermovir at 240 mg bid, 360 mg bid and 360 mg qd, letermovir daily steady-state exposure (AUC0-24h,ss; for bid regimens calculated by taking 2*AUC12h,ss) showed a tendency towards a more than dose proportional increase with increasing dose from 360 mg (qd) to 720 mg (360 mg bid). No apparent deviation from dose proportionality was observed for Cmax. Mean apparent clearance (CL/F) at steady-state decreased with increasing daily dose. Mean apparent terminal elimination half-life (t1/2z) values were similar (t1/2z was not available for 240 mg bid dose regimen). Steady-state conditions had generally been reached by Day 6/7. Accumulation to steady-state exposure was modest for the 360 mg bid dosing regimen, with mean RA(AUC) and RA(Cmax) values on Day 6 /Day 10 of 1.9 and 1.5 respectively. For the 360 mg qd dosing regimen no accumulation was observed, with mean values for RA(AUC) and RA(Cmax) on Day 6 /Day 10 of 1.1 and 0.9, respectively.

Part B - single and multiple IV dosing of 120 and 240 mg arginine phosphate buffered letermovir

Twenty four (24) healthy female subjects, 12 per dosing cohort, received daily infusions of arginine phosphate buffered IV letermovir (0.9% arginine phosphate buffered saline) or placebo (0.9% saline) on Day 1 of Period 1 and Day 1 to Day 7 of Period 2, randomized 8:4.

Safety

Single and multiple daily doses of 120 mg and 240 mg of arginine phosphate buffered letermovir given by peripheral IV infusion over 60 minutes were systemically but not locally well tolerated. There were no deaths or severe AEs. One SAE of unrelated potential deep vein thrombosis of the brachial vein on the non-infusion arm occurred in a subject dosed with 120 mg letermovir. Sixty-three (63) TEAEs were reported in 8 of 8 [100%] subjects in the 120 mg IV letermovir treatment group, 38 TEAEs in 7 of 8 (87.5%) subjects in the 240 mg IV letermovir treatment group, and 21 TEAEs in 6 of 8 [75%] subjects in the placebo treatment group. TEAEs of moderate intensity consisted of infusion site pain (2 subjects in the 120 mg letermovir group and 1 subject in the 240 mg letermovir group); infusion site

thrombosis (1 subject in the 240 mg letermovir group); and migraine (1 subject in the placebo group). Three subjects discontinued the trial due to AEs: one subject after the first dose of 120 mg letermovir due to an SAE and two due to possibly related infusion site thrombophlebitis after multiple dose treatment with 240 mg letermovir qd. The most common drug-related TEAEs were infusion site pain (4 (50%) subjects in 120 mg IV, 6 (75%) subjects in 240 mg IV and none in the placebo group), and infusion site thrombosis (5 (62.5%) subjects in 120 mg IV, 5 (62.5%) subjects in 240 mg IV and 1 (12.5%) in the placebo group). There were no clinically significant changes in clinical laboratory tests, physical examinations, vital signs, Holter ECGs, telemetry and 12-lead ECG parameters.

Pharmacokinetic Results:

The PK results demonstrated that after single IV administration and once daily infusions of 120 mg and 240 mg doses, respectively, letermovir exposure (AUC0-last, AUC0-∞ and Cmax after single dose and AUCτ,ss and Cmax after repeated dosing) did not deviate statistically significantly from dose proportionality. Mean apparent clearance (CL) and terminal elimination half-life (t1/2z) values were similar for both dose levels, both after single dose and after repeated dosing. Based on the letermovir pre-dose levels on Day 6, 7 and 8 (Day 7, 24 hour), steady-state conditions may not entirely have been reached by Day 7. The levels (arithmetic mean values) on Day 7 (272.4 ng/mL) and Day 8 (257.8 ng/mL) tended to be somewhat lower compared to Day 6 (329.4 ng/mL). Accumulation after once daily dosing for 7 days was small and similar for both dose levels with mean values for accumulation ratio calculated from AUC, RA(AUC) of 1.33 for the 120 mg dose level and 1.23 for the 240 mg dose level. Accumulation ratio RA(Cmax) during once daily dosing for 7 days was also small and similar for both dose levels with mean values for RA(Cmax) of 1.05 for the 120 mg dose level and 1.08 for the 240 mg dose level.

Part C – drug-drug interaction with digoxin

In an open-label, 2-period, 2-sequence, cross-over design, 24 female healthy volunteers received a single oral dose of 0.5 mg digoxin in one period and 240 mg letermovir twice daily for 12 days with on the 7th day of letermovir treatment concomitant dosing of a single dose of 0.5 mg digoxin in the other Period. Two subjects were withdrawn, 1 subject upon a positive test for cannabinoids and 1 subject upon a positive pregnancy test (pregnancy was terminated and the termination was unrelated to trial treatment).

Safety

Coadministration of a single oral dose of 0.5 mg digoxin to steady-state oral letermovir 240 mg bid was safe and well tolerated by the healthy subjects participating in this study. There were no serious adverse events (SAEs) and no withdrawals due to adverse events.

Safety analysis was conducted for all of the three dosing periods i.e., letermovir 240 mg bid dosing, letermovir 240 mg bid coadministered with a single dose of 0.5 mg digoxin, and single dose of 0.5 mg digoxin. Sixteen (16) TEAEs were reported in 10 (41.7%) subjects for the letermovir 240 mg bid treatment regimen. All TEAEs were of moderate (2) or mild (14) intensity. The TEAEs of moderate intensity included nausea (1) and dizziness (1). The most common TEAEs were headache and nausea. Fourteen (14) TEAEs in 8 (33.3%) subjects were considered probably or possibly treatment related, backpain and headache, reported in 3 subjects each, were most common.

Twenty-seven (27) TEAEs were reported in 12 (50.0%) subjects for the letermovir 240 mg bid + digoxin 0.5 mg treatment regimen. All TEAEs were of moderate (5) or mild (22) intensity. The TEAEs of moderate intensity included nausea (1), headache (2), somnolence (1), and ear pain (1). The most common TEAEs were headache, diarrhoea, and nausea. Nineteen (19) TEAEs in 9 (37.5%) subjects were considered probably or possibly treatment related, headache (4 subjects) and diarrhoea (3 subjects) were most common.

Twelve (12) TEAEs were reported for 8 (36.4%) subjects when subjects were dosed with digoxin alone, all were of mild intensity except for one moderate event of vomiting. Three subjects (13.6%) reported probably or possibly related TEAEs of abdominal pain, diarrhoea and headache.

There were no clinically significant changes in biochemistry, hematology, coagulation and urinalysis parameters during the trial, vital signs or ECG.

Pharmacokinetic Results:

Results of the effect of letermovir under steady state conditions on plasma levels of digoxin after a single oral dose of digoxin are summarized in the table below. Letermovir coadministration decreased the geometric mean AUC0-last and Cmax of diogoxin by 12% and 23%, respectively (90% CI 79.96 - 96.21 %). Steady-state conditions were reached within 6 days of dosing for letermovir at 240 mg bid.

Summary of the statistical evaluation of the effect of letermovir on digoxin pharmacokinetics (based on paired data)

Parameter	LS Geometric means[a]		GMR, %	90% CI, %[b]	p-value	
	Single dose of 0.5 mg digoxin (reference)	Single dose of 0.5 mg digoxin + 240 mg letermovir bid (test)			Period	Sequence
C_{max}, ng/ml	2.860	2.145	77.00	63.35 - 88.79	0.5456	0.9247
$AUC_{0\text{-}last}$, ng.h/mL	36.21	31.72	87.60	79.96 - 96.21	0.2337	0.8013
a n=22 for reference and test<br>b 90% confidence intervals GMR (Geometric Mean Ratio)						

Source data table 2A: Clinical Pharmacokinetic Final Report (CD110044, version 2.0, 26Nov2012)

AIC246-01-I-14 'A single-center, 2-part trial to investigate the safety, tolerability, and pharmacokinetics of (A) single ascending intravenous doses and (B) multiple intravenous doses of a hydroxypropyl beta ß-cyclodextrin (HPßCD) intravenous formulation of letermovir'

This randomized, double-blind, placebo-controlled trial consisted of two parts. In Part A, placebo or single ascending doses of 120 mg, 240 mg (30 min infusion), 480 mg, 720 mg, and 960 mg (60 min infusion) of a hydroxypropyl ß-cyclodextrin (HPßCD) based formulation of letermovir were administered via the IV route to healthy female subjects (6 subjects on letermovir, 2 on placebo in each group). In Part B, an IV dose of 240 mg (8 subjects) or placebo (4 subjects) was administered on Day 1 and once daily on Days 8 to 14. Pharmacokinetic samples were collected after all single dose administrations (Part A and Part B Day 1) and at steady-state (Part B, Day 14).

Safety (preliminary results)

Intravenous infusion of letermovir as single doses up to 960 mg and multiple doses of 240 mg once daily for 7 days of the hydroxylpropyl-β-cyclodextrin formulation of letermovir was generally safe and well tolerated in the population studied. One SAE of breast cancer starting ~ 2 months after the follow-up visit, was reported for a placebo-treated subject. No subjects were withdrawn due to a TEAE.

Forty-five (45) TEAEs were reported in 18/30 (60%) letermovir treated subjects of trial Part A and 15 TEAEs in 6/8 (75%) letermovir treated subjects of trial Part B. In both trial parts, 50% of placebo-treated subjects experienced AEs (5/10 in Part A and 2/4 in Part B). The majority of AEs were of mild intensity. Five probably related severe AEs were reported for one subject following administration of single dose 960 mg letermovir (nausea and 4 events of vomiting). Four AEs of moderate intensity were noted i.e., unlikely related diarrhoea after

single dose 120 mg letermovir; possibly related headache after single dose 480 mg letermovir; possibly related vomiting (2 events) after single dose 480 mg letermovir; possibly related headache after single dose placebo.

In Part A, 17 TEAEs in 8/30 (26.7%) letermovir-treated subjects were possibly or probably IMP-related; TEAEs occurring in more than one subject were headache (2 subjects in 480 mg and 1 subject in placebo group), vomiting (1 subject in 480 mg and 960 mg group each), and nausea (1 subject in 120 mg, 960 mg and placebo group each). In Part B, 8 TEAEs in 5/8 (62.5%) subjects were assessed as possibly drug related, of which headache and dizziness occurred in more than one letermovir-treated subject (2 subjects each).

Local tolerance at the IV infusion site was evaluated by regular assessments of ultrasound of the arm veins, visual analogue scale (VAS; maximum possible value = 100 mm) for assessment of pain at the infusion site, and a visual infusion phlebitis (VIP) score. All VIP score ≥2 (i.e., two of the following 'pain at IV site', 'erythema,' and/or 'swelling,') were required to be recorded as an adverse event "infusion site reaction". In trial Part A, overall, VAS score values were very small as compared to the size of the VAS (maximum possible value = 100 mm) for letermovir-treated subjects (maximum mean (SD) increases ranging from 1.17 (2.40) mm after SD 120 mg letermovir, 0.67 (1.63) after SD 960 mg letermovir and 0.50 (1.08) mm in placebo-treated subjects) and a VIP score of 2 was recorded for one subject following administration of 120 mg letermovir at 4 h after start of infusion as part of a mild, possibly-related infusion site reaction described as 'swelling, pain, tenderness' starting 3 hours and 14 min after the start of IV dosing with a duration of almost 4 days (normal ultrasound, VAS score was <2 at all timepoints except at 4 and 6 hr after start of infusion). In addition, infusion site reactions were reported for two other subjects after single letermovir dosing: one mild, probably-related infusion site reaction (dragging pain in the infusion arm 1 centimeter above the injection site to the shoulder) was reported starting 15 min after 120 mg letermovir IV dosing with a duration of 20 minutes (VIP score was zero at all timepoints; no clinical findings at ultrasound examination, VAS score was <2 at all timepoints except at pre-dose) and one mild, unlikely related AE was described as 'palpable, hardened venous cords in the cubitus on both sides' (preferred term at the infusion site: Infusion site reaction; preferred term at the PK site: Vessel puncture site reaction) starting almost 9 days after dosing with 480 mg letermovir, which was not yet resolved at the end of the trial (VIP and VAS score zero at all timepoints, all ultrasound findings were normal except at follow-up 18 days and 30 days after dosing [not clinically significant indurations of both arms in the cubital fossa]).

In Part B, VAS scores were also low, VIP scores were 0 throughout the study except for one subject with a score of 1. One subject dosed with multiple IV doses of 240 mg letermovir once daily for 7 days, had two reports of infusion site reaction. Firstly, a mild possibly-related AE described as 'infusion reaction (redness along the path of the cephalia vein left)'

(preferred term infusion erythema) was reported starting about 3 hours after dosing of the 6th dose in the multiple dose part of the trial. The duration of this AE was 4 days. Secondly, a mild possibly-related AE described as ‘infusion reaction (palpable venous courd in a length of 3 cm at venipuncture site along the path of the cephalica vein left)’ was reported starting 15 days post first dosing (preferred term infusion site reaction). A VIP score of 1 was documented from Day 13 to follow-up examination, the VAS score was zero at all timepoints. Ultrasound of arm veins did not reveal clinically significant findings in Part A or Part B. No possibly letermovir related clinically significant changes in laboratory parameters, vital signs or ECGs were observed.

Pharmacokinetics results (preliminary results)

Letermovir exposure (AUC0–last and AUC0–∞) showed a tendency towards more than dose proportional increase when subjects were treated with escalating single intravenous letermovir doses of 120 to 960 mg. Mean clearance decreased with increasing doses. No apparent deviation from dose proportionality was observed for Cmax (infusion duration 30 min for the 120 and 240 mg, 60 min for the 480 to 960 mg doses). Individual dose normalized Cmax values tended to be slightly higher after 30 min infusions as compared to 60 min infusions, however, differences are modest.

After once daily administration of 240 mg for 7 days accumulation to steady-state exposure was modest, with mean RA(AUC) = 1.22 and mean RA(Cmax) = 1.03 on Day 14 (ie, the 7th day of letermovir administration). Steady-state conditions had been reached within 7 days of once daily letermovir administration as shown by C0h values. Mean apparent terminal elimination half-life (t1/2z) increased upon daily administration, however, variability in this parameter was high. Therefore, the observed differences have to be interpreted with caution. No consistent time dependency in letermovir exposure was observed.

AIC246-01-I-16 - renal impairment trial

Oral doses of 120 mg letermovir once daily for 8 days were given to 24 male and female adult subjects i.e. 8 healthy subjects, 8 moderately renally impaired subjects (eGFR ≥30 to 59 mL/min/1.73m2), and 8 severely renally impaired adult subjects (eGFR <30 mL/min/1.73m2) in an open-label, single-center, non-randomized trial. Safety and pharmacokinetics were evaluated.

Safety (preliminary results)

Multiple doses of 120 mg letermovir were safe and well tolerated by the healthy, moderately renally impaired and severely renally impaired subjects in this trial. No deaths, SAEs or severe AEs were reported. A total of 3 TEAEs were reported for 2 subjects (1 moderate, 1 severe renal impairment). No subject discontinued to TEAEs, and no TEAEs were considered to be letermovir related. While clear baseline differences in safety laboratory

parameters were evident between the subject groups, these were consistent with the differences in clinical status between healthy subjects, and subjects with moderate or severe renal impairment. No trends in changes from baseline in safety laboratory parameters, vital signs, ECG, or physical examination findings were apparent during the trial for any subject group.

Pharmacokinetics (preliminary results):

Pharmacokinetics of Letermovir (arithmetic mean ± SD, t_{max}: median [range])	Normal renal function (reference)	Moderate renal impairment (test 1)	Severe renal impairment (test 2)
n	8	8	8
**Day 5**			
C_{0h}, ng/mL	91.64 ± 35.52	290.9 ± 171.0	235.2 ± 238.6
**Day 6**			
C_{0h}, ng/mL	80.13 ± 36.99	291.5 ± 155.6	284.7 ± 321.1
**Day 7**			
C_{0h}, ng/mL	80.15 ± 27.82	263.2 ± 175.2	309.4 ± 352.9
**Day 8**			
C_{0h}, ng/mL	82.86 ± 34.26	278.5 ± 165.9	274.9 ± 296.5
C_{min}, ng/mL	80.78 ± 32.77	271.5 ± 161.7	264.7 ± 285.7
$C_{ss,max}$, ng/mL	2614 ± 1042	3301 ± 1670	2714 ± 929.9
t_{max}, h	1.50 (1.00 - 2.50)	1.51 (1.00 - 2.00)	1.75 (1.00 - 4.00)
$AUC_{\tau,ss}$, ng.h/mL	11413 ± 3194	22694 ± 9944	21013 ± 17919
$C_{ss,av}$, ng/mL	475.5 ± 133.1	945.6 ± 414.3	875.4 ± 746.7
FI, %	521.8 ± 116.5	331.6 ± 117.7	410.2 ± 197.2
λ_z, 1/h	0.05462 ± 0.03054	0.03369 ± 0.01447	0.03940 ± 0.02172
$t_{1/2z}$, h	16.21 ± 7.705	25.95 ± 15.83	21.69 ± 9.295
CL/F, L/h	11.25 ± 3.090	6.024 ± 2.119	9.929 ± 7.124
V_z/F, L	261.4 ± 148.2	222.8 ± 142.4	314.3 ± 308.9
**LSmeans ratio (90% CI), %**			
		**test 1 vs reference**	**test 2 vs reference**
n		8 vs 8	8 vs 8
**Day 8**			
$C_{ss,max}$		125.33 (86.54 - 181.50)	106.11 (74.81 - 150.50)
$AUC_{\tau,ss}$		191.79 (142.58 - 257.98)	142.02 (83.10 - 242.71)

Total letermovir $AUC_{\tau,ss}$ was 1.92 and 1.42-fold higher for subjects with moderate and severe renal impairment, respectively, as compared to healthy subjects. Total $C_{ss,max}$ was 1.25 fold higher in subjects with moderate renal impairment compared to healthy subjects, while $C_{ss,max}$ was comparable in healthy subjects and subjects with severe renal impairment (GMR 106%).

The fraction unbound of letermovir was slightly higher for subjects with renal impairment as compared to healthy subjects, with higher values for subjects with severe renal impairment, compared to subjects with moderate renal impairment. Unbound letermovir $AUC_{\tau,ss}$ was 2.15 and 1.81-fold higher for subjects with moderate and severe renal impairment, respectively, as compared to healthy subjects. Unbound $C_{ss,max}$ was 1.41 and 1.35-fold higher, respectively, than in healthy subjects.

The 90% CIs of the abovementioned ratios were generally wide, particularly for the severe renal impairment versus normal renal function comparison for $AUC_{\tau,ss}$ (due to a relatively high between subject variability in $AUC_{\tau,ss}$ in the severe renal impairment group). Therefore, the extent of the observed differences has to be interpreted with caution.

12.7 Approximate Blood/Tissue Volumes Drawn/Collected by Trial Visit and by Sample Types

Trial Visit:	SCR	D1	D7	W2	W3	W4	W5	W6	W7	W8	W9	W10[a]	W11[a]	W12[a]	W13[a]	End of Study Therapy W14[a]	W16	W18, W20, W22	W24	W32, W40, W48	CMV Infection or Early Discon Visit
Blood Parameter	Approximate Blood Volume (mL)																				
Chemistry/Hematology[b]	5.5	5.5		5.5		5.5				5.5				5.5		5.5	5.5				5.5
Serum β-Human Chorionic Gonadotropin (β-hCG)[c]	3.5																				
Serum inhibin B, LH, FSH, testosterone in men		11														11			11		11
HIV/Hepatitis B, C Screen[d]	20																				
Blood for Future Biomedical Research		8.5																			
CMV DNA PCR	6-30[e]	6	6	6	6	6	6	6	6	6	6	6	6	6	6	6	6	6 (per visit)	6	6 (per visit)	6
CMV DNA Sequence Analysis[f]																					8
Population PK			5	5		5		5		5		5		5		5					
Intensive PK (for subset of subjects only)			25																		
Expected Total (mL)	35-59	31	36	16.5	6	16.5	6	11	6	16.5	6	11	6	16.5	6	27.5	11.5	6 (per visit)	17	6 (per visit)	30.5

a End of Study Therapy Visit may occur on Week 10, 11, 12, 13, or 14 depending on when study therapy was started during the 28-day post-transplant window Therefore, not all visits will be performed in all subjects

b For screening, values from the subject's chart for required chemistry and hematology tests are acceptable If not available, this testing should be performed by the central laboratory

c For female subjects of child bearing potential only

d Perform HIV/Hepatitis B, C Screen only if not previously documented within the last 90 days If hepatitis C virus antibody is positive, RNA PCR results should be provided (or, if not available, RNA PCR testing will be performed by the central laboratory)

e For initial screening, results of CMV DNA PCR assay performed at a local laboratory will be acceptable From the time of transplantation until randomization, CMV DNA PCR testing will be performed once a week by the central laboratory

f CMV DNA Sequence Analysis to be performed only in subjects with clinically significant CMV infection.

13.0 SIGNATURES

13.1 Sponsor's Representative

TYPED NAME	SIGNATURE	DATE
______________________	________________________	_________

13.2 Investigator

I agree to conduct this clinical trial in accordance with the design outlined in this protocol and to abide by all provisions of this protocol (including other manuals and documents referenced from this protocol). I agree to conduct the trial in accordance with generally accepted standards of Good Clinical Practice. I also agree to report all information or data in accordance with the protocol and, in particular, I agree to report any serious adverse events as defined in Section 7.0 – Assessing and Recording Adverse Events. I also agree to handle all clinical supplies provided by the Sponsor and collect and handle all clinical specimens in accordance with the protocol. I understand that information that identifies me will be used and disclosed as described in the protocol, and that such information may be transferred to countries that do not have laws protecting such information. Since the information in this protocol and the referenced Investigator's Brochure is confidential, I understand that its disclosure to any third parties, other than those involved in approval, supervision, or conduct of the trial is prohibited. I will ensure that the necessary precautions are taken to protect such information from loss, inadvertent disclosure or access by third parties.

TYPED NAME	SIGNATURE	DATE
______________________	________________________	_________

SUMMARY OF CHANGES

PRIMARY REASON(S) FOR THIS AMENDMENT:

Section Number (s)	Section Title(s)	Description of Change (s)
2.1; 5.2; 6.0; 7.1.5.1	Trial Design; Trial Treatments; Trial Flow Chart; Screening	The collection of plasma for testing for CMV viremia, creatinine clearance, and liver function testing was changed from 7 days to 5 days prior to randomization. These changes were made based on an agency request.
2.1; 4.2.3.1; 5.8; 6.0; 7.1.3.3; 7.1.5.4	Trial Design; Efficacy Endpoints; Subject Withdrawal/Discontinuation Criteria; Trial Flow Chart; CMV DNA PCR Testing; CMV Infection or Early Discontinuation Visit	A clarification was provided noting that the plasma sample for CMV DNA PCR testing is to be a <u>confirmatory</u> sample as follows:<br>• A <u>confirmatory</u> plasma sample for CMV PCR testing at the central laboratory should be collected for subjects who develop clinically significant CMV infection during the study treatment period (up to Week 14 post-transplant) and the post-treatment [follow-up] period (after Week 14 and through Week 24 post-transplant).<br>• It is mandatory to send a <u>confirmatory</u> plasma sample for CMV DNA PCR testing to the central laboratory **<u>immediately prior to</u>** (i.e., on the day of) initiating treatment for CMV disease or PET in **<u>ALL</u>** instances. In addition, a second plasma sample must be collected 48-72 hours after initiating anti-CMV therapy for CMV DNA PCR testing at the central laboratory.<br>• While any detectable CMV viral DNA results in the Roche CAP/CTM assay from <u>confirmatory plasma sample</u> sent to the central laboratory is acceptable for the purpose of documenting viremia as a component of the primary endpoint, it is strongly recommended that investigators should not initiate PET when CMV viral load is below the LLoQ, but detectable (<LLoQ, detected).<br>These changes were made based on agency requests.
4.2.3.1; 8.2.3.1.1	Efficacy Endpoints; Efficacy/Pharmacokinetic Endpoints	Documented viremia is defined as any detectable CMV viral DNA on a confirmatory sample obtained immediately prior to (i.e., on the day of) the initiation of treatment for CMV disease or PET, as measured by the Roche COBAS® AmpliPrep/COBAS

		TaqMan® (CAP/CTM) System in the central laboratory. In the event that the confirmatory result is not available, a subsequent central laboratory result collected from a sample obtained within a 7-day window will be used. This change was made based on an agency request.
4.2.3.1	Efficacy Endpoints	The guidance regarding viral load threshold for initiation of PET was revised to the following:<br><br>During the study treatment period [through Week 14 (~100 days) post-transplant]<br>• High risk: viral DNA ≥150 copies/mL<br>• Low risk: viral DNA >300 copies/mL<br><br>After Week 14 (~100 days) post-transplant<br>• High risk: viral DNA >300 copies/mL<br>• Low risk: viral DNA >300 copies/mL<br><br>This change was made based on an agency request.

ADDITIONAL CHANGE(S) FOR THIS AMENDMENT:

**Section Number (s)**	**Section Title(s)**	**Description of Change (s)**
2.1	Trial Design	A clarification was added noting that although for initial screening purposes, CMV DNA PCR results of an assay done at a local laboratory will be acceptable, CMV DNA PCR testing must be conducted once a week by the central laboratory from the time of transplantation until randomization.
2.1; 5.8; 7.1.5.4	Trial Design; Subject Withdrawal/Discontinuation Visit; CMV Infection or Early Discontinuation Visit	A clarification was provided noting that subjects will complete remaining study visits (including all subsequent treatment period visits) if they discontinue from study therapy due to clinically significant CMV infection.

5.1.2	Subject Inclusion Criteria	An inclusion criterion was added where a subject will have undetectable CMV DNA (as confirmed by the central laboratory) from a plasma sample collected within 5 days prior to randomization.
5.1.3; 6.0; 7.1.2.5	Subject Exclusion Criteria; Trial Flow Chart; Child Pugh Score	A 5-day window for laboratory tests was added in Exclusion Criteria 7, 8, and 9.
5.1.3	Subject Exclusion Criteria	A clarification was provided noting that a repeat test for AST, ALT, and serum bilirubin should occur prior to randomization.
5.5	Concomitant Medications/Vaccinations (allowed & prohibited)	Clarifications were provided noting the following:<br>• Medications/therapies listed in the section pertain to coadministration with MK-8228. When used, these agents should be administered in a manner consistent with the local product circular for these agents and dose adjustments are not required due to administration of study therapy.<br>• All drug-drug interactions for co-administered immunosuppressant agents, as per the local prescribing information for these agents, are also applicable in this study; therefore it is also important for the investigator to monitor the use of any other co-administered immunosuppressants and ensure that these therapies are administered according to local product circulars.<br>These changes were made based on an agency request.
5.5	Concomitant Medications/Vaccinations (allowed & prohibited)	A clarification was provided noting that medications are prohibited while subjects are on study therapy.
6.0; 7.1.5.4	Trial Flow Chart; CMV Infection or Early Discontinuation Visit	A clarification was provided noting that at a CMV Infection Visit, a confirmatory plasma sample for CMV PCR testing at the central laboratory should be collected. This change was made to be consistent with other changes in the protocol.
6.0	Trial Flow Chart	A clarification was provided noting that all identified subjects who choose to participate in intensive PK sampling will sign an additional informed consent prior to the collection of samples.
6.0; 7.1.2.7	Trial Flow Chart; Adverse Events Monitoring	The monitoring of infusion-site reactions was added based on an agency request.
7.1.1.9	Trial Compliance (Study	A clarification was provided noting that subjects should not interrupt therapy for ≥7

	Therapy)	consecutive days.
7.1.3.4	CMV DNA Sequence Analysis	Section was revised to include the sequencing of the UL89 terminase gene for viral resistance. This change was made based on an agency request.
8.2.4.1.1	Primary Efficacy Analysis	A sensitivity efficacy analysis was added to include subjects who have detectable CMV viral DNA on Day 1.
5.2; 9.2	Trial Treatments; Packaging and Labeling Information	The volume and duration of study therapy infusion was added based on an agency request.
12.6	Clinical Experience with IV Formulation	The early phase clinical trial data have been removed as these data have been added to the investigator brochure, which will be distributed to investigators participating in the study.

SUMMARY OF CHANGES

PRIMARY REASON(S) FOR THIS AMENDMENT:

Section Number (s)	Section Title(s)	Description of Change (s)
5.1.3	Subject Exclusion Criteria	An additional criterion was added to exclude subjects of Asian descent, defined as a subject who self-reports both parents as being of Asian heritage, i.e., the parents should trace their heritage to one of the following countries: Brunei, Cambodia, East Timor, Indonesia, Japan , Laos, Malaysia, Mongolia, Myanmar (Burma), Korea, People's Republic of China, Philippines, Singapore , Taiwan, Thailand, or Vietnam.
2.1; 5.8; 7.1.5.4	Trial Design; Subject Withdrawal/Discontinuation Criteria; CMV Infection or Early Discontinuation Visit	The protocol was changed to allow a subject to reinitiate protocol-defined study therapy (i.e., letermovir or placebo, based on initial randomization) under the instance where the confirmatory central laboratory test result for CMV DNA PCR, obtained on the day of PET initiation, is negative (CMV DNA not detectable) and PET is stopped.

ADDITIONAL CHANGE(S) FOR THIS AMENDMENT:

Section Number (s)	Section Title(s)	Description of Change (s)
2.1; 5.4	Trial Design; Stratification	A clarification was added noting that the use of *ex vivo* T-cell depleted grafts includes *ex-vivo* use of alemtuzamab [Campath™].
2.1; 6.0; 7.1.3.3	Trial Design; Trial Flow Chart; CMV DNA PCR Testing	A clarification was added that between the Screening Visit and randomization, rather than “from the time of transplantation”, CMV DNA PCR testing will be performed once a week by the central laboratory.

Section Number (s)	Section Title(s)	Description of Change (s)
2.1; 4.2.3.1; 5.8; 6.0; 7.1.3.3; 7.1.5.4	Trial Design; Efficacy Endpoints; Subject Withdrawal/Discontinuation Criteria; Trial Flow Chart; CMV DNA PCR Testing; CMV Infection or Early Discontinuation Visit	A clarification was added noting that the second plasma sample to be collected within 7 days (preferably within 48-72 hours) after PET initiation for CMV DNA PCR testing at the central laboratory only needs to be obtained **IF** the confirmatory CMV DNA PCR result obtained on the day of PET initiation is **NOT** available (e.g., sample is inadequate upon receipt at the central laboratory).
4.2.3.1; 5.1.3; 6.0; 7.1.5.1	Efficacy Endpoints; Subject Exclusion Criteria; Trial Flow Chart; Screening	A clarification was added noting that CMV DNA PCR results from the central laboratory for documented CMV viremia include "detected, not quantifiable" or "detected" with a numeric value provided. The CMV DNA PCR results output was updated to include:<br>• Not detected (<137 IU/mL)<br>• Detected, not quantifiable (<137 IU/mL)<br>• Detected, numeric value provided
5.1.3; 6.0; 7.1.3.1; 7.1.5.1; 12.6	Subject Exclusion Criteria; Trial Flow Chart; Laboratory Safety Evaluations (Hematology, Chemistry, Urinalysis); Screening; Approximate Blood/Tissue Volumes Drawn/Collected by Trial Visit and by Sample Types	A clarification was added noting that the result of an human immunodeficiency virus antibody (HIV-Ab) test documented at <u>any time</u> prior to randomization (rather than 90 days prior to randomization) is acceptable. If documentation of a previous HIV test is not available, then testing must be conducted at the central laboratory prior to randomization.
5.2.2; 6.0; 7.1.5.2.2	Dose Modification; Trial Flow Chart; Study Therapy Administration	A clarification was added noting that if cyclosporine (CsA) A is discontinued permanently or for the long-term in a subject, the next dose of MK-8228 should be increased from 240 mg to 480 mg qd. If CsA is temporarily held due to high CsA levels detected by therapeutic blood monitoring, the dose of MK-8228 need not be adjusted.

Section Number (s)	Section Title(s)	Description of Change (s)
5.5	Concomitant Medications/Vaccinations (allowed & prohibited)	The section was updated noting that:<br>• anti-CMV medications may be used for indications other than CMV treatment/prevention while a subject is on study therapy;<br>• mycophenolate mofetil is removed from the list of medications to be used with caution and language added that no dose adjustment is required when it is co-administered with MK-8228; and<br>• the list of medications to be used with caution has been expanded.
6.0	Trial Flow Chart	A clarification was added noting that any per protocol procedure listed under the Week 14 Visit should be conducted at the true End of Study Therapy Visit (at Week 10, 11, 12, 13, or 14 post-transplant, depending on when study therapy was started).
6.0	Trial Flow Chart	A clarification was added noting that prior to Week 14 post-transplant, all concomitant medications should be reviewed and documented.
7.1.2.5	Child Pugh Score	A clarification was added noting that the Child Pugh Score will be assessed at screening and within 5 days prior to randomization.
7.1.2.7; 6.0; 7.2	Adverse Events Monitoring; Trial Flow Chart; Assessing and Recording Adverse Events	Clarifications were added about adverse event reporting noting the following:<br>From the time of informed consent is signed until randomization, the following adverse events only should be reported: those resulting from protocol-specified procedures or interventions, those resulting in death, and those resulting in a patient not being randomized. After initiation of study therapy, all adverse events will be collected through the post-therapy follow-up Week 2 Visit (at 16 weeks post-transplant) in all subjects, including those who have discontinued study therapy but continue to be followed in the study.
6.0; 7.1.2.7	Trial Flow Chart; Adverse Events Monitoring	A clarification was added noting that infusion-site adverse events will be entered on the adverse event electronic case report form.
6.0; 7.1.3.2.1	Trial Flow Chart; Blood Collection for Pharmacokinetic Sampling	A clarification was added noting that blood collection for pharmacokinetic sampling at the 1 hour timepoint must occur within 10 minutes after infusion completion when the subject is administered intravenous solution.
7.1.3.4	CMV DNA Sequence Analysis	A clarification was provided that a staged genotyping approach will be used to detect resistance mutations in the UL56 and UL89 genes.

THIS PROTOCOL AMENDMENT AND ALL OF THE INFORMATION RELATING TO IT ARE CONFIDENTIAL AND PROPRIETARY PROPERTY OF MERCK SHARP & DOHME CORP., A SUBSIDIARY OF MERCK & CO., INC., WHITEHOUSE STATION, NJ, U.S.A.

SPONSOR:

Merck Sharp & Dohme Corp., a subsidiary of Merck & Co., Inc.
(hereafter referred to as the Sponsor or Merck)
One Merck Drive
P.O. Box 100
Whitehouse Station, NJ 08889-0100, U.S.A.

Protocol-specific Sponsor Contact information can be found in the Investigator Trial File Binder.

TITLE:

A Phase III Randomized, Placebo-controlled Clinical Trial to Evaluate the Safety and Efficacy of MK-8228 (Letermovir) for the Prevention of Clinically Significant Human Cytomegalovirus (CMV) Infection in Adult, CMV-Seropositive Allogeneic Hematopoietic Stem Cell Transplant Recipients

IND NUMBER: 104,706
EudraCT NUMBER: 2013-003831-31

TABLE OF CONTENTS

SUMMARY OF CHANGES **10**
1.0 TRIAL SUMMARY **16**
2.0 TRIAL DESIGN **16**
2.1 Trial Design **16**
2.2 Trial Diagram **21**
3.0 OBJECTIVE(S) & HYPOTHESIS(ES) **21**
3.1 Primary Objective(s) & Hypothesis(es) **21**
3.2 Secondary Objective(s) & Hypothesis(es) **22**
3.3 Exploratory Objectives **22**
4.0 BACKGROUND & RATIONALE **23**
4.1 Background **23**
4.1.1 Pharmaceutical and Therapeutic Background 24
4.2 Rationale **24**
4.2.1 Rationale for the Trial and Selected Subject Population 24
4.2.2 Rationale for Dose Selection/Regimen 27
4.2.3 Rationale for Endpoints 29
4.2.3.1 Efficacy Endpoints 29
4.2.3.2 Safety Endpoints 31
4.2.3.3 Pharmacokinetic Endpoints 32
4.2.3.4 Planned Exploratory Biomarker Research 32
4.2.3.5 Future Biomedical Research 32
5.0 METHODOLOGY **33**
5.1 Entry Criteria **33**
5.1.1 Diagnosis/Condition for Entry into the Trial 33
5.1.2 Subject Inclusion Criteria 33
5.1.3 Subject Exclusion Criteria 34
5.2 Trial Treatments **37**
5.2.1 Dose Selection 38
5.2.2 Dose Modification 39
5.2.3 Timing of Dose Administration 39
5.2.4 Trial Blinding/Masking 39

5.3 Randomization or Treatment Allocation 40
5.4 Stratification 40
5.5 Concomitant Medications/Vaccinations (allowed & prohibited) 41
5.6 Rescue Medications & Supportive Care 44
5.7 Diet/Activity/Other Considerations 44
5.8 Subject Withdrawal/Discontinuation Criteria 45
5.9 Subject Replacement Strategy 47
5.10 Beginning and End of the Trial 47
5.11 Clinical Criteria for Early Trial Termination 48
6.0 TRIAL FLOW CHART 49
7.0 TRIAL PROCEDURES 55
7.1 Trial Procedures 55
7.1.1 Administrative Procedures 55
7.1.1.1 Informed Consent 55
7.1.1.1.1 General Informed Consent 55
7.1.1.1.2 Consent and Collection of Specimens for Future Biomedical Research 56
7.1.1.2 Inclusion/Exclusion Criteria 56
7.1.1.3 Subject Identification Card 56
7.1.1.4 Medical History 56
7.1.1.5 Prior and Concomitant Medications Review 56
7.1.1.5.1 Prior Medications 56
7.1.1.5.2 Concomitant Medications 56
7.1.1.6 HSCT Details Review 57
7.1.1.7 Assignment of Screening Number 57
7.1.1.8 Assignment of Randomization Number 57
7.1.1.9 Trial Compliance (Study Therapy) 57
7.1.2 Clinical Procedures/Assessments 58
7.1.2.1 Physical Examination 58
7.1.2.2 Weight and Height Assessment 58
7.1.2.3 Vital Signs 58
7.1.2.4 12-Lead Electrocardiogram 58
7.1.2.5 Child Pugh Score 59

7.1.2.6 Birth Control Confirmation 59
7.1.2.7 Adverse Events Monitoring 59
7.1.2.8 CMV Disease Assessment 59
7.1.2.9 Health Outcomes Assessment 59
7.1.2.10 Quality of Life Assessment 60
7.1.3 Laboratory Procedures/Assessments 60
7.1.3.1 Laboratory Safety Evaluations (Hematology, Chemistry and Urinalysis) .. 60
7.1.3.2 Pharmacokinetic/Pharmacodynamic Evaluations 61
7.1.3.2.1 Blood Collection for Pharmacokinetic Sampling 61
7.1.3.3 CMV DNA PCR Testing 62
7.1.3.4 CMV DNA Sequence Analysis 63
7.1.3.5 Future Biomedical Research 64
7.1.4 Other Procedures 64
7.1.4.1 Withdrawal/Discontinuation 64
7.1.4.1.1 Withdrawal From Future Biomedical Research 64
7.1.4.2 Blinding/Unblinding 65
7.1.5 Visit Requirements 65
7.1.5.1 Screening 65
7.1.5.2 Study Therapy Period 66
7.1.5.2.1 Day 1 Visit 67
7.1.5.2.2 Study Therapy Administration 67
7.1.5.3 Follow-up Period 68
7.1.5.4 CMV Infection or Early Discontinuation Visit 69
7.2 Assessing and Recording Adverse Events 70
7.2.1 Definition of an Overdose for This Protocol and Reporting of Overdose to the Sponsor 71
7.2.2 Reporting of Pregnancy and Lactation to the Sponsor 72
7.2.3 Immediate Reporting of Adverse Events to the Sponsor 72
7.2.3.1 Serious Adverse Events 72
7.2.3.2 Events of Clinical Interest 73
7.2.3.3 Protocol-Specific Exceptions to Serious Adverse Event Reporting 73
7.2.4 Evaluating Adverse Events 73
7.2.5 Sponsor Responsibility for Reporting Adverse Events 76

7.3 TRIAL GOVERNANCE AND OVERSIGHT ... 76
7.3.1 Scientific Advisory Committee ... 76
7.3.2 Executive Oversight Committee ... 76
7.3.3 Data Monitoring Committee ... 76
7.3.4 Clinical Adjudication Committee ... 77
8.0 STATISTICAL ANALYSIS PLAN ... 77
8.1 Statistical Analysis Plan Summary ... 77
8.1.1 Efficacy Analyses ... 77
8.1.2 Safety Analyses ... 78
8.1.3 Power and Sample Size ... 79
8.1.4 Interim Analysis ... 79
8.2 Statistical Analysis Plan ... 80
8.2.1 Responsibility for Analyses/ In-House Blinding ... 80
8.2.2 Hypotheses/Estimation ... 81
8.2.3 Analysis Endpoints ... 81
8.2.3.1 Efficacy/Pharmacokinetic Endpoints ... 81
8.2.3.1.1 Efficacy Endpoints ... 81
8.2.3.1.2 Exploratory Endpoints ... 83
8.2.3.2 Safety Endpoints ... 83
8.2.4 Analysis Populations ... 84
8.2.4.1 Efficacy Analysis Populations ... 84
8.2.4.2 Safety Analysis Populations ... 84
8.2.5 Statistical Methods ... 85
8.2.5.1 Statistical Methods for Efficacy Analyses ... 85
8.2.5.1.1 Primary Efficacy Analysis ... 85
8.2.5.1.2 Secondary Efficacy Analysis ... 85
8.2.5.1.3 Exploratory Analysis ... 87
8.2.5.1.4 Missing Data Handling ... 87
8.2.5.2 Statistical Methods for Safety Analyses ... 87
8.2.5.3 Summaries of Baseline Characteristics, Demographics, and Other Analyses ... 89
8.2.6 Multiplicity ... 89
8.2.7 Sample Size and Power Calculations ... 90

8.2.7.1 Sample Size and Power for Efficacy Analysis 90
8.2.7.2 Sample Size and Power for Safety Analysis 90
8.2.8 Subgroup Analyses and Effects of Baseline Factors 91
8.2.9 Interim Analyses 91
8.2.10 Compliance/Medication Adherence 92
8.2.11 Extent of Exposure 92
9.0 LABELING, PACKAGING, STORAGE AND RETURN OF CLINICAL SUPPLIES 92
9.1 Investigational Product 92
9.2 Packaging and Labeling Information 93
9.3 Clinical Supplies Disclosure 93
9.4 Storage and Handling Requirements 93
9.5 Returns and Reconciliation 93
9.6 Standard Policies 94
10.0 ADMINISTRATIVE AND REGULATORY DETAILS 94
10.1 Confidentiality 94
10.1.1 Confidentiality of Data 94
10.1.2 Confidentiality of Subject Records 94
10.1.3 Confidentiality of Investigator Information 94
10.1.4 Confidentiality of IRB/IEC Information 95
10.2 Compliance with Financial Disclosure Requirements 95
10.3 Compliance with Law, Audit and Debarment 96
10.4 Compliance with Trial Registration and Results Posting Requirements 97
10.5 Quality Management System 98
10.6 Data Management 98
10.7 Publications 98
11.0 LIST OF REFERENCES 99
12.0 APPENDICES 103
12.1 Merck Code of Conduct for Clinical Trials 103
12.2 Collection and Management of Specimens for Future Biomedical Research 105
12.3 Understanding the Intent, Scope and Public Health Benefits of Exploratory Biomarker Research: A Guide for IRBs/IECs and Investigational Site Staff 111

12.4 Definition of CMV Disease in Hematopoietic Stem Cell Transplant (HSCT) Recipients 122

12.5 Child-Pugh Classification for Severity of Liver Disease 124

12.6 Approximate Blood/Tissue Volumes Drawn/Collected by Trial Visit and by Sample Types 125

13.0 SIGNATURES.. 126

13.1 Sponsor's Representative 126

13.2 Investigator.. 126

LIST OF TABLES

Table 1 Study Therapy – Oral (Tablet) Formulation 37
Table 2 Study Therapy – IV Formulation 38
Table 3 Blinding of Tablets Related to CsA Use 40
Table 4 Laboratory Tests 61
Table 5 Evaluating Adverse Events 74
Table 6 Summary of Analysis Strategy for Key Efficacy Endpoints 78
Table 7 Analysis Strategy for Efficacy Variables 86
Table 8 Analysis Strategy for Safety Parameters 88
Table 9 Upper Bound of the Two-Sided 95% Confidence Interval for the True Proportion of Subjects with an AE 90
Table 10 Product Descriptions 93

LIST OF FIGURES

Figure 1 Trial Diagram 21

SUMMARY OF CHANGES

PRIMARY REASON(S) FOR THIS AMENDMENT:

Section Number (s)	Section Title(s)	Description of Change (s)	Rationale
4.2.2; 5.2; 5.2.2; 5.2.4; 7.1.5.2.2, 9.1	Rationale for Dose Selection/Regimen; Trial Treatments; Dose Modification; Trial Blinding/Masking; Study Therapy Administration; Investigational Product	The protocol was changed to incorporate a 480 mg oral tablet formulation of letermovir.	This change was made to allow for the administration of a 480 mg letermovir tablet (rather than two 240 mg tablets) to subjects whose treatment regimen is 480 mg. The introduction of the 480 mg tablet is based on a Phase I study that provided data showing the bioequivalence of the 480 mg and 2 x 240 mg letermovir oral tablets.
4.2.1; 5.1.3	Rationale for the Trial and Selected Subject Population; Subject Exclusion Criteria	The criterion excluding subjects of Asian descent from the study was removed, and the protocol now allows the randomization of these subjects if all entry criteria are met.	This change was made based on additional Phase I multiple-dose data (Protocol 032) supporting the administration of letermovir in subjects of Japanese descent at the doses to be administered in the study without undue risk to such subjects.

ADDITIONAL CHANGE(S) FOR THIS AMENDMENT:

Section Number (s)	Section Title (s)	Description of Change (s)	Rationale
1.0; 2.1; 2.2; 6.0; 7.1.5.1	Trial Summary; Trial Design; Trial Diagram; Trial Flow Chart;	A clarification was added noting that the screening of potential	The change further clarifies when screening of subjects

	Screening	eligible subjects may begin within 15 days prior to transplantation through 28 days post-transplant. Screening test results must be available within 5 days prior to the planned randomization date.	may occur.
3.3; 4.2.3.4; 6.0; 7.1.3.5; 8.2.3.1.2; 12.2; 12.6	Exploratory Objectives; Planned Exploratory Biomarker Research; Trial Flow Chart; Future Biomedical Research; Exploratory Endpoints; Collection and Management of Specimens for Future Biomedical Research; Approximate Blood/Tissue Volumes Drawn/Collected by Trial Visit and by Sample Types	The section was updated to include the evaluation of whether genetic variation within a clinical trial population correlates with response to the treatment under evaluation.	This exploratory analysis may inform if genetic variation along with other host factors contributes to differences in clinical responses observed when treating patients with letermovir.
5.1.3	Subject Exclusion Criteria	A clarification was added to Exclusion Criterion 3 noting that evidence of CMV viremia (if tested) may be determined at any time from either the signing of the ICF or the HSCT procedure, whichever is earlier, until the time of randomization.	The change further clarifies when the evidence of CMV viremia should be determined for exclusion of subjects from the study.
5.2.2; 5.8; 7.1.5.2.2; 8.2.5.1.1; 8.2.5.1.2	Dose Modification; Subject Withdrawal/Discontinuation Criteria; Study Therapy Administration; Primary Efficacy Analysis; Secondary Efficacy Analysis	Corrections were made for typos and grammar.	These changes were made accordingly to correct the protocol language.

5.5	Concomitant Medications/Vaccinations (Allowed & Prohibited)	Information for the concomitant use of voriconazole with letermovir was added.	Phase I data examining the drug-drug interaction of voriconazole with letermovir (Protocol 025) has become available. Based on these data, the administration of an antifungal other than voriconazole should be considered in subjects for whom antifungal prophylaxis or treatment is required. If coadministration of MK-8228 and voriconazole is necessary, subjects should be monitored closely for breakthrough fungal infections.
2.1; 5.8; 7.1.5.4	Trial Design; Subject Withdrawal/Discontinuation Criteria; CMV Infection or Early Discontinuation Visit	A clarification was added noting that a subject's study therapy status should NOT be changed in IVRS until the CMV DNA PCR result at a CMV Infection Visit is confirmed and it is determined that the study therapy will be permanently discontinued.	The clarification was added so that study personnel may avoid inadvertently discontinuing the subject's study therapy in IVRS in order to allow for reinitiation of study therapy as outlined in the protocol
5.2; 9.1; 9.2	Trial Treatments; Investigational Product; Packaging and Labeling Requirements	The description of the saline placebo and diluent for the intravenous (IV) MK-8228 formulation was removed from Sections 9.1 and 9.2, and a footnote was added to Table 2 in Section 5.2	Note that saline will continue to be used as a placebo and diluent for the MK-8228 IV formulation. The placement of the descriptions in the guidelines will allow the

		stating that the types of placebo and diluent for the IV formulation of MK-8228 are described in the MK-8228-001 Study Drug Preparation Guidelines located in the Investigator Trial File Binder. .	investigators to be promptly informed of updated types of placebo controls and diluents based on compatibility testing with MK-8228, and therefore, able to implement these agents immediately in the study based on a subject's standard of care.
6.0; 7.1.5.1	Trial Flow Chart; Screening	Clarifications were added noting that Informed Consent must be obtained before any study specific procedure is performed and it is acceptable that the date of informed consent is earlier than Screening procedures.	The changes further clarify the consenting procedure.
6.0; 7.1.3.1; 7.1.3.3; 7.1.5.2.2; 7.1.5.3	Trial Flow Chart; Laboratory Safety Evaluations (Hematology, Chemistry, and Urinalysis); CMV DNA PCR Testing; Study Therapy Administration; Follow-up Period	A blood sampling window was added for laboratory safety tests and the CMV DNA PCR test; this window is within 3 days prior to a scheduled study visit and no later than the day of the scheduled study visit. The CMV DNA PCR testing window is not applicable to a CMV Infection Visit.	The change was added to provide the Investigators the opportunity of having results of laboratory safety tests and the CMV DNA PCR assay available at the subject's scheduled study visit.
6.0; 7.1.3.1; 7.1.5.1; 12.6	Trial Flow Chart; Laboratory Safety Evaluations (Hematology, Chemistry, and Urinalysis); Screening; Approximate Blood/Tissue Volumes	A clarification was added noting that chemistry, hematology, and urinalysis testing may be performed by the central laboratory for screening purposes only.	The change clarifies that it is not mandatory to conduct all screening tests for chemistry, hematology, and urinalysis via the central laboratory;

	Drawn/Collected by Trial Visit and by Sample Types		local laboratory testing is acceptable if results are reported appropriately on electronic case report forms.
6.0; 7.1.3.2; 12.6	Trial Flow Chart; Pharmacokinetic/Pharmacodynamic Evaluations; Approximate Blood/Tissue Volumes Drawn/Collected by Trial Visit and by Sample Types	A sample for population pharmacokinetic (PK) evaluation was added at Visit 2 (Day 1 post-transplant).	The population PK sample was added at Visit 2 in order to assess the baseline PK profile in subjects prior to administration of study therapy.
7.1.1.9	Trial Compliance	A clarification was added noting that study therapy may be interrupted for any reason for a time period of <7 consecutive days.	The change provides additional guidance for the Investigator regarding the interruption of study therapy.
7.1.3.4	CMV DNA Sequence Analysis	The section was updated to include a description of a phenotypic analysis that will be performed. Additionally, the section was edited for clarity and to also note that CMV DNA Sequence Analysis will be performed only on subjects enrolled in the MK-8228 arm of the study who meet the criteria for clinically significant CMV infection.	A phenotypic analysis was added to the protocol to further characterize UL56 or UL89 DNA sequences encoding amino acid substitutions in subjects who meet the criteria of clinically significant CMV infection.<br>CMV DNA Sequence Analysis will only be performed on subjects in the MK-8228 treatment arm since it is expected that only those subjects exposed to MK-8228 have the potential to develop mutations leading

			to resistance.
8.1.1; 8.2.5.1.2	Efficacy Analyses; Secondary Efficacy Analysis	Table 6 and Table 7 missing data approach: replaced N/A with Censored at last assessment in the time to event endpoints.	Clarified that the missing data approach for the time to event endpoints.

No additional changes.

1.0 TRIAL SUMMARY

Abbreviated Title	MK-8228 vs. Placebo in Prevention of CMV infection in HSCT Recipients
Trial Phase	Phase III
Clinical Indication	Prevention of clinically significant CMV infection in allogeneic HSCT recipients
Trial Type	Interventional
Type of control	Placebo
Route of administration	Oral, Intravenous
Trial Blinding	Double-blind
Treatment Groups	Arm 1: MK-8228 240 mg once daily (qd), if receiving concomitant cyclosporin A (CsA), or MK-8228 480 mg qd, if not on CsA, through Week 14 (~100 days) post-transplant<br>Arm 2: Placebo qd, through Week 14 (~100 days) post-transplant
Number of trial subjects	Approximately 540 subjects will be enrolled.
Estimated duration of trial	The sponsor estimates that the trial will require approximately 40 months from the time the first subject signs the informed consent until the last subject's last visit.
Duration of Participation	Each subject will participate in the trial for approximately 49 weeks from the time the subject signs the Informed Consent Form (ICF) through the final contact. The Screening phase may vary from 15 days prior to transplantation through 28 days post-transplant, and screening test results must be available within 5 days prior to the planned randomization date. Each subject will receive assigned study therapy (MK-8228 or placebo) through Week 14 (~100 days) post-transplant. After the end of study therapy, each subject will continue to be followed through Week 24 post-transplant. Additionally, they will have follow-up visits at Weeks 32, 40, and 48 post-transplant.
Randomization Ratio	2:1 ratio of MK-8228: placebo (~360 subjects to receive MK-8228 and ~180 subjects to receive placebo)

2.0 TRIAL DESIGN

2.1 Trial Design

This is a randomized, placebo-controlled, multi-site, double-blind trial of MK-8228 (also known as letermovir, AIC246, AIC001; hereafter referred to as MK-8228) in the prevention of clinically significant human cytomegalovirus (CMV) infection in adult, CMV-seropositive allogeneic hematopoietic stem cell transplant (HSCT) recipients. Clinically significant CMV infection is defined as the occurrence of either one of the following outcomes:

- onset of CMV end-organ disease, or
- initiation of anti-CMV pre-emptive therapy (PET) based on documented CMV viremia (<u>as measured by the central laboratory</u>) and the clinical condition of the subject.

Approximately 540 eligible HSCT recipients will be randomized in a 2:1 ratio to receive MK-8228 or placebo (i.e., ~360 and ~180 on MK-8228 and placebo, respectively) at any time from the day of transplant until 28 days post-transplant. Both oral (tablet) and intravenous (IV) formulations of MK-8228 (and placebo) will be available for study therapy. Subjects will be initiated with the oral (tablet) formulation of study therapy provided they are able to swallow and do not have a condition (e.g., vomiting, diarrhea, or a malabsorptive condition) that may interfere with the absorption of the tablets. For subjects who cannot swallow and/or have a condition that may interfere with the absorption of the oral formulation (e.g., vomiting, diarrhea, or a malabsorptive condition), study therapy can be initiated with or switched to the IV formulation. The IV formulation should be switched to oral study therapy (i.e., at the next planned dose) as soon as such subjects are able to swallow and/or the condition necessitating the use of the IV formulation resolves. Use of the IV formulation should generally be limited to 4 weeks or less in duration. However, it will be left to the investigator's discretion to continue IV administration beyond 4 weeks, if the benefit/risk ratio supports continued administration.

The dose of MK-8228 will either be 240 mg once daily (qd), for subjects receiving concomitant cyclosporin A (CsA), or 480 mg qd, if the subject is not on CsA. As CsA has been shown to increase MK-8228 levels, the dose of MK-8228 must be adjusted for subjects taking CsA (concomitant use of other immunosuppressive agents like tacrolimus does not require this adjustment). Placebo for MK-8228 will be administered to maintain study blinding.

Subjects will be stratified by 1) study center and 2) risk (for reactivation of CMV disease) factor group. Risk factor groups include 2 categories as defined below:

1. <u>High risk</u>: Subjects meeting <u>one or more of the following criteria</u> at the time of randomization:

    - Human leukocyte antigen (HLA)-related (sibling) donor with at least one mismatch at one of the following three HLA-gene loci: HLA-A, -B or -DR,
    - Haploidentical donor,
    - Unrelated donor with at least one mismatch at one of the following four HLA-gene loci: HLA-A, -B, -C and -DRB1,
    - Use of umbilical cord blood as stem cell source,
    - Use of *ex vivo* T-cell-depleted grafts (including *ex vivo* use of alemtuzamab [Campath™]),
    - Grade 2 or greater graft-versus-host disease (GVHD), requiring the use of systemic corticosteroids (defined as the use of ≥ 1 mg/kg/day of prednisone or equivalent dose of another corticosteroid).

2. <u>Low risk</u>: All subjects <u>not</u> meeting definition of high risk.

Subjects must have documented seropositivity for CMV (recipient CMV IgG seropositivity [R+]) within one year prior to transplantation to be eligible for the study. Donor CMV serostatus may either be positive (D+) or negative (D-). Screening of potential eligible subjects may begin within 15 days prior to transplantation. Screening may also occur after the transplant, but randomization and start of study therapy must occur no later than 28 days post-transplant. Subjects will have plasma samples tested for CMV viremia using the CMV DNA PCR assay (For initial screening purposes, results of the assay done at a local laboratory will be acceptable. Thereafter, until randomization, CMV DNA PCR testing will be performed once a week by the central laboratory.).

After establishing absence of CMV viremia, subjects will be tested once a week by the central laboratory using the CMV DNA PCR assay until randomization in order to minimize enrollment of those with active CMV replication in the study. Any subject who tests positive for CMV viremia (as documented by central or local laboratory test results) prior to randomization at any time point will be excluded from the study, even if subsequent tests are negative for CMV viremia. Once randomized, CMV viremia will be monitored at the time intervals detailed in the Trial Flow Chart (Section 6.0).

Study therapy (with MK-8228 or placebo) may begin as early as the day of transplant and no later than 28 days post-transplant. Study therapy will continue through Week 14 (~100 days) post-transplant with the primary intent of preventing clinically significant CMV infection. On the day of randomization, eligibility for enrollment into the study should be confirmed (including confirmation that HSCT has taken place). At that time, subjects should have no documented CMV viremia (as confirmed by the central laboratory) from a plasma sample collected within 5 days prior to randomization. In addition, creatinine clearance and liver function test results within 5 days prior to randomization should also be available and within the range allowable in this study as outlined in Section 5.1.3 (Subject Exclusion Criteria).

Once enrolled in the study, subjects will have study visits scheduled at weekly intervals during the treatment period which will be through Week 14 (~100 days) post-transplant. Thereafter, subjects will be followed through Week 24 (~6 months) post-transplant. At all study visits through Week 24 post-transplant, plasma samples will be collected for CMV DNA PCR testing (for testing by central laboratory) and the investigator must assess the subject to determine if the subject meets one of the criteria for clinically significant CMV infection (as defined above).

Following completion of the primary study period at Week 24 (~6 months) post-transplant, all subjects will remain in the study through Week 48 post-transplant in order to continue collecting information on (1) CMV disease; (2) health outcomes data such as incidence of all-cause mortality, re-hospitalizations (including those for CMV-related causes), GVHD, and opportunistic infections; and (3) quality of life (QoL) measures using validated patient reported outcome tools. Study visits will occur at Weeks 32, 40, and 48 post-transplant to collect this information and to collect plasma samples for CMV DNA PCR testing (by the central laboratory) at these time points.

<u>For subjects who develop clinically significant CMV infection during the study treatment period (up to Week 14 post-transplant)</u>: When the investigator intends to initiate either treatment for CMV disease or PET, the subject should have a CMV Infection Visit. It is very important to ensure that all procedures, as outlined in the Trial Flow Chart (Section 6.0), are performed at the CMV Infection Visit **<u>immediately prior to</u>** the initiation of treatment of CMV diseases or initiation of PET (i.e., on the day anti-CMV therapy is initiated). Most importantly, a confirmatory plasma sample for CMV PCR testing at the central laboratory should be collected at this visit. Thereafter, the subject should be discontinued from study therapy and treated according to the local standard of care (outside the context of the study). Such subjects will however, continue to be followed in the study (despite discontinuing study therapy and initiating anti-CMV therapy) and complete all remaining study visits (including all subsequent treatment period visits). At such subsequent visits, all procedures specified in the Trial Flow Chart should be completed with the exception of study therapy administration, pharmacokinetic (PK) assessments, and study medication diary review.

<u>Note on reinitiation of study therapy:</u> There may be instances where confirmatory central lab test results for CMV DNA PCR obtained on the day of PET initiation may be negative (CMV DNA undetectable) and the investigator may wish to discontinue PET. The decision to stop PET in the event of a negative (CMV not detectable) confirmatory central laboratory result collected on the day of PET initiation resides with the investigator caring for the subject. Therefore, in the event the confirmatory CMV DNA sample at PET initiation is negative for CMV viremia, the Sponsor will allow for protocol-defined study therapy (i.e., letermovir or placebo, based on initial randomization) to be restarted at the investigator's discretion, once PET is discontinued. In such instances, study therapy should be restarted within 7 days from the date on which study therapy was stopped. It is important to note that the status of the subject's study therapy in IVRS should NOT be changed until the CMV DNA PCR result is confirmed and the investigator is certain that study therapy will be permanently discontinued.

<u>For subjects with clinically significant CMV infection during the post-treatment [follow-up] period (after Week 14 and through Week 24 post-transplant)</u>: When an investigator intends to initiate either treatment for CMV disease or PET, the subject should have a CMV Infection Visit. It is very important to ensure that all procedures, as outlined in the Trial Flow Chart (Section 6.0), are performed at the CMV Infection Visit **<u>immediately prior to</u>** the initiation of treatment of CMV diseases or initiation of PET (i.e., on the day anti-CMV therapy is initiated). Most importantly, a confirmatory plasma sample for CMV PCR testing at the central laboratory should be collected at this visit. Thereafter, the subject can be treated according to the local standard of care (outside the context of the study). Such subjects will however, continue to be followed in the study (despite initiation of anti-CMV therapy) and complete all remaining study visits). At such subsequent visits, all procedures specified in the Trial Flow Chart should be completed.

Note: It is mandatory to send a confirmatory plasma sample for CMV DNA PCR testing to the central laboratory **immediately prior to** (i.e., on the day of) initiating treatment for CMV disease or PET in **ALL** instances. In the event that the confirmatory result obtained on the day of PET initiation is **NOT** available (e.g., sample is lost or mishandled by the investigator site prior to shipment, or is inadequate upon receipt at the central laboratory), a subsequent sample must be obtained and sent to the central laboratory within 7 days after PET initiation (preferably within 48-72 hours). The subject will be considered to have met the primary efficacy endpoint if this confirmatory laboratory result is positive.

In the event confirmatory test results from the central laboratory are not available within the timeframe the investigator wishes to initiate anti-CMV therapy, the investigator may use a positive local laboratory test result (CMV DNA PCR or pp65 antigen only) in order to make the decision. However, as described above, plasma samples for CMV DNA PCR testing must also be sent to the central laboratory. The local laboratory result must also be reported in such instances.

Subjects who are discontinued from the study for any reason will not be replaced. Subjects who discontinue the study early up to Week 24 post-transplant should complete an Early Discontinuation Visit. Subjects who discontinue the study early after Week 24 post-transplant should have the procedures scheduled for the Week 48 post-transplant visit completed at the time of discontinuation from the study.

In order to ensure safe trial conduct, an independent, unblinded, external Data Monitoring Committee (DMC) will be established for ongoing safety evaluation. The first safety assessment will be done when approximately 10% of the randomized subjects (~54 subjects, 36 on MK-8228 and 18 on placebo) either complete study therapy through Week 14 (~100 days) post-transplant or discontinue therapy prior to that time point. Thereafter, the DMC will review all safety data approximately every 6 months during the trial (or at longer time intervals in case of slow enrollment). In addition, the DMC will also assess futility (i.e., lack of efficacy) when approximately 40% of randomized subjects either complete study therapy through Week 14 (~100 days) post-transplant or discontinue prior to that time point. The DMC will review the safety and interim futility data, consider the overall risk and benefit of continuing the trial to study participants, and make a recommendation to the Executive Oversight Committee (EOC) whether the trial should continue in accordance with the protocol.

Additionally, an independent, blinded Clinical Adjudication Committee (CAC) will be established. This CAC will review clinical, virological, and histopathological data as well as the investigator's assessments for adjudicating all potential cases of CMV end-organ disease, as defined in Appendix 12.4, throughout the trial. The adjudication of cases by the CAC will take precedence over the investigator's assessment.

Population (sparse) pharmacokinetics will be performed on all subjects in this trial. Intensive pharmacokinetic testing will be performed on a subset of subjects (~100 subjects including ~67 on MK-8228 and ~ 33 on placebo). Viral resistance testing will be performed in subjects with clinically significant CMV infection.

Specific procedures to be performed during the trial, as well as their prescribed times and associated visit windows, are outlined in the Trial Flow Chart - Section 6.0. Details of each procedure are provided in Section 7.0 – Trial Procedures.

This study will be conducted in conformance with Good Clinical Practices.

2.2 Trial Diagram

The trial design is depicted in Figure 1.

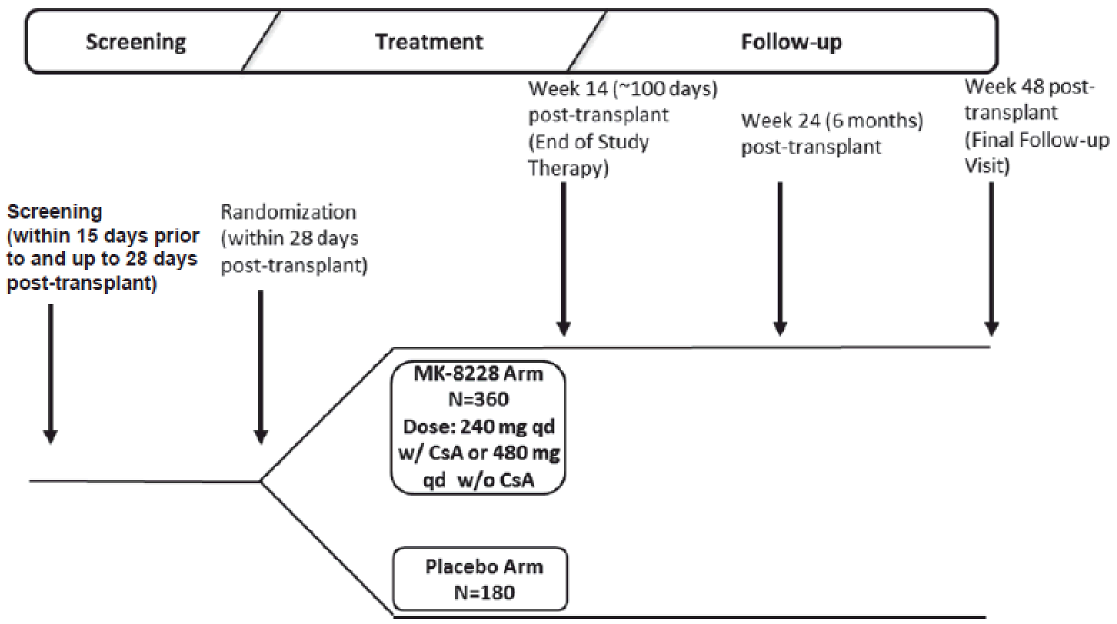

Figure 1 Trial Diagram

3.0 OBJECTIVE(S) & HYPOTHESIS(ES)

3.1 Primary Objective(s) & Hypothesis(es)

1) **Objective:** To evaluate the efficacy of MK-8228 in the prevention of clinically significant CMV infection through Week 24 (~6 months) post-transplant following administration of MK-8228 or placebo.

   Hypothesis: MK-8228 is superior to placebo in the prevention of clinically significant CMV infection, as assessed by the proportion of subjects with CMV end-organ disease or initiation of anti-CMV pre-emptive therapy (PET) based on documented CMV viremia and the subject's clinical condition through Week 24 (~6 months) post-transplant.

3.2 Secondary Objective(s) & Hypothesis(es)

1) **Objective**: To evaluate the safety and tolerability of MK-8228.

2) **Objective**: To evaluate the efficacy of MK-8228 in the prevention of clinically significant CMV infection through Week 14 (~100 days) post-transplant.

3) **Objective**: To evaluate the efficacy of MK-8228 as assessed by time to onset of clinically significant CMV infection through Week 24 (~6 months) post-transplant.

4) **Objective:** To determine the incidence of CMV disease through Week 14 post-transplant and Week 24 post-transplant.

5) **Objective:** To assess the incidence of PET for CMV viremia through Week 14 post-transplant and Week 24 post-transplant.

6) **Objective:** To assess the time to initiation of PET for CMV viremia through Week 14 post-transplant and Week 24 post-transplant.

3.3 Exploratory Objectives

1) **Objective:** To determine the incidence of CMV disease through Week 48 post-transplant.

2) **Objective:** To determine the incidence of all-cause mortality through Week 14 post-transplant, Week 24 post-transplant, and Week 48 post-transplant.

3) **Objective:** To determine the incidence of opportunistic infection other than CMV infection (i.e., systemic bacterial and invasive fungal infection) through Week 14 post-transplant, Week 24 post-transplant, and Week 48 post-transplant.

4) **Objective:** To determine the incidence of acute and/or chronic graft-versus-host disease (GVHD) after randomization through Week 14 post-transplant, Week 24 post-transplant, and Week 48 post-transplant.

5) **Objective:** To determine the incidence of all re-hospitalizations (following initial hospital discharge) and re-hospitalizations for CMV infection/disease through Week 14 post-transplant, Week 24 post-transplant, and Week 48 post-transplant.

6) **Objective:** To assess the incidence of CMV viremia through Week 14 post-transplant and Week 24 post-transplant.

7) **Objective:** To assess the time to CMV viremia through Week 14 post-transplant and Week 24 post-transplant.

8) **Objective:** To determine the incidence of engraftment through Week 14 post-transplant and Week 24 post-transplant. (Engraftment is defined as documented absolute neutrophil counts $\geq 500/mm^3$ on 3 consecutive days.)

9) **Objective:** To determine the time to engraftment through Week 14 post-transplant and Week 24 post-transplant.

10) **Objective:** To evaluate antiviral resistance to MK-8228 in prophylaxis failures.

11) **Objective:** To assess quality of life using the EuroQol (EQ)-5D and Functional Assessment of Cancer Therapy (FACT-BMT) questionnaires through Week 48 post-transplant.

12) **Objective:** To evaluate the pharmacokinetics (PK) of MK-8228.

13) **Objective:** To explore the relationship between genetic variation and response to the treatment(s) administered. Variation across the human genome will be analyzed for association with clinical data collected in this study.

4.0 BACKGROUND & RATIONALE

4.1 Background

CMV continues to be an important complication after allogeneic HSCT [1,2]. The clinical effects of CMV can be divided into direct and indirect effects [1,2]. The direct effects which have been extensively described include the spectrum of CMV disease manifestations. CMV gastroenteritis is the most common clinical presentation in this population. While pneumonia is the most serious manifestation, it has become relatively infrequent with current preventative strategies for CMV disease in HSCT recipients [2,3]. Other rare manifestations of CMV disease include hepatitis, retinitis and encephalitis [1,2]. The indirect effects of CMV include its immunosuppressive effects, which can lead to an increased incidence of systemic bacterial and invasive fungal disease as well as acute and chronic GVHD [2,4].

Recipient CMV seropositivity remains associated with poor outcomes especially in high risk patients such as unrelated donors or cord blood recipients [2,5]. The source of stem cells and the conditioning regimens may also influence both the time to reactivation as well as the severity of disease [5-7]. For preventive purposes, both antiviral prophylaxis and PET (the practice of active surveillance for viral replication and initiating treatment with the detection of viremia) with antivirals are used to manage CMV reactivation in HSCT patients [1]. A recent international survey documented that PET with ganciclovir (GCV) is more commonly used for the purpose, though GCV toxicity is associated with significant myelosuppression and prolonged neutropenia [1,2,7,8]. All currently available anti-CMV agents are associated with significant toxicity and resistance to and cross-resistance across these antiviral agents is increasingly being reported. Thus, antivirals are not routinely used for the prophylaxis of CMV infection in HSCT patients, and there is a clear need for safe and well-tolerated drugs with novel mechanisms of action against CMV that can be used for prophylaxis in HSCT patients.

MK-8228 (letermovir) is an antiviral agent with potent reversible activity against CMV with a novel mechanism of action. It has generally been safe and well tolerated in Phase I and II trials. This study will evaluate the safety and efficacy of MK-8228 in preventing clinically significant CMV infection in adult, CMV IgG-seropositive, allogeneic HSCT patients.

4.1.1 Pharmaceutical and Therapeutic Background

Refer to the Investigator's Brochure (IB) for detailed background information on MK-8228.

There is no oral anti-CMV drug approved for prophylaxis of CMV disease in HSCT patients. Use of GCV, valganciclovir (VGCV), and foscarnet are limited by their toxicity profiles (prolonged myelosuppression, and renal toxicity and electrolyte abnormalities, respectively) [9]. Acyclovir and valacyclovir have limited efficacy against the virus [2,8,10]. Additionally, there is increasing emergence of resistance and cross-resistance to currently available antiviral agents [9,11]. Thus, there is an urgent need for newer efficacious agents with better tolerability and novel mechanisms of action for CMV prophylaxis in HSCT patients.

MK-8228, which belongs to a new class of anti-CMV agents, has a novel mechanism of action. It has demonstrated potent, selective, and reversible inhibition of CMV activity in preclinical studies *in vitro* and efficacy against the virus *in vivo* [11,12]. It inhibits the viral terminase complex (UL56/UL89), an enzyme that plays an important role in cleavage of viral deoxyribonucleic acid (DNA) into unit-length genome and packaging it into procapsids [12,13].

While drug resistance remains rare, most resistance arises during treatment with GCV (or VGCV) as they are used in ~ 90% of patients as first-line agents. Drug resistance is usually seen after treatment with these antiviral agents for duration of weeks to months [9]. GCV and VGCV undergo intracellular phosphorylation by a viral kinase, which is encoded by the CMV gene UL97 during infection and the majority of resistance mutations with use of these therapies map to this gene [11]. It has been postulated that UL97 mutations arise first and confer moderate resistance to GCV (or VGCV) but not to other CMV antivirals, such as cidofovir or foscarnet.

However, all current anti-CMV agents act through the viral polymerase (UL54) and resistance mapping to this gene product leads to cross-resistance among all available agents [11]. MK-8228, through its novel mechanism of action may offer a viable alternative against virus resistant to current anti-CMV drugs. To date, no cross-resistance has been demonstrated between this agent and GCV, foscarnet, cidofovir and acyclovir [14].

4.2 Rationale

4.2.1 Rationale for the Trial and Selected Subject Population

Details regarding specific benefits and risks for subjects participating in this clinical trial may be found in the accompanying IB and Informed Consent documents.

CMV is one of the most important complications after allogeneic HSCT. It can cause multi-organ disease in HSCT recipients, including pneumonia, hepatitis, gastroenteritis, retinitis, and encephalitis [1,2]. Disease can develop both early (within 3 months) and late (> 3 months) after transplantation [15-18]. Recipient CMV seropositivity (IgG) remains a risk factor for mortality associated with transplantation despite major advances in early diagnosis and management of CMV replication and disease [17-21].

CMV infection and disease are associated with "direct effects" - the clinical manifestations of the disease - as well as "indirect effects" [1,2]. Indirect effects include increased risk of opportunistic bacterial and invasive fungal infections in patients with CMV infection [2,4, 22]. Another indirect effect of CMV infection includes the association between CMV and either acute and/or chronic GVHD. Seropositive patients with acute GVHD are at an increased risk of CMV disease [23-25]. Conversely, CMV infection can be a risk factor for acute GVHD in patients receiving T-cell-depleted grafts, as well as for chronic GVHD [19,26,27].

Existing treatment options for CMV are associated with significant toxicity, and there is an unmet medical need for an efficacious anti-CMV drug without dose-limiting toxicities such as bone marrow suppression or renal toxicity in this patient population. Additionally, there is increasing emergence of cross-resistance to existing anti-CMV drugs, albeit at a low rate. MK-8228 (letermovir) belongs to a new class of anti-CMV agents with a novel mechanism of action with:

a) significant anti-CMV activity both *in vitro* and *in vivo* studies,
b) a favorable safety profile demonstrated in several Phase I and Phase II trials, and
c) activity against viral isolates resistant to marketed anti-CMV agents which map to the UL-54 or UL-97 genes as MK-8228 activity maps to the UL56 (terminase) gene [14].

Accordingly, this trial will investigate the safety and efficacy of MK-8228 administered through Week 14 (~100 days) post-transplant for the prevention of clinically significant CMV infection in adult, CMV IgG seropositive recipients of allogeneic HSCT. The trial will include subjects particularly at high risk for CMV disease as defined in Section 2.1 (Trial Design).

Letermovir in Subjects of Asian Descent

A Phase 1 study (PN027) was conducted in Japan to assess the safety, tolerability, and pharmacokinetics of MK-8228 in Japanese subjects. In this Phase 1 study, exposure to MK-8228 was 1.5- to 2.5-fold higher in Japanese subjects administered single doses (240, 480, 720, or 960 mg) orally or intravenously, when compared to historical non-Japanese control subjects. While no safety events of concern were reported as a result of the increased exposure, this study was amended to exclude individuals of Asian descent until additional data was obtained to eliminate any potential safety issues in this population as a result of MK-8228 administration.

A second Phase 1 study (PN032) was subsequently conducted in the US in subjects of Japanese descent to assess the safety, tolerability, and pharmacokinetics of multiple doses of MK-8228 with and without cyclosporine A co-administration. Preliminary results indicate that the exposure to a 480 mg dose of MK-8228 in Japanese was moderately higher than that in non-Japanese. The geometric mean ratio Japanese/non-Japanese (90% CI) AUC_{0-24} was 1.91 (1.40, 2.64) and C_{max} 1.60 (1.22, 2.09). CsA co-administration increased the MK-8228 exposure 2.11-fold (1.72, 2.59), comparing AUC_{0-24}, and 1.48 (1.24, 1.77) comparing C_{max}. This increase is similar to that predicted in a Phase 2 study (AIC246-01-II-2) in which non-Japanese hematopoietic stem cell transplant patients were treated with both CsA and 240 mg MK-8228 PO QD for 84 days (N=17).

Japanese PK data from Phase 1 clinical trials were reviewed for comparison to the PK target used to define the Phase 3 clinical dose. The Phase 3 clinical dose, selected to meet a threshold exposure of 45,000 ng.hr/mL above which no prophylaxis failures occurred, was based on Phase 2 data in HSCT recipients (AIC246-01-II-2). The 480 mg dose for Phase 3, was selected from population pharmacokinetics modeling as the dose predicted to achieve exposure above the threshold target in > 90% of patients (predicted mean AUC 79,346 ng.hr/mL).

The safety margin for the 480 mg clinical dose is based upon PN026 (study to assess the safety, tolerability and pharmacokinetics of multiple oral and intravenous doses of MK-8228 in healthy female subjects) that administered 720 mg orally BID for 14 days. Although ~67% subjects with the highest exposures had mild/moderate gastrointestinal intolerability, no serious adverse events (AE) or discontinuation of treatment due to an AE with MK-8228 have been observed, with exposures of 328,000 ng.hr/mL (272,000, 402,000) (compared to mean exposures of ~ 70,000 ng.hr/mL with the 480 mg clinical dose). No trend of effects on vital signs (blood pressure, pulse rate), 12-lead electrocardiogram or clinical laboratory parameters has been detected with MK-8228 dosing. No dose-limiting toxicity with MK-8228 has been observed. The margin of safety established in PN026 is 4.6-fold for AUC_{0-24}. The margin of safety for C_{max} established in the QTc study (PN004) is 3-fold (with a lower 2-fold margin for the IV formulation.

MK-8228 is well tolerated with a safety margin that encompasses the increased exposures in individuals of Japanese ethnicity at the 480 mg clinical dose. In addition, 480 mg dosed (3 MK-8228:1 with placebo, N=12) for 7 days to Japanese subjects in PN032 was well tolerated. The safety margin for AUC would encompass the additional increased exposures seen with moderate hepatic or renal insufficiency in Asian patients. Increases in C_{max} greater than the margin in the QTc study are currently being assessed by safety ECG monitoring in this study.

It is concluded that maintaining the efficacy exposure target in the Japanese population with treatment with 480 mg MK-8228 alone or 240 mg MK-8228 concomitantly with CsA will not expose patients to undue risk.

4.2.2 Rationale for Dose Selection/Regimen

Please refer to the MK-8228 IB for further details of preclinical data and study results in humans.

Rationale for Dose Selection

MK-8228 belongs to a new class of anti-CMV agents which have a different mechanism of action compared to currently available drugs for the treatment of CMV infection. By inhibiting the viral terminase complex, the drug plays a key role in cleavage and packaging of genomic virus DNA into provirions.

MK-8228 is anticipated to be efficacious based on both the *in vitro* potency of letermovir as well as its *in vivo* efficacy in a Phase IIB dose-ranging trial (AIC246-01-II-02) in HSCT recipients. *In vitro*, the drug exhibits potent activity against CMV in cell cultures with EC_{90} values in the nanomolar range.

The drug has also been shown to be efficacious in the prophylaxis of CMV disease in a Phase IIB study of HSCT recipients (AIC246-01-II-02). In this trial, three doses of MK-8228, 60 mg qd (n=33), 120 mg qd (n=31) and 240 mg qd (n=34), were compared to placebo (n=31) when given over 84 days for CMV prophylaxis.

One of the primary efficacy endpoints, the incidence of overall failure of CMV prophylaxis over the 84 day treatment period, was significantly reduced in the primary population, the Full Analysis Set (FAS), with the 120 mg and 240 mg doses of MK-8228 (32%, p=0.014 and 29%, p=0.007, respectively) when compared to placebo. However, the second primary efficacy endpoint, the time to onset of overall failure was significantly reduced in the 240-mg arm alone (p=0.002), but not the 120-mg arm (p=0.126), compared to placebo in the FAS. Furthermore, all sensitivity analyses confirmed the statistical significance of both primary endpoints in the FAS for the 240-mg once-daily dose of MK-8228 versus placebo.

Of note, the only 2 subjects in the 240-mg arm who failed CMV prophylaxis in the study had CMV viremia on the first day of treatment, indicating pre-treatment CMV replication. Therefore, in effect, there was no CMV prophylaxis failure with the 240-mg once-daily dose of MK-8228 when excluding subjects with active CMV replication on Day 1 of treatment.

In a Phase IIA proof-of-concept trial (AIC001-2-001), 18 subjects were treated with 40 mg bid or 80 mg qd for 14 days. MK-8228 was generally safe and well tolerated in this study. Similarly, in the Phase IIB dose ranging study (AIC246-01-II-02, described above), 98 subjects were treated with 60 mg qd, 120 mg qd, or 240 mg qd of MK-8228 for 84 days. All doses were well tolerated with a safety profile similar to placebo.

MK-8228 has been safe and generally well tolerated in 14 Phase I and the Phase II clinical trials. In the Phase I trials MK-8228 was administered in single oral or IV doses ranging from 5 mg to 960 mg (n=235) in healthy male and female subjects, and multiple oral or IV doses ranging from 30 mg qd to 320 mg bid (n=230) in healthy subjects as well as subjects with moderate to severe hepatic impairment for up to 14.5 days. In these trials, there were no deaths and there was one serious adverse event (SAE) which was not considered to be drug-related. Similarly, MK-8228 was safe and well-tolerated in 116 transplant patients in the Phase II studies who were exposed to doses ranging from 80 mg qd/40 mg bid to 240 mg qd for up to 84 days.

In all the above trials, MK-8228 had no significant effects on vital signs, clinical or laboratory parameters, or electrocardiogram (ECG).

Phase I studies have demonstrated that co-administrations with cyclosporin A (CsA) increases MK-8228 exposure ~3 fold. Further analyses using the Phase IIB study data indicate that exposure with the 240 mg dose of MK-8228 administered alone overlaps exposure levels of the 60 and 120 mg once-daily doses which are associated with virologic failures. Most such failures occurred at MK-8228 levels with AUC_{tau} values < 45,000 ng*h/mL. Consequently, an efficacy target for success in > 90% of the subjects, was set at AUC_{tau} levels ≥ 45,000 ng*h/mL. It is predicted that this target level will be achieved with a dose of 240 mg of MK-8228 once daily in subjects receiving CsA, and with 480 mg of MK-8228 once daily in the absence of CsA. Modeling and simulation data indicate that exposure levels of MK-8228 with the 480 mg dose in the absence of CsA will not exceed exposure levels seen with the 240 mg dose of MK-8228 when administered with CsA in the Phase IIB data (n=18). These exposure levels were not also associated with any significantly increased adverse events when compared to placebo used in the study. Additional preliminary population PK analyses indicate that other covariates including gender and weight do not have a meaningful effect on MK-8228 exposure.

Based on all available safety data, MK-8228 efficacy in the Phase II studies, and the exposure-response data, this study will use a dose of 240 mg qd for subjects receiving CsA and 480 mg qd in subjects who are not receiving CsA concomitantly.

Bioequivalence of the 240 mg and 480 mg oral (tablet) formulations

A Phase I study (PN028) was conducted to characterize the pharmacokinetic profile of a 480 mg tablet of MK-8228 compared to 2 x 240 mg tablets of MK-8228. Fourteen healthy female subjects received two treatments with MK-8228 under fasting conditions; a single oral dose of one 480 mg tablet (Treatment A), and a single dose of 2 x 240 mg tablets (Treatment B). Pharmacokinetic parameters, including C_{max}, $AUC_{0\text{-last}}$, and $AUC_{0\text{-}\infty}$, were similar following single dose administration of one 480 mg tablet or 2 x 240 mg tablets. The GMR (90%CI) for C_{max}, $AUC_{0\text{-last}}$, and $AUC_{0\text{-}\infty}$ (480 mg tablet/2 x 240 mg tablets) were 1.07 (0.95, 1.21), 1.10 (1.02, 1.18) and 1.09 (1.01, 1.18), respectively, and within the bioequivalence interval of 80 - 125%.

<u>*Rationale for Study Duration*</u>

Most HSCT patients are at highest risk for CMV disease within the first 3 months (~100 days) after transplantation. Antiviral prophylaxis with MK-8228 was efficacious when used for 84 days in the Phase IIB (AIC246-01-II-02) study outlined above. Therefore, study therapy with MK-8228 will be used for antiviral prophylaxis through Week 14 (~100 days) post-transplant in this study. Subjects will then be followed through Week 24 post-transplant (an additional 10 weeks following completion of study therapy) in order to evaluate the incidence of late-onset CMV infection/disease. Finally, further information regarding CMV disease, health-outcomes measures (such as incidence of all-cause mortality, re-hospitalizations, GVHD, and opportunistic infections), as well as quality of life (QoL) measures, will be collected up to Week 48 post-transplant.

4.2.3 Rationale for Endpoints

4.2.3.1 Efficacy Endpoints

The primary efficacy endpoint of the study will be the proportion of subjects with clinically significant CMV infection through Week 24 (~6 months) post-transplant, defined as the occurrence of either one of the following outcomes:

- onset of CMV end-organ disease

  OR

- initiation of anti-CMV PET based on documented CMV viremia (<u>as measured by the central laboratory</u>) and the clinical condition of the subject. Initiation of PET in this study refers to the practice of initiating therapy with the following approved anti-CMV agents when active CMV viral replication is documented: ganciclovir, valganciclovir, foscarnet, and/or cidofovir.

CMV disease will be determined using the definitions in Appendix 12.4 and confirmed by an independent, blinded Clinical Adjudication Committee (CAC). The CAC will review clinical, virological, and histopathological data as well as the investigator's assessments throughout the trial for adjudicating all potential cases of CMV disease. The adjudication of cases by the CAC will take precedence over the investigator's assessment.

Currently, with most centers using CMV preventive strategies, including PET, the overall incidence of CMV disease in HSCT patients has declined to around 5% in the first 3 months post-transplant, from 20-30% prior to the routine use of preventive measures [2,4,6,19,28-31]. Accordingly, sample sizes required to show efficacy of novel anti-CMV drugs for antiviral prophylaxis using the incidence of CMV disease alone would be high [27,31]. Thus, the primary endpoint of this study will also include the incidence of anti-CMV PET initiation based on detection of CMV viremia and the clinical condition of the subject.

As detection of CMV in plasma or blood is associated with an increased risk of CMV disease [32-35], CMV viral DNA as a measure of CMV infection is already used routinely in clinical practice to initiate and monitor PET [2,9,29,36,37]. Patients with high viral loads or with cumulative high viral loads are at an increased risk of developing disease than those with lower viral loads [36,37]. However, there is no clinically validated viral load threshold for initiating pre-emptive therapy at this point in time. Some centers initiate pre-emptive therapy using a single cut-off value for viremia, while others use a risk-based approach for the purpose [2,9].

In this study, CMV viremia (viral load) will be measured on plasma samples using the Roche COBAS® AmpliPrep/COBAS TaqMan® (CAP/CTM) assay, which will be performed <u>by the central laboratory</u>. The lower limit of quantification (LLoQ) for this assay is 137 IU/ml which is ~150 copies/mL (using a conversion factor of 1.1 copies/IU as per the assay package insert). Results will be reported as:

- Not detected (<137 IU/mL)
- Detected, not quantifiable (<137 IU/mL)
- Detected (numeric value provided)

Documented viremia is defined as any detectable **(includes reporting of PCR results as "detected, not quantifiable" or "detected" with a numeric value provided)** CMV viral DNA on a confirmatory sample obtained immediately prior to (i.e., on the day of) the initiation of treatment for CMV disease or PET, as measured by the Roche COBAS® AmpliPrep/COBAS TaqMan® (CAP/CTM) System in the central laboratory. While any detectable CMV viral DNA results in the Roche CAP/CTM assay from confirmatory plasma sample sent to the central laboratory is acceptable for the purpose of documenting viremia as a component of the primary endpoint, it is strongly recommended that investigators should not initiate PET when CMV viral load is below the LLoQ, but detectable (detected, not quantifiable [<137 IU/mL]). The guidance regarding viral load thresholds for initiation of PET in this trial are based on risk as defined in the study stratification (see Section 2.1, Trial Design) as well as consideration of standard practice described in ref. 9, and are as follows:

<u>During the study treatment period [through Week 14 (~100 days) post-transplant]</u>

- High risk: viral DNA ≥150 copies/mL
- Low risk: viral DNA >300 copies/mL

<u>After Week 14 (~100 days) post-transplant</u>

- High risk: viral DNA >300 copies/mL
- Low risk: viral DNA >300 copies/mL

While the viral threshold suggested for initiating PET in low-risk patients in the reference cited above [9] may be as high as 1,000 copies/ml by the assay used at the Fred-Hutchinson Cancer Research Center (FHCRC), this viral DNA level corresponds to a level of ~ 300 copies/ml using the Roche CAP/CTM assay, which will be used in this study (1,000 copies/ml in the FHCRC assay ~ 250 IU/ml in the Roche CAP/CTM assay ~ 275 copies/ml in the Roche CAP/CTM assay, PPD [redacted] personal communication]). Importantly, these thresholds for initiation of PET are provided as guidance based on the subject's risk for CMV disease at baseline. However, specific thresholds for initiating PET are not mandated per protocol as a subject's risk status and clinical condition may change during the course of the trial and is best assessed by the investigator taking care of the subject.

Note: **All** study-related CMV DNA PCR samples must be sent to the central laboratory at the designated time points in the Trial Flow Chart (Section 6.0). It is strongly recommended that investigators use viremia (at levels > LLoQ) detected by the central laboratory to drive decisions for initiating PET. It is mandatory to send a confirmatory plasma sample for CMV DNA PCR testing to the central laboratory **immediately prior to** (i.e., on the day of) initiating treatment for CMV disease or PET in **ALL** instances. In the event that the confirmatory result obtained on the day of PET initiation is **NOT** available (e.g., sample is lost or mishandled by the investigator site prior to shipment, or is inadequate upon receipt at the central laboratory), a subsequent sample must be obtained and sent to the central laboratory within 7 days after PET initiation (preferably within 48-72 hours). The subject will be considered to have met the primary efficacy endpoint if this confirmatory laboratory result is positive.

In the event test results from the central laboratory are not available within the timeframe the investigator wishes to initiate anti-CMV therapy, the investigator may use a positive local laboratory test (CMV DNA PCR or pp65 antigen only) result in order to make the decision. However, as described above, plasma samples for CMV DNA PCR testing must also be sent to the central laboratory. The local laboratory result must also be reported in such instances.

4.2.3.2 Safety Endpoints

The safety and tolerability of MK-8228 will be assessed by a clinical evaluation of adverse experiences and evaluation of other study parameters including vital signs, physical examination, 12-lead ECGs, and standard laboratory safety tests at appropriate time points as specified in the Trial Flow Chart (Section 6.0). Serum inhibin B, luteinizing hormone (LH), follicle-stimulating hormone (FSH), and testosterone levels in men will be collected to monitor testicular function. Adverse experiences should be graded and recorded as outlined in Section 7.2. Subjects may be asked to return for unscheduled visits for additional safety monitoring.

4.2.3.3 Pharmacokinetic Endpoints

Population PK will be performed on all subjects. Intensive PK will be performed on a subset of subjects (~100 subjects, including ~67 on MK-8228 and ~33 on placebo). The PK endpoints for MK-8228 are: area under the concentration-time curve to the end of the dosing period ($AUC_{0\text{-}24}$), area under the concentration-time curve up to the last measurable concentration ($AUC_{0\text{-last}}$), maximum concentration observed (C_{max}, for subjects receiving tablet formulation), time to maximum observed plasma drug concentration (t_{max}, for subjects receiving tablet formulation), concentration at the end of infusion (C_{eoi}, for subjects receiving IV formulation), and minimum concentration observed (C_{trough}). Plasma samples for pharmacokinetic (PK) evaluations will be collected at the time points described in the Trial Flow Chart (Section 6.0).

4.2.3.4 Planned Exploratory Biomarker Research

Understanding genetic determinants of drug response is an important endeavor during medical research. This research will evaluate whether genetic variation within a clinical trial population correlates with response to the treatment(s) under evaluation. If genetic variation is found to predict efficacy or adverse events, the data might inform optimal use of therapies in the patient population. This research contributes to understanding genetic determinants of efficacy and safety associated with the treatments in this study. For example, variants in the *SLCO1B1* (OATP1B1) gene may be investigated in relation to pharmacokinetic variability as well as efficacy and adverse events.

4.2.3.5 Future Biomedical Research

The Sponsor will conduct Future Biomedical Research on specimens routinely and specifically collected during this clinical trial. This research may include genetic analyses (DNA), and/or the measurement of other analytes.

Such research is for biomarker testing to address emergent questions not described elsewhere in the protocol (as part of the main trial) and will only be conducted on specimens from appropriately consented subjects. The objective of collecting specimens for Future Biomedical Research is to explore and identify biomarkers that inform the scientific understanding of diseases and/or their therapeutic treatments. For instance, exploratory pharmacogenetic (PGt) studies may be performed if significant Pharmacokinetic/Pharmacodynamic (PK/PD) relationships are observed or adverse events are identified. Genomic markers of disease may also be investigated. Such retrospective pharmacogenetic studies will be conducted with appropriate biostatistical design and analysis and compared to PK/PD results or clinical outcomes. Any significant PGt relationships to outcome would require validation in future clinical trials. The overarching goal is to use such information to develop safer, more effective drugs, and/or to ensure that subjects receive the correct dose of the correct drug at the correct time. The details of this Future Biomedical Research sub-trial are presented in Section 12.2 - Collection and Management of Specimens for Future Biomedical Research. Additional informational material for institutional review boards/ethics committees (IRBs/ERCs) and investigational site staff is provided in Section 12.3.

5.0 METHODOLOGY

5.1 Entry Criteria

5.1.1 Diagnosis/Condition for Entry into the Trial

Male/Female subjects with receipt of an allogeneic HSCT of at least 18 years of age will be enrolled in this trial.

5.1.2 Subject Inclusion Criteria

In order to be eligible for participation in this trial, the subject must:

1. be ≥ 18 years of age on the day of signing informed consent.

2. have documented seropositivity for CMV (recipient CMV IgG seropositivity [R+]) within 1 year before HSCT.

3. be receiving a first allogeneic HSCT (bone marrow, peripheral blood stem cell, or cord blood transplant).

4. have undetectable CMV DNA (as confirmed by the central laboratory) from a plasma sample collected within 5 days prior to randomization.

5. be within 28 days post-HSCT at the time of randomization.

6. be highly unlikely to become pregnant or to impregnate a partner since they meet at least one of the following criteria:

   a) A female subject who is not of reproductive potential is eligible without requiring the use of contraception. A female subject who is not of reproductive potential is defined as one who: (1) has reached natural menopause (defined as 6 months of spontaneous amenorrhea with serum follicle-stimulating hormone [FSH] levels in the postmenopausal range as determined by the local laboratory, or 12 months of spontaneous amenorrhea); (2) is 6 weeks post-surgical bilateral oophorectomy with or without hysterectomy; or (3) has undergone bilateral tubal ligation. Spontaneous amenorrhea does not include cases for which there is an underlying disease that causes amenorrhea (e.g., anorexia nervosa).

   b) A male subject who is not of reproductive potential is eligible without requiring the use of contraception. A male subject who is not of reproductive potential is defined as one whom has undergone a successful vasectomy. A successful vasectomy is defined as: (1) microscopic documentation of azoospermia, or (2) a vasectomy more than 2 years ago with no resultant pregnancy despite sexual activity postvasectomy.

c) A male or female subject who is of reproductive potential agrees to true abstinence or to use (or have their partner use) 2 acceptable methods of birth control starting from the time of consent through 90 days after the last dose of study therapy. Longer periods of birth control may be required per local requirements. True abstinence is defined as abstinence in line with the preferred and usual lifestyle of the subject. Periodic abstinence (e.g., abstinence only on certain calendar days, abstinence only during ovulation period, use of symptothermal method, use of post-ovulation methods) and withdrawal are not acceptable methods of contraception. Acceptable methods of birth control are: intrauterine device (IUD), diaphragm with spermicide, contraceptive sponge, condom, and vasectomy OR use of appropriate double barrier contraception as per local regulations or guidelines. Hormonal contraceptives (e.g., birth control pills, transdermal patch, or injectables) are unacceptable methods of birth control for use in this study because it is not known whether these methods are affected by co-administration of MK-8228.

7. be able to read, understand, and complete questionnaires and diaries.

8. understand the study procedures, alternative treatment available, and risks involved with the study, and voluntarily agree to participate by giving written informed consent. The subject may also provide consent for Future Biomedical Research. However, the subject may participate in the main trial without participating in Future Biomedical Research.

5.1.3 Subject Exclusion Criteria

The subject must be excluded from participating in the trial if the subject:

1. received a previous allogeneic HSCT (**Note**: Receipt of a previous autologous HSCT is acceptable).

2. has a history of CMV end-organ disease within 6 months prior to randomization.

3. has evidence of CMV viremia (if tested) at any time from either signing of the ICF or the HSCT procedure, whichever is earlier, until the time of randomization. **(Note: Evidence of CMV viremia as reported by central lab will include reporting of test results as “detectable, not quantifiable” or “detected” with a numeric value provided.)**

4. received within 7 days prior to screening or plans to receive during the study any of the following:
    - ganciclovir
    - valganciclovir
    - foscarnet
    - acyclovir (at doses > 3200 mg PO per day or > 25 mg/kg IV per day)
    - valacyclovir (at doses > 3000 mg PO per day)
    - famciclovir (at doses > 1500 mg PO per day)

5. received within 30 days prior to screening or plans to receive during the study any of the following:
   - cidofovir
   - CMV hyper-immune globulin
   - Any investigational CMV antiviral agent/biologic therapy

6. has suspected or known hypersensitivity to active or inactive ingredients of letermovir formulations.

7. has severe hepatic insufficiency (defined as Child-Pugh Class C; see Appendix 12.5) within 5 days prior to randomization.

8. has serum aspartate aminotransferase (AST) or alanine aminotransferase (ALT) > 5 x the upper limit of normal (ULN) or serum total bilirubin > 2.5 x ULN within 5 days prior to randomization.

   Note: Subjects who meet this exclusion criterion may, at the discretion of the investigator, have one repeat testing done prior to randomization. If the repeat value does not meet this criterion, they may continue in the screening process. Only the specific out of range value should be repeated (not the entire panel).

9. has end-stage renal impairment with a creatinine clearance less than 10 mL/min, as calculated by the Cockcroft-Gault equation using serum creatinine within 5 days prior to randomization

   $$\text{Creatinine Clearance (Males)} = \frac{(\text{weight in kg})\ (140 - \text{age})}{(72)\ (\text{creatinine in mg/dL})}$$

   Creatinine Clearance (Females) = 0.85 x the value obtained with formula above

   Note: Subjects who meet this exclusion criterion may, at the discretion of the investigator, have one repeat testing done within 5 days prior to randomization. If the repeat value does not meet this criterion, they may continue in the screening process. Only the specific out of range value should be repeated (not the entire panel).

10. has both moderate hepatic insufficiency AND moderate renal insufficiency.

    Note: Moderate hepatic insufficiency is defined as Child Pugh Class B (see Appendix 12.5); moderate renal insufficiency is defined as a creatinine clearance less than 50 mL/min, as calculated by the Cockcroft-Gault equation (as above), respectively.

11. has an uncontrolled infection on the day of randomization.

12. requires mechanical ventilation or is hemodynamically unstable at the time of randomization.

13. has a documented positive result for an human immunodeficiency virus antibody (HIV-Ab) test at any time prior to randomization, or for hepatitis C virus antibody (HCV-Ab) with detectable HCV RNA, or hepatitis B surface antigen (HBsAg) within 90 days prior to randomization.

14. has active solid tumor malignancies with the exception of localized basal cell or squamous cell skin cancer or the condition under treatment (e.g., lymphomas).

15. is pregnant or expecting to conceive, is breastfeeding, or plans to breastfeed from the time of consent through 90 days after the last dose of study therapy.

16. is expecting to donate eggs or sperm starting from the time of consent through 90 days after the last dose of study therapy.

17. is currently participating or has participated in a study with an *unapproved* investigational compound or device within 28 days, or 5X half-life of the investigational compound (excluding monoclonal antibodies), whichever is longer, of initial dosing on this study. Subjects previously treated with a monoclonal antibody will be eligible to participate after a 28-day washout period.

    Note: Investigational chemotherapy regimens involving *approved* agents and investigational antimicrobial regimens involving *approved* antibacterial/antifungal/antiviral agents, investigational radiotherapy studies, or other observational studies are allowed.

18. has previously participated in this study or any other study involving MK-8228 (letermovir).

19. has previously participated or is currently participating in any study involving administration of a CMV vaccine or another CMV investigational agent, or is planning to participate in a study of a CMV vaccine or another CMV investigational agent during the course of this study.

20. is or has an immediate family member (spouse or children) who is investigational site or Sponsor staff directly involved with this trial.

21. is, at the time of signing informed consent, a user of recreational or illicit drugs or has had a recent history (within the last year) of drug or alcohol abuse or dependence.

    Note: Subject who has a history of recreational marijuana use which is not deemed excessive by the subject's investigator or does not interfere with the subject's daily function may participate in the study but must be instructed to discontinue any further use of recreational marijuana prior to entry into trial and throughout the trial period.

22. has a history or current evidence of any condition, therapy, lab abnormality, or other circumstance that might confound the results of the study, interfere with the subject's participation for the full duration of the study, or would be put at undue risk as judged by the investigator, such that it is not in the best interest of the subject to participate in this study.

5.2 Trial Treatments

In this study, subjects will be randomized in a 2:1 ratio with ~360 subjects on MK-8228 and ~180 subjects on placebo. Both oral (tablet) and IV formulations of MK-8228 (and placebo) to be used in this trial are outlined below in Table 1 and Table 2, respectively. Subjects will be initiated with the oral (tablet) formulation of study therapy provided they are able to swallow and do not have a condition (e.g., vomiting, diarrhea, or a malabsorptive condition) that may interfere with the absorption of the tablets. For subjects who cannot swallow and/or have a condition that may interfere with the absorption of the oral formulation (e.g., vomiting, diarrhea, or a malabsorptive condition), study therapy can be initiated with or switched to the IV formulation. Use of the IV formulation should generally be limited to 4 weeks or less in duration. However, it will be left to the investigator's discretion to continue IV administration beyond 4 weeks, if the benefit/risk ratio supports continued administration. Simultaneous use of IV and oral study therapy is not allowed. With this protocol amendment, both 240 mg and 480 mg oral (tablet) formulations of MK-8228 will be made available by the Sponsor. Subjects requiring the oral 480 mg dose should be initiated with one 480 mg tablet (MK-8228 or matching placebo). In the event a subject is unable to swallow the 480 mg tablet, study therapy may be initiated with 2 x 240 mg tablets (MK-8228 or matching placebo). Subjects who were initiated with two tablets of the 240 mg MK-8228 or matching placebo should continue with that regimen. (The 480 mg tablet may not be available in all countries based on country-specific requirements. In such instances, 2 x 240 mg tablets should be used for the 480 mg dose.)

Table 1 Study Therapy – Oral (Tablet) Formulation

Drug	Dose /Potency	Dose Frequency	Route of Administration	Regimen/Treatment Period	Use
MK-8228 (for subjects on CsA)	240 mg	Once daily (qd)	Oral	Through Week 14 (~100 days) post-transplant	Experimental
MK-8228 (for subjects not on CsA)	480 mg (one 480-mg tablet or two 240-mg tablets)	Once daily (qd)	Oral	Through Week 14 (~100 days) post-transplant	Experimental
Placebo to MK-8228*	NA	Once daily (qd)	Oral	Through Week 14 (~100 days) post-transplant	Placebo-comparator

CsA = Cyclosporin A
* The number of placebo tablets will mimic the MK-8228 dosing scheme in an effort to maintain the blind (i.e., 1 matching placebo tablet if on CsA; 1 or 2 matching placebo tablets if not on CsA to mimic one 480 mg tablet or two 240 mg tablets, respectively).

Table 2 Study Therapy – IV Formulation

Drug	Dose/ Potency	Dose Frequency	Route of Administration	Regimen/Treatment Period	Use
MK-8228 (for subjects on CsA) *	240 mg	Once daily (qd)	Intravenous**	Through Week 14 (~100 days) post-transplant***	Experimental
MK-8228 (for subjects not on CsA) *	480 mg	Once daily (qd)	Intravenous**	Through Week 14 (~100 days) post-transplant***	Experimental
Placebo to MK-8228*	NA	Once daily (qd)	Intravenous**	Through Week 14 (~100 days) post-transplant***	Placebo-comparator

CsA = Cyclosporin A.
* The types of placebo and diluent for the IV formulation of MK-8228 are described in the **MK-8228-001 Study Drug Preparation Guidelines** located in the Investigator Trial File Binder.
** MK-8228 intravenous formulation dosing volume is 250 mL and duration of infusion will be 60 minutes.
*** The IV formulation should be switched to oral study therapy (i.e., at the next planned dose) as soon as such subjects are able to swallow and/or the condition necessitating the use of the IV formulation resolves.

Study therapy may begin as early as the day of transplant to no later than 28 days post-transplant, once the subject is determined to be negative for CMV viremia (no evidence of CMV viremia from a central or local laboratory at any time point **and** confirmed by the central laboratory on a sample collected from the subject within 5 days prior to randomization).

5.2.1 Dose Selection

The rationale for selection of doses to be used in this trial is provided in Section 4.0 – Background & Rationale. There are no specific calculations or evaluations required to be performed in order to administer the proper dose to each subject.

Both IV and oral (tablet) formulations of MK-8228 (and matching placebo) will be available. Study therapy should be initiated with the oral formulation in all subjects who are able to swallow and do not have any condition that may interfere with the absorption of oral medication (e.g., vomiting, diarrhea, or a malabsorptive condition).

The IV formulation of MK-8228 contains the excipient hydroxylpropyl-β-cyclodextrin, which can accumulate in patients with renal insufficiency. In this regard, cyclodextrin is also an excipient in other approved IV agents, including IV voriconazole (given with the excipient, sulfolbutylether-β-cyclodextrin). Recent data suggest that, despite accumulation of cyclodextrin in patients with reduced renal function, baseline renal function is not a predictor of worsening renal function in patients receiving IV voriconazole [38]. Additionally, the quantity of cyclodextrin in the MK-8228 IV formulation is less than that in the IV voriconazole formulation (1800 mg of cyclodextrin/vial for a 240-mg dose of MK-8228 as compared to 3200 mg of cyclodextrin/vial for a 200-mg dose of voriconazole, which is administered twice a day).

Based on the above, the use of IV MK-8228 is permitted in subjects with renal insufficiency, provided creatinine clearance is >10 mL/min. However, the IV formulation should only be used when subjects are either unable to swallow or have a condition (e.g., vomiting, diarrhea, or malabsorptive condition) that may interfere with the absorption of the oral formulation. Subjects on IV MK-8228 should be switched to the oral formulation as soon as they are able to swallow and/or the condition that warranted the use of the IV formulation has resolved.

5.2.2 Dose Modification

The same dose of MK-8228 will be administered for both formulations. Subjects in the MK-8228 treatment group will receive either 240 mg MK-8228 qd, if receiving concomitant CsA, or 480 mg MK-8228 qd (administered as one tablet of 480 mg MK-8228 or two tablets of 240 mg MK-8228), if not on CsA.

As per this protocol amendment, all subjects not on CsA will be initiated with one tablet of 480 mg MK-8228 (or matching placebo). However, subjects not on CsA who were initiated with two tablets of 240 mg MK-8228 (or matching placebo) under the previous protocol version should continue with that regimen.

If CsA is initiated after starting study therapy, the next dose of MK-8228 (administered up to 24 hours later) should be decreased from 480 mg qd to 240 mg qd. If CsA is discontinued permanently or for the long term in a subject already receiving study therapy, the next dose of MK-8228 (administered up to 24 hours later) should be increased from 240 mg qd to 480 mg qd. If CsA is temporarily held due to high levels detected by therapeutic blood monitoring, the dose of MK-8228 need not be adjusted. Depending on CsA co-administration, the number of placebo tablets administered will be the same as MK-8228 tablets, in order to maintain study blind.

5.2.3 Timing of Dose Administration

Study therapy should be administered/taken at the same time each day. Tablets are to be swallowed whole (i.e., no crushing or chewing the tablet is allowed). Study therapy may be administered with or without food.

If a subject misses a dose, the missed dose should be given as soon as possible during the same day. If more than 18 hours have gone by after the regular dosing time, then the missed dose should be skipped and the normal dosing schedule should be resumed. The next dose should not be doubled in order to “make up” what has been missed.

5.2.4 Trial Blinding/Masking

A double-blind/masking technique will be used. Oral MK-8228 and matching placebo (given as oral tablet) will be packaged identically so that treatment blind/masking is maintained. The subject, the investigator and Sponsor personnel or delegate(s) who are involved in the treatment or clinical evaluation of the subjects are unaware of the treatment group assignments.

The oral (tablet) formulations will be packaged identically so that treatment blind/masking is maintained. A placebo image to MK-8228 (both 240 mg and 480 mg) will be implemented to maintain study blinding and placebo tablets will be indistinguishable from MK-8228. Subjects on CsA will receive either one tablet of 240 mg MK-8228 or one tablet of matching placebo. Subjects not on CsA will receive either 480 mg MK-8228 (administered as one tablet of 480 mg MK-8228 or two tablets of 240 mg MK-8228) or one or two tablets of matching placebo.

Table 3 Blinding of Tablets Related to CsA Use

	MK-8228 Group	Placebo Group
On CsA	One 240-mg MK-8228 tablet	One matching placebo tablet
Off CsA	One 480-mg MK-8228 tablet or two 240-mg MK-8228 tablets	One or two matching placebo tablets

IV MK-8228 and matching placebo (given as an IV infusion) will be prepared in a blinded fashion by an unblinded pharmacist (or qualified trial site personnel). The Sponsor will provide opaque covers for the IV bags in order to assist with blinding study therapy.

The IV formulation will be dispensed in a blinded fashion by an unblinded pharmacist (or qualified trial site personnel). Because this is a double-blind study, the investigator, study personnel, and subject must remain blinded to the IV study therapy. In order to maintain the blinding, the unblinded pharmacist (or qualified trial site personnel designated to prepare IV study therapy) will be responsible solely for the preparation and administration of the IV study therapy. He/she will not be involved in evaluating subjects for efficacy or safety.

See Section 7.1.4.2, Blinding/Unblinding, for a description of the method of unblinding a subject during the trial, should such action be warranted.

5.3 Randomization or Treatment Allocation

Randomization will occur centrally using an interactive voice response system/integrated web response system (IVRS/IWRS). There are 2 treatment arms. Subjects will be assigned randomized treatment in an 2:1 ratio to MK-8228 and matching placebo, respectively. With approximately 540 subjects enrolled in this study, ~360 will receive MK-8228 and ~180 will receive placebo.

5.4 Stratification

Randomization will be stratified according to the following factors:

Subjects will be stratified by:

1. Study center, and

2. Risk factor group. Risk factor groups include high or low risk, as defined below:

(i) High risk: Subjects meeting one or more of the following criteria at the time of randomization:

- Human leukocyte antigen (HLA)-related (sibling) donor with at least one mismatch at one of the following three HLA-gene loci: HLA-A, -B or -DR,
- Haploidentical donor,
- Unrelated donor with at least one mismatch at one of the following four HLA-gene loci: HLA-A, -B, -C and -DRB1,
- Use of umbilical cord blood as stem cell source,
- Use of *ex vivo* T-cell-depleted grafts (including *ex vivo* use of alemtuzamab [Campath™]),
- Grade 2 or greater graft-versus-host disease (GVHD), requiring the use of systemic corticosteroids (defined as the use of ≥ 1 mg/kg/day of prednisone or equivalent dose of another corticosteroid).

(ii) Low risk: All subjects not meeting definition of high risk.

5.5 Concomitant Medications/Vaccinations (allowed & prohibited)

Medications or vaccinations specifically prohibited in the exclusion criteria are not allowed during the ongoing trial. Listed below are some specific restrictions for concomitant therapy or vaccination during the course of the trial. If there is a clinical indication for one of these or other medications or vaccinations specifically prohibited during the trial, discontinuation from trial therapy or vaccination may be required. The investigator should discuss any questions regarding this with the local Clinical Monitor. The final decision on any supportive therapy or vaccination rests with the investigator and/or the subject's primary physician. However, the decision to continue the subject on trial therapy or vaccination schedule requires the mutual agreement of the investigator, the Sponsor and the subject.

Medications/therapies listed in this section pertain to coadministration with MK-8228. When used, these agents should be administered in a manner consistent with the local product circular for these agents and dose adjustments are not required due to administration of study therapy.

Allowed Medications/Therapies

The following medications/therapies are **allowed** in this study.

- Standard antimicrobial prophylaxis (e.g., levofloxacin for bacteria, fluconazole/ posaconazole for fungi)

- Acyclovir, valacyclovir, or famciclovir for prophylaxis and treatment of herpes simplex virus (HSV) or varicella zoster virus (VZV) infections at doses no greater than prohibited doses of these medications (see below)
- All types of prior conditioning regimens (including myeloablative, reduced-intensity, or non-myeloablative regimens)
- Prior/Ongoing graft manipulation regimens (including various *ex-vivo* or *in-vivo* T-cell depletion or selection regimens)
- GVHD prophylaxis regimens
- Mycophenolate mofetil

Prohibited Medications/Therapies

The following medications/therapies **for the prevention/treatment of CMV** are **prohibited** while subjects are on study therapy. These agents may however, be used for other indications while subjects are on study therapy e.g. foscarnet for the treatment of HHV-6.

Antiviral drugs or therapies for prevention/treatment of CMV, including but not limited to:

- ganciclovir
- valganciclovir
- foscarnet
- cidofovir
- acyclovir (at doses > 3200 mg PO per day or > 25 mg/kg IV per day)
- valacyclovir (at doses > 3000 mg PO per day)
- famciclovir (at doses > 1500 mg PO per day)
- CMV hyper-immune globulin
- any investigational CMV antiviral agent/biologic therapy, including CMV vaccines

Investigational Agents

Investigational agents are not permitted with the following exceptions: (1) Investigational chemotherapy regimens involving *approved* agents and (2) investigational antimicrobial regimens involving *approved* antibacterial/antifungal/antiviral agents.

Medications/Therapies to be Administered with Caution

The following medications/therapies are not prohibited, but should be **used with caution**.

Clinical drug-drug interaction studies suggest that MK-8228 acts as a weak to moderate inhibitor of cytochrome CYP3A4. Preclinical studies suggest MK-8228 acts as a weak to moderate inhibitor of cytochrome CYP2C8, CYP2B6 and the transporters P-gp, OAT3, OATP1B1 and OATP1B3. It is therefore possible that MK-8228 may increase the exposure of co-administered drugs whose primary route of clearance involves these enzymes or transporters.

CYP3A substrates including but not limited to:

- fentanyl
- amiodarone, flecainide, propafenone, quinidine
- pimozide
- ergot derivatives
- midazolam and triazolam
- sildenafil or tadalafil when used for the treatment of pulmonary arterial hypertension
- drosperinone
- lovastatin and simvastatin
- cisapride
- alfuzosin
- warfarin
- astemizole
- itraconazole
- alfentanil
- felodipine
- vardenafil
- quetiapine
- voriconazole*

**Concomitant use with voriconazole*

A recently completed drug-drug interaction study with MK-8228 and voriconazole (Protocol 025) showed a 44% decrease in voriconazole AUC_{0-12h} and a 39% decrease in voriconazole C_{max} when coadministered with 480 mg of letermovir. Based on these results, the administration of an antifungal other than voriconazole should be considered in subjects for whom antifungal prophylaxis or treatment is required. If coadministration of MK-8228 and voriconazole is necessary, subjects should be monitored closely for breakthrough fungal infections.

CYP2B6 substrates, including but not limited to

- buproprion

CYP2C8 substrates including but not limited to:

- repaglinide
- paclitaxel

OATP1B1, OATP1B3 and OAT3 substrates, including but not limited to:

- statins, e.g., pravastatin, rosuvastatin
- valsartan, telemsartan, olmesartan
- furosemide
- bosentan
- glyburide

Co-administration of medications/therapies known to inhibit P-gP, OATP1B1 and/or OATP1B3 may increase MK-8228 levels. It is therefore possible that MK-8228 levels may increase when co-administered with drugs whose primary route of clearance involves these enzymes. The following medications/therapies are not prohibited, but should be **used with caution**.

P-gP, OATP1B1 and/or OATP1B3 inhibitors due to their potential to increase MK-8228 levels, including but not limited to:

- cyclosporine A (for concomitant cyclosporin A use, a 50% dosage reduction in MK-8228 from 480 mg PO daily to 240 mg PO daily is recommended)
- erythromycin, clarithromycin, azithromycin
- diltiazem, amiodarone, felodipine, verapamil
- itraconazole,
- rifampin
- gemfibrozil
- eltrombopag

Concomitant Immunosuppressant Use

Levels of cyclosporine and tacrolimus have been shown to increase ~ 2 fold with MK-8228 co-administration. Therefore, levels of cyclosporine, tacrolimus, sirolimus and everolimus should be closely monitored by the investigator, especially when initiating study therapy or changing the dosage of study therapy, as dose adjustments of these immunosuppressant agents may be needed when co-administered with MK-8228. A recently completed Phase I trial demonstrated that mycophenolate mofetil and MK-8228 may be coadministered without dose adjustment of either agent. All drug-drug interactions for co-administered immunosuppressant agents, as per the local prescribing information for these agents, are also applicable in this study; therefore it is also important for the investigator to monitor the use of any other co-administered immunosuppressants and ensure that these therapies are administered according to local product circulars.

5.6 Rescue Medications & Supportive Care

In the event of clinically significant CMV infection (CMV disease or initiation of PET based on CMV viremia and the clinical condition of the subject [see Section 4.2.3.1]) at any time during the 48 week post-transplant period, study therapy will be discontinued (if the subject is on study therapy) and the subject may be treated according to the local standard of care (outside the context of the study). In this setting, any of the prohibited anti-CMV medications (outlined in Section 5.5) may be used.

5.7 Diet/Activity/Other Considerations

Study therapy can be administered with or without food.

Subjects must avoid consumption of grapefruit, Seville oranges or their respective juices, and other quinine-containing drinks or food during the trial from 2 weeks prior to study drug administration until 72 hours after the final administration of study drug.

5.8 Subject Withdrawal/Discontinuation Criteria

Subjects may withdraw consent at any time for any reason or be dropped from the trial at the discretion of the investigator should any untoward effect occur. In addition, a subject may be withdrawn by the investigator or the Sponsor if enrollment into the trial is inappropriate, the trial plan is violated, or for administrative and/or other safety reasons. Specific details regarding discontinuation or withdrawal procedures; including specific details regarding withdrawal from Future Biomedical Research, are provided in Section 7.1.4 – Other Procedures.

In this trial, a subject may discontinue from treatment but continue to participate in the regularly scheduled activities, as long as the subject does not withdraw consent. Note on reinitiation of study therapy: There may be instances where confirmatory central lab test results for CMV DNA PCR obtained on the day of PET initiation may be negative (CMV DNA undetectable) and the investigator may wish to discontinue PET. The decision to stop PET in the event of a negative (CMV not detectable) confirmatory central laboratory result collected on the day of PET initiation resides with the investigator caring for the patient. Therefore, in the event the confirmatory CMV DNA sample at PET initiation is negative for CMV viremia, the Sponsor will allow for protocol-defined study therapy (i.e., letermovir or placebo, based on initial randomization) to be restarted at the investigator's discretion, once PET is discontinued. In such instance, study therapy should be restarted within 7 days from the date on which study therapy was stopped.

A subject must be discontinued from the trial for any of the following reasons:

- The subject or legal representative (such as a parent or legal guardian) withdraws consent.

A subject **must** be discontinued from study therapy (<u>but should continue to be monitored in the study</u>) for any of the following reasons:

- The subject meets the criteria for clinically significant CMV infection (see Section 4.2.3.1).
- The subject becomes pregnant during the study.
- The subject's investigator feels it is in the best interest of the subject to discontinue.
- The subject **may** be discontinued from study therapy for any of the following reasons:
  - Any AE/SAE assessed by the physician investigator as possibly or probably related to study therapy. Investigator may continue the subject in the trial if it is deemed to be in the best interest of the subject to stay on study therapy.
  - Failure to comply with the dosing, evaluations, or other requirements of the trial.

- The subject has a medical condition or personal circumstance which, in the opinion of the investigator and/or Sponsor, places the subject at unnecessary risk through continued participation in the trial or does not allow the subject to adhere to the requirements of the protocol.

For subjects who develop clinically significant CMV infection during the study treatment period (up to Week 14 post-transplant): When the investigator intends to initiate either treatment for CMV disease or PET, the subject should have a CMV Infection Visit. It is very important to ensure that all procedures, as outlined in the Trial Flow Chart (Section 6.0), are performed at the CMV Infection Visit **immediately prior to** the initiation of treatment of CMV diseases or initiation of PET (i.e., on the day anti-CMV therapy is initiated). Most importantly, a confirmatory plasma sample for CMV PCR testing at the central laboratory should be collected at this visit. Thereafter, the subject should be discontinued from study therapy and treated according to the local standard of care (outside the context of the study). Such subjects will however, continue to be followed in the study (despite discontinuing study therapy and initiating anti-CMV therapy) and complete all remaining study visits (including all subsequent treatment period visits). At such subsequent visits, all procedures specified in the Trial Flow Chart should be completed with the exception of study therapy administration, pharmacokinetic (PK) assessments, and study medication diary review.

Note on reinitiation of study therapy: There may be instances where confirmatory central lab test results for CMV DNA PCR obtained on the day of PET initiation may be negative (CMV DNA undetectable) and the investigator may wish to discontinue PET. The decision to stop PET in the event of a negative (CMV not detectable) confirmatory central laboratory result collected on the day of PET initiation resides with the investigator caring for the subject. Therefore, in the event the confirmatory CMV DNA sample at PET initiation is negative for CMV viremia, the Sponsor will allow for protocol-defined study therapy (i.e., letermovir or placebo, based on initial randomization) to be restarted at the investigator's discretion, once PET is discontinued. In such instances, study therapy should be restarted within 7 days from the date on which study therapy was stopped. It is important to note that the status of the subject's study therapy in IVRS should NOT be changed until the CMV DNA PCR result is confirmed and the investigator is certain that study therapy will be permanently discontinued.

For subjects with clinically significant CMV infection during the post-treatment [follow-up] period (after Week 14 and through Week 24 post-transplant): When an investigator intends to initiate either treatment for CMV disease or PET, the subject should have a CMV Infection Visit. It is very important to ensure that all procedures, as outlined in the Trial Flow Chart (Section 6.0), are performed at the CMV Infection Visit **immediately prior to** the initiation of treatment of CMV diseases or initiation of PET (i.e., on the day anti-CMV therapy is initiated). Most importantly, a confirmatory plasma sample for CMV PCR testing at the central laboratory should be collected at this visit. Thereafter, the subject can be treated according to the local standard of care (outside the context of the study). Such subjects will however, continue to be followed in the study (despite initiation of anti-CMV therapy) and complete all remaining study visits. At such subsequent visits, all procedures specified in the Trial Flow Chart should be completed.

Note: It is mandatory to send a confirmatory plasma sample for CMV DNA PCR testing to the central laboratory **immediately prior to** (i.e., on the day of) initiating treatment for CMV disease or PET in **ALL** instances. In the event that the confirmatory result obtained on the day of PET initiation is **NOT** available (e.g., sample is lost or mishandled by the investigator site prior to shipment, or is inadequate upon receipt at the central laboratory), a subsequent sample must be obtained and sent to the central laboratory within 7 days after PET initiation (preferably within 48-72 hours). The subject will be considered to have met the primary efficacy endpoint if this confirmatory laboratory result is positive.

In the event confirmatory test results from the central laboratory are not available within the timeframe the investigator wishes to initiate anti-CMV therapy, the investigator may use a positive local laboratory test result (CMV DNA PCR or pp65 antigen only) in order to make the decision. However, as described above, plasma samples for CMV DNA PCR testing must also be sent to the central laboratory. The local laboratory result must also be reported in such instances.

Subjects who are discontinued from the study for any reason will not be replaced. Subjects who discontinue the study early up to Week 24 post-transplant should complete an Early Discontinuation Visit. Subjects who discontinue the study early after Week 24 post-transplant should have the procedures scheduled for the Week 48 post-transplant visit completed at the time of discontinuation from the study.

5.9 Subject Replacement Strategy

A subject that discontinues from the trial will not be replaced.

5.10 Beginning and End of the Trial

The overall trial begins when the first subject signs the informed consent form. The overall trial ends when the last subject completes the last trial visit, discontinues from the trial or is lost to follow-up (i.e. the subject is unable to be contacted by the investigator).

5.11 Clinical Criteria for Early Trial Termination

Early trial termination will be the result of the criteria specified below:

1. The trial is deemed to be futile based on the results of the interim analysis for futility.
2. The EOC determines that the extent (incidence and/or severity) of emerging adverse effects/clinical endpoints is such that the risk/benefit ratio to the trial population as a whole is unacceptable.

6.0 TRIAL FLOW CHART

Visit No.	1	2	3	4	5	6	7	8	9	10	11	12	13	14	15	16	17	18	19	20	21	22	23	24	25
	Screen	Treatment (Week 1)		Treatment (Weeks 2 to 14[c])													Post-treatment Follow-up (Weeks 15 to 24[c])					Post-treatment Follow-up (Weeks 25 to 48[c])			
Week No. (unless otherwise indicated)	SCR[a]	Day 1[b]	Day 7	2	3	4	5	6	7	8	9	10[d]	11[d]	12[d]	13[d]	End of Study Therapy Visit 14[d]	16	18	20	22	24	32	40	48	CMV Infection or Early Discon Visit[e]
Follow-up (FU) Week No.																	FU2	FU4	FU6	FU8	FU10	FU18	FU26	FU34	
Visit Window			±2 days	±3 days													±4 days					±2 weeks			
**Administrative Procedures**																									
Informed Consent[f]	X																								
Informed Consent for Future Biomedical Research (optional)	X																								
Inclusion/Exclusion Criteria	X	X																							
Subject Identification Card	X																								
Medical History	X																								
Prior/Concomitant Medication Review[g]	X	X	X	X	X	X	X	X	X	X	X	X	X	X	X	X	X	X	X	X	X	X	X	X	X
Study Therapy Allocation/ Randomization		X																							
HSCT Details Review[h]		X																							
Study Medication Diary Review[i]		X	X	X	X	X	X	X	X	X	X	X	X	X	X	X									X
**Clinical Procedures/ Assessments**																									
Physical Examination[j]	X	X	X	X	X	X	X	X	X	X	X	X	X	X	X	X	X								X
Weight	X															X									X
Height	X																								

Visit No.	1	2	3	4	5	6	7	8	9	10	11	12	13	14	15	16	17	18	19	20	21	22	23	24	25
	Screen	Treatment (Week 1)		Treatment (Weeks 2 to 14[c])													Post-treatment Follow-up (Weeks 15 to 24[c])					Post-treatment Follow-up (Weeks 25 to 48[c])			
Week No. (unless otherwise indicated)	SCR[a]	Day 1[b]	Day 7	2	3	4	5	6	7	8	9	10[d]	11[d]	12[d]	13[d]	End of Study Therapy Visit 14[d]	16	18	20	22	24	32	40	48	CMV Infection or Early Discon Visit[e]
Follow-up (FU) Week No.																	FU2	FU4	FU6	FU8	FU10	FU18	FU26	FU34	
Visit Window			±2 days	±3 days													±4 days					±2 weeks			
Vital Signs (heart rate, blood pressure, respiratory rate, body temperature)[k]	X	X	X	X	X	X	X	X	X	X	X	X	X	X	X	X	X								X
12-Lead Electrocardiogram[l]	X			X												X									
Child-Pugh Score (see Appendix 12.5)	X																								
Subject Confirmation of Birth Control[m]	X	X	X	X	X	X	X	X	X	X	X	X	X	X	X	X	X	X	X	X	X				X
Adverse Events Monitoring[n]	X	X	X	X	X	X	X	X	X	X	X	X	X	X	X	X	X	X	X	X	X	X	X	X	X
**Laboratory Procedures/ Assessments[o]**																									
Chemistry/ Hematology	X[p]	X		X		X				X				X		X	X								X
Urinalysis	X[p]	X														X									X
Serum β-Human Chorionic Gonadotropin (β-hCG) in women of childbearing potential	X																								
Urine Pregnancy Test in women of childbearing potential		X				X				X						X									X
Serum inhibin B,		X														X					X				X

Visit No.	1	2	3	4	5	6	7	8	9	10	11	12	13	14	15	16	17	18	19	20	21	22	23	24	25
	Screen	Treatment (Week 1)		Treatment (Weeks 2 to 14[c])													Post-treatment Follow-up (Weeks 15 to 24[c])					Post-treatment Follow-up (Weeks 25 to 48[c])			
Week No. (unless otherwise indicated)	SCR[a]	Day 1[b]	Day 7	2	3	4	5	6	7	8	9	10[d]	11[d]	12[d]	13[d]	End of Study Therapy Visit 14[d]	16	18	20	22	24	32	40	48	CMV Infection or Early Discon Visit[e]
Follow-up (FU) Week No.																	FU2	FU4	FU6	FU8	FU10	FU18	FU26	FU34	
Visit Window			±2 days	±3 days													±4 days					±2 weeks			
LH, FSH, testosterone levels in men																									
HIV/Hepatitis B, C Screen[q]	X																								
Blood for Genetics[r]		X																							
**CMV Procedures/ Assessments**																									
CMV DNA PCR[s]	X	X	X	X	X	X	X	X	X	X	X	X	X	X	X	X	X	X	X	X	X	X	X	X	X
CMV Disease Assessment (see Appendix 12.4)	X	X	X	X	X	X	X	X	X	X	X	X	X	X	X	X	X	X	X	X	X	X	X	X	X
Health Outcomes Assessment[t]		X				X				X						X	X		X		X	X	X	X	X
Quality of Life Assessment[u]		X														X					X			X	X
CMV DNA Sequence Analysis[v]																									X
**Pharmacokinetics**																									
Population PK[w]		X	X	X		X		X		X		X		X		X									
Intensive PK[x]			X																						
**Study Therapy Administration**																									
MK-8228/ Placebo[y,z]		X	X	X	X	X	X	X	X	X	X	X	X	X	X	X									

a. Subjects may be screened during a period starting from 15 days prior to transplantation through 28 days post-transplant. Screening test results must be available within 5 days prior to the planned randomization date. Subjects will have plasma samples tested for CMV viremia using the CMV DNA PCR assay (for initial screening purposes, results of the assay done at a local laboratory will be acceptable). After establishing absence of CMV viremia, subjects will be tested once a week by the central laboratory using the CMV DNA PCR assay until randomization (Day 1). The following are to be assessed within 5 days prior to randomization: Child Pugh Score; serum AST, ALT, and total bilirubin; and creatinine clearance.

b. Start of study therapy is Day 1. On the day of randomization, eligibility for enrollment into the study should be confirmed (including confirmation that HSCT has taken place). At that time, subjects should have **NO** documented CMV viremia, as confirmed by CMV DNA PCR assay at the central laboratory (the Roche COBAS® AmpliPrep/COBAS TaqMan® [CAP/CTM] assay) in a plasma sample collected within 5 days prior to randomization**. (NOTE: Evidence of CMV viremia as reported by the central lab will include reporting of test results as "detectable, not quantifiable" or "detected" with a numeric value provided.)** Creatinine clearance and liver function test results within 5 days prior to randomization should also be available and be within the range allowable in this study, as outlined in Section 5.1.3 (Subject Exclusion Criteria). Study therapy may begin as early as the day of transplant and no later than 28 days post-transplant. Study therapy will continue through Week 14 (~100 days) post-transplant. Day 1 procedures/assessments must be performed prior to first dose of study therapy.

c. Weeks correspond to the end of the numbered weeks after randomization, i.e., Week 2, 3, 4, etc. corresponds to Days 14, 21, 28 after randomization, respectively.

d. End of Study Therapy Visit may occur on Week 10, 11, 12, 13, or 14 depending on when study therapy was started during the 28-day post-transplant window. For example, if study therapy was started on the day of transplant, the End of Study Therapy Visit would be the Week 14 Visit (which corresponds to Week 14 post-transplant). If study therapy was started 28 days post-transplant, the End of Study Therapy Visit would be the Week 10 Visit (which corresponds to Week 14 post-transplant). Any per protocol procedure listed under the Week 14 Visit should be conducted at the true End of Study Therapy Visit (at Week 10, 11, 12, 13, or 14 post-transplant, depending on when study therapy was started).

e. The visit will be a CMV Infection Visit for all subjects who discontinue study therapy due to clinically significant CMV infection defined as the occurrence of CMV disease or the initiation of PET, or for subjects who are either diagnosed with CMV disease or require initiation of PET after study therapy completion during the follow-up period (through Week 24 post-transplant). The visit will be an Early Discontinuation Visit for those subjects who are prematurely discontinued from the trial up to Week 24 post-transplant. All procedures should be performed at this visit **immediately prior to** the initiation of treatment of CMV diseases or initiation of PET (i.e., on the day anti-CMV therapy is initiated). Most importantly, a confirmatory plasma sample for CMV PCR testing at the central laboratory should be collected at this visit. In the event that the confirmatory result obtained on the day of PET initiation is **NOT** available (e.g., sample is lost or mishandled by the investigator site prior to shipment, or is inadequate upon receipt at the central laboratory), a subsequent sample must be obtained and sent to the central laboratory within 7 days after PET initiation (preferably within 48-72 hours). The subject will be considered to have met the primary efficacy endpoint if this confirmatory laboratory result is positive.

f. The informed consent must be obtained before any study-specific procedure is performed. It is acceptable that the date of obtaining informed consent is earlier than the day of performing screening procedures. However, once informed consent is obtained adverse event reporting must be conducted according to Section 7.1.2.7.

g. Includes review of consumption of grapefruit, Seville oranges or their respective juices, and other quinine-containing drinks or food. **Anti-CMV medications administered for treatment of CMV disease or for initiation of PET and all drug/biologic therapies used to prevent/treat GVHD should be recorded at every visit through Week 48 post-transplant.** Prior to Week 14 post-transplant all concomitant medications should be reviewed and documented. During the follow-up period (after Week 14 through Week 48 post-transplant), concomitant medication review is limited to the above and all antimicrobials (antibacterials, antifungals, antivirals), chemotherapy agents, and immunosuppressant agents.

h. To collect all relevant data at randomization (Day 1) related to the recent HSCT including details regarding the conditioning regimen used, the date and type of transplant, the source of stem cells, type of graft manipulation, presence of GVHD, and GVHD prophylaxis regimen (if any) used.

i. Study Medication Diary completion and review will begin once subject is discharged from the hospital.

j. After randomization (Day 1), the physical examination does not need to be performed at every visit; a targeted physical exam should be performed only if a subject has any complaints.

k. Vital signs include heart rate (sitting), blood pressure (sitting), respiratory rate, and body temperature (oral). Subjects should be resting in a semi-recumbent position for at least 10 minutes prior to measurement of vital signs. After randomization (Day 1), vital signs should only be performed if targeted physical examination is performed.

l. All 12-Lead ECGs will be obtained after the subject has remained in a semi-recumbent position for 10 minutes.

m. Subject must use acceptable methods of contraception from the time of consent through 90 days after the last dose of study therapy.

n. Adverse event (AE) monitoring prior to randomization (Day 1) is limited to AEs resulting from protocol-related procedures or interventions, those resulting in death, and those resulting in a subject not being randomized. After initiating study therapy, all AEs should be collected from randomization (Day 1) through Follow-up Week 2 visit (i.e. through Week 16 post-transplant) in all subjects. Thereafter, only drug related serious adverse events (SAEs) and SAEs leading to death will be collected from week 16 through Week 48 post-transplant. Refer to Section 7.2 (Assessing and Recording Adverse Events) for further details. Safety monitoring of infusion-site adverse events should be performed by the evaluation of the site of infusion during and at the end of IV study therapy. Events should be entered on the AE electronic case report form. The trial site guidance for assessment and follow-up for infusion-site adverse events can be found in the Investigator Trial File Binder.

o. Refer to Section 7.1.3 (Laboratory Procedures/Assessments) for further details regarding the laboratory safety tests. Laboratory safety tests may be conducted within 3 days prior to a scheduled study visit and no later than the day of the scheduled study visit.

p. For screening purposes, values from the subject's chart for required chemistry, hematology, and urinalysis tests are acceptable. If not available, this testing may be performed by the central laboratory.

q. Perform /Hepatitis B, C Screen only if not previously documented within the last 90 days. If hepatitis C virus antibody is positive, RNA PCR results should be provided (or, if not available, RNA PCR testing will be performed by the central laboratory). HIV antibody test results documented at any time prior to randomization of the subject will be acceptable. A copy of the report must be available. If documentation of a previous HIV test is not available, the HIV antibody test must be conducted using the central laboratory.

r. This sample will be drawn for *SLCO1B1* (OATP1B1) genotyping and for planned genetic analysis of DNA and drug response. Data analysis will be limited to *SLCO1B1* (OATP1B1) genotyping if there is either a documented law or regulation prohibiting the planned genetic analysis of DNA and drug response, or if the IRB/IEC does not approve of the planned genetic analysis of DNA and drug response. Any leftover extracted DNA will be stored for future biomedical research if the subject signs the FBR consent.

s. For initial screening, results of CMV DNA PCR assay performed at a local laboratory will be acceptable to establish absence of CMV viremia. After the Screening Visit until randomization, CMV DNA PCR testing will be performed once a week by the central laboratory using the Roche CAP/CTM Assay. Thereafter, CMV DNA PCR testing will be performed by the central laboratory at every visit as specified in the Flow Chart. The CMV DNA PCR test may be conducted within 3 days prior to a scheduled study visit and no later than the day of the scheduled study visit. (This CMV DNA PCR testing window is not applicable to a CMV Infection Visit. See note below.) Plasma samples for CMV PCR testing at the central laboratory will also be collected at the CMV Infection or Early Discontinuation Visit. Note: It is mandatory to send a confirmatory plasma sample for CMV DNA PCR testing to the central laboratory immediately prior to (i.e., on the day of) initiating treatment for CMV disease or PET in ALL instances. In the event that the confirmatory result obtained on the day of PET initiation is **NOT** available (e.g., sample is lost or mishandled by the investigator site prior to shipment, or is inadequate upon receipt at the central laboratory), a subsequent sample must be obtained and sent to the central laboratory within 7 days after PET initiation (preferably within 48-72 hours). Any results of local laboratory CMV PCR must also be reported. If the subject consents to the Future Biomedical Research sub-study, any leftover samples from CMV DNA PCR will be stored for future research.

t. To collect information such as all-cause mortality, re-hospitalizations, GVHD, opportunistic infections (e.g. systemic bacterial and invasive fungal infections), and engraftment.

u. The EuroQol (EQ)-5D and Functional Assessment of Cancer Therapy – Bone Marrow Transplant (FACT-BMT) questionnaires which are validated patient reported outcome tools will be used to measure quality of life.

v. CMV DNA Sequence Analysis to be performed only in subjects with clinically significant CMV infection.

w. Population (sparse) PK samples will be collected in all subjects. The 9 samples at the indicated visits will be collected 0 – 2 hours pre-dose. As treatment may range 10 – 14 weeks, the Week 12 and Week 14 visit samples may not be collected in all subjects.

x Intensive PK sampling will be performed in a subset of ~100 subjects. The 5 samples will be collected at the Day 7 visit at the following time points: pre-dose, 1 hour (± 10 min) following oral administration (or within less than 10 min **after** infusion completion, when given IV), 2.5 hours following oral/IV administration (± 30 min), 8 hours following oral/IV administration (range of 6-10 hours), and 24 hours following oral/IV administration (range of 22-24 hours; 0-2 hours prior to next day's dose). All identified subjects who choose to participate in intensive PK sampling will sign an additional informed consent prior to the collection of samples.

y. Both oral (tablet) and intravenous (IV) formulation of MK-8228 (and placebo) will be available for study therapy. Subjects will be initiated with the oral (tablet) formulation of study therapy provided they are able to swallow and do not have a condition (e.g., vomiting, diarrhea, or malabsorptive condition) that may interfere with the absorption of the tablets. For subjects who cannot swallow and/or have a condition that may interfere with the absorption of the oral formulation, study therapy can be initiated with or switched to the IV formulation. The IV formulation should be switched to oral study therapy (i.e., at the next planned dose) as soon as such subjects are able to swallow and/or the condition necessitating the use of the IV formulation resolves. Use of the IV formulation should generally be limited to 4 weeks or less in duration. However, it will be left to the investigator's

	discretion to continue IV administration beyond 4 weeks, if the benefit/risk ratio supports continued administration.
z.	In this study, the dose of MK-8228 will change based on whether concomitant CsA is received. The same dose of MK-8228 will be administered for both formulations. Subjects in the MK-8228 treatment group will receive either 240 mg MK-8228 qd, if receiving concomitant CsA, or 480 mg MK-8228 qd, if not on CsA. If CsA is initiated after starting study therapy, the next dose of MK-8228 (administered up to 24 hours later) should be decreased to 240 mg qd. If CsA is discontinued permanently or for the long-term in a subject, the next dose of MK-8228 (administered up to 24 hours later) should be increased from 240 mg to 480 mg qd. If CsA is temporarily held due to high levels detected by therapeutic blood monitoring, the dose of MK-8228 need not be adjusted. Corresponding changes in tablets for oral formulation with changes in CsA dosing will also occur in the placebo group in an effort to maintain the study blind (see Section 5.2.3).

7.0 TRIAL PROCEDURES

7.1 Trial Procedures

The Trial Flow Chart - Section 6.0 summarizes the trial procedures to be performed at each visit. Individual trial procedures are described in detail below. It may be necessary to perform these procedures at unscheduled time points if deemed clinically necessary by the investigator.

Furthermore, additional evaluations/testing may be deemed necessary by the investigator and or the Sponsor for reasons related to subject safety. In some cases, such evaluation/testing may be potentially sensitive in nature (e.g., HIV, Hepatitis C, etc.), and thus local regulations may require that additional informed consent be obtained from the subject. In these cases, such evaluations/testing will be performed in accordance with those regulations.

7.1.1 Administrative Procedures

7.1.1.1 Informed Consent

The investigator must obtain documented consent from each potential subject prior to participating in a clinical trial or Future Biomedical Research.

7.1.1.1.1 General Informed Consent

Consent must be documented by the subject's dated signature or by the subject's legally acceptable representative's dated signature on a consent form along with the dated signature of the person conducting the consent discussion.

A copy of the signed and dated consent form should be given to the subject before participation in the trial.

The initial informed consent form, any subsequent revised written informed consent form and any written information provided to the subject must receive the IRB/ERC's approval/favorable opinion in advance of use. The subject or his/her legally acceptable representative should be informed in a timely manner if new information becomes available that may be relevant to the subject's willingness to continue participation in the trial. The communication of this information will be provided and documented via a revised consent form or addendum to the original consent form that captures the subject's dated signature or by the subject's legally acceptable representative's dated signature.

Specifics about a trial and the trial population will be added to the consent form template at the protocol level.

The informed consent will adhere to IRB/ERC requirements, applicable laws and regulations and Sponsor requirements.

7.1.1.1.2 Consent and Collection of Specimens for Future Biomedical Research

The investigator or qualified designee will explain the Future Biomedical Research consent to the subject, answer all of his/her questions, and obtain written informed consent before performing any procedure related to the Future Biomedical Research sub-trial. A copy of the informed consent will be given to the subject.

7.1.1.2 Inclusion/Exclusion Criteria

All inclusion and exclusion criteria will be reviewed by the investigator or qualified designee to ensure that the subject qualifies for the trial at screening and on Day 1 (randomization).

7.1.1.3 Subject Identification Card

All subjects will be given a Subject Identification Card identifying them as participants in a research trial. The card will contain trial site contact information (including direct telephone numbers) to be utilized in the event of an emergency. The investigator or qualified designee will provide the subject with a Subject Identification Card after the subject provides written informed consent.

7.1.1.4 Medical History

A medical history will be obtained by the investigator or qualified designee at screening.

7.1.1.5 Prior and Concomitant Medications Review

7.1.1.5.1 Prior Medications

The investigator or qualified designee will review prior medication use, including any protocol-specified washout requirement, and record prior medication taken by the subject within 30 days prior to screening. This includes review of consumption of grapefruit, Seville oranges or their respective juices, and other quinine-containing drinks or food.

7.1.1.5.2 Concomitant Medications

The investigator or qualified designee will record medication, if any, taken by the subject during the trial. Anti-CMV medications administered for treatment of CMV disease or for initiation of PET and all drug/biologic therapies used to prevent/treat GVHD should be recorded at every visit through Week 48 post-transplant. During the follow-up period (after Week 14 through Week 48 post-transplant), concomitant medication review is limited to the above and all antimicrobials (antibacterials, antifungals, antivirals), chemotherapy agents, and immunosuppressant agents.

7.1.1.6 HSCT Details Review

All relevant data about the HSCT will be collected on Day 1 (at randomization). This includes details regarding the conditioning regimen used, the date and type of transplant, the source of stem cells, type of graft manipulation, presence of GVHD, and GVHD prophylaxis regimen (if any) used.

7.1.1.7 Assignment of Screening Number

All consented subjects will be given a unique screening number that will be used to identify the subject for all procedures that occur prior to randomization or treatment allocation. Each subject will be assigned only one screening number. Screening numbers must not be re-used for different subjects. Any subject who is screened multiple times will retain the original screening number assigned at the initial screening visit.

7.1.1.8 Assignment of Randomization Number

All eligible subjects will be randomly allocated to trial treatment and will receive a randomization number. The randomization number identifies the subject for all procedures occurring after randomization. Once a randomization number is assigned to a subject, it can never be re-assigned to another subject.

A single subject cannot be assigned more than 1 randomization number.

7.1.1.9 Trial Compliance (Study Therapy)

The Study Medication Diary will be completed electronically via a hand-held device once the subject is discharged from the hospital. The investigator/study coordinator will train the subject in the use of the electronic Study Medication Diary. The subject will be instructed to enter the number of tablets of study therapy taken during the study therapy period. At visits when used/unused study therapy are returned, site personnel must verify the accuracy of the dosing diary by comparing entries with amounts of returned study therapy. If a discrepancy is noted, the investigator/study coordinator must discuss the discrepancy with the subject, and the explanation must be documented. The investigator/study coordinator will be responsible for transferring the appropriate information from the Study Medication Diary to the appropriate case report form.

Study therapy may be interrupted for any reason for a time period of <7 consecutive days.

Interruptions from the protocol specified treatment plan for ≥7 consecutive days require consultation between the investigator and the Sponsor and written documentation of the collaborative decision on subject management.

7.1.2 Clinical Procedures/Assessments

7.1.2.1 Physical Examination

All physical examinations must be performed by the principal investigator or subinvestigator (physician, physician assistant or nurse practitioner).

A complete physical examination, performed at the screening visit and Day 1 (randomization), includes the following assessments: general appearance, head, eyes, ears/nose/throat, neck, lymph nodes, skin, lungs, heart, abdomen, musculoskeletal, and neurologic evaluations. Breast, rectal, and genitourinary/pelvic exams should be performed when clinically indicated.

After randomization (Day 1), the physical examination does not need to be performed at every visit; a targeted physical exam should be performed only if a subject has any complaints. The timing of physical examinations is indicated in the Trial Flow Chart (Section 6.0).

7.1.2.2 Weight and Height Assessment

The subject's weight and height will be assessed as indicated in the Trial Flow Chart (Section 6.0).

7.1.2.3 Vital Signs

Vital signs will be assessed as indicated in the Trial Flow Chart (Section 6.0).

Vital signs will include heart rate (sitting), blood pressure (sitting), respiratory rate, and body temperature (oral). Subjects should be resting in a semi-recumbent position for at least 10 minutes prior to measurement of vital signs.

Note: Oral temperatures should be taken orally, but if oral is not possible, tympanic, rectal, and axillary temperatures are acceptable.

After Day 1 (randomization), vital signs should only be performed if targeted physical examination is performed.

7.1.2.4 12-Lead Electrocardiogram

12-Lead ECG measurements will be performed using a central vendor as indicated in the Trial Flow Chart (Section 6.0).

Special care must be taken for proper lead placement. Subjects should be shaved as necessary for proper lead placement. Subjects should be resting in a semi-recumbent position for at least 10 minutes prior to having ECG readings obtained.

7.1.2.5 Child Pugh Score

The Child Pugh Score will be assessed at screening and within 5 days prior to randomization according to Appendix 12.5.

7.1.2.6 Birth Control Confirmation

Subjects must use acceptable methods of contraception from the time of consent through 90 days after the last dose of study therapy. Confirmation must be obtained by site personnel that subjects and their partner(s) are using acceptable methods of contraception. This assessment must be documented in the subject's study chart at each specified visit.

7.1.2.7 Adverse Events Monitoring

From the time informed consent is signed until randomization, the following adverse events only should be reported: those resulting from protocol-specified procedures or intervention, those resulting in death, and those resulting in a subject not being randomized. After initiation of study therapy, adverse event monitoring will include the collection of all adverse events through Follow-up Week 2 Visit (16 weeks post-transplant) in all subjects, including those who have discontinued study therapy but continue to be followed-up in the study. Thereafter, only drug related serious adverse events (SAEs) and SAEs leading to death will be collected through Week 48 post-transplant. Refer to Section 7.2 (Assessing and Recording Adverse Events) for further details.

Infusion-Site Adverse Events for Subjects Administered IV Study Therapy

Safety monitoring of infusion-site adverse events will be performed by the evaluation of the site of infusion during and at the end of IV study therapy. Events will be entered on the adverse events electronic case report form. The trial site guidance for assessment and follow-up for infusion-site adverse events can be found in the Investigator Trial File Binder.

7.1.2.8 CMV Disease Assessment

CMV disease will be assessed from screening through Week 48 post-transplant. Diagnostic criteria for the evaluation of CMV disease are outlined in Appendix 12.4. The investigator will ensure that clinical information, radiology results, and specimens for the appropriate diagnostic tests (including, but not limited to, viral culture, histopathology, immunohistochemical analysis, *in situ* hybridization, CMV PCR) as outlined in Appendix 12.4 will be collected.

7.1.2.9 Health Outcomes Assessment

Information such as all-cause mortality, re-hospitalizations, GVHD, opportunistic infections (i.e., systemic bacterial and invasive fungal infections), and engraftment will be collected as part of the Health Outcomes Assessment for this study as indicated in the Trial Flow Chart (Section 6.0).

7.1.2.10 Quality of Life Assessment

Two questionnaires, the EuroQol (EQ)-5D version 3L and the Functional Assessment of Cancer Therapy – Bone Marrow Transplant (FACT-BMT) version 4, are validated tools of patient reported outcomes. These 2 questionnaires will be used to assess quality of life (QoL). These QoL assessments will be collected at the time points indicated in the Trial Flow Chart (Section 6.0).

The EQ-5D consists of five general health questions and a visual analog scale.

The FACT-BMT comprises 47 questions measuring the following domains: physical well-being, social/family well-being, emotional well-being, functional well-being and additional concerns related to the subject's clinical condition.

These measures will be completed electronically via a hand-held device. The investigator/study coordinator will train the subject in the use of the device. The subject will be instructed to complete these measures at the designated visits prior to any study procedures. The investigator/study coordinator will also confirm that the measures have been completed prior to performing any study procedures.

7.1.3 Laboratory Procedures/Assessments

Details regarding specific laboratory procedures/assessments to be performed in this trial are provided below. The total amount of blood/tissue to be drawn/collected over the course of the trial (from pre-trial to post-trial visits), including approximate blood/tissue volumes drawn/collected by visit and by sample type per subject can be found in Section 12.6.

7.1.3.1 Laboratory Safety Evaluations (Hematology, Chemistry and Urinalysis)

The specific laboratory tests for hematology, chemistry and urinalysis to be performed within this study are specified in Table 4. The time points for these assessments are indicated in the Trial Flow Chart (Section 6.0) and may be conducted within 3 days prior to the scheduled study visit and no later than the day of the scheduled study visit.

Table 4 Laboratory Tests

Hematology[a]	Chemistry[a]	Urinalysis[a]	Other
Hematocrit	Albumin	Blood	Follicle Stimulating Hormone (FSH), luteinizing hormone (LH), testosterone and inhibin B levels in males
Hemoglobin	Alkaline phosphatase	Glucose	Serum β-human chorionic gonadotropin (β-hCG)
Platelet count	Alanine aminotransferase (ALT)	Protein	Urine β-human chorionic gonadotropin (β-hCG)
WBC (total and differential)	Aspartate aminotransferase (AST)	Specific gravity	Hepatitis B surface antigen (HBsAg)[b]
	Bicarbonate	Microscopic exam, if abnormal results are noted	Hepatitis C virus antibody (HCV-Ab)[b]
	Calcium		Hepatitis C RNA PCR[b]
	Chloride		HIV antibody (HIV-Ab)[b]
	Creatinine		CMV DNA PCR[c]
	Creatinine Clearance (screening only)		CMV DNA Sequence Analysis[d]
	Glucose		
	Phosphorus		
	Potassium		
	Sodium		
	Total Bilirubin		
	Direct Bilirubin		
	Indirect Bilirubin		
	Total protein		
	Blood Urea Nitrogen		
	Prothrombin time (PT)<br>International normalized ratio (INR)		

a For screening, values from the subject's chart for required chemistry, hematology, and urinalysis tests are acceptable. If not available, this testing may be performed by the central laboratory.
b Hepatitis B, C testing only performed if results not previously documented within 90 days of screening. If hepatitis C virus antibody is positive, RNA PCR results should be provided (or, if not available, RNA PCR testing will be performed by the central laboratory). HIV antibody test results documented at any time prior to randomization of the subject will be acceptable. A copy of the report must be available. If documentation of a previous HIV test is not available, the HIV antibody test must be conducted using the central laboratory.
c For initial screening, results of CMV DNA PCR assay performed at a local laboratory will be acceptable to establish absence of CMV viremia. Thereafter, CMV DNA PCR testing will be performed by the central laboratory using the Roche CAP/CTM Assay.
d CMV DNA Sequence Analysis to be performed only in subjects with clinically significant CMV infection.

7.1.3.2 Pharmacokinetic/Pharmacodynamic Evaluations

7.1.3.2.1 Blood Collection for Pharmacokinetic Sampling

Sample collection, storage, and shipment instructions for plasma samples will be provided in the operations/laboratory manual.

Population (sparse) PK samples will be collected in all subjects. The 9 samples will be collected 0-2 hours pre-dose at the Day 1, Day 7, Weeks 2, 4, 6, 8, 10, 12 and 14 visits. As treatment may range 10 – 14 weeks, the Week 12 and 14 visit samples may not be collected in all subjects.

Intensive PK sampling will be performed in a subset of subjects (~100 subjects, including ~67 subjects on MK-8228 and ~33 subjects on placebo). The 5 samples will be collected at the Day 7 visit (i.e., on Days 5-9 after starting study therapy) at the following time points: pre-dose, 1 hour (± 10 min) following oral administration (or within 10 min **after** infusion completion, when given IV), 2.5 hours following oral/IV administration (± 30 min), 8 hours following oral/IV administration (range of 6-10 hours), and 24 hours following oral/IV administration (range of 22-24 hours; 0-2 hours prior to next day's dose).

7.1.3.3 CMV DNA PCR Testing

Sample collection, storage, and shipment instructions for plasma samples will be provided in the operations/laboratory manual.

CMV DNA PCR testing will be performed using the Roche COBAS® AmpliPrep/COBAS TaqMan® (CAP/CTM) assay at the central laboratory. After the initial screening visit until randomization, samples must be collected **once a week** and sent to the **central laboratory**. Thereafter, samples must be collected **at every visit** and sent to the **central laboratory**, as indicated in the Trial Flow Chart (Section 6.0). CMV DNA PCR testing may be conducted within 3 days prior to a scheduled study visit and no later than the day of the scheduled study visit. (This CMV DNA PCR testing window is not applicable to a CMV Infection Visit. See note below.)

Note: It is mandatory to send a confirmatory plasma sample for CMV DNA PCR testing to the central laboratory **immediately prior to** (i.e., on the day of) initiating treatment for CMV disease or PET. In the event that the confirmatory result obtained on the day of PET initiation is **NOT** available (e.g., sample is lost or mishandled by the investigator site prior to shipment, or is inadequate upon receipt at the central laboratory), a subsequent sample must be obtained and sent to the central laboratory within 7 days after PET initiation (preferably within 48-72 hours). The subject will be considered to have met the primary efficacy endpoint if this confirmatory laboratory result is positive.

In the event test results from the central laboratory are not available within the timeframe the investigator wishes to initiate anti-CMV therapy, the investigator may use a positive local laboratory test result in order to make the decision. However, as described above, plasma samples for CMV DNA PCR testing must also be sent to the central laboratory. The local laboratory result must also be reported in such instances.

7.1.3.4 CMV DNA Sequence Analysis

Sample collection, storage, and shipment instructions for plasma samples will be provided in the central laboratory manual.

CMV DNA Sequence Analysis will be performed only on subjects enrolled in the MK-8228 arm of the study who meet the criteria for clinically significant CMV infection. Resistance to MK-8228 will be monitored by genotypic analysis of the CMV terminase complex gene UL56 (and under certain conditions, the UL89 gene) in DNA extracted from plasma samples collected as indicated in the Trial Flow Chart (Section 6.0). Samples will be analyzed by standard population sequencing technology through an established contract laboratory with validated protocols in place. In subjects with multiple CMV-positive samples, the last on-therapy and follow-up samples will be used for analysis.

CMV DNA sequencing will focus on the UL56 gene. The genotyping assay for this study has a target validation cutoff of 1000 copies/mL; therefore, CMV DNA sequencing will only be conducted on samples with CMV DNA levels ≥ 1000 copies/mL. Based on in vitro selection and characterization of mutant viruses that escape inhibition by MK-8228, several substitutions in the UL56 protein have been confirmed as necessary and sufficient for resistance to MK-8228. No MK-8228 resistance-associated mutations have been identified in other CMV genes, including those encoding other presumed terminase subunits (such as UL89). However, previous studies with structurally-unrelated CMV terminase inhibitors have identified mutations in the UL89 gene that confer resistance to those compounds. Therefore, the CMV DNA Sequence Analysis will be staged as follows:

1. Sequencing of the complete UL56 gene will be attempted for subjects enrolled in the MK-8228 arm of the study who meet the criteria for clinically significant CMV infection and whose CMV DNA levels are ≥ 1000 copies/mL.

2. UL89 sequencing will be attempted when the following conditions are met:

    - An alignment of the sample UL56 DNA sequence either exactly matches the reference UL56 DNA sequence, or the only mismatches are at sites of UL56 DNA sequence polymorphisms known not to confer MK-8228 resistance;

      - and -

    - Sufficient plasma sample is available to allow additional genotyping.

Phenotypic analysis will be performed on any UL56 or UL89 DNA sequences which encode amino acid substitutions that: a) have not been previously characterized via phenotypic analysis; or b) are at sites of DNA sequence polymorphisms known not to confer MK-8228 resistance. For each mutant virus that meets these criteria, the MK-8228 susceptibility will be measured and compared to that of the reference UL56/UL89 wild-type virus.

7.1.3.5 Future Biomedical Research

The following specimens are to be obtained as part of Future Biomedical Research:

- Leftover DNA for future research
- Leftover main study plasma collected for CMV DNA PCR for future research

7.1.4 Other Procedures

7.1.4.1 Withdrawal/Discontinuation

When a subject discontinues/withdraws prior to trial completion, all applicable activities scheduled for the Early Discontinuation Visit (if subject discontinues/withdraws from the trial up to Week 24 post-transplant) or Week 48 post-transplant (if subject discontinues/withdraws from the trial after Week 24 post-transplant) should be performed at the time of discontinuation. Any adverse events which are present at the time of discontinuation/withdrawal should be followed in accordance with the safety requirements outlined in Section 7.2 - Assessing and Recording Adverse Events. **Note:** When a subject is discontinued from *study therapy* due to clinically significant CMV infection, a CMV Infection Visit must be performed prior to initiation of treatment of CMV diseases or initiation of PET. These subjects will continue to be followed in the study and complete all remaining study visits through Week 48 post-transplant.

7.1.4.1.1 Withdrawal From Future Biomedical Research

Subjects may withdraw their consent for Future Biomedical Research and have their specimens and all derivatives destroyed. Subjects may withdraw consent at any time by writing to the principal investigator for the main trial. If medical records for the main trial are still available, the investigator will contact the Sponsor using the designated mailbox PPD [redacted] and a form will be provided by the Sponsor to obtain appropriate information to complete specimen withdrawal. Subsequently, the subject's specimens will be removed from the biorepository and be destroyed. A letter will be sent from the Sponsor to the investigator confirming the destruction. It is the responsibility of the investigator to inform the subject of completion of destruction. Any analyses in progress at the time of request for destruction or already performed prior to the request being received by the Sponsor will continue to be used as part of the overall research trial data and results. No new analyses would be generated after the request is received.

In the event that the medical records for the main trial are no longer available (e.g., if the investigator is no longer required by regulatory authorities to retain the main trial records) or the specimens have been completely anonymized, there will no longer be a link between the subject's personal information and their specimens. In this situation, the request for specimen destruction cannot be processed.

7.1.4.2 Blinding/Unblinding

IVRS/IWRS should be used for emergency unblinding treatment assignment in the event that this is required for subject safety.

In the event that unblinding has occurred, the circumstances around the unblinding (e.g., date and reason) must be documented promptly, and the Sponsor Clinical Monitor notified as soon as possible. Only the principal investigator or delegate and the respective subject's code should be unblinded. Trial site personnel and Sponsor personnel directly associated with the conduct of the trial should not be unblinded.

7.1.5 Visit Requirements

Visit requirements are outlined in Section 6.0 - Trial Flow Chart. Specific procedure-related details are provided above in Section 7.1 - Trial Procedures.

7.1.5.1 Screening

Subjects may be screened during a period starting from 15 days prior to transplantation through 28 days post-transplant (laboratory test results necessary for randomization must be available within 5 days prior to the planned day of randomization). The informed consent must be obtained before any study-specific procedure is performed. It is acceptable that the date of obtaining informed consent is earlier than the day of performing screening procedures. However, once informed consent is obtained adverse event reporting must be conducted according to Section 7.1.2.7.

Potential subjects will be evaluated to determine if they fulfill the Inclusion/Exclusion entry requirements as described in Section 5.1 (Entry Criteria). The investigator will discuss with each potential subject the nature of the study and its requirements/restrictions. All screening procedures listed under Visit 1 of the Trial Flow Chart (Section 6.0) will be performed. Subjects will be instructed that they are required to use two acceptable methods of birth control starting from the time of consent through 90 days after the last dose of study therapy (or longer if dictated by local regulations). Subjects will also be instructed about the restrictions for concomitant medications, as noted in Section 5.5.

For screening purposes, values from the subject's chart for required chemistry, hematology, and urinalysis tests are acceptable. If not available, this testing may be performed by the central laboratory. HIV antibody test results documented at any time prior to randomization of the subject will be acceptable. A copy of report must be available. If documentation of a previous HIV test is not available, the HIV antibody test must be conducted using the central laboratory. Hepatitis B, and hepatitis C screening should only be performed if not previously documented within the last 90 days. If hepatitis C virus antibody is positive, RNA PCR results should be provided (or, if not available, RNA PCR testing will be performed by the central laboratory).

CMV procedures/assessments will also be performed at screening. For initial screening, results of CMV DNA PCR assay performed at a local laboratory will be acceptable to establish absence of CMV viremia. Thereafter, CMV DNA PCR testing will be performed once a week by the central laboratory, using the Roche COBAS® AmpliPrep/COBAS TaqMan® (CAP/CTM) assay, in order to exclude those with active CMV replication prior to study therapy initiation.

On the day of randomization, eligibility for enrollment into the study should be confirmed. At that time, subjects have already received their HSCT and will be considered eligible for randomization once (a) they are determined to be negative for CMV viremia (**NO** evidence of CMV viremia from a central or local laboratory at any time point **and** confirmed by the central laboratory on a sample collected from the subject within 5 days prior to randomization), and (b) have acceptable creatinine clearance and liver function test values (i.e., within the range allowable in this study, as outlined in Section 5.1.3 [Subject Exclusion Criteria]) from testing performed within 5 days prior to randomization. **(NOTE: Evidence of CMV viremia as reported by the central lab will include reporting of test results as "detectable, not quantifiable" or "detected" with a numeric value provided.)**

Presence of CMV disease in the screening period will be assessed according to Appendix 12.4.

7.1.5.2 Study Therapy Period

Study therapy (with MK-8228 or placebo) may begin as early as the day of transplant and no later than 28 days post-transplant. Study therapy will continue through Week 14 (~100 days) post-transplant. Study therapy visits will occur weekly through Week 14 (~100 days) post-transplant.

The Day 1 Visit (as shown in the Trial Flow Chart, Section 6.0) will be day the subject is randomized and study therapy is initiated. Study therapy will continue through the End of Study Therapy Visit. The End of Study Therapy Visit may occur at the Week 10, 11, 12, 13, or 14 Visit depending on when study therapy is started during the 28-day post-transplant window. For example, if study therapy is started on the day of transplant, the End of Study Therapy Visit will be the Week 14 Visit (which corresponds to Week 14 post-transplant). If study therapy is started 28 days post-transplant, the End of Study Therapy Visit will be the Week 10 Visit (which corresponds to Week 14 post-transplant).

All procedures listed under the weekly study therapy visits in the Trial Flow Chart (Section 6.0) will be performed at the corresponding visit. After randomization, the physical examination does not need to be performed at every visit; a targeted physical exam should be performed only if a subject has any complaints. After randomization, vital signs should only be performed if targeted physical examination is performed.

7.1.5.2.1 Day 1 Visit

Day 1 procedures/assessments listed on the Trial Flow Chart must be performed prior to initiation of study therapy. Assessment of quality of life (using FACT-BMT and EQ-5D questionnaires) should be completed prior to any study procedures at this visit.

Laboratory safety evaluations (hematology, chemistry, and urinalysis) specified in Section 7.1.3.1 will be performed prior to study therapy initiation. These samples will be sent to the appropriate central laboratory(ies) following the procedure(s) described in the manual(s).

For female subjects, a urine pregnancy test will be performed at the site prior to study therapy initiation. If the urine pregnancy test result is negative, the subject will be eligible for randomization and the remainder of the Day 1 testing/procedures will be performed. If the urine pregnancy result is positive, the subject must not be randomized.

For male subjects, serum inhibin B, luteinizing hormone (LH), follicle stimulating hormone (FSH), and testosterone testing will be performed.

7.1.5.2.2 Study Therapy Administration

Following completion of the Day 1 procedures/assessments and confirmation of eligibility (including availability of results from samples for CMV PCR, creatinine clearance, and liver function tests), the subject will be randomized. The site pharmacist or study coordinator will contact the IVRS for assignment of the study therapy to be administered. Sites should not call the IVRS for study therapy administration until the subject has met all criteria for the study and is ready to receive the first dose of study therapy on Day 1.

The first dose of study therapy will be administered at the Day 1 Visit. The oral or IV formulation of MK-8228 or placebo will be dispensed via the IVRS. Subjects will be initiated with the oral (tablet) formulation of study therapy provided they are able to swallow and do not have a condition (e.g., vomiting, diarrhea, or a malabsorptive condition) that may interfere with the absorption of the tablets. For subjects who cannot swallow and/or have a condition that may interfere with the absorption of the oral formulation, study therapy can be initiated with or switched to the IV formulation. The IV formulation should be switched to oral study therapy (i.e., at the next planned dose) as soon as such subjects are able to swallow and/or the condition necessitating the use of the IV formulation resolves. Use of the IV formulation should generally be limited to 4 weeks or less in duration. However, it will be left to the investigator’s discretion to continue IV administration beyond 4 weeks, if the benefit/risk ratio supports continued administration.

The same dose of MK-8228 will be administered for both formulations. Subjects in the MK-8228 treatment group will receive either 240 mg MK-8228 qd, if receiving concomitant CsA, or 480 mg MK-8228 qd, if not on CsA. If CsA is initiated after starting study therapy, the next dose of MK-8228 (administered up to 24 hours later) should be decreased to 240 mg qd. If CsA is discontinued permanently or for the long-term in a subject, the next dose of MK-8228 (administered up to 24 hours later) should be increased from 240 mg to 480 mg qd. If CsA is temporarily held due to high levels detected by therapeutic blood monitoring, the dose of MK-8228 need not be adjusted. Corresponding changes in tablets for oral formulation with changes in CsA dosing will also occur in the placebo group in an effort to maintain the study blind (see Section 5.2.3).

With this protocol amendment, both 240 mg and 480 mg oral (tablet) formulations of MK-8228 (and matching placebo) are available for study therapy. Subjects not on CsA requiring the oral 480 mg dose should be initiated with one 480 mg tablet (MK-8228 or matching placebo). In the event a subject is unable to swallow the 480 mg tablet, study therapy may be initiated with 2 x 240 mg tablets (MK-8228 or matching placebo). Subjects not on CsA who were initiated with two tablets of the 240 mg MK-8228 or matching placebo should continue with that regimen. After Day 1, study therapy will continue through Week 14 (~100 days) post-transplant. During this period, samples for CMV DNA PCR should be sent **at every visit** to the **central laboratory** as per the Trial Flow Chart (Section 6.0). The CMV DNA PCR test may be conducted within 3 days prior to a scheduled study visit and no later than the day of the scheduled study visit. (This CMV DNA PCR testing window is not applicable to a CMV Infection Visit. See Section 7.1.3.3)

The subject will be trained in the use of the electronic Study Medication Diary. Once the subject is discharged from the hospital, he/she will be instructed to enter the number of tablets of study therapy taken during the study therapy period.

7.1.5.3 Follow-up Period

After the last day of study therapy, subjects will continue to be followed though Week 24 (~6 months) post-transplant. Visits will occur every 2 weeks between Week 14 post-transplant and Week 24 post-transplant, and all procedures listed in the Trial Flow Chart (Section 6.0) corresponding to the visits will be performed.

Following the primary study period through Week 24 (~6 months) post-transplant, subjects will remain in the study through Week 48 post-transplant in order to continue collecting information on CMV disease, health outcomes, and quality of life. Visits will occur at Weeks 32, 40, and 48 post-transplant and all procedures listed in the Trial Flow Chart (Section 6.0) corresponding to the visits will be performed.

During the follow-up period, samples for CMV DNA PCR should be sent **at every visit** to the **central laboratory** as per the Trial Flow Chart (Section 6.0). The CMV DNA PCR test may be conducted within 3 days prior to a scheduled study visit and no later than the day of the scheduled study visit. (This CMV DNA PCR testing window is not applicable to a CMV Infection Visit. See Section 7.1.3.3.)

Adverse event monitoring should include the collection of all adverse events while on study therapy and for 2 weeks following completion of study therapy (i.e., through Follow-up Week 2 Visit) in all subjects, including those who have discontinued study therapy but are continuing in the study. Thereafter, only drug related SAEs and SAEs leading to death will be collected through Week 48 post-transplant (i.e., through Follow-up Week 34 Visit).

7.1.5.4 CMV Infection or Early Discontinuation Visit

The CMV Infection Visit will be performed for all subjects who will be discontinued from study therapy due to clinically significant CMV infection requiring either treatment of disease or initiation of PET. It is very important to ensure that all procedures, as outlined in the Trial Flow Chart (Section 6.0), are performed at the CMV Infection Visit **immediately prior to** the initiation of treatment of CMV diseases or initiation of PET (i.e., on the day anti-CMV therapy is initiated). Most importantly, a confirmatory plasma sample for CMV PCR testing at the central laboratory should be collected at this visit.

After this visit, such subjects will continue to be followed in the study and complete all remaining visits (including all subsequent treatment period visits) through Week 48 post-transplant as outlined in the Trial Flow Chart (Section 6.0). All specified procedures during the study therapy period will be completed for these subjects with the exception of study therapy administration, PK assessments, and study medication diary review.

The CMV Infection Visit will also be performed for all subjects who require either treatment for disease or initiation of PET after study therapy completion, during the follow-up period (after Week 14 through Week 24 post-transplant). It is very important to ensure that all procedures, as outlined in the Trial Flow Chart (Section 6.0), are performed at the CMV Infection Visit **immediately prior to** the initiation of treatment of CMV diseases or initiation of PET (i.e., on the day anti-CMV therapy is initiated). Most importantly, a plasma sample for CMV PCR testing at the central laboratory should be collected at this visit.

After this visit, such subjects will continue to be followed in the study and complete all remaining visits through Week 48 post-transplant as outlined in the Trial Flow Chart (Section 6.0).

Note: It is mandatory to send a confirmatory plasma sample for CMV DNA PCR testing to the central laboratory **immediately prior to** (i.e., on the day of) initiating treatment for CMV disease or PET in **ALL** instances. In the event that the confirmatory result obtained on the day of PET initiation is **NOT** available (e.g., sample is lost or mishandled by the investigator site prior to shipment, or is inadequate upon receipt at the central laboratory), a subsequent sample must be obtained and sent to the central laboratory within 7 days after PET initiation (preferably within 48-72 hours). The subject will be considered to have met the primary efficacy endpoint if this confirmatory laboratory result is positive.

In the event confirmatory test results from the central laboratory are not available within the timeframe the investigator wishes to initiate anti-CMV therapy, the investigator may use a positive local laboratory test result (from CMV DNA PCR or pp65 antigen only) to make the decision. However, as described above, plasma samples for CMV DNA PCR testing must also be sent to the central laboratory. The local laboratory result must also be reported in such instances.

Note on reinitiation of study therapy: There may be instances where confirmatory central lab test results for CMV DNA PCR obtained on the day of PET initiation may be negative (CMV DNA undetectable) and the investigator may wish to discontinue PET. The decision to stop PET in the event of a negative (CMV not detectable) confirmatory central laboratory result collected on the day of PET initiation resides with the investigator caring for the subject. Therefore, in the event the confirmatory CMV DNA sample at PET initiation is negative for CMV viremia, the Sponsor will allow for protocol-defined study therapy (i.e., letermovir or placebo, based on initial randomization) to be restarted at the investigator's discretion, once PET is discontinued. In such instances, study therapy should be restarted within 7 days from the date on which study therapy was stopped. It is important to note that the status of the subject's study therapy in IVRS should NOT be changed until the CMV DNA PCR result is confirmed and the investigator is certain that study therapy will be permanently discontinued.

The Early Discontinuation Visit will be performed for all subjects who are prematurely discontinued up to Week 24 post-transplant from the *study, not study therapy*. It is very important to ensure that all procedures, as outlined in the Trial Flow Chart (Section 6.0), are performed in such subjects at this visit prior to discontinuing the subject from the trial. Most importantly, a plasma sample for CMV PCR testing at the central laboratory should be collected at this visit.

7.2 Assessing and Recording Adverse Events

An adverse event is defined as any untoward medical occurrence in a patient or clinical investigation subject administered a pharmaceutical product and which does not necessarily have to have a causal relationship with this treatment. An adverse event can therefore be any unfavourable and unintended sign (including an abnormal laboratory finding, for example), symptom, or disease temporally associated with the use of a medicinal product or protocol-specified procedure, whether or not considered related to the medicinal product or protocol-specified procedure. Any worsening (i.e., any clinically significant adverse change in frequency and/or intensity) of a preexisting condition that is temporally associated with the use of the Sponsor's product, is also an adverse event.

Changes resulting from normal growth and development that do not vary significantly in frequency or severity from expected levels are not to be considered adverse events. Examples of this may include, but are not limited to, teething, typical crying in infants and children and onset of menses or menopause occurring at a physiologically appropriate time.

Sponsor's product includes any pharmaceutical product, biological product, device, diagnostic agent or protocol-specified procedure, whether investigational (including placebo

or active comparator medication) or marketed, manufactured by, licensed by, provided by or distributed by the Sponsor for human use.

Adverse events may occur during the course of the use of the Sponsor's product in clinical trials or within the follow-up period specified by the protocol, or prescribed in clinical practice, from overdose (whether accidental or intentional), from abuse and from withdrawal.

Adverse events may also occur in screened subjects during any pre-allocation baseline period as a result of a protocol-specified intervention, including washout or discontinuation of usual therapy, diet, placebo treatment or a procedure.

From the time informed consent is signed until randomization, the following adverse events only should be reported: those resulting from protocol-specified procedures or intervention, those resulting in death and those resulting in a patient not being randomized. After Randomization and initiation of study therapy, adverse event monitoring will include the collection of all adverse events through the Follow-up Week 2 Visit (16 weeks post-transplant) in all subjects, including those who have discontinued study therapy but continue to be followed-up in the study. Thereafter, only drug-related serious adverse events (SAEs) and SAEs leading to death will be collected through Week 48 post-transplant. All adverse events will be recorded on the Adverse Event case report forms/worksheets accordingly.

7.2.1 Definition of an Overdose for This Protocol and Reporting of Overdose to the Sponsor

In this trial, an overdose is any dose higher than two times the dose specified in Section 5.2 (Trial Treatments).

If an adverse event(s) is associated with ("results from") the overdose of Sponsor's product or vaccine, the adverse event(s) is reported as a serious adverse event, even if no other seriousness criteria are met.

If a dose of Sponsor's product or vaccine meeting the protocol definition of overdose is taken without any associated clinical symptoms or abnormal laboratory results, the overdose is reported as a non-serious Event of Clinical Interest (ECI), using the terminology "accidental or intentional overdose without adverse effect."

All reports of overdose with and without an adverse event must be reported within 24 hours to the Sponsor either by electronic media or paper. Sponsor Contact information can be found in the Investigator Trial File Binder.

7.2.2 Reporting of Pregnancy and Lactation to the Sponsor

Although pregnancy and lactation are not considered adverse events, it is the responsibility of investigators or their designees to report any pregnancy or lactation in a subject (spontaneously reported to them) that occurs during the trial or within 14 days of completing the trial. All subjects who become pregnant must be followed to the completion/termination of the pregnancy. Pregnancy outcomes of spontaneous abortion, missed abortion, benign hydatidiform mole, blighted ovum, fetal death, intrauterine death, miscarriage and stillbirth must be reported as serious events (Important Medical Events). If the pregnancy continues to term, the outcome (health of infant) must also be reported.

Such events must be reported within 24 hours to the Sponsor either by electronic media or paper. Sponsor Contact information can be found in the Investigator Trial File Binder.

7.2.3 Immediate Reporting of Adverse Events to the Sponsor

7.2.3.1 Serious Adverse Events

A serious adverse event is any adverse event occurring at any dose or during any use of Sponsor's product that:

- Results in death;
- Is life threatening;
- Results in persistent or significant disability/incapacity;
- Results in or prolongs an existing inpatient hospitalization;
- Is a congenital anomaly/birth defect;
- Is a cancer;
- Is associated with an overdose;
- Is an other important medical event

Refer to Table 5 for additional details regarding each of the above criteria.

Any serious adverse event, or follow up to a serious adverse event, including death due to any cause that occurs to any subject from the time the consent is signed through 2 weeks following the end of the treatment period (Week 16, Follow-up Week 2) for all subjects including those who have discontinued study therapy, whether or not related to the Sponsor's product, must be reported within 24 hours to the Sponsor either by electronic media or paper. Sponsor Contact information can be found in the Investigator Trial File Binder.

Additionally, any serious adverse event, considered by an investigator who is a qualified physician to be related to the Sponsor's product or death due to any cause that is brought to the attention of the investigator at any time outside of the time period specified in the previous paragraph also must be reported immediately to the Sponsor.

All subjects with serious adverse events must be followed up for outcome.

7.2.3.2 Events of Clinical Interest

Selected non-serious and serious adverse events are also known as Events of Clinical Interest (ECI) and must be recorded as such on the Adverse Event case report forms/worksheets and reported within 24 hours to the Sponsor either by electronic media or paper. Sponsor Contact information can be found in the Investigator Trial File Binder.

Events of clinical interest for this trial include:

3. an overdose of Sponsor's product, as defined in Section 7.2.1 - Definition of an Overdose for This Protocol and Reporting of Overdose to the Sponsor, that is not associated with clinical symptoms or abnormal laboratory results.

4. an elevated AST or ALT lab value that is greater than or equal to 3X the upper limit of normal and an elevated total bilirubin lab value that is greater than or equal to 2X the upper limit of normal and, at the same time, an alkaline phosphatase lab value that is less than 2X the upper limit of normal, as determined by way of protocol-specified laboratory testing or unscheduled laboratory testing.*

   *Note: These criteria are based upon available regulatory guidance documents. The purpose of the criteria is to specify a threshold of abnormal hepatic tests that may require an additional evaluation for an underlying etiology. The trial site guidance for assessment and follow up of these criteria can be found in the Investigator Trial File Binder.

7.2.3.3 Protocol-Specific Exceptions to Serious Adverse Event Reporting

7.2.4 Evaluating Adverse Events

An investigator who is a qualified physician will evaluate all adverse events with respect to the elements outlined in Table 5 The investigator’s assessment of causality is required for each adverse event. Refer to Table 5 for instructions in evaluating adverse events.

Table 5 Evaluating Adverse Events

**Maximum Intensity**	**Mild**	awareness of sign or symptom, but easily tolerated (for pediatric trials, awareness of symptom, but easily tolerated)
	**Moderate**	discomfort enough to cause interference with usual activity (for pediatric trials, definitely acting like something is wrong)
	**Severe**	incapacitating with inability to work or do usual activity (for pediatric trials, extremely distressed or unable to do usual activities)
**Seriousness**	A serious adverse event (AE) is any adverse event occurring at any dose or during any use of Sponsor's product that:	
	†**Results in death**; or	
	†**Is life threatening; or** places the subject, in the view of the investigator, at immediate risk of death from the event as it occurred [Note: This does not include an adverse event that, had it occurred in a more severe form, might have caused death.]; or	
	†**Results in a persistent or significant disability/incapacity** (substantial disruption of one’s ability to conduct normal life functions); or	
	†**Results in or prolongs an existing inpatient hospitalization** (hospitalization is defined as an inpatient admission, regardless of length of stay, even if the hospitalization is a precautionary measure for continued observation. (Note: Hospitalization [including hospitalization for an elective procedure] for a preexisting condition which has not worsened does not constitute a serious adverse event.); or	
	†**Is a congenital anomaly/birth defect (**in offspring of subject taking the product regardless of time to diagnosis); or	
	**Is a cancer; or**	
	**Is associated with an overdose** (whether accidental or intentional). Any adverse event associated with an overdose is considered a serious adverse event. An overdose that is not associated with an adverse event is considered a non-serious event of clinical interest and must be reported within 24 hours.	
	**Other important medical events** that may not result in death, not be life threatening, or not require hospitalization may be considered a serious adverse event when, based upon appropriate medical judgment, the event may jeopardize the subject and may require medical or surgical intervention to prevent one of the outcomes listed previously (designated above by a †).	
**Duration**	Record the start and stop dates of the adverse event. If less than 1 day, indicate the appropriate length of time and units	
**Action taken**	Did the adverse event cause the Sponsor's product to be discontinued?	
**Relationship to Sponsor's Product**	Did the Sponsor's product cause the adverse event? The determination of the likelihood that the Sponsor's product caused the adverse event will be provided by an investigator who is a qualified physician. The investigator’s signed/dated initials on the source document or worksheet that supports the causality noted on the AE form, ensures that a medically qualified assessment of causality was done. This initialed document must be retained for the required regulatory time frame. The criteria below are intended as reference guidelines to assist the investigator in assessing the likelihood of a relationship between the test drug and the adverse event based upon the available information.<br>**The following components are to be used to assess the relationship between the Sponsor's product and the AE**; the greater the correlation with the components and their respective elements (in number and/or intensity), the more likely the Sponsor's product caused the adverse event:	
	**Exposure**	Is there evidence that the subject was actually exposed to the Sponsor's product such as: reliable history, acceptable compliance assessment (pill count, diary, etc.), expected pharmacologic effect, or measurement of drug/metabolite in bodily specimen?
	**Time Course**	Did the AE follow in a reasonable temporal sequence from administration of the Sponsor's product?<br>Is the time of onset of the AE compatible with a drug-induced effect (applies to trials with investigational medicinal product)?
	**Likely Cause**	Is the AE not reasonably explained by another etiology such as underlying disease, other drug(s)/vaccine(s), or other host or environmental factors

**Relationship to Sponsor's Product (continued)**	**The following components are to be used to assess the relationship between the Sponsor's product and the AE: (continued)**	
	**Dechallenge**	Was the Sponsor's product discontinued or dose/exposure/frequency reduced?<br>If yes, did the AE resolve or improve?<br>If yes, this is a positive dechallenge. If no, this is a negative dechallenge.<br>(Note: This criterion is not applicable if: (1) the AE resulted in death or permanent disability; (2) the AE resolved/improved despite continuation of the Sponsor's product; (3) the trial is a single-dose drug trial); or (4) Sponsor's product(s) is/are only used one time.)
	**Rechallenge**	Was the subject re-exposed to the Sponsor's product in this trial?<br>If yes, did the AE recur or worsen?<br>If yes, this is a positive rechallenge. If no, this is a negative rechallenge.<br>(Note: This criterion is not applicable if: (1) the initial AE resulted in death or permanent disability, or (2) the trial is a single-dose drug trial); or (3) Sponsor's product(s) is/are used only one time.)<br>NOTE: IF A RECHALLENGE IS PLANNED FOR AN ADVERSE EVENT WHICH WAS SERIOUS AND WHICH MAY HAVE BEEN CAUSED BY THE SPONSOR'S PRODUCT, OR IF RE-EXPOSURE TO THE SPONSOR'S PRODUCT POSES ADDITIONAL POTENTIAL SIGNIFICANT RISK TO THE SUBJECT THEN THE RECHALLENGE MUST BE APPROVED IN ADVANCE BY THE U.S. CLINICAL MONITOR AND THE INSTITUTIONAL REVIEW BOARD/INDEPENDENT ETHICS COMMITTEE.
	**Consistency with Trial Treatment Profile**	Is the clinical/pathological presentation of the AE consistent with previous knowledge regarding the Sponsor's product or drug class pharmacology or toxicology?
The assessment of relationship will be reported on the case report forms /worksheets by an investigator who is a qualified physician according to his/her best clinical judgment, including consideration of the above elements.		
**Record one of the following**:		**Use the following scale of criteria as guidance (not all criteria must be present to be indicative of a Sponsor's product relationship).**
**Yes, there is a reasonable possibility of Sponsor's product relationship.**		There is evidence of exposure to the Sponsor's product. The temporal sequence of the AE onset relative to the administration of the Sponsor's product is reasonable. The AE is more likely explained by the Sponsor's product than by another cause.
**No, there is not a reasonable possibility of Sponsor's product relationship**		Subject did not receive the Sponsor's product OR temporal sequence of the AE onset relative to administration of the Sponsor's product is not reasonable OR there is another obvious cause of the AE. (Also entered for a subject with overdose without an associated AE.)

7.2.5 Sponsor Responsibility for Reporting Adverse Events

All Adverse Events will be reported to regulatory authorities, IRB/IECs and investigators in accordance with all applicable global laws and regulations.

7.3 TRIAL GOVERNANCE AND OVERSIGHT

7.3.1 Scientific Advisory Committee

This trial was developed in collaboration with a Scientific Advisory Committee (SAC). The SAC comprises both Sponsor and non-Sponsor scientific experts who provide input with respect to trial design, interpretation of trial results and subsequent peer-reviewed scientific publications.

7.3.2 Executive Oversight Committee

The Executive Oversight Committee (EOC) comprises members of Sponsor Senior Management. The EOC will receive and decide upon any recommendations made by the Data Monitoring Committee (DMC) regarding the trial.

7.3.3 Data Monitoring Committee

To supplement the routine trial monitoring outlined in this protocol, an external Data Monitoring Committee (DMC) will monitor the interim data from this trial. The voting members of the committee are external to the Sponsor. The members of the DMC must not be involved with the trial in any other way (e.g., they cannot be trial investigators) and must have no competing interests that could affect their roles with respect to the trial. The DMC will include 4 clinicians experienced in Infectious Diseases and 1 external statistician; this is in addition to the unblinded trial statistician who will be a non-voting member of the committee.

The DMC will make recommendations to the EOC regarding steps to ensure both subject safety and the continued ethical integrity of the trial. Also, the DMC will review interim trial results, consider the overall risk and benefit to trial participants (see Section 8.1.4 - Interim Analyses) and recommend to the EOC if the trial should continue in accordance with the protocol.

Specific details regarding responsibilities and governance, including the roles and responsibilities of the various members and the Sponsor protocol team; meeting facilitation; the trial governance structure; and requirements for proper documentation of DMC reports, minutes, and recommendations will be described in a separate charter that is reviewed and approved by the DMC. The DMC will monitor the trial at an appropriate frequency, as described in the detailed DMC charter. The DMC will also make recommendations to the Sponsor protocol team regarding steps to ensure both subject safety and the continued ethical integrity of the trial.

7.3.4 Clinical Adjudication Committee

A Clinical Adjudication Committee (CAC) will evaluate the following events for the purposes of confirming them according to the criteria in Section 8.0 – Statistical Analysis Plan, as well as evaluating the presence of confounding factors.

1) CMV disease, as defined in Appendix 12.4.: This role is important to standardize the evaluation of all suspected cases of CMV disease occurring during the trial.

All personnel involved in the adjudication process will remain blinded to treatment allocation throughout the trial. Specific details regarding endpoint definitions can be found in the Adjudication Charter.

8.0 STATISTICAL ANALYSIS PLAN

8.1 Statistical Analysis Plan Summary

This section contains a brief summary of the statistical analyses for this trial. Full detail is in the Statistical Analysis Plan (SAP) (Section 8.2).

8.1.1 Efficacy Analyses

The primary and key secondary endpoints, primary analysis population, and statistical methods that will be employed for the efficacy analyses are presented in Table 6 below.

The FAS population consists of all randomized subjects who have received at least one dose of study medication and had no detectable CMV viral DNA (measured by central laboratory) on Day 1 (when study therapy is initiated).

The primary hypothesis will be evaluated by comparing MK-8228 to placebo in the proportion of subjects with clinically significant CMV infection (as defined in Sections 2.1 and 4.2.3.1) through Week 24 post-transplant in the FAS population. Other efficacy analyses will be considered supportive and/or explanatory.

Table 6 Summary of Analysis Strategy for Key Efficacy Endpoints

Endpoint/Variable (Description, Timepoint)	Statistical Method	Analysis Population	Missing Data Approach
Primary:			
Proportion of subjects with clinically significant CMV infection through Week 24 (~6 months) post-transplant	Stratified Mantel-Haenszel	Full Analysis set	Non-Completer = Failure*
Key Secondary:			
Proportion of subjects with clinically significant CMV infection through Week 14 (~100 days) post-transplant	Stratified Mantel-Haenszel	Full Analysis set	Non-Completer = Failure*
Time to onset of clinically significant CMV infection through Week 24 (~6 months) post-transplant	Kaplan-Meier plot	Full Analysis set	Censored at last assessment
* Non-completers refer to subjects who prematurely discontinued from the study (See Section 8.2.5.1.4 for details).			

8.1.2 Safety Analyses

All AEs will be collected through 14 days after completion of treatment period (through Follow-up Week 2 Visit). Thereafter, any SAEs related to study medication or SAEs leading to death will be collected through Week 48 post-transplant. Safety and tolerability will be assessed by statistical and clinical review of all safety data collected throughout the study. The All-Subjects-as-Treated population will be employed for safety analyses.

Statistical analyses of adverse events will follow the 3-tiered analysis approach. For this study, there is no pre-specified Tier 1 event that will be formally compared using inferential statistics. Tier 2 events include AEs that occur in ≥ 4 subjects in any one treatment group and also include (1) at least one adverse event; (2) a drug-related adverse event; (3) a serious adverse event; (4) a serious and drug-related adverse event and (5) an adverse event leading to discontinuation. Tier 2 events will be assessed via point estimates with 95% confidence intervals provided for between-group comparisons. Descriptive safety endpoints (Tier 3) include all other safety parameters not analyzed as a Tier 2 safety endpoint. These Tier 3 safety endpoints include AEs, laboratory assessments, vital signs, and ECGs. Only point estimates by treatment group are provided for Tier 3 safety parameters.

8.1.3 Power and Sample Size

Based on literature data for placebo and on the results from the Phase II study AIC246-01-II-02, the incidence rate of clinically significant CMV infection for subjects receiving placebo is expected to be approximately 35%. It is expected that the MK-8228 arm will reduce this incidence by half to an incidence of approximately 17%. It is further anticipated that about 20% of subjects will be discontinued from the trial for both treatment arms for reasons other than virologic failure. Since the primary missing data approach will be Non-Completer = Failure approach, 20% was added to the expected incidence of clinically significant CMV infection for the placebo arm (55%) and the MK-8228 arm (37%) for sample size and power calculations.

A sample size of approximately 540 subjects is planned using a 2:1 randomization ratio (~360 subjects in the MK-8228 arm and ~180 subjects in the placebo arm). Excluding 15% subjects with detectable CMV DNA on Day 1, the evaluable number of subjects in the FAS population is 459 in total (306 in the MK-8228 arm and 153 in the placebo arm). A futility interim analysis (IA) will be conducted when approximately 40% of the subjects have completed treatment or discontinued prior to completing treatment. With the current sample size, this study will have 90.5% overall power. If the study were designed without the futility analysis, the power would be 95%.

Although there is no intention for stopping for overwhelming efficacy at the interim time, a small amount of alpha ($\alpha = 0.0001$) will be allocated for statistical rigor. At the end of the trial, a 1-sided p-value that is less than or equal to 0.0249 will be used for declaring efficacy success.

8.1.4 Interim Analysis

A futility analysis will be conducted when 40% randomized subjects either complete study therapy through Week 14 (~100 days) post-transplant or discontinue prior to that time point. Results will be reviewed by the DMC. The endpoint, timing, and purpose of the interim analyses are summarized in the table below. The decision rule and other statistical details are further described in Section 8.2.

In addition, periodic safety reviews will be conducted. The first safety review will occur when 10% of randomized subjects either complete study therapy through Week 14 (~100 days) post-transplant or discontinue prior to that time point. Thereafter, the DMC will review all safety data approximately every 6 months during the trial (or at longer time intervals in case of slow enrollment).

8.2 Statistical Analysis Plan

This section outlines the statistical analysis strategy and procedures for the study. If, after the study has begun, changes are made to primary and/or key secondary objectives, or the statistical methods related to those objectives, then the protocol will be amended (consistent with ICH Guideline E-9). Changes to exploratory or other non-confirmatory analyses made after the protocol has been finalized, along with an explanation as to when and why they occurred, will be listed in the Clinical Study Report (CSR) for the study. Post hoc exploratory analyses will be clearly identified in the CSR. No separate Statistical Analysis Plan (SAP) will be issued for this study.

8.2.1 Responsibility for Analyses/ In-House Blinding

The Clinical Biostatistics department or designee will generate the randomized allocation schedule(s) for study treatment assignment. Randomization will be implemented in an interactive voice/web response system (IVRS/IWRS). The statistical analysis of the data obtained from this study will be the responsibility of the Clinical Biostatistics department of the SPONSOR. Certain specific analyses such as those for pharmacokinetic (PK), pharmacogenetics, and quality of life measures will be the responsibility of the appropriate departments of the SPONSOR.

This study has a primary study period (from Day 1 through Week 24 post-transplant), followed by an extension period to Week 48 post-transplant in order to collect CMV disease, health outcomes and quality of life data. The primary study period will be conducted as a double-blind study under in-house blinding procedures. The official, final database for the primary study period will not be unblinded until medical/scientific review has been performed, protocol violators have been identified, and data have been declared final and complete. The CSR will be finalized after results from the primary study period (through Week 24 post-transplant) are complete; all available post Week 24 data pertaining to mortality and CMV disease will be provided. Results of the extension period (through Week 48 post-transplant) will be presented in a separate report.

The Clinical Biostatistics department or designee will generate the randomized allocation schedule(s) for study treatment assignment. Randomization will be implemented in an interactive voice/web response system (IVRS/IWRS).

Planned interim analyses are described in Section 8.1.4. Study enrollment is likely to be ongoing at the time of any interim analyses. Blinding to treatment assignment will be maintained at all investigational sites. The results of interim analyses will not be shared with the investigators prior to the completion of the study. Subject-level unblinding will be restricted to an external unblinded group of statisticians. Treatment-level results of the interim analysis will be provided by the external statisticians to the DMC. Limited additional SPONSOR personnel may be unblinded to the treatment level results of the interim analysis (analyses), if required, in order to act on the recommendations of the DMC. The extent to which individuals are unblinded with respect to results of interim analyses will be documented by the external statisticians.

Prior to final study unblinding, the external statisticians will not be involved in any discussions regarding modifications to the protocol, statistical methods, identification of protocol violators, or data validation efforts.

Pharmacokinetic measurements will be conducted in support of exposure-response evaluations. Additionally, a small team as specified in a separate Modeling and Simulation (M&S) Modeling Analysis Plan, and who are separate from the study team, will be unblinded for the purpose of preparing the pharmacokinetic analyses. No interim data or results will be shared with the study team, and the unblinded group will not be members of the study team. No alpha adjustment will be made for this administrative look.

8.2.2 Hypotheses/Estimation

Objectives and hypotheses of the study are stated in Section 3.0.

8.2.3 Analysis Endpoints

Efficacy and safety endpoints that will be evaluated are listed in the following sections.

8.2.3.1 Efficacy/Pharmacokinetic Endpoints

8.2.3.1.1 Efficacy Endpoints

An initial description of efficacy measures is provided in Section 4.2.3.1.

The primary efficacy endpoint will be the proportion of subjects with clinically significant CMV infection through Week 24 (~6 months) post-transplant, defined as the occurrence of either one of the following outcomes:

- onset of CMV end-organ disease

  OR

- initiation of anti-CMV PET based on documented CMV viremia (as measured by the central laboratory) and the clinical condition of the subject.

CMV end-organ disease will be determined using the definitions in Appendix 12.4 and confirmed by an independent, blinded Clinical Adjudication Committee (CAC). The adjudication of cases by the CAC (i.e., the final CAC assessment) will take precedence over the investigator's assessment. Only the CAC-confirmed cases of CMV end-organ disease will be included in the CMV end-organ disease category. However, investigator-assessed CMV end-organ disease cases which were not confirmed by the CAC but in whom anti-CMV therapy was initiated (in the setting of documented CMV viremia at a central laboratory) will be included in the initiation of PET category and, therefore, qualify as having clinically significant CMV infection. Concordance/discordance between CAC and investigator assessment will be summarized.

Documented viremia is defined as any detectable CMV viral DNA on a confirmatory sample obtained immediately prior to (i.e., on the day of) the initiation of treatment for CMV disease or PET, as measured by the Roche COBAS® AmpliPrep/COBAS TaqMan® (CAP/CTM) System in the central laboratory. In the event that the confirmatory result is not available, a subsequent central laboratory result collected from a sample obtained within 7 days will be used. Initiation of anti-CMV therapy without documented CMV viremia (using the central laboratory) will not be considered as a case for clinically significant CMV infection. Similarly, detectable CMV viral DNA alone without initiation of anti-CMV therapy will not be considered as a case for clinically significant CMV infection.

The secondary efficacy endpoints are:

1) Proportion of subjects with clinically significant CMV infection through Week 14 (~100 days) post-transplant.

For this endpoint, case counting will use the same definition as in the primary efficacy endpoint.

2) Time to onset of clinically significant CMV infection through Week 24 (~6 months) post-transplant.

The time to onset of clinically significant CMV infection will be calculated in days, from the day of randomization to the day of onset of CMV end-organ disease or to the day of initiation of anti-CMV PET. For cases where CMV end-organ disease is confirmed by the CAC, the earliest clinical manifestation (sign/symptom) related to the CMV organ involvement will be identified by the CAC as part of their medical review and used as the time of onset of CMV end organ disease. For cases where anti-CMV PET is initiated in the setting of documented viremia (including those applicable cases where CMV end-organ disease was not confirmed by the CAC), the start date of anti-CMV therapy will be used. If both criteria for clinically significant CMV infection are met, the time to onset will be calculated from the day of randomization to the earlier day on which one of the criteria is met.

3) Proportion of subjects with CMV disease through Week 14 post-transplant and Week 24 post-transplant.

For this endpoint, case counting will use the same definition for CMV end-organ disease as in the primary efficacy endpoint.

4) Proportion of subjects with initiation of PET for documented CMV viremia through Week 14 post-transplant and Week 24 post-transplant.

For this endpoint, case counting will use the same definition for initiation of PET for documented CMV viremia as in the primary efficacy endpoint.

5) The time to initiation of PET for documented CMV viremia through Week 24 post-transplant.

The time to initiation of PET for documented CMV viremia will be calculated in days, from the day of randomization to the start date of anti-CMV therapy including those applicable cases where CMV end-organ disease was not confirmed by the CAC.

8.2.3.1.2 Exploratory Endpoints

1 Proportion of subjects with CMV disease through Week 48 post-transplant.

2 Proportion of subjects with all-causes mortality through Week 14 post-transplant, Week 24 post-transplant, and Week 48 post-transplant.

3 Proportion of subjects with opportunistic infection other than CMV infection (i.e., systemic bacterial and invasive fungal infection) through Week 14 post-transplant, Week 24 post-transplant, and Week 48 post-transplant.

4 Proportion of subjects with acute and/or chronic graft-versus-host disease (GVHD) after randomization through Week 14 post-transplant, Week 24 post-transplant, and Week 48 post-transplant.

5 Proportion of subjects with all re-hospitalizations (following initial hospital discharge) and re-hospitalizations for CMV infection/disease through Week 14 post-transplant, Week 24 post-transplant, and Week 48 post-transplant.

6 Proportion of subjects with documented CMV viremia through Week 14 post-transplant, and Week 24 post-transplant.

7 The time to documented CMV viremia through Week 24 post-transplant.

8 Proportion of subjects with engraftment through Week 14 post-transplant and Week 24 post-transplant.

9 The time to onset of engraftment through Week 24 post-transplant.

10 Antiviral resistance to MK-8228 in prophylaxis failures.

11 Quality of life assessment (see Section 7.1.2.10 for details).

12 Pharmacokinetic Endpoints (see Section 4.2.3.3 for details).

13 Pharmacogenetic Endpoints (see Section 4.2.3.4 for details).

8.2.3.2 Safety Endpoints

An initial description of safety measures is provided in Section 4.2.3.2.

All AEs will be collected through 14 days after completion of treatment period (through Follow-up Week 2 Visit). Thereafter, any SAEs related to study medication or SAEs leading to death will be collected through Week 48 post-transplant.

Safety endpoints will be analyzed using a 3-tiered approach (see Section 8.2.5.2).

8.2.4 Analysis Populations

8.2.4.1 Efficacy Analysis Populations

The Full Analysis Set (FAS) population will serve as the primary population for the analysis of efficacy data in this study. The FAS consists of all randomized subjects who receive at least one dose of study medication and had no detectable CMV viral DNA (measured by the central laboratory) on Day 1 (when study therapy is initiated).

A supportive analysis using the Per Protocol (PP) Set will be performed for the primary and key secondary efficacy endpoints. The PP population is a subset of the FAS population and it excludes subjects due to important deviations from the protocol that may substantially affect the results of the primary and key secondary efficacy endpoints. Potential violations that may result in the exclusion of a subject from the PP population include:

- Failure to reasonably adhere to the dosing schedule for the study medication
- Failure to comply with specific inclusion/exclusion criteria
- Use of a prohibited concomitant medication during the treatment period that may impact on the efficacy assessment

The final determination on protocol violations will be made prior to the final unblinding of the database and will be documented in a protocol violator memo.

Subjects will be included in the treatment group to which they are randomized for the analysis of efficacy data using both the FAS and PP populations.

8.2.4.2 Safety Analysis Populations

The All Subjects as Treated (ASaT) population will be used for the analysis of safety data in this study. The ASaT population consists of all randomized subjects who received at least one dose of study treatment. Subjects will be included in the treatment group corresponding to the study treatment they actually received for the analysis of safety data using the ASaT population. For most subjects this will be the treatment group to which they are randomized. Subjects who take incorrect study treatment for the entire treatment period will be included in the treatment group corresponding to the study treatment they actually received.

At least one laboratory or vital sign measurement obtained subsequent to at least one dose of study treatment is required for inclusion in the analysis of each specific parameter. To assess change from baseline, a baseline measurement is also required.

8.2.5 Statistical Methods

8.2.5.1 Statistical Methods for Efficacy Analyses

8.2.5.1.1 Primary Efficacy Analysis

To test the primary hypothesis that MK-8228 is superior to placebo in the prevention of clinically significant CMV infection, the stratum-adjusted Mantel-Haenszel method (with continuity correction) will be used to compare the proportion of subjects with clinically significant CMV infection through Week 24 (~6 months) post-transplant between the two treatment groups. Stratification factors such as high and low risk groups will be included in the primary efficacy analysis. Cochran Mantel-Haenszel weights will be used to calculate the overall between group differences across strata. MK-8228 is concluded superior to placebo if 1-sided p-value is less than or equal to 0.0249 (see Section 8.2.6 for alpha adjustment). Due to the expected large number of study centers, center will not be included as a stratification factor in the primary efficacy analysis, but may be explored as a sensitivity analysis.

The primary efficacy analysis will be performed on the FAS population. A sensitivity analysis including those subjects who had detectable CMV viral DNA on Day 1 will be provided. The primary missing data approach will be the Non-Completer= Failure approach (See Section 8.2.5.1.4 for details). Supportive analyses using PP population and different missing data approaches will also be conducted (see Table 7).

8.2.5.1.2 Secondary Efficacy Analysis

To assess the first secondary endpoint, the proportion of subjects with clinically significant CMV infection through Week 14 (~100 days) post-transplant, the same stratum-adjusted Mantel-Haenszel method as described in the primary efficacy analysis will be performed. No formal hypothesis testing will be done on this endpoint.

Analysis on the second secondary endpoint- time to onset of clinically significant CMV infection through Week 24 (~6 months) post-transplant will be conducted using the non-parametric Kaplan-Meier method. The Kaplan-Meier curve will be plotted by treatment group and p-value for the between group difference in time to onset of clinically significant CMV infection will be provided using the log-rank test.

To assess the third secondary endpoint- proportion of subjects with CMV disease through Week 14 post-transplant and Week 24 post-transplant, stratum-adjusted Mantel-Haenszel method as described in the primary efficacy analysis will be performed. Only CAC confirmed CMV end-organ disease cases will be included in this analysis. Concordance/discordance between CAC and investigator assessment in CMV end-organ disease will be summarized.

To assess the fourth secondary endpoint- proportion of subjects with initiation of PET for documented viremia through Week 14 post-transplant and Week 24 post-transplant, stratum-adjusted Mantel-Haenszel method as described in the primary efficacy analysis will be performed. If there are cases where anti-CMV therapy is initiated with no detectable CMV

DNA using the central laboratory data, a sensitivity analysis will be provided using the local laboratory results. In addition, another sensitivity analysis will be performed for initiation of PET based on viremia using the protocol recommended viral load threshold (see Section 4.2.3.1) instead of any detectable CMV DNA for documented viremia.

Analysis on the fifth secondary endpoint- time to initiation of PET for documented CMV viremia through Week 24 post-transplant will be conducted using the non-parametric Kaplan-Meier method. The Kaplan-Meier curve will be plotted by treatment group and p-value for the between group difference in time to initiation of PET for documented CMV viremia will be provided using the log-rank test.

Table 7 Analysis Strategy for Efficacy Variables

Endpoint/Variable (Description, Time Point)	Primary vs. Supportive Approach†	Statistical Method	Analysis Population	Missing Data Approach*
**Primary Endpoint**				
Proportion of subjects with clinically significant CMV infection through Week 24 (~6 months) post-transplant	P	Stratified M & H‡	FAS	NC=F
	S	Stratified M & H‡	PP	NC=F
	S	Stratified M & H‡	FAS	DAO
**Secondary Endpoints**				
Proportion of subjects with clinically significant CMV infection through Week 14 (~100 days) post-transplant	P	Stratified M & H‡	FAS	NC=F
	S	Stratified M & H‡	PP	NC=F
	S	Stratified M & H‡	FAS	DAO
Time to onset of clinically significant CMV infection through Week 24 (~6 months) post- transplant	P	Kaplan-Meier plot	FAS	Censored at last assessment
Proportion of subjects with CMV disease through Week 14 post-transplant and Week 24 post-transplant	P	Stratified M & H‡	FAS	NC=F
	S	Stratified M & H‡	PP	NC=F
	S	Stratified M & H‡	FAS	DAO
Proportion of subjects with initiation of PET for documented CMV viremia through Week 14 post-transplant and Week 24 post-transplant.	P	Stratified M & H‡	FAS	NC=F
	S	Stratified M & H‡	PP	NC=F
	S	Stratified M & H‡	FAS	DAO
The time to initiation of PET for documented CMV viremia through Week 24 post-transplant.	P	Kaplan-Meier plot	FAS	Censored at last assessment

* NC=F: Non-Completers equal failure. Non-completers refer to subjects who prematurely discontinued from the study. DAO = Data as observed.
† P=Primary approach; S=Secondary/supportive approach.
‡ Stratified Mantel-Haenszel method by high and low risk factors.

8.2.5.1.3 Exploratory Analysis

Summary statistics and 95% confidence intervals will be provided by treatment group for the following exploratory endpoints:

- proportion of subjects with CMV disease through Week 48 post-transplant
- proportion of subjects with all-cause mortality through Week 14 post-transplant, Week 24 post-transplant, and Week 48 post-transplant
- proportion of subjects with opportunistic infection (i.e., systemic bacterial infection or invasive fungal infection) through Week 14 post-transplant, Week 24 post-transplant, and Week 48 post-transplant
- proportion of subjects with GVHD through Week 14 post-transplant, Week 24 post-transplant, and Week 48 post-transplant
- proportion of subjects with all re-hospitalizations (following initial hospital discharge) and re-hospitalizations for CMV infection/disease through Week 14 post-transplant, Week 24 post-transplant, and Week 48 post-transplant.
- proportion of subjects with documented viremia (as measured by the central laboratory) through Week 14 post-transplant and Week 24 post-transplant.
- proportion of subjects with engraftment through Week 14 post-transplant and Week 24 post-transplant.

Kaplan-Meier plots will be provided for the time to documented viremia and the time to onset of engraftment through Week 24 post-transplant.

8.2.5.1.4 Missing Data Handling

The primary missing data approach will be the Non-Completer= Failure approach. Non-completers refer to subjects who prematurely discontinued from the study. A subject who discontinued study medication but remained in the study follow-up will not be considered as a non-completer.

A secondary missing data approach is the Data-As-Observed (DAO). With this approach, any subject with missing value for a particular endpoint will be excluded from the analysis.

8.2.5.2 Statistical Methods for Safety Analyses

Safety and tolerability will be assessed by clinical review of all relevant parameters including adverse experiences, laboratory tests, vital signs, and ECG measurements

The analysis of safety results will follow a 3-tiered approach. The tiers differ with respect to the analyses that will be performed. Safety parameters or adverse events that are identified a priori constitute "Tier 1" safety endpoints that will be subject to inferential testing for statistical significance with p-values and 95% confidence intervals provided for between-group comparisons. Other safety parameters will be considered "Tier 2" or "Tier 3". Tier 2 parameters (requires that at least 4 subjects in each treatment group exhibit the event) will be assessed via point estimates with 95% confidence intervals provided for between-group comparisons; only point estimates by treatment group are provided for Tier 3 safety parameters.

The threshold of at least 4 events was chosen because the 95% confidence interval for the between-group difference in percent incidence will always include zero when treatment groups of equal size each have less than 4 events and thus would add little to the interpretation of potentially meaningful differences. Because many 95% confidence intervals may be provided without adjustment for multiplicity, the confidence intervals should be regarded as a helpful descriptive measure to be used in review, not a formal method for assessing the statistical significance of the between-group differences in adverse experiences and predefined limits of change.

For this protocol, there are no Tier 1 events (see Table 8 below). In addition to the criteria described above, the following will also be analyzed as Tier 2 events: Proportion of subjects with: (1) at least one adverse event; (2) a drug-related adverse event; (3) a serious adverse event; (4) a serious and drug-related adverse event and (5) an adverse event leading to discontinuation.

Table 8 Analysis Strategy for Safety Parameters

Safety Tier	Safety Endpoint	p-Value	95% CI for Treatment Comparison	Descriptive Statistics
Tier 1	No tier 1 event defined in the current protocol	X	X	X
Tier 2	Any AE		X	X
	Any Drug-Related AE		X	X
	Any Serious AE		X	X
	Any Drug-Related Serious AE		X	X
	Discontinuation due to AE		X	X
	Any AE with incidence ≥ 4 subjects in one of the treatment group		X	X
Tier 3	Any AE with incidence < 4 subjects in both treatment groups			X
	Change from Baseline Results (laboratory, ECG, vital signs)			X

AE = adverse event; CI = confidence interval

95% confidence intervals will be based on the method of Miettenen and Nurminen [39]

Missing values will be handled using the Data-As-Observed (DAO) approach.

8.2.5.3 Summaries of Baseline Characteristics, Demographics, and Other Analyses

Demographic and Baseline Characteristics

The comparability of the treatment groups for each relevant characteristic will be assessed by the use of tables. No statistical hypothesis tests will be performed on these characteristics. The number and percentage of subjects screened, randomized, the primary reasons for screen failure, and the primary reason for discontinuation will be displayed. Demographic variables (e.g., age, gender, and high and low risk), indication for HSCT, prior and concomitant therapies will be summarized by treatment either by descriptive statistics or categorical tables.

PK/PD

Based on pharmacokinetic data obtained within this study, population PK and PK/PD analyses will be performed. The prospective details of this analysis will be specified in a separate modeling analysis plan.

Quality of Life Analysis

Standard algorithms will be used to compute total and subscale scores for the FACT-BMT and EQ-5D questionnaires as specified by the instrument developers. This trial is not powered to detect statistically significant differences in QOL scores between the treatment arms. Therefore, the analysis plan for the QOL instruments will be primarily descriptive in nature.

Questionnaire data will be analyzed using summary statistics at each administration time point by treatment arm. Actual subject information collected will be used with no imputations for missing data. Simple t-tests may be conducted post-hoc to calculate if there is a statistically significant difference in Quality of life scores at each time point of measurement between the treatment arms. Multivariate models may also be developed to understand subject risk factors that are significantly associated with patient quality of life scores.

8.2.6 Multiplicity

This study has one interim futility analysis, periodic interim safety reviews and one primary hypothesis testing at the end. Since stopping for futility and periodic safety checks do not inflate type I error rate, no alpha spending is planned for these analyses.

However, although there is no intention for stopping for overwhelming efficacy at the interim time, a small amount of alpha ($\alpha = 0.0001$) will be allocated for statistical rigor. At the end of the trial, a 1-sided p-value that is less than or equal to 0.0249 will be used for declaring efficacy success.

8.2.7 Sample Size and Power Calculations

8.2.7.1 Sample Size and Power for Efficacy Analysis

Based on literature data for placebo and on the results from the Phase II study AIC246-01-II-02, the incidence rate of clinically significant CMV infection for subjects receiving placebo is expected to be approximately 35%. It is expected that the MK-8228 arm will reduce this incidence by half to an incidence of approximately 17%. It is further anticipated that about 20% of subjects will be discontinued from trial for both treatment arms for reasons other than virologic failure. Since the primary missing data approach will be noncompleter = failure approach, 20% was added to the expected incidence of clinically significant CMV infection for the placebo arm (55%) and the MK-8228 arm (37%) for sample size and power calculations.

A sample size of approximately 540 subjects is planned using a 2:1 randomization ratio (~360 subjects in the MK-8228 arm and ~180 subjects in the placebo arm). Excluding 15% subjects with detectable CMV DNA on Day 1, the evaluable number of subjects in the FAS population is 459 in total (306 in the MK-8228 arm and 153 in the placebo arm). An Interim futility analysis will be conducted when approximately 40% of the subjects have completed treatment or discontinued prior to completing treatment. With the current sample size, this study will have 90.5% overall power. If the study were designed without the futility analysis, the power would be 95%.

8.2.7.2 Sample Size and Power for Safety Analysis

Table 9 gives the upper bound of the two-sided 95% Clopper-Pearson exact confidence interval for the true proportion of subjects with a particular adverse experience corresponding to various observed numbers of subjects with such adverse experience in a sample of 360 subjects and 180 subjects. For example, if a particular adverse experience is not observed in any of the 360 subjects in the MK-8228 arm, then we can conclude with 95% confidence that the true proportion is no more than 1.0%.

Table 9 Upper Bound of the Two-Sided 95% Confidence Interval for the True Proportion of Subjects with an AE

n	Observed Number of Subjects With AE (%)	95% Upper Bound for the True Proportion (%)
360	0 (0.0)	1.0
	12 (3.3)	5.7
	24 (6.7)	9.8
	36 (10.0)	13.6
180	0 (0.0)	2.0
	6 (3.3)	7.1
	12 (6.7))	11.4
	18 (10.0)	15.3

8.2.8 Subgroup Analyses and Effects of Baseline Factors

To determine whether the treatment effect is consistent across various subgroups, the estimate of the treatment effect (with a nominal 95% CI) for the primary efficacy endpoint will be estimated within each category of the following classification variables:

- Gender (Male, Female)
- Age (use median age cutoff)
- Race (white, black, Asian, other)
- Ethnicity (Hispanic, Not Hispanic)
- High and low risk

The consistency of the treatment effect will be assessed descriptively via summary statistics by category for the classification variables listed above. Other clinically relevant variables may be identified for which additional subgroup analyses may be performed.

8.2.9 Interim Analyses

An interim futility analysis will be conducted when 40% of randomized subjects either complete study therapy through Week 14 (~100 days) post-transplant or discontinue prior to that time point. The non-binding stopping boundary for the futility analysis will be based on the Hwang-Shih-DeCani spending function with gamma = 3.1. This trial may be stopped for futility at the IA if the 1-sided p-value for comparing MK-8228 to placebo is greater than 0.196. This p-value boundary corresponds to an approximate difference of 6.6 percentage points in the observed incidence of clinically significant CMV infection between the two groups. Under the null hypothesis of no treatment difference, the probability of correctly stopping the trial for futility is approximately 80%. Under the alternative hypothesis, the probability of incorrectly stopping the trial is approximately 7%.

In addition, periodic safety reviews will be conducted. The first safety review will occur when 10% of randomized subjects either complete study therapy through Week 14 (~100 days) post-transplant or discontinue prior to that time point. Thereafter, the DMC will review all safety data approximately every 6 months during the trial (or at longer time intervals in case of slow enrollment).

The interim analyses will be performed by an external independent statistician and the results will be shared with the DMC members. The DMC recommendations will be communicated to the Sponsor as specified in the DMC Charter.

Blinding to treatment assignment will be maintained at all investigational sites. The results of the interim analyses will not be shared with the investigators prior to the completion of the trial. The Executive Oversight Committee (EOC) of the sponsor will receive recommendations throughout the trial from the DMC and is responsible for acting upon the recommendations of the DMC. The EOC will not have access to unblinded data or reports unless it is deemed necessary by the DMC to have this information in order to be able to act upon a DMC recommendation. The EOC will be completely independent of, and separate

from, the trial team performing the medical monitoring and supervising the operational aspects of the protocol.

The external unblinded statistician who will prepare the analyses will serve as a non-voting member of the DMC. This individual will not be involved in any discussions regarding modifications to the protocol, statistical methods, identification of protocol violations, or data validation efforts after the interim analyses.

8.2.10 Compliance/Medication Adherence

Drug accountability data for MK-8228 and the corresponding placebo will be collected during the study. A day within the study will be considered an "On-Therapy" day if the subject takes at least one dose. For a subject who is followed for the entire study period, the "Number of Days Should be on Therapy" is the total number of days from randomization to the last scheduled day for treatment administration for that subject. For a subject who discontinued from the study medication, the "Number of Days Should be on Therapy" is the total number of days from randomization to the date of the last dose of study medication.

For each subject, percent compliance will then be calculated using the following formula:

$$\text{Percent Compliance} = \frac{\text{Number of Days on Therapy}}{\text{Number of Days Should be on Therapy}} \text{ x } 100.$$

Compliance rates will be summarized for each treatment group and individual compliance rates will factor into the identification of protocol violators as discussed in Section 8.2.4.

In addition, percent of subjects on CsA and duration of CsA use will be reported.

8.2.11 Extent of Exposure

The extent of exposure to study treatment will be evaluated by summary statistics for the "Number of Days on Therapy" by treatment group.

9.0 LABELING, PACKAGING, STORAGE AND RETURN OF CLINICAL SUPPLIES

9.1 Investigational Product

The investigator shall take responsibility for and shall take all steps to maintain appropriate records and ensure appropriate supply, storage, handling, distribution and usage of investigational product in accordance with the protocol and any applicable laws and regulations.

Clinical Supplies will be provided by the Sponsor as summarized in Table 10.

Table 10 Product Descriptions

Product Name & Potency	Dosage Form
MK-8228 480 mg or Placebo	Tablet
MK-8228 240 mg or Placebo	Tablet
MK-8228 240 mg	IV

9.2 Packaging and Labeling Information

Clinical supplies will be affixed with a clinical label in accordance with regulatory requirements.

For the tablet formulation, subjects will receive blinded bottles (each containing 1 week supply of study therapy). No kitting is required.

For the IV formulation, open-label vials of MK-8228 will be supplied. The IV formulation will be dispensed in a blinded fashion by an unblinded pharmacist (or qualified trial site personnel. The MK-8228 intravenous formulation dosing volume will be 250 mL and duration of infusion will be 60 minutes. The Sponsor will provide opaque covers for the IV bags to assist with blinding the study therapy. Because this is a double-blind study, the investigator, study personnel, and subject must remain blinded to the IV study therapy. In order to maintain the blinding, the unblinded pharmacist (or qualified study site personnel designated to prepare the IV supplies) will be responsible solely for the preparation and administration of the IV study therapy. He/she will not be involved in evaluating subjects for efficacy or safety.

9.3 Clinical Supplies Disclosure

The central electronic randomization system (IVRS/IWRS) should be used in order to unblind subjects and to unmask drug identity. The Sponsor will not provide random code/disclosure envelopes or lists with the clinical supplies.

9.4 Storage and Handling Requirements

Clinical supplies must be stored in a secure, limited-access location under the storage conditions specified on the label.

Receipt and dispensing of trial medication must be recorded by an authorized person at the trial site.

Clinical supplies may not be used for any purpose other than that stated in the protocol.

9.5 Returns and Reconciliation

The investigator is responsible for keeping accurate records of the clinical supplies received from the Sponsor or designee, the amount dispensed to and returned by the subjects and the amount remaining at the conclusion of the trial.

For all trial sites, the local country Sponsor personnel or designee will provide appropriate documentation that must be completed for drug accountability and return.

9.6 Standard Policies

Trial site personnel will have access to a central electronic randomization system (IVRS/IWRS system) to allocate subjects, to assign drug to subjects and to manage the distribution of clinical supplies. Each person accessing the IVRS system must be assigned an individual unique PIN. They must use only their assigned PIN to access the system, and they must not share their assigned PIN with anyone.

10.0 ADMINISTRATIVE AND REGULATORY DETAILS

10.1 Confidentiality

10.1.1 Confidentiality of Data

By signing this protocol, the investigator affirms to the Sponsor that information furnished to the investigator by the Sponsor will be maintained in confidence, and such information will be divulged to the institutional review board, ethics review committee (IRB/ERC) or similar or expert committee; affiliated institution and employees, only under an appropriate understanding of confidentiality with such board or committee, affiliated institution and employees. Data generated by this trial will be considered confidential by the investigator, except to the extent that it is included in a publication as provided in the Publications section of this protocol.

10.1.2 Confidentiality of Subject Records

By signing this protocol, the investigator agrees that the Sponsor (or Sponsor representative), IRB/ERC, or regulatory authority representatives may consult and/or copy trial documents in order to verify worksheet/case report form data. By signing the consent form, the subject agrees to this process. If trial documents will be photocopied during the process of verifying worksheet/case report form information, the subject will be identified by unique code only; full names/initials will be masked prior to transmission to the Sponsor.

By signing this protocol, the investigator agrees to treat all subject data used and disclosed in connection with this trial in accordance with all applicable privacy laws, rules and regulations.

10.1.3 Confidentiality of Investigator Information

By signing this protocol, the investigator recognizes that certain personal identifying information with respect to the investigator, and all subinvestigators and trial site personnel, may be used and disclosed for trial management purposes, as part of a regulatory submissions, and as required by law. This information may include:

- name, address, telephone number and e-mail address;

- hospital or clinic address and telephone number;
- curriculum vitae or other summary of qualifications and credentials; and
- other professional documentation.

Consistent with the purposes described above, this information may be transmitted to the Sponsor, and subsidiaries, affiliates and agents of the Sponsor, in your country and other countries, including countries that do not have laws protecting such information. Additionally, the investigator's name and business contact information may be included when reporting certain serious adverse events to regulatory authorities or to other investigators. By signing this protocol, the investigator expressly consents to these uses and disclosures.

If this is a multicenter trial, in order to facilitate contact between investigators, the Sponsor may share an investigator's name and contact information with other participating investigators upon request.

10.1.4 Confidentiality of IRB/IEC Information

The Sponsor is required to record the name and address of each IRB/IEC member that reviews and approves this trial. The Sponsor is also required to document that each IRB/IEC meets regulatory and ICH GCP requirements by requesting and maintaining records of the names and qualifications of the IRB/IEC members and to make these records available for regulatory agency review upon request by those agencies.

10.2 Compliance with Financial Disclosure Requirements

Financial Disclosure requirements are outlined in the US Food and Drug Administration Regulations, Financial Disclosure by Clinical Investigators (21 CFR Part 54). It is the Sponsor's responsibility to determine, based on these regulations, whether a request for Financial Disclosure information is required. It is the investigator's/subinvestigator's responsibility to comply with any such request.

The investigator/subinvestigator(s) agree, if requested by the Sponsor in accordance with 21 CFR Part 54, to provide his/her financial interests in and/or arrangements with the Sponsor to allow for the submission of complete and accurate certification and disclosure statements. The investigator/subinvestigator(s) further agree to provide this information on a Certification/Disclosure Form, commonly known as a financial disclosure form, provided by the Sponsor or through a secure password-protected electronic portal provided by the Sponsor. The investigator/subinvestigator(s) also consent to the transmission of this information to the Sponsor in the United States for these purposes. This may involve the transmission of information to countries that do not have laws protecting personal data.

10.3 Compliance with Law, Audit and Debarment

By signing this protocol, the investigator agrees to conduct the trial in an efficient and diligent manner and in conformance with this protocol; generally accepted standards of Good Clinical Practice (e.g., International Conference on Harmonization of Technical Requirements for Registration of Pharmaceuticals for Human Use Good Clinical Practice: Consolidated Guideline and other generally accepted standards of good clinical practice); and all applicable federal, state and local laws, rules and regulations relating to the conduct of the clinical trial.

The Code of Conduct, a collection of goals and considerations that govern the ethical and scientific conduct of clinical investigations sponsored by Merck, is provided in Section 12.1 - Merck Code of Conduct for Clinical Trials.

The investigator also agrees to allow monitoring, audits, IRB/ERC review and regulatory authority inspection of trial-related documents and procedures and provide for direct access to all trial-related source data and documents.

The investigator agrees not to seek reimbursement from subjects, their insurance providers or from government programs for procedures included as part of the trial reimbursed to the investigator by the Sponsor.

The investigator shall prepare and maintain complete and accurate trial documentation in compliance with Good Clinical Practice standards and applicable federal, state and local laws, rules and regulations; and, for each subject participating in the trial, provide all data, and, upon completion or termination of the clinical trial, submit any other reports to the Sponsor as required by this protocol or as otherwise required pursuant to any agreement with the Sponsor.

Trial documentation will be promptly and fully disclosed to the Sponsor by the investigator upon request and also shall be made available at the trial site upon request for inspection, copying, review and audit at reasonable times by representatives of the Sponsor or any regulatory authorities. The investigator agrees to promptly take any reasonable steps that are requested by the Sponsor as a result of an audit to cure deficiencies in the trial documentation and worksheets/case report forms.

The investigator must maintain copies of all documentation and records relating to the conduct of the trial in compliance with all applicable legal and regulatory requirements. This documentation includes, but is not limited to, the protocol, worksheets/case report forms, advertising for subject participation, adverse event reports, subject source data, correspondence with regulatory authorities and IRBs/ERCs, consent forms, investigator's curricula vitae, monitor visit logs, laboratory reference ranges, laboratory certification or quality control procedures and laboratory director curriculum vitae. By signing this protocol, the investigator agrees that documentation shall be retained until at least 2 years after the last approval of a marketing application in an ICH region or until there are no pending or contemplated marketing applications in an ICH region or until at least 2 years have elapsed since the formal discontinuation of clinical development of the investigational product.

Because the clinical development and marketing application process is variable, it is anticipated that the retention period can be up to 15 years or longer after protocol database lock. The Sponsor will determine the minimum retention period and notify the investigator when documents may be destroyed. The sponsor also recognizes that documents may need to be retained for a longer period if required by local regulatory requirements. All trial documents shall be made available if required by relevant regulatory authorities. The investigator must consult with and obtain written approval by the Sponsor prior to discarding trial and/or subject files.

ICH Good Clinical Practice guidelines recommend that the investigator inform the subject's primary physician about the subject's participation in the trial if the subject has a primary physician and if the subject agrees to the primary physician being informed.

The investigator will promptly inform the Sponsor of any regulatory authority inspection conducted for this trial.

Persons debarred from conducting or working on clinical trials by any court or regulatory authority will not be allowed to conduct or work on this Sponsor's trials. The investigator will immediately disclose in writing to the Sponsor if any person who is involved in conducting the trial is debarred or if any proceeding for debarment is pending or, to the best of the investigator's knowledge, threatened.

In the event the Sponsor prematurely terminates a particular trial site, the Sponsor will promptly notify that trial site's IRB/IEC.

According to European legislation, a Sponsor must designate an overall coordinating investigator for a multi-center trial (including multinational). When more than one trial site is open in an EU country, Merck, as the Sponsor, will designate, per country, a national principal coordinator (Protocol CI), responsible for coordinating the work of the principal investigators at the different trial sites in that Member State, according to national regulations. For a single-center trial, the Protocol CI is the principal investigator. In addition, the Sponsor must designate a principal or coordinating investigator to review the trial report that summarizes the trial results and confirm that, to the best of his/her knowledge, the report accurately describes the conduct and results of the trial [Clinical Study Report (CSR) CI]. The Sponsor may consider one or more factors in the selection of the individual to serve as the Protocol CI and or CSR CI (e.g., availability of the CI during the anticipated review process, thorough understanding of clinical trial methods, appropriate enrollment of subject cohort, timely achievement of trial milestones). The Protocol CI must be a participating trial investigator.

10.4 Compliance with Trial Registration and Results Posting Requirements

Under the terms of the Food and Drug Administration Modernization Act (FDAMA) and the Food and Drug Administration Amendments Act (FDAAA), the Sponsor of the trial is solely responsible for determining whether the trial and its results are subject to the requirements for submission to the Clinical Trials Data Bank, http://www.clinicaltrials.gov. Merck, as Sponsor of this trial, will review this protocol and submit the information necessary to fulfill

these requirements. Merck entries are not limited to FDAMA/FDAAA mandated trials. Information posted will allow subjects to identify potentially appropriate trials for their disease conditions and pursue participation by calling a central contact number for further information on appropriate trial locations and trial site contact information.

By signing this protocol, the investigator acknowledges that the statutory obligations under FDAMA/FDAAA are that of the Sponsor and agrees not to submit any information about this trial or its results to the Clinical Trials Data Bank.

10.5 Quality Management System

By signing this protocol, the Sponsor agrees to be responsible for implementing and maintaining a quality management system with written development procedures and functional area standard operating procedures (SOPs) to ensure that trials are conducted and data are generated, documented, and reported in compliance with the protocol, accepted standards of Good Clinical Practice, and all applicable federal, state, and local laws, rules and regulations relating to the conduct of the clinical trial.

10.6 Data Management

The investigator or qualified designee is responsible for recording and verifying the accuracy of subject data. By signing this protocol, the investigator acknowledges that his/her electronic signature is the legally binding equivalent of a written signature. By entering his/her electronic signature, the investigator confirms that all recorded data have been verified as accurate.

Detailed information regarding Data Management procedures for this protocol will be provided by the Sponsor.

10.7 Publications

This trial is intended for publication, even if terminated prematurely. Publication may include any or all of the following: posting of a synopsis online, abstract and/or presentation at a scientific conference, or publication of a full manuscript. The Sponsor will work with the authors to submit a manuscript describing trial results within 12 months after the last data become available, which may take up to several months after the last subject visit in some cases such as vaccine trials. However, manuscript submission timelines may be extended on OTC trials. For trials intended for pediatric-related regulatory filings, the investigator agrees to delay publication of the trial results until the Sponsor notifies the investigator that all relevant regulatory authority decisions on the trial drug have been made with regard to pediatric-related regulatory filings. Merck will post a synopsis of trial results for approved products on www.clinicaltrials.gov by 12 months after the last subject's last visit for the primary outcome, 12 months after the decision to discontinue development, or product marketing (dispensed, administered, delivered or promoted), whichever is later.

These timelines may be extended for products that are not yet marketed, if additional time is needed for analysis, to protect intellectual property, or to comply with confidentiality agreements with other parties. Authors of the primary results manuscript will be provided the complete results from the Clinical Study Report, subject to the confidentiality agreement. When a manuscript is submitted to a biomedical journal, the Sponsor's policy is to also include the protocol and statistical analysis plan to facilitate the peer and editorial review of the manuscript. If the manuscript is subsequently accepted for publication, the Sponsor will allow the journal, if it so desires, to post on its website the key sections of the protocol that are relevant to evaluating the trial, specifically those sections describing the trial objectives and hypotheses, the subject inclusion and exclusion criteria, the trial design and procedures, the efficacy and safety measures, the statistical analysis plan, and any amendments relating to those sections. The Sponsor reserves the right to redact proprietary information.

For multicenter trials, subsequent to the multicenter publication (or after public disclosure of the results online at www.clinicaltrials.gov if a multicenter manuscript is not planned), an investigator and his/her colleagues may publish their data independently. In most cases, publication of individual trial site data does not add value to complete multicenter results, due to statistical concerns. In rare cases, publication of single trial site data prior to the main paper may be of value. Limitations of single trial site observations in a multicenter trial should always be described in such a manuscript.

Authorship credit should be based on 1) substantial contributions to conception and design, or acquisition of data, or analysis and interpretation of data; 2) drafting the article or revising it critically for important intellectual content; and 3) final approval of the version to be published. Authors must meet conditions 1, 2 and 3. Significant contributions to trial execution may also be taken into account to determine authorship, provided that contributions have also been made to all three of the preceding authorship criteria. Although publication planning may begin before conducting the trial, final decisions on authorship and the order of authors' names will be made based on participation and actual contributions to the trial and writing, as discussed above. The first author is responsible for defending the integrity of the data, method(s) of data analysis and the scientific content of the manuscript.

The Sponsor must have the opportunity to review all proposed abstracts, manuscripts or presentations regarding this trial 45 days prior to submission for publication/presentation. Any information identified by the Sponsor as confidential must be deleted prior to submission; this confidentiality does not include efficacy and safety results. Sponsor review can be expedited to meet publication timelines.

11.0 LIST OF REFERENCES

1. Ljungman P, Griffiths P, Paya C. Definitions of cytomegalovirus infection and disease in transplant recipients. Clin Infect Dis 2002;34:1094-7.

2. Boeckh M. Complications, diagnosis, management, and prevention of CMV infections: Current and future. Am Soc Hematol 2011:305-9.

3. Boeckh M, Geballe AP. Cytomegalovirus: Pathogen, paradigm, and puzzle. J Clin Invest 2011;121(5):1673-80.

4. Boeckh M, Nichols WG. The impact of cytomegalovirus serostatus of donor and recipient before hematopoietic stem cell transplantation in the era of antiviral prophylaxis and preemptive therapy. Blood 2004;103:2003-8.

5. Nakamae H, Kirby KA, Sandmaier BM, Norasetthada L, Maloney DG, Maris MB. Effect of conditioning regimen intensity on cmv infection in allogeneic hematopoietic cell transplantation. Biol Blood Marrow Transplant 2009;15:694-703.

6. Marty FM, Ljungman P, Papanicolaou GA, Winston DJ, Chemaly RF, Strasfeld L, et al. Maribavir prophylaxis for prevention of cytomegalovirus disease in recipients of allogeneic stem-cell transplants: A phase 3, double-blind, placebo-controlled, randomised trial. Lancet Infect Dis 2011;11:284-92.

7. Sauter C, Abbound M, Jia X, Heller G, Gonzales A, Lubin M, et al. Serious infection risk and immune recovery after double-unit cord blood transplantation without antithymocyte globulin. Biol Blood Marrow Transplant 2011;17:1460-71.

8. Pollack M, Heugel J, Xie H, Leisenring W, Storek J, Young J, et al. An international comparison of current strategies to prevent herpesvirus and fungal infections in hematopoietic cell transplant recipients. Biol Blood Marrow Transplant 2011;17:664-73.

9. Boeckh M, Ljungman P. How we treat cytomegalovirus in hemtopoietic cell transplant recipients. Blood 2009;113:5711-9.

10. Biron KK. Antiviral drugs for cytomegalovirus diseases. Antiviral Res 2006;71:154-63.

11. Lurain NS, Chou S. Antiviral drug resistance of human cytomegalovirus. Clin Microbiol Rev 2010;23(4):689-712.

12. Lischka P, Hewlett G, Wunberg T, Baumeister J, Paulsen D, Goldner T, et al. In vitro and in vivo activities of the novel anticytomegalovirus compound AIC246. Antimicrob Agents Ch 2010;54(3):1290-7.

13. Goldner T, Hewlett G, Ettischer N, Ruebsamen-Schaeff H, Zimmerman H, Lischka P. The novel anticytomegalovirus compound AIC246 (letermovir) inhibits human cytomegalovirus replication through a specific antiviral mechanism that involves the viral terminase. J Virol 2011;85(20):10884-93.

14. Kaul DR, Stoelben S, Cober E, Ojo T, Sandusky E, Lischka P, et al. First report of successful treatment of multidrug-resistant cytomegalovirus disease with the novel anti-CMV compound AIC246. Am J Transplant 2011;11:1079-84.

15. Boeckh M, Leisenring W, Riddell SR, Bowden RA, Huang M-L, Myerson D, et al. Late cytomegalovirus disease and mortality in recipients of allogeneic hematopoietic stem cell transplants: Importance of viral load and T-cell immunity. Blood 2003;101(2):407-14.

16. Krause H, Hebart H, Jahn G, Müller CA, Einsele H. Screening for CMV-specific T cell proliferation to identify patients at risk of developing late onset CMV disease. Bone Marrow Transpl 1997;19:1111-6.

17. Zaia JA, Gallez-Hawkins M, Tegtmeier BR, ter Veer A, Li X, Niland JC, et al. Late cytomegalovirus disease in marrow transplantation is predicted by virus load in plasma. J Infect Dis 1997;176:782-5.

18. Broers AEC, van der Holt R, van Esser JWJ, Gratama J-W, Henzen-Logmans S, Kuenen-Boumeester V, et al. Increased transplant-related morbidity and mortality in CMV-seropositive patients despite highly effective prevention of CMVdisease after allogeneic T-cell-depleted stem cell transplantation. Blood 2000;95(7):2240-5.

19. Craddock C, Szydlo RM, Dazzi F, Olavarria E, Cwynarski K, Yong A, et al. Cytomegalovirus seropositivity adversely influences outcome after T-depleted unrelated donor transplant in patients with chronic myeloid leukaemia: The case for tailored graft-versus-host disease prophylaxis. Br J Haematol 2001;112:228-36.

20. Meijer E, Dekker AW, Rozenberg-Arska M, Weersink AJL, Verdonck LF. Influence of cytomegalovirus seropositivity on outcome after T cell-depleted bone marrow transplantation: Contrasting results between recipients of grafts from related and unrelated donors. Clin Infect Dis 2002;35:703-12.

21. Ljungman P, Perez-Bercoff L, Jonsson J, Avetisyan G, Sparrelid E, Aschan J, et al. Risk factors for the development of cytomegalovirus disease after allogeneic stem cell transplantation. Haematologica 2006;91(1):78-83.

22. Miller W, Flynn P, McCullough J, Balfour Jr. HH, Goldman A, Haake R, et al. Cytomegalovirus infection after bone marrow transplantation: An association with acute graft-v-host disease. Blood 1986;67(4):1162-7.

23. Özdemir E, Saliba RM, Champlin RE, Couriel DR, Giralt SA, de Lima M. Risk factors associated with late cytomegalovirus reactivation after allogeneic stem cell transplantation for hematological malignancies. Bone Marrow Transpl 2007;40:125-36.

24. Martino R, Rovira M, Carreras E, Solano C, Sierra J, De La Rubia J, et al. Severe infections after allogeneic peripheral blood stem cell transplantation: a matched-pair comparison of unmanipulated and CD34+ cell-selected transplantation. Haematologica 2001;86:1075-86.

25. Söderberg C, Larsson S, Bergstedt-Lindqvist S, Möller E. Definition of a subset of human peripheral blood mononuclear cells that are permissive to human cytomegalovirus infection. J Virol 1993;67(6):3166-75.

26. Larsson K, Aschan J, Remberger M, Ringdén O, Winiarski J, Ljungman P. Reduced risk for extensive chronic graft-versus-host disease in patients receiving transplants with human leukocyte antigen-identical sibling donors given polymerase chain reaction-based preemptive therapy against cytomegalovirus. Transplantation 2004;77(4):526-31.

27. Marty FM, Boeckh M. Maribavir and human cytomegalovirus-what happened in the clinical trials and why might the drug have failed? Curr Opin Vir 2011;1:555-62.

28. Boeckh M, Bowden RA, Goodrich JM, Pettinger M, Meyers JD. Cytomegalovirus antigen detection in peripheral blood leukocytes after allogeneic marrow transplantation. Blood 1992;80(5):1358-64.

29. Winston DJ, Young JH, Pullarkat V, Papanicolaou GA, Vij R, Vance E, et al. Maribavir prophylaxis for prevention of cytomegalovirus infection in allogeneic stem cell transplant recipients: a multicenter, randomized, double-blind, placebo-controlled, dose-ranging study. Blood 2008;111(11):5403-10.

30. Ljungman P, Aschan J, Lewensohn-Fuchs I, Carlens S, Larsson K, Lönnqvist B, et al. Results of different strategies for reducing cytomegalovirus-associated mortality in allogeneic stem cell transplant recipients. Transplantation 1998;66(10):1330-4.

31. Snydman DR. Why did maribavir fail in stem-cell transplants? Lancet Infect Dis 2011;11:255-7.

32. Gerna G, Lilleri D, Furione M, Baldanti F. Management of human cytomegalovirus infection in transplantation: Validation of virologic cut-offs for preemptive therapy and immunological cut-offs for protection. New Microbiol 2011;34:229-54.

33. Lowance D, Neumayer H-H, Legendre CM, Squifflet J-P, Kovarik J, Brennan PJ, et al. Valacyclovir for the prevention of cytomegalovirus disease after renal transplantation. N Engl J Med 1999;340:1462-70.

34. Gor D, Sabin C, Prentice HG, Vyas N, Man S, Griffiths PD, et al. Longitudinal fluctuations in cytomegalovirus load in bone marrow transplant patients: Relationship between peak virus load, donor/recipient serostatus, acute gvhd and cmv disease. Bone Marrow Transpl 1998;21:597-605.

35. Atkinson C, Emery VC. Cytomegalovirus quantification: Where to next in optimising patient management? J Clin Virol 2011;51:219-24.

36. Härter G, Michel D. Antiviral treatment of cytomegalovirus infection: An update. Expert Opin Pharmacother 2012;13(5):623-7.

37. Emery VC, Sabin CA, Coppe AV, Gor D, Hassan-Walker AF, Griffiths PD. Application of viral-load kinetics to identify patients who develop cytomegalovirus disease after transplantation. Lancet 2000;355:2032-6.

38. Neofytos D, Lombardi LR, Shields RK, Ostrander D, Warren L, Nguyen MH, et al. Administration of Voriconazole in Patients With Renal Dysfunction. Clin Infect Dis 2012;54(7):913-21.

39. Miettinen O, Nurminen M. Comparative analysis of two rates. Stat Med 1985;4:213-26.

12.0 APPENDICES

12.1 Merck Code of Conduct for Clinical Trials

Merck*
Code of Conduct for Clinical Trials

I. Introduction

A. Purpose

Merck, through its subsidiaries, conducts clinical trials worldwide to evaluate the safety and effectiveness of our products. As such, we are committed to designing, implementing, conducting, analyzing and reporting these trials in compliance with the highest ethical and scientific standards. Protection of subject safety is the overriding concern in the design of clinical trials. In all cases, Merck clinical trials will be conducted in compliance with local and/or national regulations and in accordance with the ethical principles that have their origin in the Declaration of Helsinki.

B. Scope

Such standards shall be endorsed for all clinical interventional investigations sponsored by Merck irrespective of the party (parties) employed for their execution (e.g., contract research organizations, collaborative research efforts). This Code is not intended to apply to trials which are observational in nature, or which are retrospective. Further, this Code does not apply to investigator-initiated trials which are not under the control of Merck.

II. Scientific Issues

A. Trial Conduct

1. Trial Design

Except for pilot or estimation trials, clinical trial protocols will be hypothesis-driven to assess safety, efficacy and/or pharmacokinetic or pharmacodynamic indices of Merck or comparator products. Alternatively, Merck may conduct outcomes research trials, trials to assess or validate various endpoint measures, or trials to determine subject preferences, etc.

The design (i.e., subject population, duration, statistical power) must be adequate to address the specific purpose of the trial. Research subjects must meet protocol entry criteria to be enrolled in the trial.

2. Site Selection

Merck selects investigative sites based on medical expertise, access to appropriate subjects, adequacy of facilities and staff, previous performance in Merck trials, as well as budgetary considerations. Prior to trial initiation, sites are evaluated by Merck personnel to assess the ability to successfully conduct the trial.

3. Site Monitoring/Scientific Integrity

Trial sites are monitored to assess compliance with the trial protocol and general principles of Good Clinical Practice. Merck reviews clinical data for accuracy, completeness and consistency. Data are verified versus source documentation according to standard operating procedures. Per Merck policies and procedures, if fraud, misconduct or serious GCP-non-Compliance are suspected, the issues are promptly investigated. When necessary, the clinical site will be closed, the responsible regulatory authorities and ethics review committees notified and data disclosed accordingly.

B. Publication and Authorship

To the extent scientifically appropriate, Merck seeks to publish the results of trials it conducts. Some early phase or pilot trials are intended to be hypothesis-generating rather than hypothesis testing. In such cases, publication of results may not be appropriate since the trial may be underpowered and the analyses complicated by statistical issues of multiplicity.

Merck's policy on authorship is consistent with the requirements outlined in the ICH-Good Clinical Practice guidelines. In summary, authorship should reflect significant contribution to the design and conduct of the trial, performance or interpretation of the analysis, and/or writing of the manuscript. All named authors must be able to defend the trial results and conclusions. Merck funding of a trial will be acknowledged in publications.

III. Subject Protection

A. IRB/ERC review

All clinical trials will be reviewed and approved by an independent IRB/ERC before being initiated at each site. Significant changes or revisions to the protocol will be approved by the IRB/ERC prior to implementation, except that changes required urgently to protect subject safety and well-being may be enacted in anticipation of IRB/ERC approval. For each site, the IRB/ERC and Merck will approve the subject informed consent form.

B. Safety

The guiding principle in decision-making in clinical trials is that subject welfare is of primary importance. Potential subjects will be informed of the risks and benefits of, as well as alternatives to, trial participation. At a minimum, trial designs will take into account the local standard of care. Subjects are never denied access to appropriate medical care based on participation in a Merck clinical trial.

All participation in Merck clinical trials is voluntary. Subjects are enrolled only after providing informed consent for participation. Subjects may withdraw from a Merck trial at any time, without any influence on their access to, or receipt of, medical care that may otherwise be available to them.

C. Confidentiality

Merck is committed to safeguarding subject confidentiality, to the greatest extent possible. Unless required by law, only the investigator, sponsor (or representative) and/or regulatory authorities will have access to confidential medical records that might identify the research subject by name.

D. Genomic Research

Genomic Research will only be conducted in accordance with informed consent and/or as specifically authorized by an Ethics Committee.

IV. Financial Considerations

A. Payments to Investigators

Clinical trials are time- and labor-intensive. It is Merck's policy to compensate investigators (or the sponsoring institution) in a fair manner for the work performed in support of Merck trials. Merck does not pay incentives to enroll subjects in its trials. However, when enrollment is particularly challenging, additional payments may be made to compensate for the time spent in extra recruiting efforts.

Merck does not pay for subject referrals. However, Merck may compensate referring physicians for time spent on chart review to identify potentially eligible subjects.

B. Clinical Research Funding

Informed consent forms will disclose that the trial is sponsored by Merck, and that the investigator or sponsoring institution is being paid or provided a grant for performing the trial. However, the local IRB/ERC may wish to alter the wording of the disclosure statement to be consistent with financial practices at that institution. As noted above, publications resulting from Merck trials will indicate Merck as a source of funding.

C. Funding for Travel and Other Requests

Funding of travel by investigators and support staff (e.g., to scientific meetings, investigator meetings, etc.) will be consistent with local guidelines and practices including, in the U.S., those established by the American Medical Association (AMA).

V. Investigator Commitment

Investigators will be expected to review Merck's Code of Conduct as an appendix to the trial protocol, and in signing the protocol, agree to support these ethical and scientific standards.

* In this document, "Merck" refers to Merck Sharp & Dohme Corp. and Schering Corporation, each of which is a subsidiary of Merck & Co., Inc. Merck is known as MSD outside of the United States and Canada. As warranted by context, Merck also includes affiliates and subsidiaries of Merck & Co., Inc."

12.2 Collection and Management of Specimens for Future Biomedical Research

1. Definitions

a. Biomarker: A biological molecule found in blood, other body fluids, or tissues that is a sign of a normal or abnormal process or of a condition or disease. A biomarker may be used to see how well the body responds to a treatment for a disease or condition.[1]

b. Pharmacogenomics: The investigation of variations of DNA and RNA characteristics as related to drug/vaccine response.2

c. Pharmacogenetics: A subset of pharmacogenomics, pharmacogenetics is the influence of variations in DNA sequence on drug/vaccine response.2

d. DNA: Deoxyribonucleic acid.

e. RNA: Ribonucleic acid.

2. Scope of Future Biomedical Research

The left over DNA and plasma specimen(s) collected in the current trial will be used to study various causes for how subjects may respond to a drug/vaccine. The left over DNA and plasma specimen(s) will be stored to provide a resource for future trials conducted by Merck focused on the study of biomarkers responsible for how a drug/vaccine enters and is removed by the body, how a drug/vaccine works, other pathways a drug/vaccine may interact with, or other aspects of disease. The specimen(s) may be used for future assay development and/or drug/vaccine development.

It is now well recognized that information obtained from studying and testing clinical specimens offers unique opportunities to enhance our understanding of how individuals respond to drugs/vaccines, enhance our understanding of human disease and ultimately improve public health through development of novel treatments targeted to populations with the greatest need. All specimens will be used by Merck or designees and research will be monitored and reviewed by a committee of our scientists and clinicians.

3. Summary of Procedures for Future Biomedical Research

a. Subjects for Enrollment

All subjects enrolled in the clinical trial will be considered for enrollment in the Future Biomedical Research sub-trial.

b. Informed Consent

Informed consent for specimens (i.e., DNA, RNA, protein, etc.) will be obtained during screening for protocol enrollment from all subjects or legal guardians, at a trial visit by the investigator or his or her designate. Informed consent for Future Biomedical Research should be presented to the subjects on Visit 1. If delayed, present consent at next possible Subject Visit. Informed consent must be obtained prior to collection of all Future Biomedical Research specimens. Consent forms signed by the subject will be kept at the clinical trial site under secure storage for regulatory reasons. Information contained on the consent form alone cannot be traced to any specimens, test results, or medical information once the specimens have been rendered de-identified.

Subjects are not required to participate in the Future Biomedical Research sub-trial in order to participate in the main trial. Subjects who decline to sign the Future Biomedical Research informed consent will not have the specimen collected nor will they be discontinued from the main trial.

A template of each trial site's approved informed consent will be stored in the Sponsor's clinical document repository. Each consent will be assessed for appropriate specimen permissions.

Each informed consent approved by an ethics committee is assigned a unique tracking number. The tracking number on this document will be used to assign specimen permissions for each specimen into the Entrusted Keyholder's Specimen Database.

c. eCRF Documentation for Future Biomedical Research Specimens

Documentation of both consent and acquisition of Future Biomedical Research specimens will be captured in the electronic Case Report Forms (eCRFs). Reconciliation of both forms will be performed to assure that only appropriately-consented specimens are used for this sub-trial's research purposes. Any specimens for which such an informed consent cannot be verified will be destroyed.

d. Future Biomedical Research Specimen Collections

Blood specimens for DNA or RNA isolation will usually be obtained at a time when the subject is having blood drawn for other trial purposes. Specimens like tissue and bone marrow will usually be obtained at a time when the subject is having such a procedure for clinical purposes.

Specimens will be collected and sent to the laboratory designated for the trial where they will be processed (e.g., DNA or RNA extraction, etc) following the Merck approved policies and procedures for specimen handling and preparation.

If specimens are collected for a specific genotype or expression analysis as an objective to the main trial, this analysis is detailed in the main body of this protocol (**Section 8.0 – Statistical Analysis Plan**). These specimens will be processed, analyzed, and the remainder of the specimen will be destroyed. The results of these analyses will be reported along with the other trial results. A separate specimen will be obtained from properly-consented subjects in this protocol for storage in the biorepository for Future Biomedical Research.

4. Confidential Subject Information for Future Biomedical Research

In order to optimize the research that can be conducted with Future Biomedical Research specimens, it is critical to link subject' clinical information with future test results. In fact little or no research can be conducted without connecting the clinical trial data to the specimen. The clinical data allow specific analyses to be conducted. Knowing subject characteristics like gender, age, medical history and treatment outcomes are critical to understanding clinical context of analytical results.

To maintain privacy of information collected from specimens obtained for Future Biomedical Research, Merck has developed secure policies and procedures. All specimens will be de-identified as described below.

At the clinical trial site, unique codes will be placed on the Future Biomedical Research specimens for transfer to the storage facility. This first code is a random number which does not contain any personally identifying information embedded within it. The link (or key) between subject identifiers and this first unique code will be held at the trial site. No personal identifiers will appear on the specimen tube.

This first code will be replaced with a second code at a Merck designated storage/lab facility. The second code is linked to the first code via a second key. The specimen is now double coded. Specimens with the second code are sometimes referred to as de-identified specimens. The use of the second code provides additional confidentiality and privacy protection for subjects over the use of a single code. Access to both keys would be needed to link any data or specimens back to the subject's identification.

The second code is stored separately from the first code and all associated personal specimen identifiers. A secure link, the second key, will be utilized to match the second code to the first code to allow clinical information collected during the course of the trial to be associated with the specimen. This second key will be transferred under secure procedures by the Merck designated facility to an Entrusted Keyholder at Merck. The second code will be logged into the primary biorepository database at Merck and, in this database, this identifier will not have identifying demographic data or identifying clinical information (i.e., race, sex, age, diagnosis, lab values) associated with it. The specimen will be stored in a designated biorepository site with secure policies and procedures for specimen storage and usage.

The second key can be utilized to reconstruct the link between the results of future biomedical research and the clinical information, at the time of analysis. This linkage would not be possible for the scientist conducting the analysis, but can only be done by the Merck Entrusted Keyholder under strict security policies and procedures. The Merck Entrusted Keyholder will link the information and then issue a de-identified data set for analysis. The only other circumstance by which future biomedical research data would be directly linked to the full clinical data set would be those situations mandated by regulatory authorities (e.g., EMEA, FDA), whereby this information would be directly transferred to the regulatory authority.

5. Biorepository Specimen Usage

Specimens obtained for the Merck Biorepository will be used for analyses using good scientific practices. However, exploratory analyses will not be conducted under the highly validated conditions usually associated with regulatory approval of diagnostics. The scope of research performed on these specimens is limited to the investigation of the variability in biomarkers that may correlate with a clinical phenotype in subjects.

Analyses utilizing the Future Biomedical Research specimens may be performed by Merck, or an additional third party (e.g., a university investigator) designated by Merck. The investigator conducting the analysis will be provided with double coded specimens. Re-association of analysis results with corresponding clinical data will only be conducted by the Merck Entrusted Keyholder. Any contracted third party analyses will conform to the specific scope of analysis outlined in this sub-trial. Future Biomedical Research specimens remaining with the third party after the specific analysis is performed will be

returned to the sponsor or destroyed and documentation of destruction will be reported to Merck.

6. Withdrawal From Future Biomedical Research

Subjects may withdraw their consent for Future Biomedical Research and have their specimens and all derivatives destroyed. Subjects may withdraw consent at any time by contacting the principal investigator for the main trial. If medical records for the main trial are still available, the investigator will contact Merck using the designated mailbox PPD and a form will be provided by Merck to obtain appropriate information to complete specimen withdrawal. Subsequently, the subject's specimens will be removed from the biorepository and be destroyed. A letter will be sent from Merck to the investigator confirming the destruction. It is the responsibility of the investigator to inform the subject of completion of destruction. Any analyses in progress at the time of request for destruction or already performed prior to the request being received by the Sponsor will continue to be used as part of the overall research trial data and results. No new analyses would be generated after the request is received.

In the event that the medical records for the main trial are no longer available (e.g., if the investigator is no longer required by regulatory authorities to retain the main trial records) or the specimens have been completely anonymized, there will no longer be a link between the subject's personal information and their specimens. In this situation, the request for specimen destruction can not be processed.

7. Retention of Specimens

Future Biomedical Research specimens will be stored in the biorepository for potential analysis for up to 20 years from acquisition. Specimens may be stored for longer if a regulatory or governmental authority has active questions that are being answered. In this special circumstance, specimens will be stored until these questions have been adequately addressed.

Specimens from the trial site will be shipped to a central laboratory and then shipped to the Merck designated biorepository. The specimens will be stored under strict supervision in a limited access facility which operates to assure the integrity of the specimens. Specimens will be destroyed according to Merck policies and procedures and this destruction will be documented in the biorepository database.

8. Data Security

Separate databases for specimen information and for results from the Future Biomedical Research sub-trial will be maintained by Merck. This is done to separate the future exploratory test results (which include genetic data) from the clinical trial database thereby maintaining a separation of subject number and these results. The separate databases are accessible only to the authorized Sponsor and the designated trial administrator research personnel and/or collaborators. Database user authentication is highly secure, and is accomplished using network security policies and practices based in international standards (e.g., ISO17799) to protect against unauthorized access. The Merck Entrusted Keyholder maintains control over access to all specimen data. These

data are collected for future biomedical research purposes only as specified in this sub-trial will not be used for any other purpose.

9. Reporting of Future Biomedical Research Data to Subjects

There is no definitive requirement in either authoritative ethical guidelines or in relevant laws/regulations globally that research results have to be, in all circumstances, returned to the trial participant. Some guidelines advocate a proactive return of data in certain instances. No information obtained from exploratory laboratory studies will be reported to the subject or family, and this information will not be entered into the clinical database maintained by Merck on subjects. Principle reasons not to inform or return results to the subject include: lack of relevance to subject health, limitations of predictive capability, concerns of misinterpretation and absence of good clinical practice standards in exploratory research typically used for diagnostic testing.

If any exploratory results are definitively associated with clinical significance for subjects while the clinical trial is still ongoing, investigators will be contacted with information as to how to offer clinical diagnostic testing (paid for by Merck) to subjects enrolled and will be advised that counseling should be made available for all who choose to participate in this diagnostic testing.

If any exploratory results are definitively associated with clinical significance after completion of a clinical trial, Merck will publish the results without revealing specific subject information, inform all trial sites who participated in the Merck clinical trial and post anonymized results on our website or other accredited website(s) that allow for public access (e.g., disease societies who have primary interest in the results) in order that physicians and patients may pursue clinical diagnostic testing if they wish to do so.

10. Gender, Ethnicity and Minorities

Although many diagnoses differ in terms of frequency by ethnic population and gender, every effort will be made to recruit all subjects diagnosed and treated on Merck clinical trials for future biomedical research. When trials with specimens are conducted and subjects identified to serve as controls, every effort will be made to group specimens from subjects and controls to represent the ethnic and gender population representative of the disease under current investigation.

11. Risks Versus Benefits of Future Biomedical Research

For future biomedical research, risks to the subject have been minimized. Risks include those associated with venipuncture to obtain the whole blood specimen. This specimen will be obtained at the time of routine blood specimens drawn in the main trial.

Merck has developed strict security, policies and procedures to address subject data privacy concerns. Data privacy risks are largely limited to rare situations involving possible breach of confidentiality. In this highly unlikely situation there is risk that the information, like all medical information, may be misused.

It is necessary for subject-related data (i.e., ethnicity, diagnosis, drug therapy and dosage, age, toxicities, etc.) to be re-associated to double coded specimens at the time of data analysis. These subject data will be kept in a separate, secure Merck database, and all specimens will be stripped of subject identifiers. No information concerning results

obtained from future biomedical research will be entered into clinical records, nor will it be released to outside persons or agencies, in any way that could be tied to an individual subject.

12. Self-Reported Ethnicity

Subjects who participate in future biomedical research will be asked to provide self-reported ethnicity. Subjects who do not wish to provide this data may still participate in future biomedical research.

13. Questions

Any questions related to the future biomedical research should be e-mailed directly to PPD

14. References

1. National Cancer Institute: http://www.cancer.gov/dictionary/?searchTxt=biomarker

2. International Conference on Harmonization: DEFINITIONS FOR GENOMIC BIOMARKERS, PHARMACOGENOMICS, PHARMACOGENETICS, GENOMIC DATA AND SAMPLE CODING CATEGORIES - E15; http://www.ich.org/LOB/media/MEDIA3383.pdf

12.3 Understanding the Intent, Scope and Public Health Benefits of Exploratory Biomarker Research: A Guide for IRBs/IECs and Investigational Site Staff

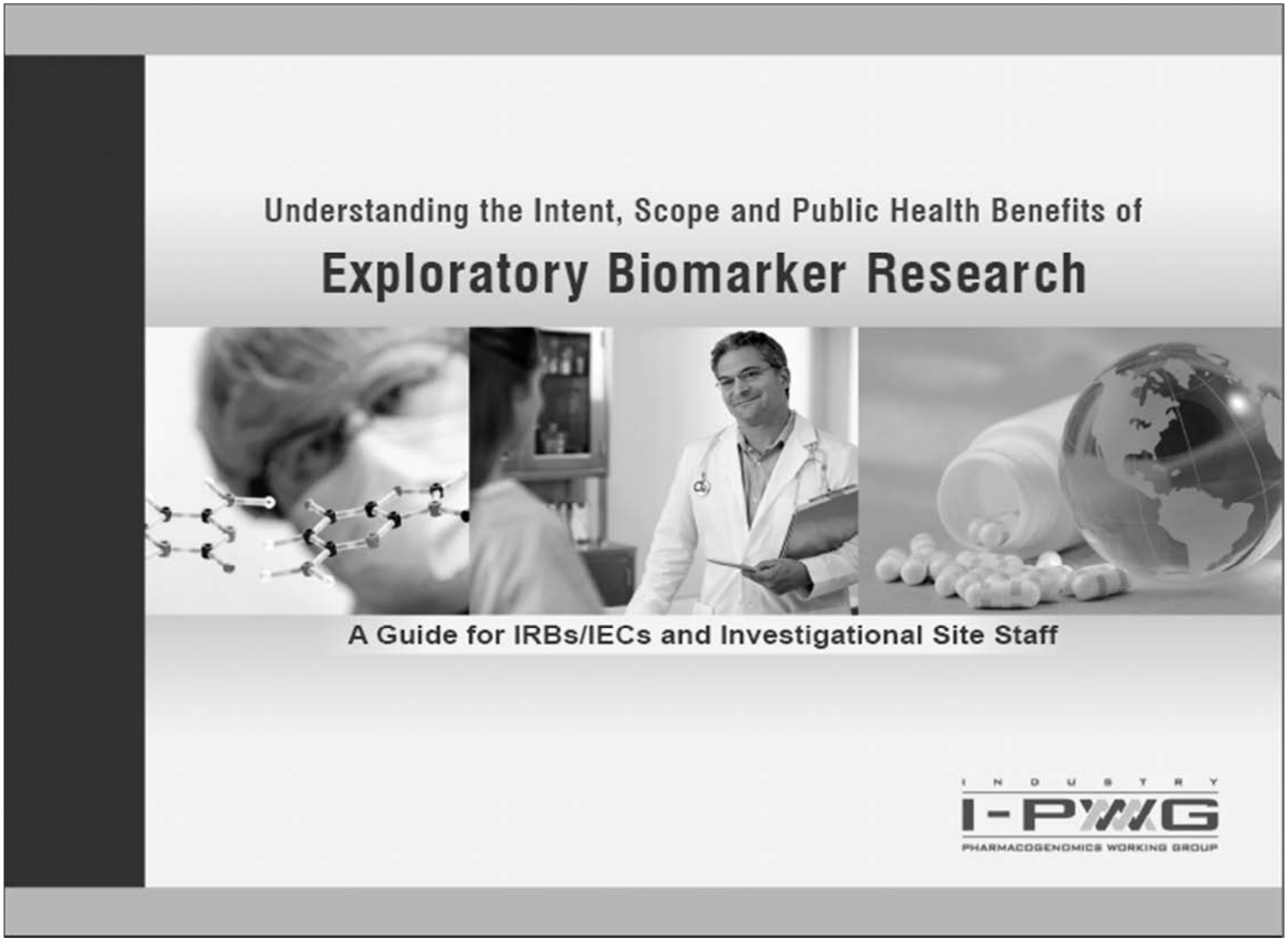

This informational brochure is intended for IRBs/IECs and Investigational Site Staff. The brochure addresses issues relevant to specimen collection for biomarker research in the context of pharmaceutical drug and vaccine development.

Developed by
The Industry Pharmacogenomics Working Group (I-PWG)
www.i-pwg.org

1. What is a Biomarker and What is Biomarker Research?

A biomarker is a *"characteristic that is objectively measured and evaluated as an indicator of normal biological processes, pathogenic processes, or pharmacologic responses to a therapeutic intervention"*. [1]

Biomarker research, including research on pharmacogenomic biomarkers, is a tool used to improve the development of pharmaceuticals and understanding of disease. It involves the analysis of biomolecules (such as DNA, RNA, proteins, and lipids), or other measurements (such as blood pressure or brain images) in relation to clinical endpoints of interest. Biomarker research can be influential across all phases of drug development, from drug discovery and preclinical evaluations to clinical development and post-marketing studies. This brochure focuses on biomarker research involving analysis of biomolecules from biological samples collected in clinical trials. Please refer to I-PWG Pharmacogenomic Informational Brochure[2] and ICH Guidance E15[3] for additional information specific to pharmacogenomic biomarkers.

2. Why is Biomarker Research Important?

Importance to Patients and Public Health

Biomarker research is helping to improve our ability to predict, detect, and monitor diseases and improve our understanding of how individuals respond to drugs. This research underlies personalized medicine: a tailored approach to patient treatment based on the molecular analysis of genes, proteins, and metabolites.[4] The goal of biomarker research is to aid clinical decision-making toward safer and more efficacious courses of treatment, improved patient outcomes, and overall cost-savings. It also allows for the continued development and availability of drugs that are effective in certain sub-populations when they otherwise might not have been developed due to insufficient efficacy in the broader population.

Recent advances in biomedical technology, including genetic and molecular medicine, have greatly increased the power and precision of analytical tools used in health research and have accelerated the drive toward personalized medicine. In some countries, highly focused initiatives have been created to promote biomarker research (e.g., in the US: www.fda.gov/oc/initiatives/criticalpath/ ; in the EU: www.imi.europa.eu/index_en.html).

Importance to Drug Development

Biomarker research is being used by the pharmaceutical industry to streamline the drug development process. Some biomarkers are used as substitutes or "surrogates" for safety or efficacy endpoints in clinical trials particularly where clinical outcomes or events cannot practically or ethically be measured (e.g., cholesterol as a surrogate for cardiovascular disease).[5] By using biomarkers to assess patient response, ineffective drug candidates may be terminated earlier in the development process in favor of more promising drug candidates. Biomarkers are being used to optimize clinical trial designs and outcomes by identifying patient populations that are more likely to respond to a drug therapy or to avoid specific adverse events.

1

I-PWG

Biomarker research is also being used to enhance scientific understanding of the mechanisms of both treatment response and disease processes, which can help to identify future targets for drug development. Depending on the clinical endpoints in a clinical trial, biomarker sample collection may either be a required or optional component of the trial. However, both mandatory and optional sample collections are important for drug development.

3. Importance of Biomarkers to Regulatory Authorities

Regulatory health authorities are increasingly aware of the benefits of biomarkers and how they may be used for drug approval, clinical trial design, and clinical care. Biomarkers have been used to establish risk:benefit profiles. For example, the FDA has modified the US warfarin (Coumadin®) label to include the analysis of *CYP2C9* and *VKORC1* genes to guide dosing regimens. Health authorities such as the FDA (USA), EMEA (European Union), MHLW (Japan), and ICH (International) are playing a key role in advancing this scientific field as it applies to pharmaceutical development by creating the regulatory infrastructure to facilitate this research. Numerous regulatory guidances and concept papers have already been issued, many of which are available through www.i-pwg.org. Global regulatory authorities have highlighted the importance of biomarker research and the need for the pharmaceutical industry to take the lead in this arena.[3, 6-24]

4. How are Biomarkers Being Used in Drug/Vaccine Development?

Biomarker research is currently being used in drug/vaccine development to:

- Explain variability in response among participants in clinical trials
- Better understand the mechanism of action or metabolism of investigational drugs
- Obtain evidence of pharmacodynamic activity (i.e., how the drug affects the body) at the molecular level
- Address emerging clinical issues such as unexpected adverse events
- Determine eligibility for clinical trials to optimize trial design
- Optimize dosing regimens to minimize adverse reactions and maximize efficacy
- Develop drug-linked diagnostic tests to identify patients who are more likely or less likely to benefit from treatment or who may be at risk of experiencing adverse events
- Provide better understanding of mechanisms of disease
- Monitor clinical trial participant response to medical interventions

> Biomarker research, including research on banked samples, should be recognized as an important public health endeavor for the overall benefit of society, whether by means of advancement of medical science or by development of safer and more effective therapies.[7] Since the value of collected samples may increase over time as scientific discoveries are made, investment in long-term sample repositories is a key component of biomarker research.

I-PWG

5. Biomarkers are Already a Reality in Health Care

A number of drugs now have biomarker information included in their labels.[25] Biomarker tests are already being used in clinical practice to serve various purposes:

Predictive biomarkers (efficacy) – In clinical practice, predictive efficacy biomarkers are used to predict which patients are most likely to respond, or not respond, to a particular drug. Examples include: i) *Her2/neu* overexpression analysis required for prescribing trastuzumab (Herceptin®) to breast cancer patients, ii) *c-kit* expression analysis prior to prescribing imatinib mesylate (Gleevec®) to gastrointestinal stromal tumor patients, and iii) *KRAS* mutational status testing prior to prescribing panitumumab (Vectibix®) or cetuximab (Erbitux®) to metastatic colorectal cancer patients.

Predictive biomarkers (safety) – In clinical practice, predictive safety biomarkers are used to select the proper drug dose or to evaluate the appropriateness of continued therapy in the event of a safety concern. Examples include: i) monitoring of blood potassium levels in patients receiving drospirenone and ethinyl estradiol (Yasmin®) together with daily long-term drug regimens that may increase serum potassium, and ii) prospective *HLA-B*5701* screening to identify those at increased risk for hypersensitivity to abacavir (Ziagen®).

Surrogate biomarkers – In clinical practice, surrogate biomarkers may be used as alternatives to measures such as survival or irreversible morbidity. Surrogate biomarkers are measures that are reasonably likely, based on epidemiologic, therapeutic, pathophysiologic, or other evidence, to predict clinical benefit. Examples include: i) LDL level as a surrogate for risk of cardiovascular diseases in patients taking lipid-lowering agents such as atorvastatin calcium (Lipitor®), ii) blood glucose as a surrogate for clinical outcomes in patients taking anti-diabetic agents, and iii) HIV plasma viral load and CD4 cell counts as surrogates for time-to-clinical-events and overall survival in patients receiving antiretroviral therapy for HIV disease.

Prognostic biomarkers – Biomarkers can also help predict clinical outcomes independent of any treatment modality. Examples of prognostic biomarkers used in clinical practice include: i) CellSearch™ to predict progression-free survival in breast cancer, ii) anti-CCP (cyclic citrullinated protein) for the severity of rheumatoid arthritis, iii) estrogen receptor status for breast cancer, and iv) anti-dsDNA for the severity of systemic lupus erythematosus.

6. Biomarker Samples from Clinical Trials: An Invaluable Resource

Adequate sample sizes and high-quality data from controlled clinical trials are key to advancements in biomarker research. Samples collected in clinical trials create the opportunity for investigation of biomarkers related to specific drugs, drug classes, and disease areas. Clinical drug development programs are therefore an invaluable resource and a unique opportunity for highly productive biomarker research. In addition to conducting independent research, pharmaceutical companies are increasingly contributing to consortia efforts by pooling samples, data, and expertise in an effort to conduct rigorous and efficient biomarker research and to maximize the probability of success.[26-27]

7. Informed Consent for Collection & Banking of Biomarker Samples

Collection of biological samples in clinical trials must be undertaken with voluntary informed consent of the participant (or legally-acceptable representative). Policies

and regulations for legally-appropriate informed consent vary on national, state, and local levels, but are generally based on internationally recognized pillars of ethical conduct for research on human subjects.[28-31]

Optional vs. Required Subject Participation

Depending on the relevance of biomarker research to a clinical development program at the time of protocol development, the biomarker research may be a core required component of a trial (e.g., key to elucidating the drug mechanism of action or confirming that the drug is interacting with the target) or may be optional (e.g., to gain valuable knowledge that enhances the understanding of diseases and drugs). Informed consent for the collection of biomarker samples may be presented either in the main clinical informed consent form or as a separate informed consent form, with approaches varying somewhat across pharmaceutical companies. The relevance of biomarker research to a clinical development program may change over time as the science evolves. The samples may therefore increase in value after a protocol is developed.

Consent for Future Research Use

While it can be a challenge to specify the details of the research that will be conducted in the future, the I-PWG holds the view that future use of samples collected for exploratory biomarker research in clinical trials should be permissible when i) the research is scientifically sound, ii) participants are informed of the scope of the intended future research, even if this is broadly defined (see potential uses in Section 4 above), iii) autonomy is respected by providing the option to consent separately to future use of samples or by providing the option to terminate further use of samples upon request (consent withdrawal / sample destruction), and iv) industry standards for confidentiality protection per Good Clinical Practice guidelines are met.[3, 31] Importantly, any research using banked samples should be consistent with the original informed consent, except where otherwise permitted by local law or regulation.

Important elements of informed consent for **future use** of samples include, but are not limited to:[39]

The scope of research – Where the scope of the potential future research is broad, participants should be informed of the boundaries of the research. While it may not be possible to describe the exact analytical techniques that will be used, or specific molecules that will be analyzed, it is possible to clearly articulate in reasonable detail the type of research to be conducted and its purpose. Information regarding whether stored samples may be shared with other parties or utilized for commercialization purposes should also be addressed.

Withdrawal of consent / sample destruction – The informed consent form should inform participants of their right to withdraw their consent / request destruction of their samples. This should include the mechanisms for exercising that right and any limitations to exercising that right. For example, participants should be informed that it is not possible to destroy samples that have been anonymized.[3] In addition, according to industry standards and regulatory guidance, participants should be informed that data already generated prior to a consent withdrawal request are to be maintained as part of the study data.[38]

The duration of storage – The permissible duration of storage may vary according to the nature and uses of the samples and may also vary on national, state, and local levels. The intended duration of storage, including indefinite storage, should be specified.

I-PWG

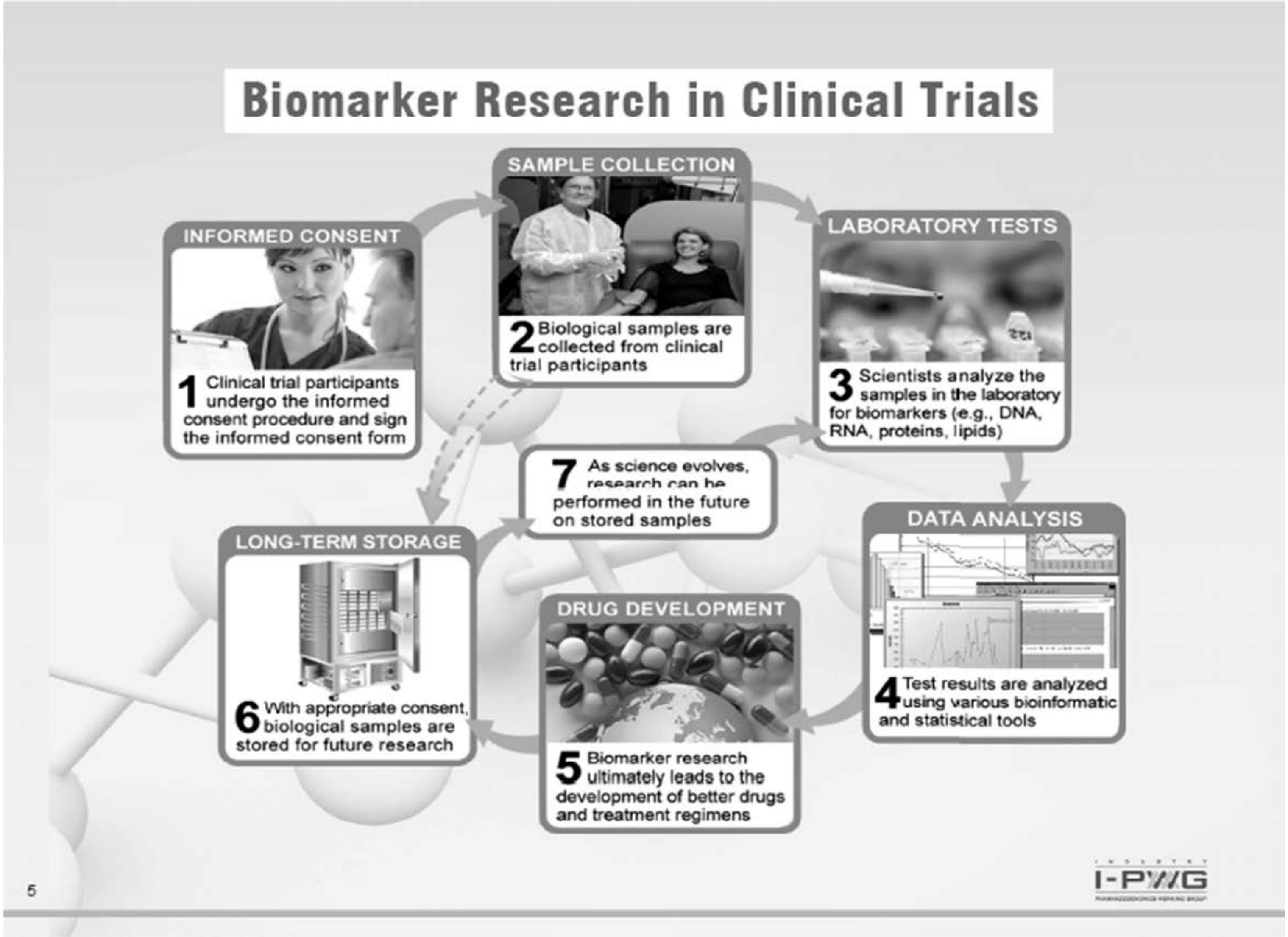
Biomarker Research in Clinical Trials
INFORMED CONSENT
1 Clinical trial participants undergo the informed consent procedure and sign the informed consent form
SAMPLE COLLECTION
2 Biological samples are collected from clinical trial participants
LABORATORY TESTS
3 Scientists analyze the samples in the laboratory for biomarkers (e.g., DNA, RNA, proteins, lipids)
DATA ANALYSIS
4 Test results are analyzed using various bioinformatic and statistical tools
DRUG DEVELOPMENT
5 Biomarker research ultimately leads to the development of better drugs and treatment regimens
LONG-TERM STORAGE
6 With appropriate consent, biological samples are stored for future research
7 As science evolves, research can be performed in the future on stored samples
5
I-PWG

8. Biomarker Sample Collection in Different Countries

Collection of biological samples for biomarker research is straightforward in most jurisdictions. Some countries have specific laws and regulations regarding collection, labeling, storage, export, and/or use of exploratory samples. In addition, some regulations distinguish between DNA and non-DNA samples or between samples used for diagnostic purposes and samples collected for scientific research. Processes for the collection, labeling, storage, export, and/or use of biomarker samples should always adhere to the laws and regulations of the country/region in which those samples are collected.

9. Return of Research Results to Study Participants

Policies for the return of biomarker research results to study participants who request them vary among pharmaceutical companies. There are many considerations that pharmaceutical companies weigh when determining their policy regarding the return of biomarker research results to study participants. These include:

i) the conditions under which biomarker research results were generated (i.e., exploratory research laboratory versus accredited diagnostic laboratory)

ii) whether the results will have an impact on the medical care of the participant or on a related person, if applicable

iii) whether genetic counseling is recommended (for genetic results)

iv) the ability to accurately link the result to the individual from whom the sample was collected

v) international, national, and local guidelines, policies, legislation, and regulations regarding participants' rights to access data generated on them

Renegar *et al*. 2006 and Article 29 Data Protection Working Party (an advisory committee to the European Commission on the European Data Protection Directive) have addressed these considerations in detail in relation to pharmacogenomic research data and provided a list of documents addressing the general issue of return of research results.[34-35]

10. Benefits and Risks Associated with Biomarker Research

Benefits
While it may not always directly benefit the study participant who is providing the samples, biomarker research can improve overall understanding of disease and treatment of future patients receiving therapies developed from such research. Patients are now benefiting from retrospective biomarker research conducted on samples collected from clinical trials and stored for exploratory research. One example is the recent label update to the EGFR antibody drugs cetuximab (Erbitux®) and panitumumab (Vectibix®) which highlights the value of *KRAS* status as a predictive biomarker for treatment of metastatic colorectal cancer with this class of drug.

The humanitarian benefit of human research is recognized by the Nuremberg Code.[28,33] Provided that the degree of risk does not exceed that determined by the humanitarian importance of the problem to be solved, research participants should not be denied the right to contribute to the greater common good.[28,32]

Risks
Risks associated with biomarker research are primarily related to the physical aspects of obtaining the sample and to patient privacy concerns.

Physical risks associated with biomarker sample collection in clinical trials can be characterized in two ways: i) negligible additional risk when the biomarker sample is collected as part of a procedure conducted to support

other core trial objectives, and ii) some added risk where the sampling procedure would otherwise have not been performed as a core component of a trial. Risks are also determined by the invasiveness of the sample collection procedure.

Privacy risks are generally those associated with the inappropriate disclosure and misuse of data. Pharmaceutical companies have policies and procedures for confidentiality protection to minimize this risk for all data collected and generated in clinical trials. These may vary across companies, but are based on industry standards of confidentiality and privacy protection highlighted in the following section. Importantly, privacy risks inherent to biomarker data are no greater than other data collected in a clinical trial.

11. Privacy, Confidentiality, and Patient Rights

Maintaining the privacy of study participants and the confidentiality of information relating to them is of paramount concern to industry researchers, regulators, and patients. Good Clinical Practice (GCP), the standard adhered to in pharmaceutical clinical research, is a standard that

"...provides assurance that the data and reported results are credible and accurate, and that the rights, integrity, and confidentiality of trial subjects are protected",

where confidentiality is defined as, *"The prevention of disclosure, to other than authorized individuals, of a sponsor's proprietary information or of a subject's identity."*

This standard dictates that *"the confidentiality of records that could identify subjects should be protected, respecting the privacy and confidentiality rules in accordance with applicable regulatory requirements."* [31]

Exploratory biomarker research in pharmaceutical development is commonly conducted in research laboratories that are not accredited to perform diagnostic tests used for healthcare decision-making. Therefore, results from exploratory biomarker research usually are not appropriate for use in making decisions about a trial participant's health. In addition, exploratory research data should not be included as part of a participant's medical record accessible for use by insurance companies. Legislation and policies to protect individuals against discrimination based on genetic information continually evolve based on social, ethical, and legal considerations. Examples of such legislation include the Human Tissue Act 2004 (UK) and the Genetic Information Nondiscrimination Act (GINA) 2008 (USA).[36-37]

12.Where to Get More Information?

Educational resources related to biomarker and pharmacogenomic research that caters to health care professionals, IRBs/IECs, scientists, and patients are continually being created and are publicly available. Links to many of these resources are available through the I-PWG website: www.i-pwg.org.

13. What is I-PWG?

The Industry Pharmacogenomics Working Group (I-PWG) (formerly the Pharmacogenetics Working Group) is a voluntary association of pharmaceutical companies engaged in pharmacogenomic research. The Group's activities focus on non-competitive educational, informational, ethical, legal, and regulatory topics. The Group provides information and expert opinions on these topics and sponsors educational/informational programs to promote better understanding of pharmacogenomic and other biomarker research for key stakeholders. The I-PWG interacts with regulatory author-

ities and policy groups to ensure alignment. More information about the I-PWG is available at: www.i-pwg.org.

14. Contributing authors

Monique A. Franc, Teresa Hesley, Feng Hong, Ronenn Roubenoff, Jasjit Sarang, Andrea Tyukody Renninger, Amelia Warner

15. References

1. Atkinson AJ, Colburn WA, DeGruttola VG, et al. Biomarkers and surrogate endpoints: Preferred definitions and conceptual framework. Clinical Pharmacology & Therapeutics 2001; 69(3): 89-95. (Accessed at: www.ncbi.nlm.nih.gov/pubmed/11240971)
2. I - PWG Pharmacogenomics Informational Brochure, 2008. (Accessed at: http//:www.i-pwg.org/cms/index.php?option=com_docman&task=doc_download&gid=77&itemid=118)
3. ICH E15 – Definitions for Genomic Biomarkers, Pharmacogenomics, Pharmacogenetics, Genomic Data and Sample Coding Categories. April 2008. (Accessed at: www.fda.gov/OHRMS/DOCKETS/98fr/FDA-2008-D-0199-gdl.pdf and at: http://www.ich.org/LOB/media/MEDIA3383.pdf)
4. Davis JC, Furstenthal L, Desai AA, et al. The microeconomics of personalized medicine: today's challenge and tomorrow's promise. Nature Reviews Drug Discovery, 2009; 8: 279. (Accessed at:http: www.nature.com/nrd/journal/v8/n4/abs/nrd2825.html)
5. Berns B, Démolis P, Scheulen ME. How can biomarkers become surrogate endpoints? European Journal of Cancer Supplements 2007; 5: 37-40. (Accessed at: www.journals.elsevierhealth.com/periodicals/ejcsup/issues/contents?issue_key=S1359-6349%2807%29X0031-4)
6. Lesko LJ, Woodcock J. Translation of pharmacogenomics and pharmacogenetics: a regulatory perspective. Nature Reviews Drug Discovery, 2004; 3: 763-769. (Accessed at: www.nature.com/nrd/journal/v3/n9/abs/nrd1499.html)
7. Lesko LJ, Woodcock J. Pharmacogenomic-guided drug development: regulatory perspective. The Pharmacogenomics Journal, 2002; 2: 20-24. (Accessed at www.ncbi.nlm.nih.gov/pubmed/11990376)
8. Petricoin EF, Hackett JL, Lesko LJ, et al. Medical applications of microarray technologies: a regulatory science perspective. Nat Genet., 2002; 32: 474-479. (Accessed at: www.nature.com/ng/journal/v32/n4s/abs/ng1029.html)
9. Lesko LJ, Salerno RA, Spear BB, et al. Pharmacogenetics and pharmacogenomics in drug development and regulatory decision making: report of the first FDA-PWG-PhRMA-DruSafe Workshop. J Clin Pharmacol., 2003; 43: 342-358. (Accessed at: http://jcp.sagepub.com/cgi/content/abstract/43/4/342)
10. Salerno RA, Lesko LJ. Pharmacogenomics in Drug Development and Regulatory Decision-making: the Genomic Data Submission (GDS) Proposal. Pharmacogenomics, 2004; 5: 25-30. (Accessed at: www.futuremedicine.com/doi/pdf/10.2217/14622416.5.1.25)
11. Frueh FW, Goodsaid F, Rudman A, et al. The need for education in pharmacogenomics: a regulatory perspective. The Pharmacogenomics Journal, 2005; 5: 218-220. (Accessed at: www.nature.com/tpj/journal/v5/n4/abs/6500316a.html)
12. Genomic Biomarkers Related to Drug Response: Context, Structure and Format of Qualification Submissions. ICH E16 Step 3 draft. (Accessed at: www.emea.europa.eu/pdfs/human/ich/38063609endraft.pdf)
13. Guiding principles Processing Joint FDA EMEA Voluntary Genomic Data Submissions (VGDSs) within the framework of the Confidentiality Arrangement. May 19, 2006. (Accessed at: www.fda.gov/downloads/Drugs/ScienceResearch/ResearchAreas/Pharmacogenetics/ucm085378.pdf)
14. Guidance for Industry Pharmacogenomic Data Submissions. FDA. March 2005. (Accessed at: www.fda.gov/downloads/Drugs/GuidanceComplianceRegulatoryInformation/Guidances/ucm079849.pdf)
15. Pharmacogenomic Data Submissions - Companion Guidance. FDA Draft Guidance. August 2007. (Accessed at: www.fda.gov/downloads/Drugs/GuidanceComplianceRegulatoryInformation/Guidances/ucm079855.pdf)
16. Reflection Paper on Pharmacogenomics in Oncology. EMEA. 2008. (Accessed at: www.emea.europa.eu/pdfs/human/pharmacogenetics/12843506endraft.pdf)
17. Position paper on Terminology in Pharmacogenetics. EMEA. 2002. (Accessed at: www.emea.europa.eu/pdfs/human/press/pp/307001en.pdf)
18. Concept paper on the development of a Guideline on the use of pharmacogenomic methodologies in the pharmacokinetic evaluation of medicinal products. EMEA. 2009. (Accessed at: www.emea.europa.eu/pdfs/human/pharmacogenetics/6327009en.pdf)
19. Reflection paper on Pharmacogenomic samples, testing and data handling. EMEA. 2007. (Accessed at: www.emea.europa.eu/pdfs/human/pharmacogenetics/20191406en.pdf)
20. Ishiguro A, Toyoshima S, Uyama Y. Current Japanese regulatory situations of pharmacogenomics in drug administration. Expert Review of Clinical Pharmacology, 2008;1: 505-514. (Accessed at: www.ingentaconnect.com/content/ftd/ecp/2008/00000001/00000004/art00007)
21. Amur S, Frueh FW, Lesko LJ, et al. Integration and use of

biomarkers in drug development, regulation and clinical practice: A US regulatory practice. Biomarkers Med. 2008; 2, 305-311. (Accessed at: www.ingentaconnect.com/content/fm/bmm/2008/00000002/00000003/art00010?crawler=true)

22. Mendrick DL, Brazell C, Mansfield EA, et al. Pharmacogenomics and regulatory decision making: an international perspective. The Pharmacogenomics Journal. 2006; 6(3), 154-157. (Accessed at: www.nature.com/tpj/journal/v6/n3/abs/6500364a.html)

23. Pendergast MK. Regulatory agency consideration of pharmacogenomics. Exp Biol Med (Maywood). 2008; 233:1498-503. (Accessed at: www.ebmonline.org/cgi/content/abstract/233/12/1498)

24. Goodsaid F, Frueh F. Process map proposal for the validation of genomic biomarkers. Pharmacogenomics., 2006; 7(5):773-82 (Accessed at: www.futuremedicine.com/doi/abs/10.2217/14622416.7.5.773)

25. FDA Table of Valid Genomic Biomarkers in the Context of Approved Drug Labels. (Accessed at: www.fda.gov/Drugs/ScienceResearch/ResearchAreas/Pharmacogenetics/ucm083378.htm)

26. International Serious Adverse Event Consortium. (Accessed at: www.saeconsortium.org)

27. Predictive Safety Testing Consortium. (Accessed at: www.c-path.org/pstc.cfm)

28. Nuremberg code. (Accessed at: http://ohsr.od.nih.gov/guidelines/nuremberg.html)

29. Declaration of Helsinki. (Accessed at: http://ohsr.od.nih.gov/guidelines/helsinki.html)

30. Belmont report. (Accessed at: http://ohsr.od.nih.gov/guidelines/belmont.html)

31. ICH E6(R1) – Guideline for Good Clinical Practice. June 1996. (Accessed at: www.ich.org/LOB/media/MEDIA482.pdf)

32. Barnes M, Heffernan K. The "Future Uses" Dilemma: Secondary Uses of Data and Materials by Researchers for Commercial Research Sponsors. Medical Research Law & Policy, 2004; 3: 440-450.

33. Eriksson S, Helgesson G. Potential harms, anonymization, and the right to withdraw consent to biobank research. Eur J Hum Genet., 2005; 13:1071-1076. (Accessed at: www.nature.com/ejhg/journal/v13/n9/pdf/5201458a.pdf)

34. Renegar G, Webster CJ, Stuerzebecher S, et al. Returning genetic research results to individuals: points-to-consider. Bioethics 2006; 20: 24-36. (Accessed at: http://www3.interscience.wiley.com/cgi-bin/fulltext/118562753/PDFSTART)

35. Article 29 Data Protection Working Party. (Accessed at: www.ec.europa.eu/justice_home/fsj/privacy/workinggroup/index_en.htm)

36. Human Tissue Act 2004 (UK). (Accessed at: www.opsi.gov.uk/acts/acts2004/en/ukpgaen_20040030_en_1)

37. Genetic Information Nondiscrimination Act. (Accessed at: http://frwebgate.access.gpo.gov/cgi-bin/getdoc.cgi?dbname=110_cong_public_laws&docid=f:publ233.110.pdf)

38. Guidance for Sponsors, Clinical Investigators, and IRBs Data Retention When Subjects Withdraw from FDA-Regulated Clinical Trials. FDA October 2008 www.fda.gov/OHRMS/DOCKETS/98fr/FDA-2008-D-0576-gdl.pdf

39. Anderson C, Gomez-Mancilla B, Spear BB, Barnes DM, Cheeseman K, Shaw P, Friedman J, McCarthy A, Brazell C, Ray SC, McHale D, Hashimoto L, Sandbrink R, Watson ML, Salerno RA, on behalf of The Pharmacogenetics Working Group. Elements of Informed Consent for Pharmacogenetic Research; Perspective of the Pharmacogenetics Working Group. Pharmacogenomics Journal 2002;2:284-92. (Accessed at: www.nature.com/tpj/journal/v2/n5/abs/6500131a.html)

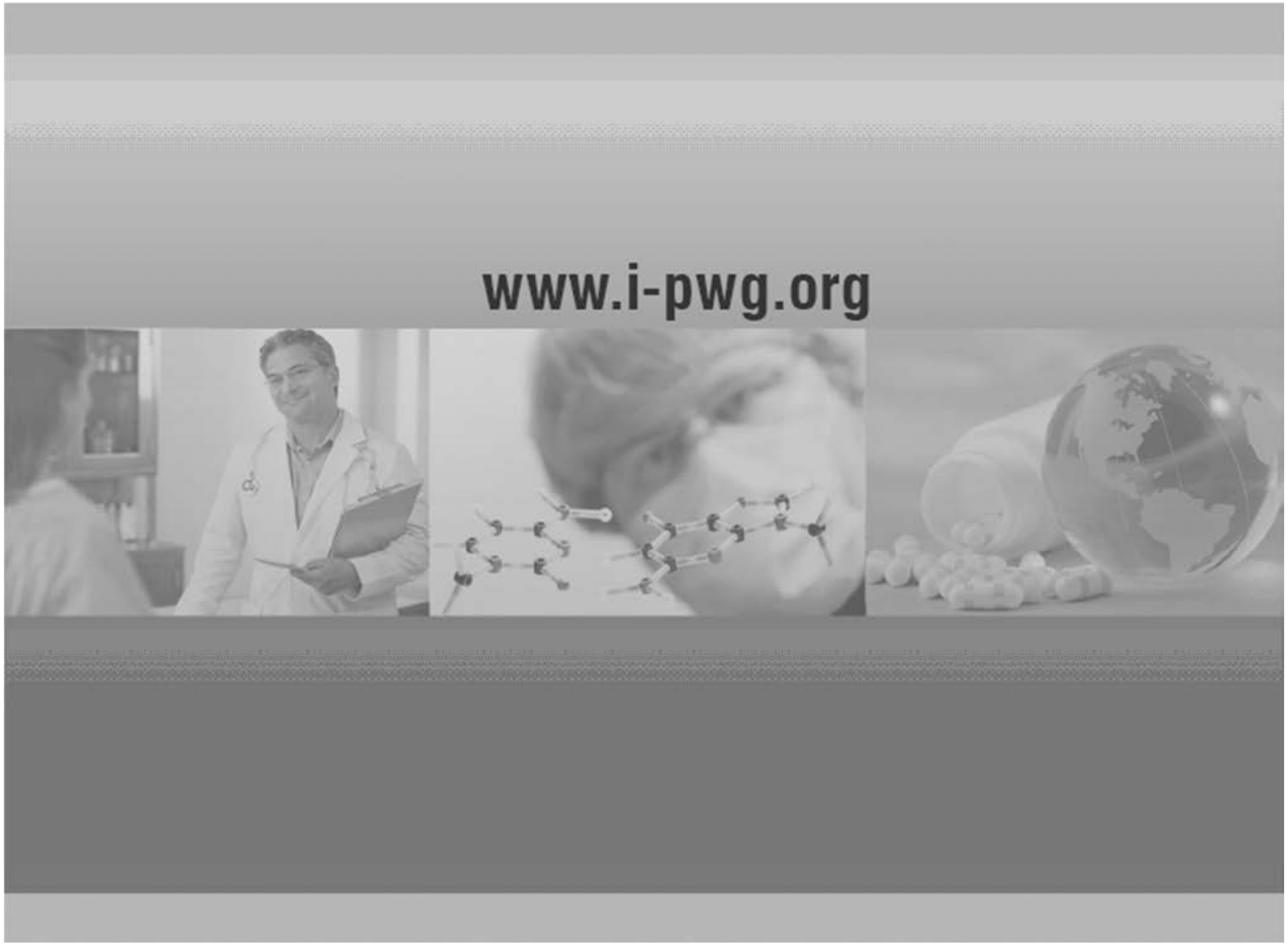
www.i-pwg.org

12.4 Definition of CMV Disease in Hematopoietic Stem Cell Transplant (HSCT) Recipients

CMV Disease	Diagnostic Criteria	Notes
Pneumonia	Signs and/or symptoms of pulmonary disease<br>**AND**<br>detection of CMV in BAL or tissue samples (virus isolation, histopathology, immunohistochemical analysis or *in situ* hybridization).	• PCR may be too sensitive, so detection of CMV by PCR alone is insufficient for the diagnosis of CMV pneumonia.<br>• Detection of fungal copathogens like *Aspergillus spp.* + "halo" sign (radiology) indicates fungal, rather than CMV pneumonia.<br>• Superinfection or coinfection with other pathogens may occur and should be noted when present.
Gastrointestinal (GI) Disease	Symptoms from upper or lower GI tract<br>**AND**<br>macroscopic endoscopic findings on endoscopy<br>**AND**<br>detection of CMV virus (isolation, histopathology, immunohistochemical analysis or *in situ* hybridization) in a GI biopsy specimen.	• Detection of CMV by PCR alone is insufficient for the diagnosis of CMV GI disease.
Hepatitis	Increased bilirubin and/or increased enzymes<br>**AND**<br>no other documented cause of hepatitis<br>**AND**<br>detection of CMV infection (culture, histopathology, immunohistochemical analysis or *in situ* hybridization) in a liver biopsy specimen.	• Detection of CMV by PCR alone is insufficient as it may represent transient viremia. Hence, PCR is insufficient to diagnose CMV hepatitis.<br>• Documentation of CMV in liver biopsy specimen (i.e., by culture, histopathology, immunohistochemical analysis or *in situ* hybridization) is needed.<br>• Coinfection with other pathogens like HCV may be present without excluding the diagnosis of CMV hepatitis.
Central Nervous System (CNS) disease	CNS symptoms<br>**AND**<br>detection of CMV in CSF samples (culture or PCR) OR in a brain biopsy specimen (culture, histopathology, immunohistochemical analysis or *in situ* hybridization).	N/A
Retinitis	Lesions typical of CMV retinitis must be confirmed by an ophthalmologist.	N/A
Nephritis	CMV infection (culture, immunohistochemical analysis or *in situ* hybridization)<br>**AND**<br>histologic features of CMV infection in a renal biopsy specimen in a patient with renal dysfunction.	• Detection of CMV by PCR alone is insufficient for the diagnosis of CMV nephritis.<br>• Furthermore, detection of CMV in urine of patient with renal dysfunction does not fulfill the definition of CMV nephritis.

CMV Disease	Diagnostic Criteria	Notes
Cystitis	CMV infection (culture, immunohistochemical analysis or *in situ* hybridization)<br>**AND**<br>histologic features of CMV infection in bladder biopsy specimen in a patient with cystitis.	• Detection of CMV by PCR alone is insufficient for the diagnosis of CMV cystitis.<br>• Furthermore, detection of CMV in urine of patient with symptoms does not fulfill the definition of CMV cystitis.
Myocarditis	CMV infection (culture, immunohistochemical analysis or *in situ* hybridization)<br>**AND**<br>histologic features of CMV infection in heart biopsy specimen in a patient with myocarditis.	• Detection of CMV by PCR alone is insufficient for diagnosis of CMV myocarditis.
Pancreatitis	CMV infection (culture, immunohistochemical analysis or *in situ* hybridization)<br>**AND**<br>histologic features of CMV infection in pancreatitis biopsy specimen in a patient with pancreatitis.	• Detection of CMV by PCR alone is insufficient for the diagnosis of CMV pancreatitis.
Other disease categories	Compatible signs and symptoms<br>**AND**<br>documentation of CMV by biopsy.	• Detection of CMV by PCR alone is insufficient.
Reference [1]<br>BAL = bronchoalveolar lavage; CMV = cytomegalovirus; CNS = central nervous system; CSF = cerebrospinal fluid; GI = gastrointestinal; HCV = hepatitis C virus; PCR = polymerase chain reaction.		

12.5 Child-Pugh Classification for Severity of Liver Disease

	**Scoring by Anomaly**		
**Signs or symptom**	**1 point**	**2 points**	**3 points**
Hepatic encephalopathy[1]	absent	Grade 1 or Grade 2	Grade 3 or Grade 4
Ascites	absent	mild	moderate
Bilirubin (μmol/L)	< 2 mg/dL	2 – 3 mg/dL	> 3 mg/dL
Albumin (g/dL)	> 3.5 g/dL	2.8 – 3.5 g/dL	< 2.8 g/dL
Prothrombin time (INR)	< 1.7	1.7 – 2.3	> 2.3

[1]Hepatic encephalopathy grading:
Grade 1: Altered mood/confusion
Grade 2: Inappropriate behavior, impending stupor, somnolence
Grade 3: Markedly confused, stuporous but arousable
Grade 4: Comatose/unresponsive

**Child Pugh Score Interpretation**	
5 – 6 points	Child-Pugh stage A (mild hepatic insufficiency)
7 – 9 points	Child-Pugh stage B (moderate hepatic insufficiency*)
>10 points	Child-Pugh stage C (severe hepatic insufficiency)

*If hypoalbuminemia is the only abnormality noted, the subject will need to have a score of ≥7 to qualify for moderate hepatic insufficiency for this study.

12.6 Approximate Blood/Tissue Volumes Drawn/Collected by Trial Visit and by Sample Types

Trial Visit:	SCR	D1	D7	W2	W3	W4	W5	W6	W7	W8	W9	W10[a]	W11[a]	W12[a]	W13[a]	End of Study Therapy W14[a]	W16	W18, W20, W22	W24	W32, W40, W48	CMV Infection or Early Discon Visit
Blood Parameter	Approximate Blood Volume (mL)																				
Chemistry/Hematology[b]	5.5	5.5		5.5		5.5				5.5				5.5		5.5	5.5				5.5
Serum β-Human Chorionic Gonadotropin (β-hCG)[c]	3.5																				
Serum inhibin B, LH, FSH, testosterone in men		11														11			11		11
HIV/Hepatitis B, C Screen[d]	20																				
Blood for Genetics		8.5																			
CMV DNA PCR	6-30[e]	6	6	6	6	6	6	6	6	6	6	6	6	6	6	6	6	6 (per visit)	6	6 (per visit)	6
CMV DNA Sequence Analysis[f]																					8
Population PK		5	5	5		5		5		5		5		5		5					
Intensive PK (for subset of subjects only)			25																		
Expected Total (mL)	35-59	36	36	16.5	6	16.5	6	11	6	16.5	6	11	6	16.5	6	27.5	11.5	6 (per visit)	17	6 (per visit)	30.5

a. End of Study Therapy Visit may occur on Week 10, 11, 12, 13, or 14 depending on when study therapy was started during the 28-day post-transplant window. Therefore, not all visits will be performed in all subjects.
b. For screening, values from the subject's chart for required chemistry and hematology tests are acceptable. If not available, this testing may be performed by the central laboratory.
c. For female subjects of child bearing potential only.
d. Perform Hepatitis B, C Screen only if not previously documented within the last 90 days of screening. If hepatitis C virus antibody is positive, RNA PCR results should be provided (or, if not available, RNA PCR testing will be performed by the central laboratory). HIV antibody test results documented at any time prior to randomization of the subject will be acceptable. A copy of the report must be available. If documentation of a previous HIV test is not available, the HIV antibody test must be conducted using the central laboratory.
e. For initial screening, results of CMV DNA PCR assay performed at a local laboratory will be acceptable. From the time of transplantation until randomization, CMV DNA PCR testing will be performed once a week by the central laboratory.
f. CMV DNA Sequence Analysis to be performed only in subjects with clinically significant CMV infection.

13.0 SIGNATURES

13.1 Sponsor's Representative

TYPED NAME	SIGNATURE	DATE
________________________	__________________________	__________

13.2 Investigator

I agree to conduct this clinical trial in accordance with the design outlined in this protocol and to abide by all provisions of this protocol (including other manuals and documents referenced from this protocol). I agree to conduct the trial in accordance with generally accepted standards of Good Clinical Practice. I also agree to report all information or data in accordance with the protocol and, in particular, I agree to report any serious adverse events as defined in Section 7.0 – Assessing and Recording Adverse Events. I also agree to handle all clinical supplies provided by the Sponsor and collect and handle all clinical specimens in accordance with the protocol. I understand that information that identifies me will be used and disclosed as described in the protocol, and that such information may be transferred to countries that do not have laws protecting such information. Since the information in this protocol and the referenced Investigator's Brochure is confidential, I understand that its disclosure to any third parties, other than those involved in approval, supervision, or conduct of the trial is prohibited. I will ensure that the necessary precautions are taken to protect such information from loss, inadvertent disclosure or access by third parties.

TYPED NAME	SIGNATURE	DATE
________________________	__________________________	__________
