# Supplementary material for: A Mortality Analysis of Letermovir Prophylaxis for Cytomegalovirus (CMV) in CMV-seropositive Recipients of Allogeneic Hematopoietic Cell Transplantation
Source: Clin Infect Dis. 2019 Jun 8;70(8):1525–33. doi: 10.1093/cid/ciz490 (PMC7146004; doi:10.1093/cid/ciz490)
Supplement: ciz490_suppl_Supplementary_Materials [file ciz490_suppl_supplementary_materials.docx]

# Supplementary Figure 1. Kaplan-Meier plot of time to all-cause mortality through week 24 and post-HCT (including post-study information, FAS). Abbreviations: CI, confidence interval; KM, Kaplan-Meier.

# Supplementary Figure 2. Kaplan-Meier plot of time to all-cause mortality through week 48 post-HCT (including post-study information, FAS). Abbreviations: CI, confidence interval; KM, Kaplan-Meier.
